# Generation of a transient base-stabilised arylalumylene for the facile deconstruction of aromatic molecules†

**DOI:** 10.1039/d2sc01436j

**Published:** 2022-05-02

**Authors:** Debabrata Dhara, Arumugam Jayaraman, Marcel Härterich, Rian D. Dewhurst, Holger Braunschweig

**Affiliations:** Institute for Inorganic Chemistry, Julius-Maximilians-Universität Würzburg Am Hubland 97074 Würzburg Germany h.braunschweig@uni-wuerzburg.de; Institute for Sustainable Chemistry & Catalysis with Boron, Julius-Maximilians-Universität Würzburg Am Hubland 97074 Würzburg Germany

## Abstract

While a stable base-free arylalumylene bearing a sterically encumbered terphenyl substituent has been reported previously, we herein report that our attempts to form a base-stabilised arylalumylene bearing a relatively small terphenyl substituent and an *N*-heterocyclic carbene base led instead to a “masked” dialumene (LRAl=AlRL), self-stabilised by one peripheral aromatic group. Intriguingly, examining the behavior of this species or its transient dialumene formed from reducing the diiodoarylalane in aromatic solvents under different conditions reveals that they both decouple into the desired base-stabilised arylalumylene. This transient acyclic, dicoordinate alumylene is highly reactive, deconstructing benzene and toluene to furnish dialuminium derivatives of pentalene, providing the first example of a neutral Al^I^ compound able to deconstruct these less reactive arenes. Computational insights were also gained on the dialumene dissociation and on the mechanism of arene deconstruction by alumylene.

## Abstract 2

Attempts to form a base-stabilised arylalumylene by reducing an NHC-coordinated diiodoterphenylalane led to a masked dialumene. Reactivity studies showed it decouples to initially aimed arylalumylene, which easily deconstructs less reactive arenes.

## Graphical abstract

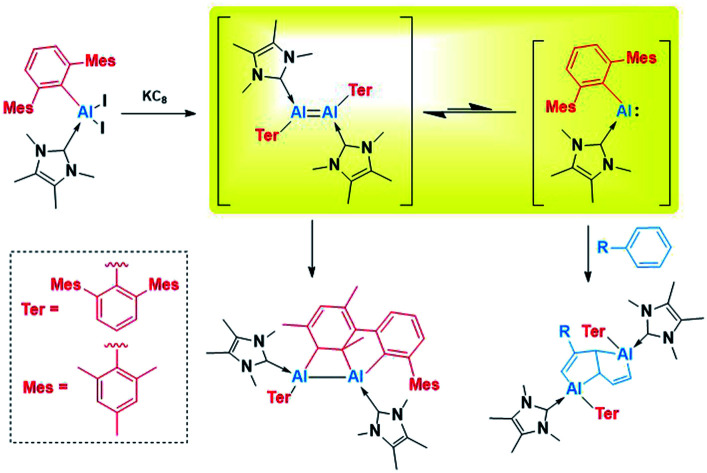

## Introduction

**Scheme 1:**
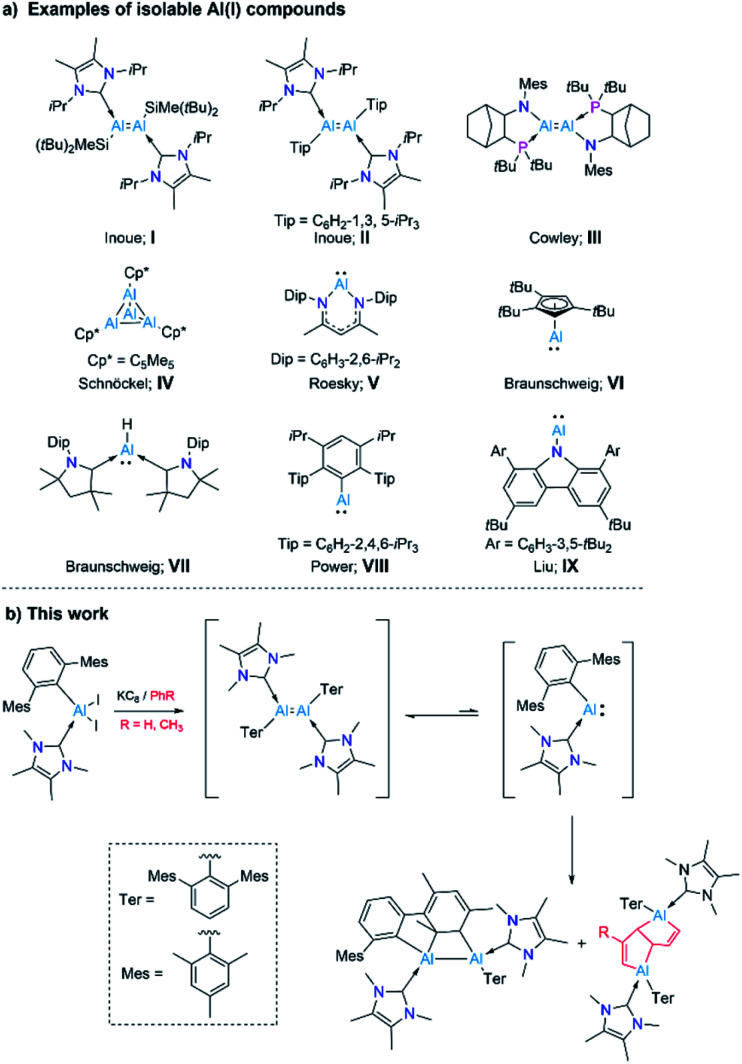
(a) Examples of isolable Al(i) species (I–IX); and (b) synthesis of an arrested dialumene and deconstruction of arenes by a masked alumylene (Al^I^) presented in this work.

Aluminium and its compounds are most commonly found in their zerovalent (metallic Al, Al^0^) and +3 oxidation states (trivalent Al, Al^III^). Thus it is not surprising that compounds of aluminium in its +1 and +2 oxidation states – generally rare and unstable species – have a marked tendency to disproportionate into Al^0^ (metal) and Al^III^ compounds.^1^ A number of Al^I^ compounds ([:AlX], [:AlH], [Al_2_O]) have been realized by cryochemical methods.^2^ However, a major development in low-valent aluminium chemistry was the near-ambient temperature isolation of an Al^II^ compound, R_2_Al-AlR_2_ (R = CH(SiMe_3_)_2_), the first Al–Al bonded molecular species, by Uhl in 1988.^3^ Several dialanes have since been isolated using a similar strategy.^4^ A further breakthrough in low-valent Al chemistry was the generation of transient dialumenes RAl=AlR (R = *m*-terphenyl, aryl, or silyl groups), featuring Al^I^ atoms, which were trapped as cycloaddition products with aromatic species.^5^ Only recently, Inoue and coworkers reported a stable, neutral, doubly NHC-stabilised dialumene (I), which contains silyl substituents (Scheme 1a).^6*a*^ Shortly, the same group presented a doubly NHC-stabilised diaryldialumene, II.^6*b*^ These two compounds have shown exciting abilities in small-molecule activation and catalysis.^6^ In 2021, Krämer and Cowley reported the third stable dialumene (III), which bears amino substituents with chelating phosphine bases.^7^ Interestingly, this species was demonstrated to exhibit a reversible dissociation behavior.

Monovalent aluminium species (Al^I^) have been known since 1991, with the isolation of the tetrameric Al^I^ species 
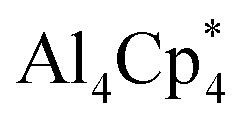
 (Cp* = pentamethylcyclopentadienyl; IV, Scheme 1a),^8^ which was shown to dissociate into its monomer [AlCp*] upon heating.^9^ However, the isolation of a Al^I^ compound with a β-diketiminato ligand by Roesky and coworkers (V, Scheme 1a) allowed access to a stable mono-Al^I^ species.^10^ In 2018, our group reported the compound [(η^5^-Cp^3t^)Al^I^] (VI, Scheme 1a) (Cp^3t^ = 1,3,5-tri-*tert*-butylcyclopentadienyl), a monomeric species that showed several interesting reactivity patterns.^11^ We also reported a parent alumylene (*i.e.*, Al^I^H) species (VII, Scheme 1a) stabilised by two cyclic (alkyl)(amino)carbenes (CAACs) which showed 36% singlet diradical character (Al^III^).^12^ While these discoveries provided mono-Al^I^ species for further reaction, they both bear further stabilising groups, meaning that neither is monocoordinate. A stunning recent discovery by Power *et al.* presented a first stable, monomeric, base-free, and monocoordinate Al^I^ species (VIII, Scheme 1a), bearing a sterically bulky terphenyl substituent.^13^ Shortly after this discovery, the groups of Liu and Hinz independently reported the synthesis of another base-free alumylene that carries a sterically hindered amino substituent (IX).^14^ We note that although various terminologies, including alanediyl,^13^ aluminylene,^14*a*^ and aluminyl,^7^ have been used in the literature for Al^I^ compounds of the type [:AlR(L)_*n*_] (*n* = 0–2), for simplicity and due to their analogy to carbenes and borylenes we use the terminology “alumylenes” for this type of compounds. In addition to these two types of Al^I^ compounds, a third form of Al^I^ species has also emerged relatively recently, namely aluminyl anions of the form [AlR_2_]^−^, which adopt diverse structures and show high Al-centered reactivity with electrophiles.^15^

Despite these developments, examples of Al^I^ species, such as monomeric alumylenes [:AlR], dialumenes ([RAl=AlR]) and aluminyl anions ([AlR_2_]^−^) are still very scarce due to their highly reactive nature and lack of general synthetic strategies.^15*b*,16^ Our research interests within low-valent aluminium chemistry have been in preparing and probing the reactivity of monomeric Al^I^ compounds. Given that a base-free arylalumylene has been isolated,^13^ while attempts to isolate an NHC-bound analogue led only to isolation of a diaryl dialumene,^6*b*^ we have been interested in developing a method to prepare an NHC-coordinated arylalumylene. We reasoned that the reduction of an NHC-stabilised dihaloalane bearing a superbulky aryl group (Ar*), *i.e.* [(NHC)AlX_2_Ar*] (Ar* = 2,6-C_6_H_3_Mes_2_, Mes = 2,4,6-Me_3_C_6_H_2,_ NHC = NHC^Me4^, X = I) might lead to a stable, monomeric alumylene of the form [(NHC)Ar*Al:] – effectively an acyclic version of Roesky's alumylene (V, Scheme 1a). This study has led instead to the discovery of highly unusual outcomes of reducing a dihaloalane: an intramolecular formal [2 + 2] cycloaddition product by dearomatization of an outlying aryl group (Mes) by the generated dialumene of the form ([(NHC)Ar*Al=AlAr*(NHC)]), and bicyclic bis(alane) products arising from the deconstruction of aromatic solvent molecules by the anticipated transient alumylene of the form [(NHC)Ar*Al:] (Scheme 1b). Insights gained through a combination of experiments and computations hint that from the reduction reaction, the initial species formed is a dialumene of the form [(NHC)Ar*Al=AlAr*(NHC)], a species responsible for the intramolecular cycloaddition product. An additional hint gained was that the diaryldialumene can unravel into its monomeric alumylene [(NHC)Ar*Al:], which engages in deconstructing the less reactive arenes benzene and toluene.

## Results and discussion

**Scheme 2:**
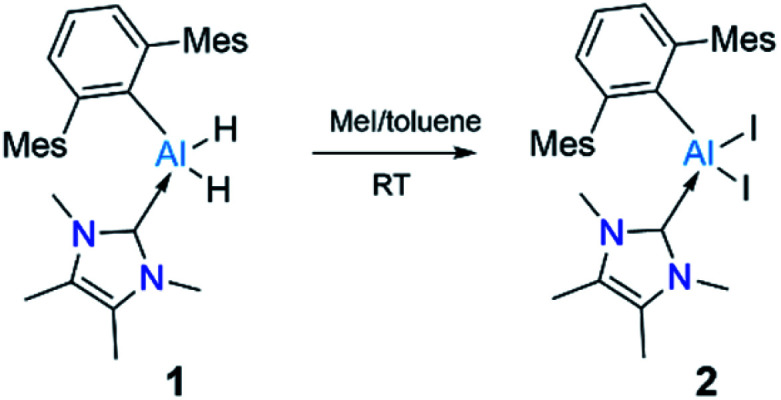
Synthesis of compound 2. Mes = 2,4,6-Me_3_C_6_H_2._

We chose a bulky terphenyl moiety (2,6-C_6_H_3_Mes_2_, Mes = 2,4,6-Me_3_C_6_H_2_) and a small *N*-heterocyclic carbene (1,3,4,5-tetramethylimidazol-2-ylidene, NHC^Me4^) for our study. The bulky terphenyl-substituted, NHC-coordinated Al^III^ diiodide precursor 2 was synthesized by iodination of the corresponding hydride, 1, with an excess of methyliodide in toluene at room temperature (Scheme 2).^17*a*^

**Fig. 1:**
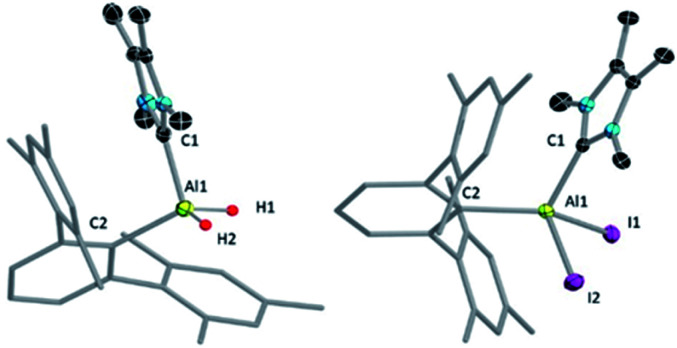
Molecular structures of 1 (left) and 2 (right) with ellipsoids at the 50% probability level. All hydrogen atoms except H1 and H2 are omitted for clarity in 1. All hydrogen atoms along with toluene (solvent of crystallization) are omitted for clarity in 2. The terphenyl substituent in 1 and 2 is depicted in wireframe style for simplicity. Selected bond lengths [Å] and bond angles [°] for 1: Al1–C1 2.040(8), Al1–C2 2.028(1); C1–Al1–C2 106.9(5); for 2: Al1–C1 2.033(6), Al1–C2 1.994(6); C1–Al1–C2 114.2(3).

The NHC^Me4^-substituted terphenylalane 1 was prepared by reacting TerLi^18^ with the corresponding carbene-stabilised alane.^17*a*^ The synthetic routes to 1 and 2 are analogous to the procedures of Inoue.^17^ Formation of 1 and 2 were confirmed by ^1^H and ^27^Al{^1^H} NMR spectroscopy. The ^1^H NMR signal of the Al-bound hydrides of 1 appeared at high field 4.04 (br, s AlH_2_, FWHM: 257 Hz) ppm, while a ^27^Al{^1^H} NMR signal was found at low field 112.4 ppm compared to that of NHC^Me4^ AlH_3_ (^1^H: 4.45 (br, s AlH_2_, FWHM: 779 Hz) ppm; ^27^Al{^1^H}: 106.5 ppm). These values match well with those of similar reported compounds.^6^ The identities of 1 and 2 as NHC-stabilised terphenylalanes were further confirmed by their solid-state structures (Fig. 1).

**Scheme 3:**
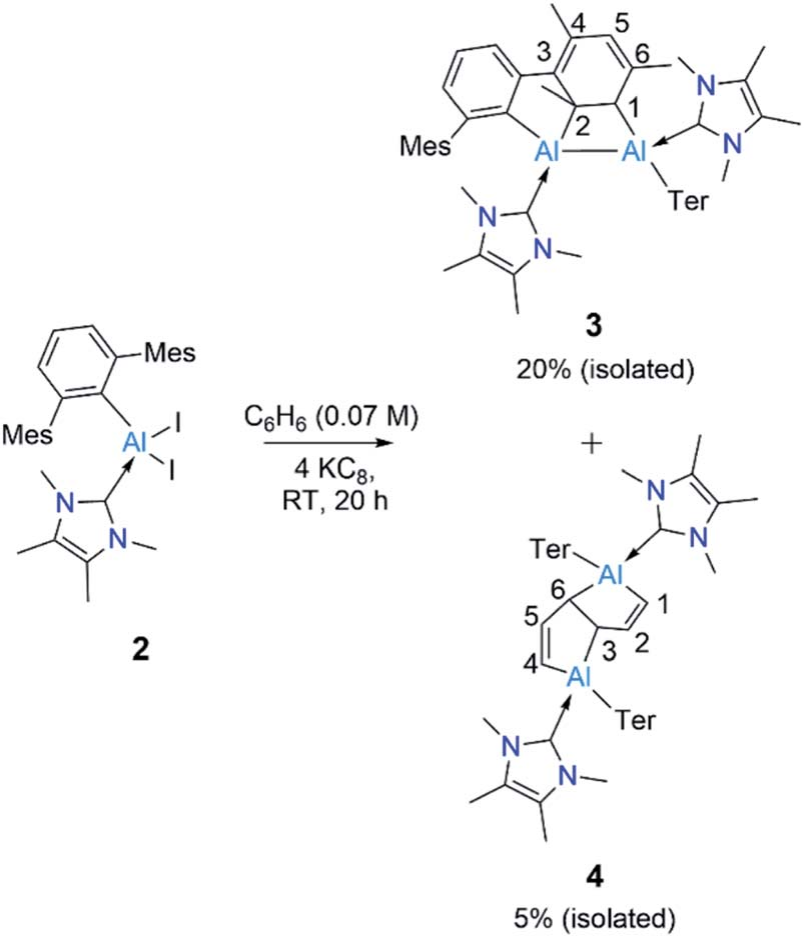
Synthesis of 3 and 4. Ter = 2,6-C_6_H_3_Mes_2_, Mes = 2,4,6-Me_3_C_6_H_2._

Reduction of 2 with four equivalents of KC_8_ at room temperature in benzene led to the compounds 3 and 4 (64% and 36% respectively, relative NMR conversion, Scheme 3). Separation of the compounds was achieved by taking advantage of their differing solubility. Repeated washing of the reaction mixture with hexanes allowed us to separate 4 (5% isolated yield, see ESI† for details). The remaining solid was dissolved in a minimum amount of benzene and allowed to evaporate slowly inside a glovebox to afford bright yellow crystals of 3 (20%, isolated yield). After extraction of 3 and 4, the remaining solution contained a mixture of the two compounds, which we could not separate further. Both 3 and 4 were characterized by multinuclear solution NMR spectroscopy and solid-state molecular structure determination. The ^1^H NMR signal for the vinylic proton of the activated mesityl group in 3 (attached to C^5^, Scheme 3) appeared at 5.14 ppm, while the signal for the allylic proton (attached to C^1^) appeared much more upfield (0.7 ppm) than those of conventional allylic protons (*ca.* 1.8 ppm). This is likely due to aluminium attachment to the C^1^ center, making the proton attached to C^1^ more hydridic. The ^13^C{^1^H} NMR spectroscopic signal of the vinylic carbon C^5^ was found at 116 ppm (as confirmed by HSQC). The ^1^H NMR spectroscopic signal for the C^1,4^ vinylic protons of 4 was found at 5.01 ppm (d, ^3^*J* = 12 Hz), in the usual range for signals of vinylic protons but slightly upfield compared to that of 3. The signal for the C^2,5^ vinylic protons was found significantly further downfield (7.32 ppm), which is in line with the ^1^H resonances of analogous protons in a similar compound reported by Crimmin.^19^ Solid samples of 4 were poorly soluble while also being unstable in benzene, partly explaining its poor isolated yield. Partial decomposition of 4 was noted after *ca.* 4 h in the presence of benzene, as indicated by ^1^H NMR spectroscopy.

**Fig. 2:**
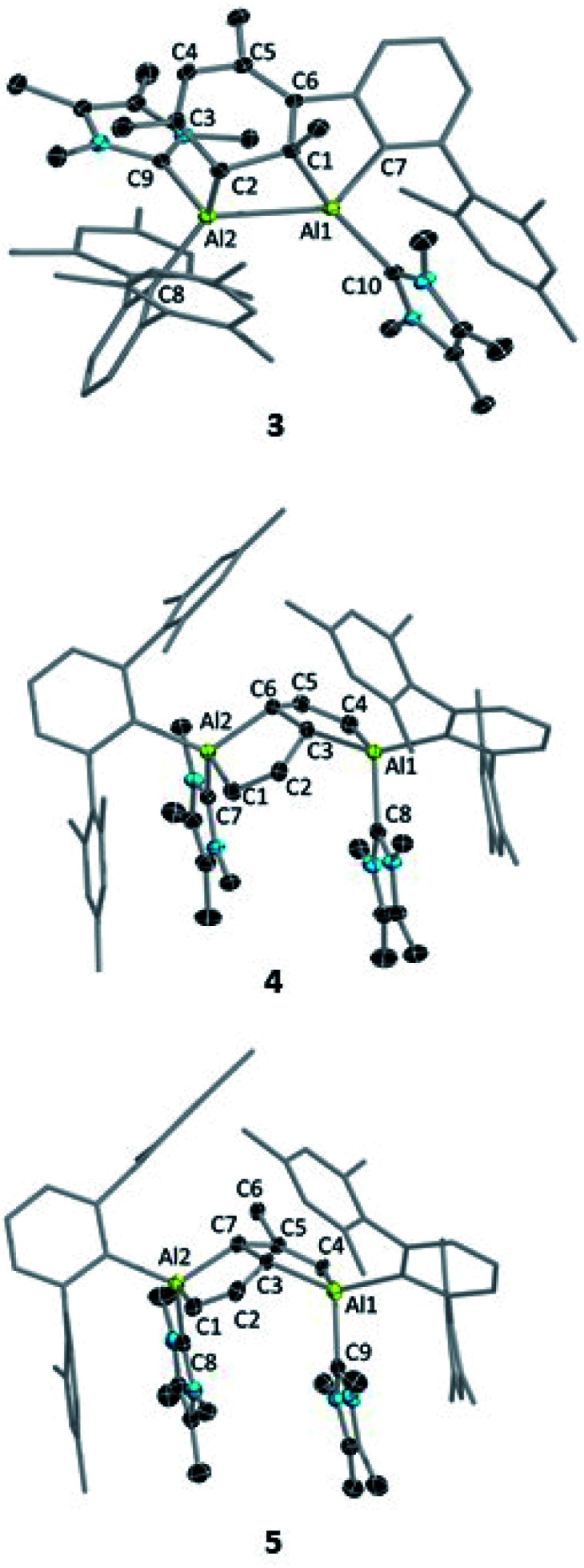
Molecular structures of 3, 4 and 5 with ellipsoids at the 50% probability level. All hydrogen atoms and two benzene molecules in 3 (solvent of crystallization), and one benzene molecule in 5 are omitted for clarity. The terphenyl substituents in all structures are depicted in wireframe style for simplicity. Selected bond lengths [Å] and bond angles [°]: for 3: Al1–Al2 2.6091(5), C3–C4 1.3520(13), C6–C5 1.3662(12), C1–C2 1.5629(12), Al1–C10 2.0828(9), Al2–C9 2.0753(10), Al1–C7 2.0062(9), Al2–C8 2.0605(9), Al2–C2 2.0849(9), Al1–C1 2.0485(9); C7–Al1–C1 91.15(4), Al1–Al2–C2 74.56(3), Al2–Al1–C1 73.82(3), Al1–C1–C2 104.64(5), Al2–C2–C1 100.93(5); for 4: Al1–C3 = Al2–C6 2.0277(12), C3–C2 = C6–C5 1.4862(17), C1–C2 = C4–C5 1.3503(18), Al2–C1 = Al1–C4 1.9591(13), Al1–C8 = Al2–C7 2.0770(13); C6–Al2–C1 = C3–Al1–C4 92.08(5), C2–C3–Al1 = Al2–C6–C5 114.75(9), C2–C3–C6–C5 130.7 (1); for 5: Al1–C3 2.089(15), Al2–C7 1.982(19), C3–C2 1.48(3), C7–C5 1.53(2), C2–C1 1.345(14), C5–C4 1.45(2), Al1–C4 1.910(15), C3–C7 1.576(15); Al1–C3–C2 115.5(11), Al2–C7–C5 126.8(12), C1–Al2–C7 91.2(6), C3–Al1–C4 92.0(5), C2–C3–C7–C5 148.1(1).

Despite the instability of the latter, X-ray quality single-crystals of 3 and 4 were obtained from saturated benzene solutions. The solid-state structure of 3 (Fig. 2) showed that one of the mesitylene rings loses its planarity (and thus also its aromaticity), forming an Al_2_C_2_ four-membered ring. The Al–Al distance in 3 is 2.6091(5) Å, significantly longer than those of the carbene-stabilised dialumene II (Al=Al: 2.4039(8) Å)^6*b*^ and the 1,2-dialuminacyclobutene [R_2_Al_2_(CSiMe_3_)_2_] (R = 2,6-C_6_H_3_Dip_2_, Dip = 2,6-C_6_H_3_*i*Pr_2_) Al–Al: 2.4946(9) Å,^20^ slightly shorter than a carbene-stabilised dialuminacyclobutane, [(NHC)(*t*Bu_2_SiMe)AlCH_2_CH_2_Al(NHC)(*t*Bu_2_SiMe)] (NHC = 1,3-diisopropyl-4,5-dimethylimidazol-2-ylidene) Al–Al 2.6503(10) Å (ref. 6a) but similar to a range of other Al–Al single-bond distances (2.5 to 2.95 Å).^16^ The four-membered Al_2_C_2_ ring deviates from planarity with an Al1–Al2–C2–C1 dihedral angle of 23.51(6)°, which matches well with previously reported Al_2_C_2_ rings.^20^

The structure of 4 (Fig. 2) exhibits two fused five-membered rings resembling a dihydropentalene structure, connected in an endo fashion (torsion angle Al1–C3–C6–Al2: 118.36(7)°; C2–C3–C6–C5: 130.7(1)°). Each ring of the dihydropentalene structure contains one tetrahedral Al^III^ center, while the carbon backbone is clearly derived from the activation of the benzene solvent. A similar dearomatized product was observed in the reaction of biphenylene with Roesky's Al^I^ complex V, reported by Crimmin *et al.*,^19^ however, this Al^I^ complex does not undergo a similar activation of benzene or toluene.

**Scheme 4:**
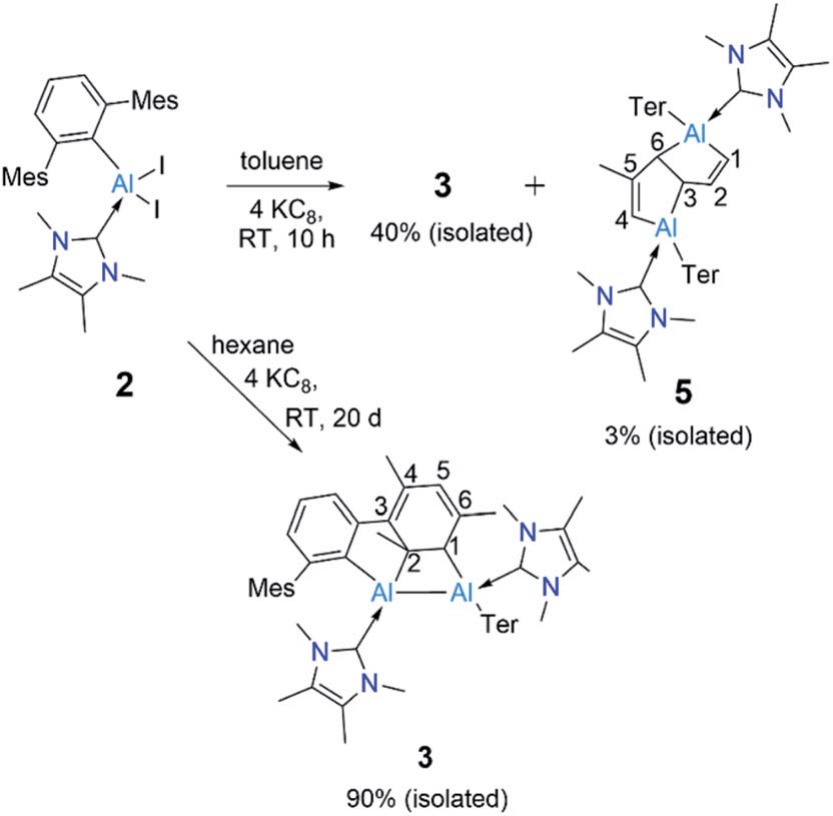
Reduction of 2 in toluene and hexanes to obtain 3 and 5. (Ter = 2,6-C_6_H_3_Mes_2_, Mes = 2,4,6-Me_3_C_6_H_2._)

The ring-opening of benzene derivatives is rare and is generally considered to be very difficult. An early work demonstrating the ring-opening of benzene by a main-group compound was reported by Meller in 1988 on the reduction of a dihaloaminoborane in a benzene/dimethoxyethane solvent mixture, which led to the formation of a diboron derivative of pentalene.^21^ In 2019, the groups of Goicoechea and Aldridge presented the reversible insertion of a reactive aluminium anion into benzene at 80 °C, the product of which was trapped using Me_2_SnCl_2_.^22^ In our case, to confirm that the solvent was the source of the backbone of the product 4, we repeated the reaction in toluene at room temperature. The ^1^H NMR spectrum of the reaction mixture showed the formation of 3 (*ca.* 68%) along with two new peaks, a doublet of doublets and a singlet in the vinyl region, indicating the formation of 5 (*ca.* 28%, Scheme 4). Compound 5 is more soluble than 3, allowing its isolation by hexane washing and crystallization (3% isolated yield). The remaining yellow solid was dried, providing 3 in 40% isolated yield. Repeating the reaction with a short reaction time (*ca.* 10 h) and starting at −78 °C led to greater selectivity for 3, allowing its isolation in 70% yield. The ^1^H NMR spectrum of 5 exhibited two characteristic signals for the vinyl protons attached to C^1^ and C^4^ at 5.01 ppm (dd, ^4^*J* = 2 Hz, ^3^*J* = 12 Hz) and 4.83 ppm (s), respectively. The signal for the proton attached to C^2^ was found further downfield at 7.34 ppm as doublet-of-doublets (^3^*J* = 3 Hz (coupling with bridging proton, confirmed by COSY), ^3^*J* = 12 Hz (coupling with vinylic proton, confirmed by COSY)). Like 4, 5 has a similar endo geometry but unlike 4 it has no center of inversion as observed from its solid-state structure (Fig. 2). The two localized C–C double bonds significantly differ in length: the length of C2–C1 (1.345(14) Å) is closer to that of the C2–C1 bond distance of 4, whereas the C5–C4 distance in 5 (1.45(2) Å) is longer than typical C–C double bonds. The structures of 4 and 5 indicate that they arise from the deconstruction of benzene and toluene, respectively, by a low-valent aluminium species. To further support this, and to provide 3 more selectively, we performed the reduction of 2 in hexane (Scheme 4). As expected, this reaction resulted in 3 almost exclusively according to ^1^H NMR of the crude reaction mixture and afforded it in 90% isolated yield.

Compound 3 can be thought of as a pseudo-dialumene internally stabilised by [2 + 2] cycloaddition between an Al=Al bond and the π system of a peripheral mesitylene ring. Earlier studies by Power^5*a*^ and Tokitoh^5*b*^ independently disclosed the synthesis of bicyclic masked base-free dialumenes formed by [4 + 2] cycloaddition of toluene or benzene with the Al=Al bond of intermediate dialumenes. It is worth mentioning that while [2 + 2] cycloaddition is symmetry forbidden, it can occur with bent double bonds between heavier main group elements,^23^ which show “charge-shift” bonding having substantial amounts of second-order Jahn–Teller effects. Doubly base-coordinated compounds with bent Al=Al bonds are also an exception for the symmetry forbidden [2 + 2] cycloaddition reactions, as several examples of addition reactions have recently been reported with substrates such as alkynes, alkenes and CO_2_.^6^ However, thus far, there is no precedence demonstrating an addition reaction with arenes. Compound 3 serves as the first example in this regard, though the addition is an intramolecular one.

We presume that dialumene 6 is the initial species formed from the two-fold reduction of 2 before it transforms to 3. We suggest this because dialumene II was the main product of reducing an NHC-coordinated diiodoalane supported by the relatively small aryl group 2,4,6-tri-*iso*-propylphenyl (Tipp).^6*b*^ To ascertain if dialumene 6 is the initially formed species, attempts were made to halt the reduction at the formation of the diiodoalane intermediate [(NHC)(I)Ar*Al–AlAr*(I)(NHC)] by employing substoichiometric amounts of KC_8_ (1.5 equiv) at room temperature. Calculations performed at the PBE1PBE-D3BJ/Def2TZVP/SMD(benzene)//PBE1PBE-D3BJ/Def2SVP level of theory on this reaction hinted that diiododialane formation from the homocoupling of the early NHC-stabilised iodo(aryl)aluminyl radical [(NHC)(I)Ar*Al]˙ is highly likely, as the coupled species is markedly stable (Δ*G* = −25.2 kcal mol^−1^). Unfortunately, experiments invariably formed 3 and the starting material in a ratio *ca.* 2 : 3, contrasting the results of reducing a base-free diiodoarylalane, wherein the diiododialane is the primary product.^5*a*^ This may have resulted from reducing the *in situ* generated diiododialane to dialumene 6 more rapidly than the starting material 2.

**Scheme 5:**
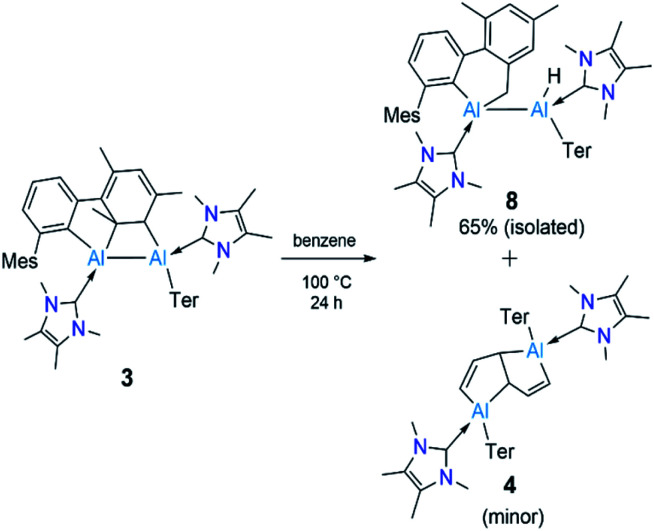
Thermolysis of 3 to form 8 and 4. Ter = 2,6-C_6_H_3_Mes_2_, Mes = 2,4,6-Me_3_C_6_H_2._

**Fig. 3:**
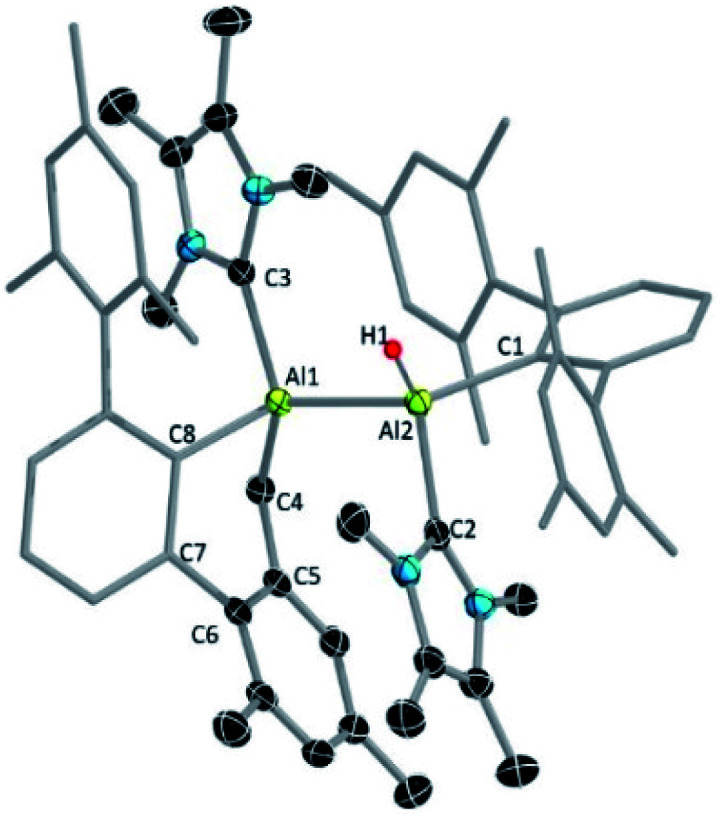
Molecular structure of 8 with ellipsoids at the 50% probability level. All but one hydrogen atom and a single hexane molecule (solvent of crystallization) are omitted for clarity. The terphenyl substituent and residues of the C–H activated terphenyl are depicted in wireframe style for simplicity. Selected bond lengths [Å] and bond angles [°]: Al1–Al2 2.6228 (5), Al1–C4 2.0331(14), Al1–C3 2.0866(14), Al1–C8 2.0449(14), Al2–C1 2.0638(14), Al2–C2 2.07840(15); Al1–C4–C5 105.69(9), C8–Al1–C4 90.90(6), C3–Al1–Al2 113.22(4), C3–Al1–C8 107.19(6), C2–Al2–C1 105.12(6), C2–Al2–Al1–C3 173.03(7), C8–Al1–Al2–C1 178.57(7).

Since compound 3 can be viewed as a masked dialumene, we anticipated that simple heating might allow the generation of a dialumene. However, no decomposition or changes were observed during a variable-temperature NMR experiment on a benzene solution of 3 from 25 °C up to 100 °C. Prolonged heating at 100 °C (24 h) led to the formation of 8, the product of a formal intermolecular C–H activation (Scheme 5), along with a small batch of crystals of 4. The reduction of 2 in benzene at 100 °C also provided a similar outcome, with 8 as the major product. The ^1^H NMR spectrum of 8 showed a distinctive, broad signal (FWHM = 129 Hz) at 4.41 ppm corresponding to the Al-bound hydride, which is somewhat downfield of that of aluminium hydride 1 but in the range of similar carbene-stabilised dialumene hydrides.^6^ A broad signal (FWHM = 75 Hz) at 1.68 ppm was also observed corresponding to the newly formed methylene group. Interestingly, in the solid-state structure of 8 (Fig. 3), the Al–Al bond (Al1–Al2 2.6228(5) Å) was found to be significantly longer than that of 3 (Al–Al: 2.6091(5) Å (Fig. 3)).

**Fig. 4:**
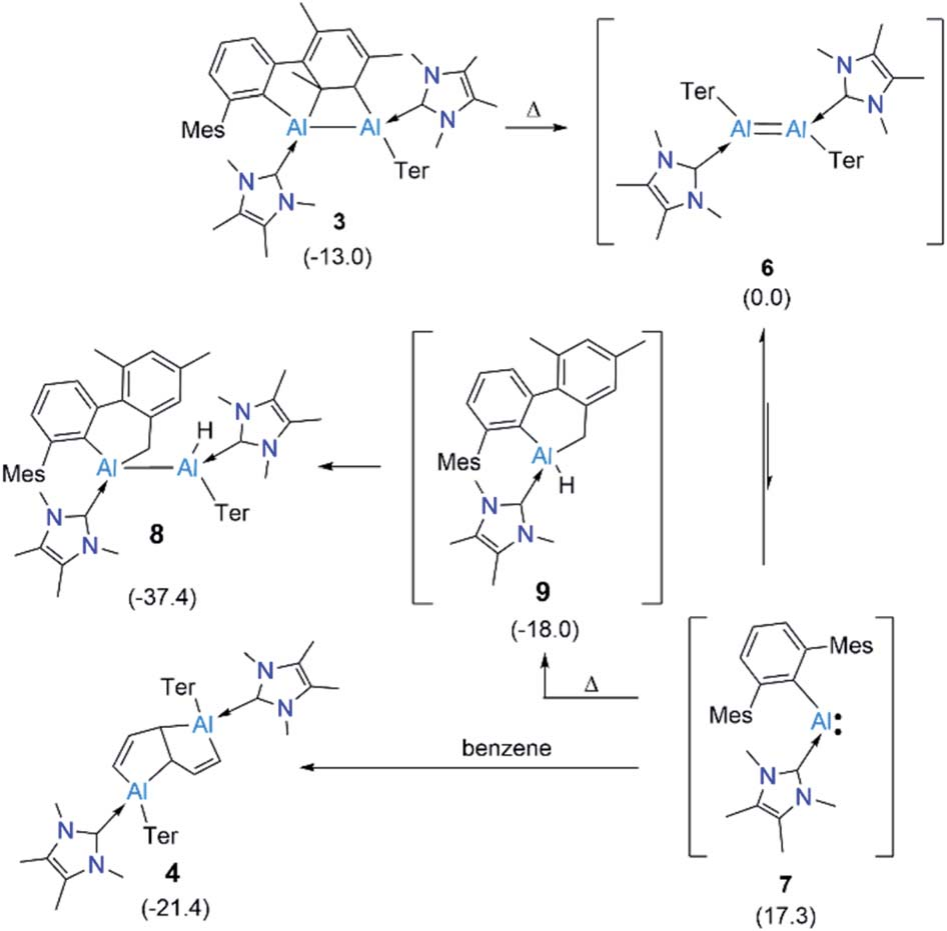
Plausible route to the C–H activation product 8 and the benzene-deconstructed product 4 from masked dialumene 3. Ter = 2,6-C_6_H_3_Mes_2_, Mes = 2,4,6-Me_3_C_6_H_2._ Free energy differences (Δ*G*), in kcal mol^−1^, computed among all species are given in parentheses.

The outcome of this experiment suggests that upon heating, 3 first reverts to 6, which then transforms to 8 (Fig. 4). In other words, 3 is a kinetic product and 8 is a thermodynamic product from 6. The thermodynamics computed for 3, 6 and 8 are in line with our experimental observation that 3 is significantly more stable than 6 (Δ*G* = −13.0 kcal mol^−1^), while 8 is substantially more stable than 3 (Δ*G* = −24.4 kcal mol^−1^) and 6 (Δ*G* = −37.4 kcal mol^−1^). It can be assumed that 3 forms *via* an intramolecular [2 + 2] cycloaddition in 6. For 8, however, given that the added methylene and hydride functionalities in 8 are *trans*-oriented, a straightforward C–H activation across the Al=Al bond in 6 may not be viable as it can only lead to a *cis*-isomer of 8. Conversion of this *cis*-isomer to the obtained *trans*-isomer *via* rotation of the Al–Al single bond may not be feasible due to sterics. Thus, we suggest 8 forms *via* an intramolecular C–H activation in alumylene 7 that was generated from the dissociation of dialumene 6, leading to a hydridoalane intermediate (9), followed by a formal insertion of another 7 into the Al–H bond in 9 (Fig. 4). Only a minor amount of the pentalene product 4 obtained from the same thermal reaction signals that the dialumene 6 generated from the reduction reaction somehow reacts with benzene before self-stabilising to form 3. Even for this reaction, we propose the involvement of alumylene 7*via* dissociation of 6 (Fig. 4).

Compelling evidence for the dissociation of compounds with heavier main group multiple bonds under mild conditions has been known for nearly five decades.^24^ However, only very recently, a report by Krämer and Cowley demonstrated the reversible dissociation of a doubly base-stabilised dialumene, III, for which the dissociation energy (Δ*G*_diss_) was calculated to be small (7.1 kcal mol^−1^).^7^ The Δ*G*_diss_ computed for dialumene 6 is 17.3 kcal mol^−1^, which is lower than the Δ*G*_diss_ of dialumene II (29.8 kcal mol^−1^). This suggests 6 can easily dissociate into 7 at room temperature.§§The ground state for alumylene 7 is singlet (Δ*G*_singlet–triplet_ = 17.0 kcal mol^−1^). Experimental evidence for the dissociation of 6 comprises: (i) a low yield of 5 and a high yield of 3 when the reduction was carried out at low temperatures in toluene (*vide supra*). The low yield of 5 can be primarily attributed to suppressing the dissociation of 6 into 7 at low temperatures; and (ii) conducting the reduction in a much more dilute benzene solution (0.02 M), for which the ^1^H NMR spectrum showed the formation of a greater proportion of 4 (65%) than 3 (35%).¶¶Note that the proportion mentioned is only a relative ratio as per ^1^H NMR spectroscopic data, and it is not scaled against any external standard. Under these dilute conditions, alumylene 7, which is dissociated from 6 can persist long enough to react with benzene to form the deconstruction product 4. From comparing the outcomes of the reduction of 2 at two different temperatures and that of the elevated temperature reaction of 3, it becomes evident that the room-temperature reduction reaction is the optimal condition to obtain arene deconstruction products in good yields.

**Fig. 5:**
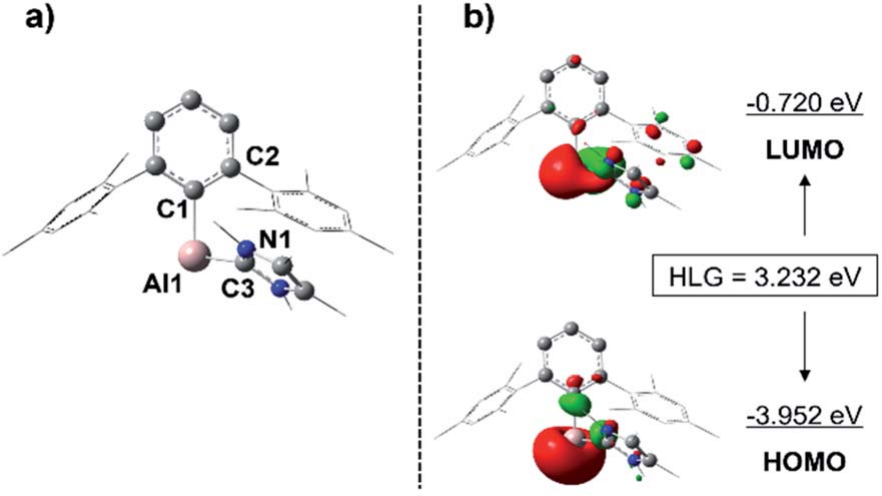
Computed structure (a) and frontier orbitals (±0.05 isovalue) (b) of alumylene 7. HLG: HOMO–LUMO gap. Hydrogens are omitted for clarity. Mesityl groups of the terphenyl substituent and the methyl groups of the NHC are depicted in wireframe style for simplicity. Selected bond lengths [Å] and bond angles [°]: Al1–C1 2.072, Al1–C3 2.128, C1–Al1–C3 96.4, C2–C1–Al1–C3 69.2, N1–C3–Al1–C1 61.6.

Given that a stable base-free arylalumylene has been isolated recently, and this does not seem to react with benzene,^13^ the fact that our base-stabilised aryl alumylene 7 is transient and tends to react with benzene and toluene suggests the presence of a crucial disparity in the frontier orbitals between base-stabilised aryl alumylenes and their respective base-free versions. The computed structure of 7 and its frontier orbitals are shown in Fig. 5. Analogous data for the corresponding base-free alumylene is provided in the ESI (Fig. S34†). The frontier orbitals of 7 display that the HOMO and LUMO are both located on the aluminium center and that the HOMO–LUMO gap (HLG) is 3.232 eV (Fig. 5), which is a significantly decreased energy gap than that found in its base free alumylene (HLG = 3.818 eV, Fig. S34†). Importantly, NHC coordination raises the HOMO energy. This is in accord with the proposal by Liu^25^ that occupation of the LUMO of free alumylenes is a prerequisite to altering the HOMO energy and enhancing their reactivity.

**Fig. 6:**
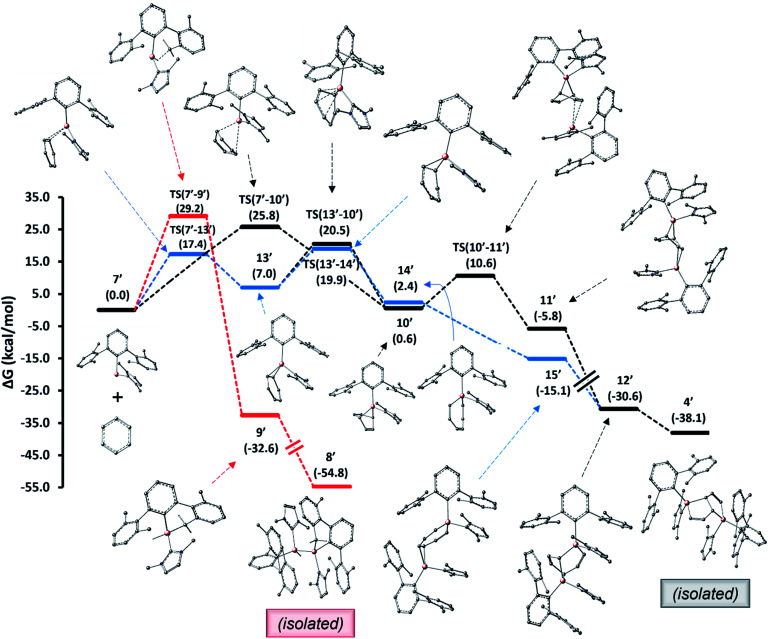
Mechanisms computed for the formation of benzene deconstruction product 4 (black and blue profiles) and the C–H activation product 8 (red profile) from alumylene 7. Free energies in parentheses are in kcal mol^−1^.

For the pentalene product 4 formed from alumylene 7 and benzene, two mechanistic pathways, as shown in Fig. 6, have been conceived and explored computationally. We utilized a truncated terphenyl group for these calculations where the mesityl groups were replaced with 2,6-dimethylphenyl groups and NHC^Me4^ was replaced by NHC^Me2^ (commonly known as IMe).||||A few mechanistic steps computed on both pathways using the full experimental system displayed similar energetics to the truncated model, suggesting that the model used can reliably represent the pathways followed by the experimental system. Note that the identification numbers of the computational model compounds are derived from their experimental counterparts, with the addition of a prime symbol. The first pathway entails a (1 + 4) cycloaddition between alumylene and benzene, followed by a (1 + 2) cycloaddition by the second alumylene and subsequent transformation (black profile). This pathway follows the mechanistic steps proposed by Crimmin *et al.* to deconstruct biphenylene by Roesky's alumylene V.^19^ The second pathway involves an initial formal insertion of alumylene into the aromatic C–C bond of benzene, followed by a second alumylene addition and subsequent transformation (blue profile). The insertion route of the latter pathway was assumed from the report of Aldridge *et al.*, which demonstrates the reversible insertion of an anionic Al^I^ compound into the benzene ring.^22^ The mechanistic study results reveal that both pathways are feasible under the employed reaction conditions and that they may both be (almost equally) competing to form the pentalene product 4.

The computed pathways show that the initial process involved in the reaction of alumylene 7′ with benzene is a [1 + 2] cycloaddition, leading to the alumirane intermediate 13′. Intriguingly, this intermediate presents similar kinetics and thermodynamics in the next stage to form the bicyclic intermediate 10′ and the insertion intermediate 14′. This paves the way for divergent pathways for the arene deconstruction reaction. Forming 10′ from 7′ and benzene directly *via* the [1 + 4] cycloaddition transition state TS(7′–10′), as proposed by Crimmin for the biphenylene deconstruction,^19^ can be less regarded, as this route holds a relatively large activation barrier. As intermediates 10′ and 14′ lie only slightly uphill from their starting materials, 7′ and benzene, and hold easily attainable, reversible activation barriers, we propose they form reversibly from their starting materials. In contrast, the C–H activation intermediate 9′ (red profile), which we presumed to form at high temperatures from 7′ and to be responsible for the formation of 8′, while having a larger barrier than predicted for forming 10′ and 14′, lies significantly lower in energy from its starting material 7′; thus, the reversibility of this step is less likely. These disparities explain why 8 was the major product when the reduction was carried out at high temperatures and when the benzene solution of 3 was heated.

## Conclusions

We report that reducing an NHC-stabilised diiodoalane bearing a sterically encumbered aryl substituent, 2,6-dimesitylphenyl, in different hydrocarbon solvents leads to different outcomes. In hexanes, a highly unusual self-stabilised dialumene species was the product, instead of the anticipated acyclic, dicoordinate alumylene (Al^I^). In benzene or toluene, the primary product was a dialuminadihydropentalene arising from C–C bond activation and deconstruction of the respective arene solvent. Subsequent probing through experiments and DFT calculations showed that the self-stabilised dialumene and its dialumene precursor dissociate to form the initially-sought alumylene before reacting with benzene and toluene. This reactivity of the transient alumylene suggests that it is more reactive than the previously reported di- and monocoordinate Al^I^ species of Roesky and Power, respectively. We envisage that careful tuning of the aryl and base groups could enable isolation of a similar base-stabilised alumylene and allow the full exploitation of the fascinating reactivity of this family of compounds.

## Data availability

Full experimental and computational details are provided as part of the ESI.†

## Author contributions

H. B. supervised the project. D. D. carried out the synthetic work. D. D. and M. H. carried out the X-ray crystallographic analyses. A. J. carried out the computational studies. D. D., R. D. D. and A. J. prepared the manuscript. D. D. and A. J. prepared the ESI. All authors discussed the results and commented on the manuscript.

## Conflicts of interest

There are no conflicts to declare.

## Supplementary Material

SC-013-D2SC01436J-s001

SC-013-D2SC01436J-s002
