# Supplementary material for: Generation of a transient base-stabilised arylalumylene for the facile deconstruction of aromatic molecules
Source: Chem Sci. 2022 May 2;13(19):5631–8. doi: 10.1039/d2sc01436j (PMC9116349; doi:10.1039/d2sc01436j)
Supplement: SC-013-D2SC01436J-s001 [file SC-013-D2SC01436J-s001.pdf]

Electronic Supplementary Material (ESI) for Chemical Science.
This journal is © The Royal Society of Chemistry 2022

Electronic Supplementary Information

Generation of a transient base-stabilised arylalumylene for the facile deconstruction of aromatic molecules

Debabrata Dhara,[a,b‡] Arumugam Jayaraman,[a,b‡] Marcel Härterich,[a,b] Rian D. Dewhurst[a,b] and Holger Braunschweig[a,b*]

[a] Institute for Inorganic Chemistry, Julius-Maximilians-Universität Würzburg, Am Hubland, 97074 Würzburg, Germany

[b] Institute for Sustainable Chemistry & Catalysis with Boron, Julius-Maximilians-Universität Würzburg, Am Hubland, 97074 Würzburg, Germany

‡ These authors contributed equally.

Table of Contents

1. Experimental details 3

1.1. General considerations 3

1.2. Synthetic procedures 4

1.3. NMR spectra 14

1.4. UV-Vis spectra 30

1.5. Crystal structure determination 31

2. Computational chemistry 40

2.1. Computational details 40

2.2. Homocoupling of the radical [(NHC)Ar*Al·] 41

2.3. Free energy differences among selected experimental compounds 46

2.4. Optimized structures, frontier orbitals and dissociation of dialumenes **6** and **II** 62

2.5. Frontier orbitals of alumylene **7** and its base-free version 73

2.6. Mechanisms explored for the formation of benzene deconstruction product and C-H activation product using model alumylene **7’** 76

2.7. First few mechanistic steps explored for the formation of benzene deconstruction product and C-H activation product using the experimental alumylene **7** 111

3. References 129

1. Experimental details

1.1. General considerations

All manipulations were performed under an atmosphere of dry argon using glovebox or standard Schlenk line techniques. Deuterated solvents were dried over 4 Å molecular sieves and degassed by three freeze-pump-thaw cycles. All other solvents were dried by distillation from appropriate drying agents under an argon atmosphere and stored under argon over activated 4 Å molecular sieves. All NMR spectra were obtained from a Bruker Avance III HD 300 NMR spectrometer ($^{13}C\{^1H\}$: 75.5 MHz), from a Bruker Avance I 400 NMR spectrometer (^1H: 400.6 MHz, $^{13}C\{^1H\}$: 100.6 MHz) at 298 K unless otherwise stated. Chemical shifts (δ) are provided in ppm and internally referenced to the carbon nuclei ($^{13}C\{^1H\}$, residual protons (^1H) of the solvent. ^{27}Al NMR spectra were referenced to external $Al(NO_3)_3$. UV/Vis absorption spectra were measured on a METTLER TOLEDO UV/Vis-Excellence UV5 spectrophotometer. Solid-state IR spectra were recorded on a Bruker FT-IR spectrometer ALPHA II inside a glovebox. Microanalyses (C, H, N, S) were performed on an Elementar Vario MICRO cube elemental analyzer. High-resolution mass spectrometry (HRMS) data were obtained from a Thermo Scientific Exactive Plus spectrometer. Aluminium hydride *N*,*N*-dimethyl ethyl amine complex solution was purchased from Sigma-Aldrich / Merck and used without further purification. Iodomethane was purchased commercially, dried over CaH_2, and degassed via the freeze-pump-thaw method. NHC^{Me4} (1,3,4,5-tetramethylimidazolin-2-ylidene)[1] and KC_8[2] were synthesized according to the literature procedures. Melting points were determined in closed NMR tubes under argon atmosphere and are reported without correction. We note that both elemental analyses and HRMS were carried out for all new compounds, but in some cases, they decomposed too rapidly and only one of these analyses was possible.

1.2. Synthetic procedures

Synthesis of $NHC^{Me4}\cdot AlH_3$

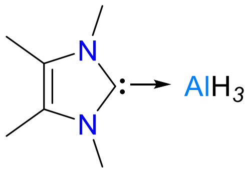

$NHC^{Me4}\cdot AlH_3$ was synthesized by a modified literature procedure.[3] **NHC^{Me4}** (2.00 g, 16.1 mmol) was added to a dry Schlenk flask and the flask was cooled to –78 ºC. Aluminium hydride *N*,*N*-dimethylethylamine complex solution (32.2 mL, 16.1 mmol, 0.5 M in toluene) was added to the flask. After addition, the solution was stirred at –78 °C for 1 h, followed by stirring for 2 h at room temperature. The solution was then evaporated to dryness and the residue was washed with 10 mL of cold dry hexanes to obtain a white powder. Isolated yield: 2.35 g (95%). ^{1}H NMR (400 MHz, C_6D_6, 298 K): δ = 1.09 (s, 6H, CH_3-C-C-CH_3, NHC^{Me4}), 3.15 (s, 6H, N-CH_3), 4.45 (br, s AlH_2, FWHM: 779 Hz) ppm. $^{13}C\{^{1}H\}$ NMR (100 MHz, C_6D_6, 298 K): δ = 8.1 (2C, CH_3-C-C-CH_3, NHC^{Me4}), 34.0 (2C, N-CH_3, NHC^{Me4}), 125.7 (2C, CH_3-*C*-*C*-CH_3, NHC^{Me4}) ppm. $^{27}Al\{^{1}H\}$ NMR (78.2 MHz, C_6D_6, 298 K): δ = 106.5 (br) ppm.

Note: Traces of unknown impurities were detected in the ^{1}H NMR spectrum, however, the product was nevertheless used for the next step.

Synthesis of 1

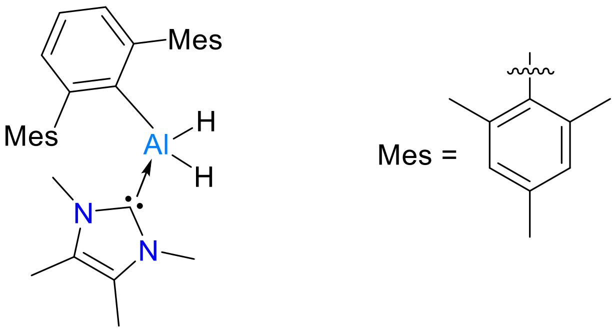

1

$NHC^{Me4}\cdot AlH_3$ (1.00 g, 6.49 mmol) and **$TerLi\cdot Et_2O$** (3.20 g, 8.05 mmol; Ter = 2,6-$C_6H_3Mes_2$, Mes = 1,3,5-$C_6H_2Me_3$) were added to a dry Schlenk flask, and the flask was cooled to –78 ºC followed by addition of 200 mL of toluene. The reaction mixture was then warmed to room temperature for 3 h, followed by 12 h of stirring at room temperature. After completion of the reaction (confirmed by ^{1}H NMR spectroscopy) the volatiles were evaporated and the resulting solid was washed with 20 mL of cold hexanes. X-ray quality crystals of the product were grown from a saturated toluene solution at –20 ºC overnight. Yield: 2.1 g (70%). M.P.: > 200 °C; ^{1}H NMR (400 MHz, C_6D_6, 298 K): δ = 1.15 (s, 6H, C*H*$_3$-C-C-C*H*$_3$, NHC^{Me4}), 2.13 (s, 6H, C*H*$_3$, Mes), 2.31(s, 12H, C*H*$_3$, Mes), 2.91 (s, 6H, N-C*H*$_3$, NHC^{Me4}), 4.04 (br, s Al*H*$_2$, FWHM: 258 Hz), 6.76 (s, 4H, Ar-*H*), 7.08 (s, 1H, Ar-*H*), 7.407 (t, ^3J = 7.6 Hz, 1H, Ar-*H*) ppm. ^{13}C{^{1}H} NMR (100 MHz, C_6D_6, 298 K): δ = 8 1 (2C, *C*H$_3$-C-C-*C*H$_3$, NHC^{Me4}), 21.6 (4C, *C*H$_3$, Mes), 21.9 (2C, *C*H$_3$, Mes), 34.2 (N-*C*H$_3$ from NHC^{Me4}), 124.5 (2C, CH$_3$-*C*-*C*-CH$_3$), 127.0 (2C, Ar-*C*H), 127.9 (1C, Ar-*C*H), 128.3 (3C, Ar-*C*H), 128.7 (1C, Ar-*C*H), 135.2 (1C, Ar-C_q)), 136.6 ((1C, Ar-C_q), 144.5 (1C, Ar-C_q), 151.8 (1C, Ar-C_q). *We did not find peaks for the carbene carbon or the quaternary carbon nuclei attached to aluminium due to quadrupolar broadening by the Al nucleus*. ^{27}Al{^{1}H} NMR (78.2 MHz, C_6D_6, 298 k): δ = 112.4 (br) ppm. FT-IR (KBr, cm^{-1}): ῡ (cm^{-1}) = 3035 (w), 2961 (w), 2906 (w), 2860 (w), 1770 (s, Al-*H*), 1750 (s, Al-*H*), 1443 (ms), 1231 (w), 1158 (w), 1102 (mw), 1028 (mw), 844 (m), 771 (s), 716 (vs), 651 (s), 568 (m). Elemental analysis: calcd. for ($C_{31}H_{39}AlN_2$): C, 79.79; H, 8.42; N, 6.00; found: C, 79.68; H, 8.31; N, 6.02. HRMS LIFDI for $[C_{31}H_{39}AlN_2]^+$ = $[M]^+$: calcd. 466.2929; found 466.2920.

Synthesis of 2

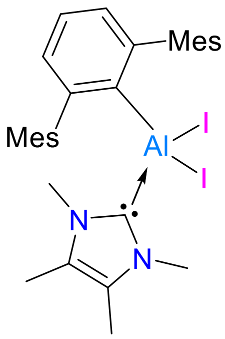

2

2 was synthesized by a modified literature procedure.[4] **1** (1.10 g, 2.30 mmol) was taken in a dry Schlenk and dissolved in 200 mL toluene. The mixture was then cooled to 0 ºC followed by addition of iodomethane (1 mL, 16.5 mmol). It was afterward fitted with an oil bubbler to allow release of byproduct methane and stirred for 12 h at room temperature. After completion of the reaction (as confirmed by ^{1}H NMR spectroscopy), the volatiles were evaporated and solid was washed with 30 mL of cold hexanes to obtain white powder of the product. Yield: 1.2 g (80%). X-ray quality single crystals were grown from a saturated toluene solution at –30 ºC overnight. **M.P.:** 190 °C (color change starts at 160 °C, decomposed); ^{1}H NMR (400 MHz, C_6D_6, 298 K): δ = 1.10 (s, 6H, C*H*$_3$-C-C-C*H*$_3$, NHC^{Me4}), 2.16 (s, 6H, s, 6H, C*H*$_3$, Mes), 2.24 (s, 12H, C*H*$_3$, Mes), 3.03 (s, 6H, N-C*H*$_3$, NHC^{Me4}), 6.76 (s, 4H, Ar-*H,* Mes), 6.94 (s, br, 1H, Ar-*H*), 6.96 (s, br, 1H, Ar-*H*), 7.28 (t, ^{3}J = 8 Hz, 1H, Ar-*H*) ppm. ^{13}C{^{1}H} NMR (100 MHz, C_6D_6, 298 K): δ = 8.2 (2C, *C*H$_3$-C-C-*C*H$_3$, NHC^{Me4}), 21.6 (2C, *C*H$_3$, Mes), 21.8 (4C, *C*H$_3$, Mes), 36 7 (N-*C*H$_3$ from NHC^{Me4}), 125.6 (2C, CH$_3$-*C*-*C*-CH$_3$), (1C, Ar-*C*H), 128.9 (3C, Ar-*C*H), 129.1 (2C, Ar-*C*H), 129.5(1C, Ar-*C*H), 134.5 (1C, Ar-*C*$_q$), 136.5 (1C, Ar-*C*$_q$), 137.3 (1C, Ar-*C*$_q$), 142.3 (1C, Ar-*C*$_q$), 151.5(1C, Ar-*C*$_q$) ppm. *We did not find peaks for the carbene carbon or quaternary carbon nucleus attached to aluminium, or any* ^{27}*Al{*^{1}*H} NMR signal, due to quadrupolar broadening by the Al nucleus.* FT-IR (KBr, cm^{-1}): ῡ (cm^{-1}) = 2958 (mw), 2924 (mw), 2855 (w), 1676 (w), 1622 (w), 1552 (w), 1442 (s), 1372 (s), 1276 (s), 1111 (ms), 1014 (s), 861 (vs), 807 (vs), 752 (S), 683 (ms), 558 (w), 448 (s). Elemental analysis: calcd. for ($C_{31}H_{37}AlI_2N_2$): C, 51.83; H, 5.19; N, 3.90; found: C, 51.93; H, 5.19; N, 3.90.

Synthesis of 3:

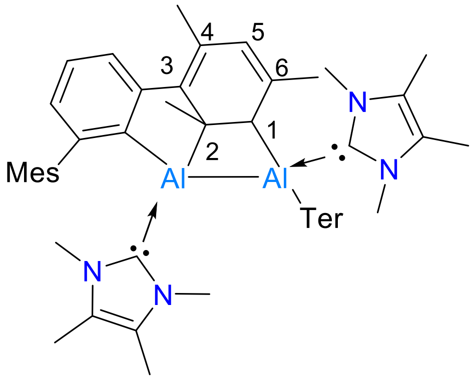

3

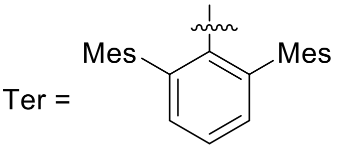

Route a: Inside a glove box, dry benzene (10 mL) was added to a vial containing **2** (0.50 g, 0.70 mmol, 0.07 M solution) and freshly prepared potassium graphite (0.38 g, 2.80 mmol) at room temperature. The mixture was stirred vigorously for 17 h at room temperature. The ^{1}H NMR spectrum of the solution showed consumption of the starting material **2** and the appearance of signals corresponding to **3** (64%) and **4** (36%). All the volatiles were removed under a high vacuum to obtain a yellow solid. The yellow solid was first extracted with hexanes (15 mL), and the remaining solid was extracted with benzene (10 mL). Overnight a white crystalline solid precipitated from the hexanes solution, filtered, and washed with cold hexanes (–40 °C) to obtain **4**. From the benzene solution, yellow crystals of **3** were precipitated after 2 d, which were separated and washed with hexanes. Yield of **3**: 65 mg (20%); yield of **4**: 15 mg (5%).

Route b: Inside a glove box, **2** (0.30 g, 0.42 mmol, 0.04 M solution in toluene) and freshly prepared potassium graphite (0.28 g, 2.10 mmol) were taken in a vial and stirred vigorously for 17 h at room temperature. A ^{1}H NMR spectrum of the mixture showed consumption of starting material **2** and the appearance of signals corresponding to **3** (67%) and **5** (28%). The solution was filtered and evaporated to obtain a yellow solid, extracted with hexanes (7 mL × 2), and left to crystallize at –35 ºC. The remaining solid was then extracted with 10 mL of benzene, concentrated to ca. 5 mL, and then left to crystallize at RT. The white crystalline compound **5** precipitated from hexanes overnight and was washed with cold (–40 ºC) hexanes. Slow evaporation of the benzene solution gave bright yellow crystals of **3**. Yield of **3**: 78 mg (40%); yield of **5**: 6 mg (3%).

Note: The remaining benzene solution after separation of ***3*** *contains both* ***3*** *and* ***4/5****. They have similar solubilities (a few crystals of* ***3*** *are occasionally found to grow from the hexanes solution), so it is not easy to separate pure* ***3*** *and* ***4/5*** *in good yields as indicated by* ^{1}*H NMR spectroscopy.*

Route c: 2 (1.00 g, 1.39 mmol) and freshly prepared potassium graphite (0.94 g, 6.90 mmol) were added to a dry Schlenk flask, and the mixture was cooled to –78 ºC followed by the addition of 15 mL of toluene. The mixture was slowly warmed to room temperature over 4 h with continuous stirring and stirred for 6 h at room temperature. The ^{1}H NMR spectrum of the crude reaction mixture showed consumption of **2** and almost exclusively the appearance of signals of **3**. The solution was filtered and evaporated to obtain a yellow solid, which was then washed with a mixture of cold (0 ºC) benzene (1 mL) and hexanes (10 mL), providing a pure yellow solid (**3**). Yield: 450 mg (70%).

Route d: Inside a glove box, dry hexanes (15 mL) was added to a vial containing **2** (0.30 g, 0.42 mmol) and freshly prepared potassium graphite (0.28 g, 2.10 mmol) at room temperature. The reaction mixture was then stirred vigorously for 20 d at room temperature. A ^{1}H NMR spectrum of the resulting mixture showed complete consumption of the starting material **2** and the exclusive presence of signals of **3**. The solution was then evaporated, and the solid was washed with 5 mL of cold hexanes to obtain a bright yellow solid (**3**). Yield: 195 mg (90%).
M.P.: 170 °C (decomposition); ^{1}H NMR (400 MHz, C_6D_6, 298 K): δ = 0.70 (s, 1H, Al-C*H*) 1.21 (s, 3H, C*H*$_3$ attached C^2), 1.22 (s, 3H, C*H*$_3$-C-C-C*H*$_3$, NHC^{Me4}), 1.41 (s, 6H, C*H*$_3$-C-C-C*H*$_3$, NHC^{Me4}), 1.47 (s, 3H, C*H*$_3$, C*H*$_3$-C-C-C*H*$_3$, NHC^{Me4}), 1.59 (s, 3H, C*H*$_3$, Mes), 1.61 (s, 3H, C*H*$_3$, Mes), 1.79 (s, 6H, C*H*$_3$, Mes), 2.01 (s, 3H, C*H*$_3$, Mes), 2.02 ((s, 3H, C*H*$_3$, Mes), 2.20 (s, 3H, C*H*$_3$, Mes), 2.36 (s, 3H, C*H*$_3$, Mes), 2.41 (s, 3H, C*H*$_3$, Mes), 2.49 (s, 3H, C*H*$_3$, Mes), 2.61 (s, 3H, C*H*$_3$, Mes), 2.83 (s, 3H, N-C*H*$_3$, NHC^{Me4}), 2.96 (s, 3H, N-C*H*$_3$, NHC^{Me4}), 3.26 (s, 3H, N-C*H*$_3$, NHC^{Me4}), 3.29 (s, 3H, N-C*H*$_3$, NHC^{Me4}), 5.14 (s, 1H, vinylic C-*H,* C^5), 6.22 (s, 1H, Ar-*H*), 6.33 (s, 1H, Ar-*H*), 2.56 (s, 1H, Ar-*H*), 6.73 (s, 1H, Ar-*H*), 6.87-6.91 (m, 3H, Ar-*H*), 7.07-7.09 (m, 1H, Ar-*H*), 7. 28 (t, ^{3}J = 7.6 Hz, 1H, Ar-*H*), 7.34-7.36 (m, 2H, Ar-*H*), 7.72 (d, ^{3}J = 7.6 Hz, 1H, Ar-*H*) ppm. ^{13}C{^{1}H} NMR (100 MHz, C_6D_6, 298 K): δ = 8.4 (4C, *C*H$_3$-C-C-*C*H$_3$, NHC^{Me4}), 20.44 (1C, *C*H$_3$, Mes), 20.9 (1C, *C*H$_3$, Mes), 21.5 (2C, *C*H$_3$, Mes), 21.6 (1C, *C*H$_3$, Mes), 21.8 (1C, *C*H$_3$, Mes), 21.9 (1C, *C*H$_3$, Mes), 22.3 (1C, *C*H$_3$, Mes), 23.2 (1C, *C*H$_3$, Mes), 24.0 (1C, *C*H$_3$, Mes), 25.4 (1C, *C*H$_3$ attached C^2), 27.1 (1C, *C*H$_3$, Mes), 34.5 (1C, N-*C*H$_3$, NHC^{Me4}), 35.4 (1C, N-*C*H$_3$, NHC^{Me4}), 35.7 (1C, N-*C*H$_3$, NHC^{Me4}), 35.9 (1C, N-*C*H$_3$, NHC^{Me4}),

115.9 (1C, =*C*H), 123.4 (1C, Ar-*C*H), 124.5 (1C, Ar-*C*H), 126.2 (1C, Ar-*C*H), 126.6 (1C, Ar-*C*H), 127.1 (1C, Ar-*C*H), 127.2 (1C, Ar-*C*H), 127.3 (1C, Ar-*C*H), 128.0 (1C, Ar-*C*H), 128.3 (1C, Ar-*C*H), 128.5 (1C, Ar-*C*H), 128.9 (1C, Ar-*C*H), 129.7 (1C, Ar-*C*H), 121.4 (4C, CH_3-*C*-*C*-CH_3, NHC^{Me4}), 124.3 (1C, Ar-C_q), 124.7 (2C, Ar-C_q), 133.7 (1C, Ar-C_q), 133.8 (1C, Ar-C_q), 134.5(1C, Ar-C_q), 135.2 (1C, Ar-C_q), 135.3 (1C, Ar-C_q), 135.9 (1C, Ar-C_q), 136.2 (1C, Ar-C_q), 136.5 (1C, Ar-C_q), 136.5 (1C, Ar-C_q), 140.0 (1C, Ar-C_q), 145.3 (1C, Ar-C_q), 146.3 (1C, Ar-C_q), 146.5 (1C, Ar-C_q), 147.4 (1C, Ar-C_q), 149.8 (1C, Ar-C_q), 151.6 (1C, Ar-C_q), 152.9 (1C, Ar-C_q), 226.5 (2C, Ar-C_q), 244.9 (2C, Ar-C_q) ppm. The allylic carbon nucleus was obscured due to quadrupolar broadening by the Al nucleus. FT-IR (KBr, cm^{-1}): ῡ (cm^{-1}) = 1656 (w), 1616 (w), 1578 (w), 1547 (w), 1432 (s),1362 (m), 1239 (w), 1162 (w), 1108 (w), 1077 (w), 1024 (w), 954 (w), 884 (w), 854 (s), 785 (m), 730 (s). UV/Vis λ_{max} = 303, 369 nm. Elemental analysis: calcd. for ($C_{62}H_{74}Al_2N_4$): C, 80.14; H, 8.03; N, 6.03. found: C, 79.82; H, 8.17; N, 6.06. HRMS LIFDI for $[C_{62}H_{74}Al_2N_4]^{+}$= $[M]^{+}$: calcd. 928.5544; found 928.5539.

Note: The solubility of ***3*** *is not good enough to obtain a high-resolution* $^{13}C\{^{1}H\}$ *NMR spectrum. We used a 400 MHz instrument and recorded 10,000 scans, yet the S/N ratio of the* ^{13}C *nuclei did not improve.*

Synthesis of 4:

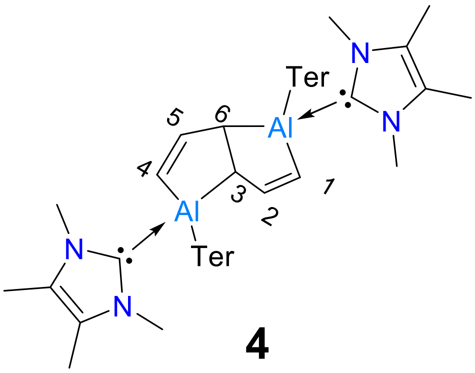

4

Compound **4** was prepared as a byproduct of the synthesis of **3** via **Route a**. X-ray quality single crystals of **4** were grown from the crude saturated reaction mixture in benzene.

Note: Although the reaction was performed in benzene, crystalline ***4*** *is not very soluble in benzene and is unstable in this solvent. The compound started to decompose within 3-4 h upon standing in* C_6D_6*. Thus, we provide only selected signals for the* ^{13}C *NMR spectroscopic data based on 2D NMR spectra.*

M.P.: 175 °C (decomposition); ^{1}H NMR (400 MHz, C_6D_6, 298 K): 1.06 (s, 2H, bridging C*H*, C^3 & C^6), 1.22 (s, 18H, 12H from CH_3-C-C-CH_3, NHC^{Me4}, 6H from CH_3 of Mes), 1.86 (s, 12H,

CH_3 of Mes), 2.29 (s, 12H, CH_3 of Mes), 2.38 (s, 12H, 6H from CH_3 of Mes, and 6H from N-CH_3 of NHC^{Me4}), 2.51 (br, 6H, N-CH_3 of NHC^{Me4}), 5.01 (d, ^3J = 12 Hz, 2H, C^{1,4}-*H*), 6.79 (s, 4H, Ar-*H*), 6.88 (s, 4H, Ar-*H*), 6.94 (s, 2H, Ar-*H*), 6.96 (s, 2H, Ar-*H*), 7.26-7.30 (m, 2H, Ar-*H*), 7.32 (br, 1H, C$^{2/5}$), 7.33 (s, 1H, C$^{2/5}$-*H*) ppm. ^{13}C{^{1}H} NMR (100 MHz, C_6D_6, 298 K) selected signals: δ = 8.5 (4C, *C*H$_3$-C-C-*C*H$_3$, NHC^{Me4}), 21.4 (1C, *C*H$_3$, Mes), 21.8 (1C, *C*H$_3$, Mes), 22.5 (1C, *C*H$_3$, Mes), 123.7 (4C, CH$_3$-*C*-*C*-CH$_3$, NHC^{Me4}), 127.7 (Ar-*C*H), 127.9 (Ar-*C*H), 128.2 (Ar-*C*H), 128.4 (Ar-*C*H), 128.5 (Ar-*C*H), 128.7 (Ar-*C*H), 128.9 (Ar-*C*H), 129.0 (Ar-C_q), 135.0 (Ar-C_q), 137.2 (Ar-C_q), 144.8 (Ar-C_q), 151.3 (Ar-C_q) ppm. FT-IR (KBr, cm^{-1}): ῡ (cm^{-1}) = 1650 (w), 1604 (w), 1546 (w), 1504 (s), 1440 (w), 1378 (m), 1217 (w), 1174 (vw), 1100 (w), 1032 (m), 946 (s), 860 (s), 804 (s), 730 (s), 607 (m), 557 (m), 502 (s), 460 (vs). Elemental analysis: calcd. for ($C_{68}H_{80}Al_2N_4$): C, 81.08; H, 8.00; N, 5.56. found: C, 80.64; H, 8.04; N, 5.43.

HRMS LIFDI for $[C_{68}H_{80}Al_2N_4]^+$ = $[M]^+$: calcd. 1006.6014; found 1006.6008

Synthesis of 5:

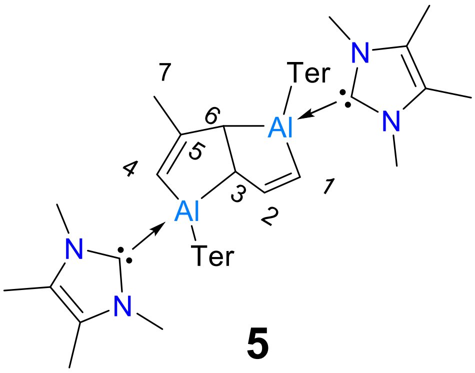

5

Compound **5** was prepared as a byproduct from the synthesis of **3** via **Route b**. X-ray quality single crystals of **5** were grown from a saturated benzene solution. **5** is relatively stable at room temperature in benzene solution. We observed an intractable decomposition product in the ^{1}H NMR spectrum after 48 h at room temperature. M.P.: 180 °C (decomposition); ^{1}H NMR (400 MHz, C_6D_6, 298 K): 1.28 (br, s, 6H, CH_3-C-C-CH_3, NHC^{Me4}), 1.31 (s, 6H, CH_3-C-C-CH_3, NHC^{Me4}), 1.42 (br, 2H, bridging C*H*, C^3 & C^6), 1.79 (s, CH_3 from C^7), 1.88 (s, 6H, CH_3, Mes), 1.94 (s, 6H, CH_3, Mes), 2.31 (s, 6H, CH_3, Mes), 2.32 (s, 6H, CH_3, Mes), 2.37 (s, 6H, CH_3, Mes), 2.40 (s, 6H, CH_3, Mes), 2.62 (s, br, 12H, N-CH_3, NHC^{Me4}), 4.83 (s, 1H, C^4-*H*), 5.01 (dd, ^3J = 12 Hz, ^4J = 2 Hz, 1H, C^1-*H*), 6.82-6.86 (m, 6H, Ar-*H*), 6.91 (br, S, 2H, Ar-*H*), 6.95-6.99 (m, 4H, Ar-*H*), 7.29 (t, 2H, ^3J = 7.6 Hz, Ar-*H*), 7.34 (1H, dd, ^3J = 12 Hz, ^3J = 3.6 Hz, C*H* of C^2)

ppm. $^{13}C\{^1H\}$ NMR (100 MHz, C_6D_6, 298 K): δ = 8.5 (4C, *C*H_3-C-C-*C*H_3, NHC^{Me4}), 21.2 (1C, *C*H_3, Mes), 21.4 (1C, *C*H_3, Mes), 21.7 (1C, *C*H_3, Mes), 21.8 (1C, *C*H_3, Mes), 22.6 (1C, *C*H_3, Mes), 23.0 (1C, *C*H_3, Mes), 30.4 (*C*H_3 of C^7), 34.33 (4C, N-*C*H_3, NHC^{Me4}), 124.0 (4C, CH_3-*C*-*C*-CH_3, NHC^{Me4}), 126.9 (C*H*, C^2), 127.0 (*C*H C^1), 127.7 (1C, Ar-*C*H) 127.9 (1C, Ar-*C*H), 128.0 (2C, Ar-*C*H), 128.1 (2C, Ar-*C*H), 128.7 (3C, Ar-*C*H), 128.93 (1C, Ar-*C*H), 128.97 (2C, Ar-*C*H), 129.05 (2C, Ar-*C*H), 164.32 (*C*H, C^3). *Signals for C^4 and C^6 were not observed due to quadrupolar broadening by the Al nucleus.* 128.4 (Ar-C_q), 128.5 (Ar-C_q), 128.7 (Ar-C_q), 134.9 (Ar-C_q), 135.0 (Ar-C_q), 137.0 (Ar-C_q), 137.2 (Ar-C_q), 137.3 (Ar-C_q), 144.9 (Ar-C_q), 151.2 (Ar-C_q), 151.3 (Ar-C_q), 172.5 (*C*-CH_3, C^5) ppm. FT-IR (KBr, cm^{-1}): ῡ (cm^{-1}) = 1654 (mw), 1602 (mw), 1516 (s), 1446 (s),1368 (s), 1255 (m), 1195 (w), 1152 (w), 1082 (m), 1056 (s), 1030 (s), 883 (w), 840 (s), 797 (s),743(s), 683(s), 614 (w), 553 (s), 450 (vs). Elemental analysis: calcd. for ($C_{69}H_{82}Al_2N_4$): C, 81.14; H, 8.09; N, 5.49; found: C, 81.72; H, 8.10; N, 5.45.
HRMS LIFDI for $[C_{69}H_{82}Al_2N_4]^+$ = $[M]^+$: calcd. 1020.6170; found 1020.6165.

Synthesis of 8:

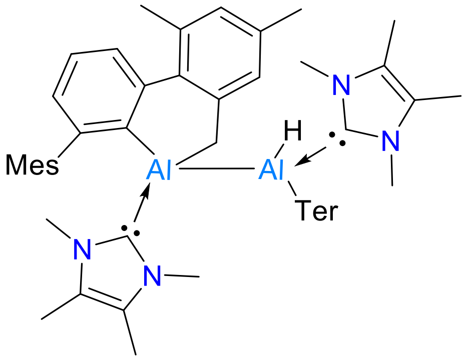

8

3 (0.03 g, 0.03 mmol) was added to a sealable NMR tube with 0.7 mL of benzene. The reaction mixture was then heated at 100 ºC for 24 h. The ^1H NMR spectrum of the mixture showed complete consumption of **3**. The solution was then filtered and left to crystallize by slow diffusion of pentane or hexanes. A small sample of colorless crystals of **4** emerged overnight. After separating these crystals of **4** the solution was allowed to evaporate slowly to obtain **8** as colorless crystals. Yield: 20.0 mg (70%) isolated. M.P.: >200 °C (decomposition); ^1H NMR (400 MHz, C_6D_6, 298 K): δ = 1.44 (s, 3H, C*H*$_3$-C-C-C*H*$_3$, NHC^{Me4}), 1.47 (s, 9H, C*H*$_3$-C-C-C*H*$_3$, NHC^{Me4}), 1.55 (s, 3H, C*H*$_3$, Mes), 1.68 (br, 2H, C*H*$_2$), 1.88 (3H, C*H*$_3$, Mes), 2.06 (s, 9H, C*H*$_3$, Mes), 2.15 (s, 3H, C*H*$_3$, Mes), 2.29 (s, 6H, C*H*$_3$, Mes), 2.52 (s, 9H, C*H*$_3$, Mes), 2.98 (s, 9H, N-

CH_3, NHC^{Me4}), 3.26 (s, 3H, N-CH_3, NHC^{Me4}), 4.41 (1H, Al-*H*), 6.22 (s, 2H, Ar-*H*), 6.40 (s, 1H, Ar-*H*), 6.64-6.67 (m, 2H, Ar-*H*), 6.90 (s, 2H, Ar-*H*), 6.92 (s, 2H, Ar-*H*), 7.04-7.11 (s, 2H, Ar-*H*), 7.15- 7.19 (m, 2H, Ar-*H*), 7.24 (t, ^3J = 7.5 Hz, 1H, Ar-*H*) ppm. $^{13}C\{^1H\}$ NMR (100 MHz, C_6D_6, 298 K): δ = 8.8 (4C, *C*H$_3$-C-C-*C*H$_3$, NHC^{Me4}), 21.3 (1C, *C*H$_3$, Mes) , 21.6 (2C, *C*H$_3$, Mes), 21.7 (2C, *C*H$_3$, Mes), 21.9 (1C, *C*H$_3$, Mes), 22.4 (1C, *C*H$_3$, Mes), 23.2 (1C, *C*H$_3$, Mes), 23.4 (*C*H$_2$), 34.5 (3C, *C*H$_3$, Mes), 34.8 (2C, N-*C*H$_3$, NHC^{Me4}), 36.7 (2C, N-*C*H$_3$, NHC^{Me4}), 124.1 (4C, CH$_3$-*C*-*C*-CH$_3$, NHC^{Me4}), 125.1 (1C, Ar-*C*H), 126.2 (1C, Ar-*C*H), 126.4 (1C, Ar-*C*H), 126.5 (1C, Ar-*C*H), 127.0 (1C, Ar-*C*H), 127.2 (1C, Ar-*C*H), 127.7 (2C, Ar-*C*H), 128.0 (1C, Ar-*C*H) 128.7 (2C, Ar-*C*H), 128.8 (1C, Ar-*C*H), 128.9 (1C, Ar-*C*H), 129.4 (1C, Ar-*C*H), 126.9 (Ar-C$_q$), 127.0 (Ar-C_q), 127.2 (Ar-C_q), 128.3 (Ar-C_q), 128.5 (Ar-C_q), 128.7 (Ar-C_q), 133.4 (Ar-C_q), 133.5 (Ar-C_q), 133.7 (Ar-C_q), 134.2 (Ar-C_q), 135.8 (Ar-C_q), 136.1 (Ar-C_q), 139.1 (Ar-C_q), 149.1(Ar-C_q), 149.6 (Ar-C_q) ppm. FT-IR (KBr, cm^{-1}): ῡ (cm^{-1}) = 1694 (m), 1663 (w), 1616 (m), 1578 (w), 1440 (s), 1378 (s), 1085 (w), 1054 (w), 1031 (s), 861 (m), 799 (s), 723 (s). Elemental analysis: calcd. for ($C_{62}H_{74}Al_2N_4 \cdot C_5H_{12}$): C, 80.36; H, 8.66; N, 5.59; found: C, 80.27; H, 8.62; N, 5.61.
HRMS LIFDI for $[C_{62}H_{74}Al_2N_4]^+ = [M]^+$: calcd. 928.5544; found 928.5512.

Reduction of 2 in dilute benzene solutions:

In a glove box, dry benzene (15 mL) was added to a vial containing **2** (0.24 g, 0.32 mmol, 0.02 molar solution) and freshly prepared potassium graphite (0.22 g, 1.60 mmol) at room temperature, and the mixture was stirred vigorously for 17 h at room temperature. The ^{1}H NMR spectrum of the resulting mixture showed consumption of the starting material **2** and the presence of signals for **3** (35%) and **4** (65%). The reaction mixture was repeatedly washed with hexanes (3×5 mL) and left to crystallize. After two days, compound **4** was isolated from this mixture (11 mg; 7%).

Note: Longer reaction times led to the formation of unidentified side products. The reaction in dilute toluene did not increase the formation of **5** *but instead increased the formation of unidentified side products*.

Reduction of 2 in dilute hexanes solutions:

In a glove box, dry hexanes (20 mL) was added to a vial containing **2** (0.05 g, 0.07 mmol) and freshly prepared potassium graphite (0.285 g, 2.1 mmol) at room temperature. Then reaction suspension was stirred vigorously for 20 days at room temperature. A ^{1}H NMR spectrum of the resulting mixture showed consumption of all starting materials and the presence of signals of **3**

along with some unidentified side products. The solution was then evaporated, and the solid was washed with 3 mL of cold hexanes to obtain a bright yellow solid (**3**). Yield: 24 mg (75%). In this case, we got very few crystals of **8** from the hexanes wash.

Note: The molarity of the reaction mixture could not be determined as compound ***2*** *is not fully soluble in hexanes.*

1.3. NMR spectra

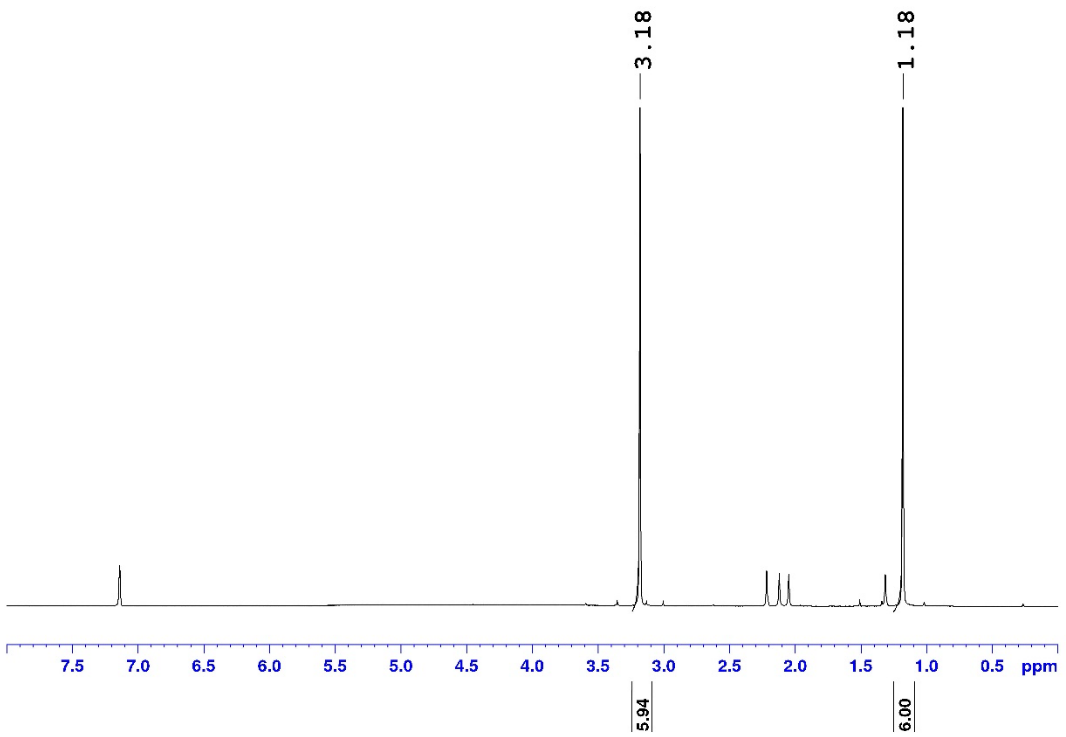

Figure S1. $^{1}H\{^{27}Al\}$ NMR spectrum of **NHC^{Me4}·AlH_3** in C_6D_6 at RT.

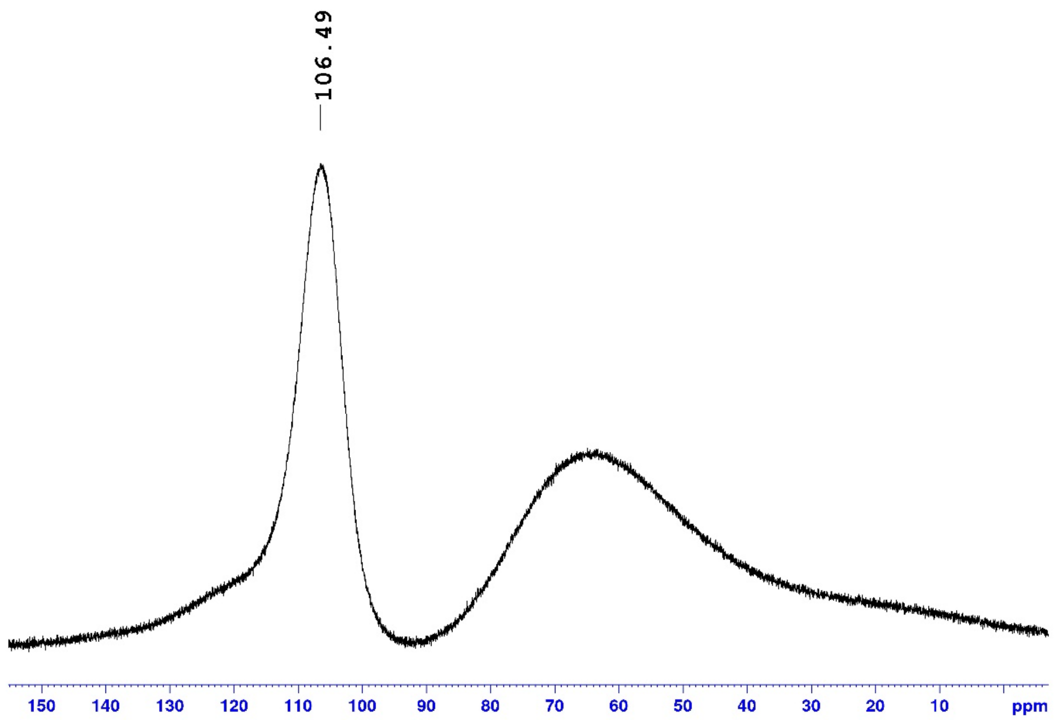

Figure S2. $^{27}Al\{^{1}H\}$ NMR spectrum of **NHC^{Me4}·AlH_3** in C_6D_6 at RT.

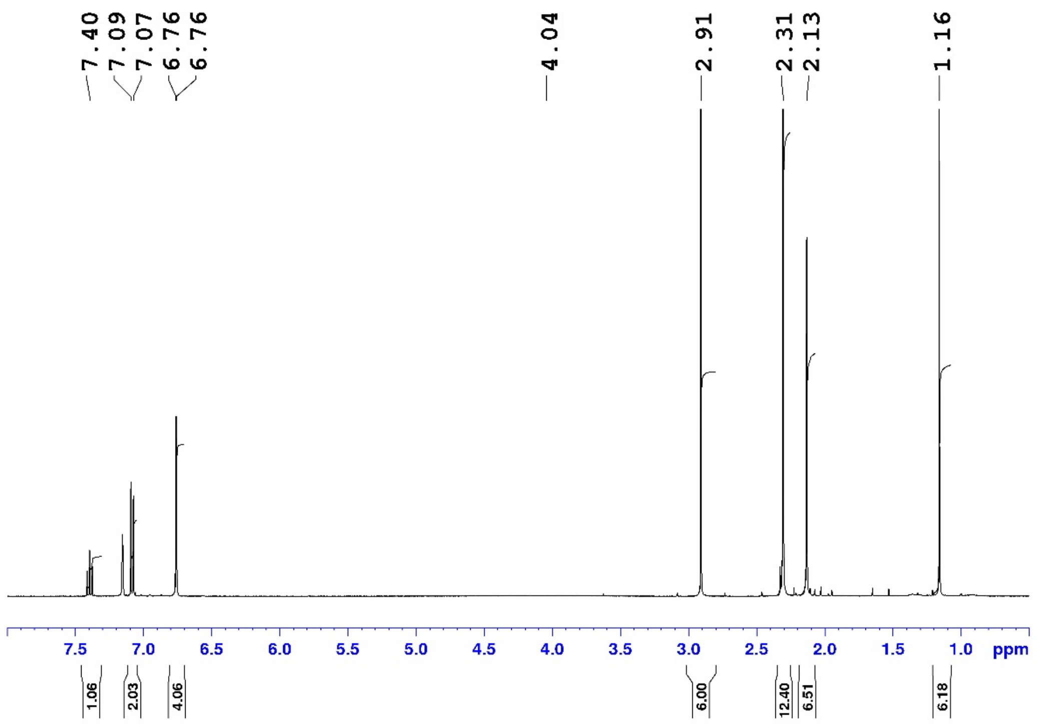

Figure S3.^{1}H NMR spectrum of **1** in C_6D_6 at RT.

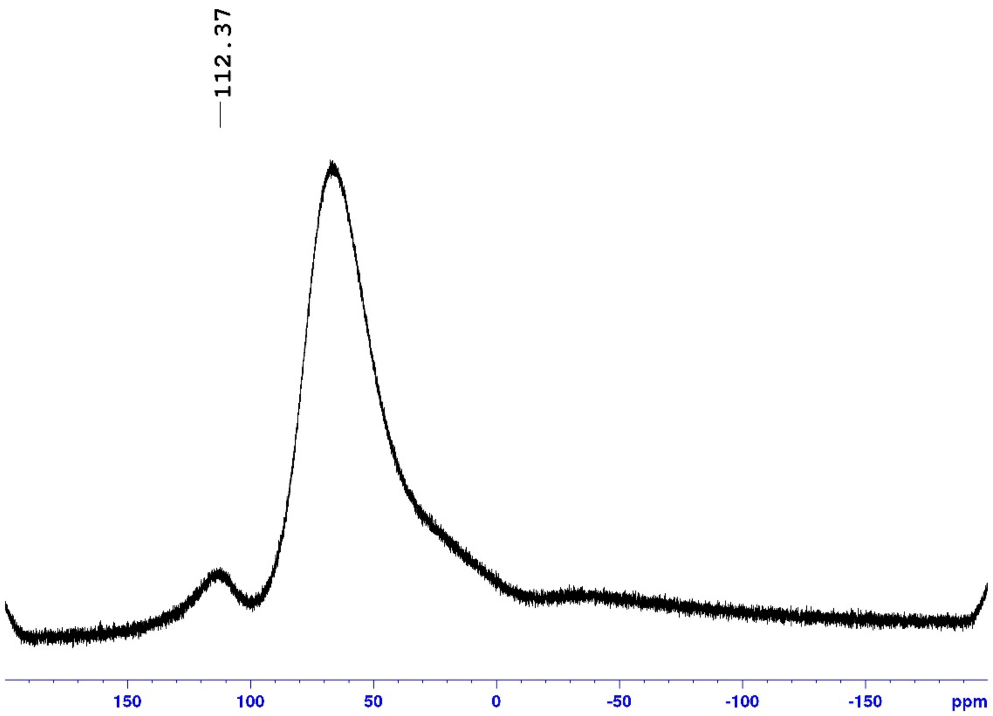

Figure S4. ^{27}Al{^{1}H} NMR spectrum of **1** in C_6D_6 at RT.

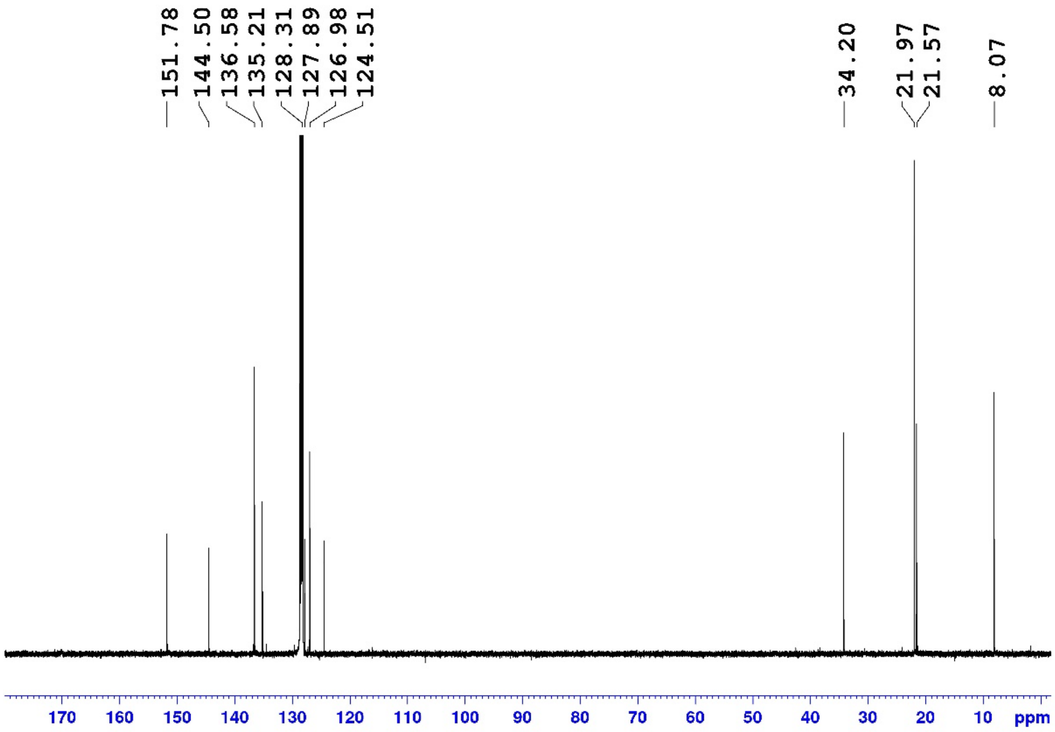

Figure S5. $^{13}C\{^{1}H\}$ NMR spectrum of **1** in in C_6D_6 at RT.

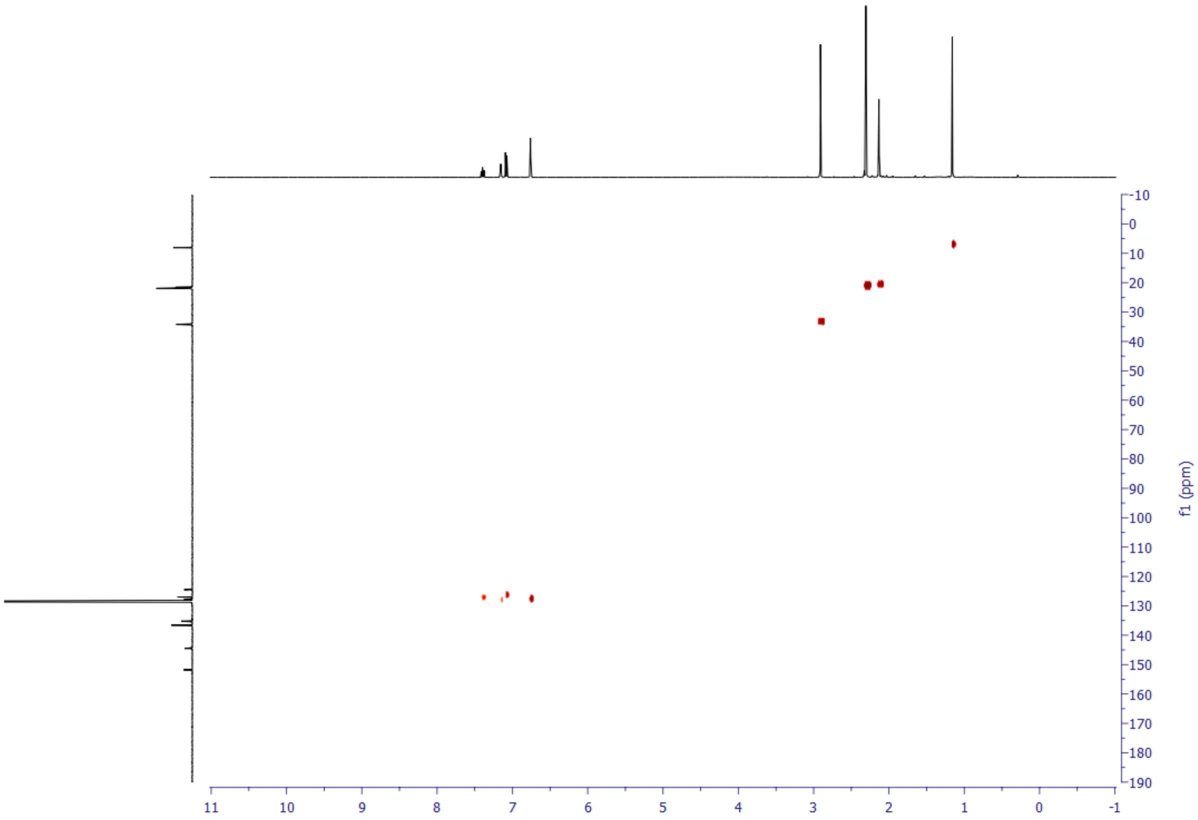

Figure S6. ^{13}C-^{1}H HSQC spectrum of **1** in C_6D_6 at RT.

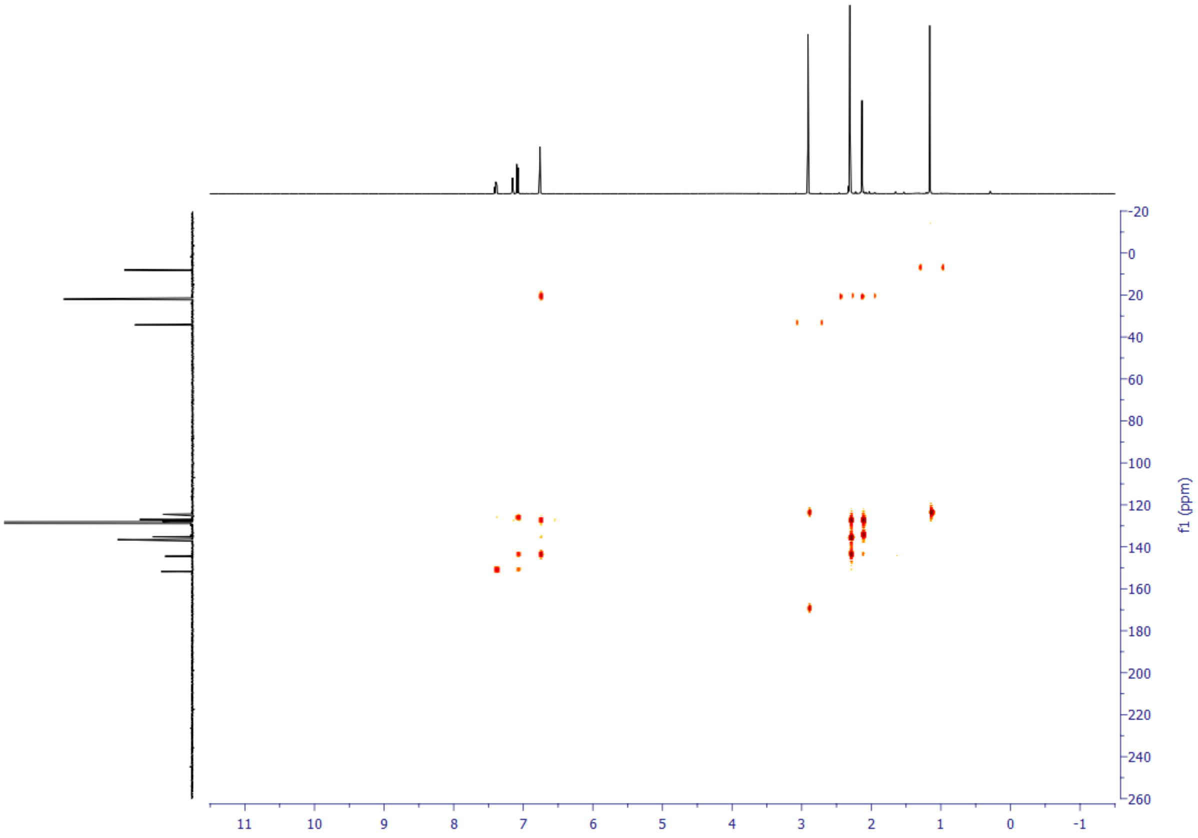

Figure S7. ^{13}C-^{1}H HMBC spectrum of **1** in C_6D_6 at RT.

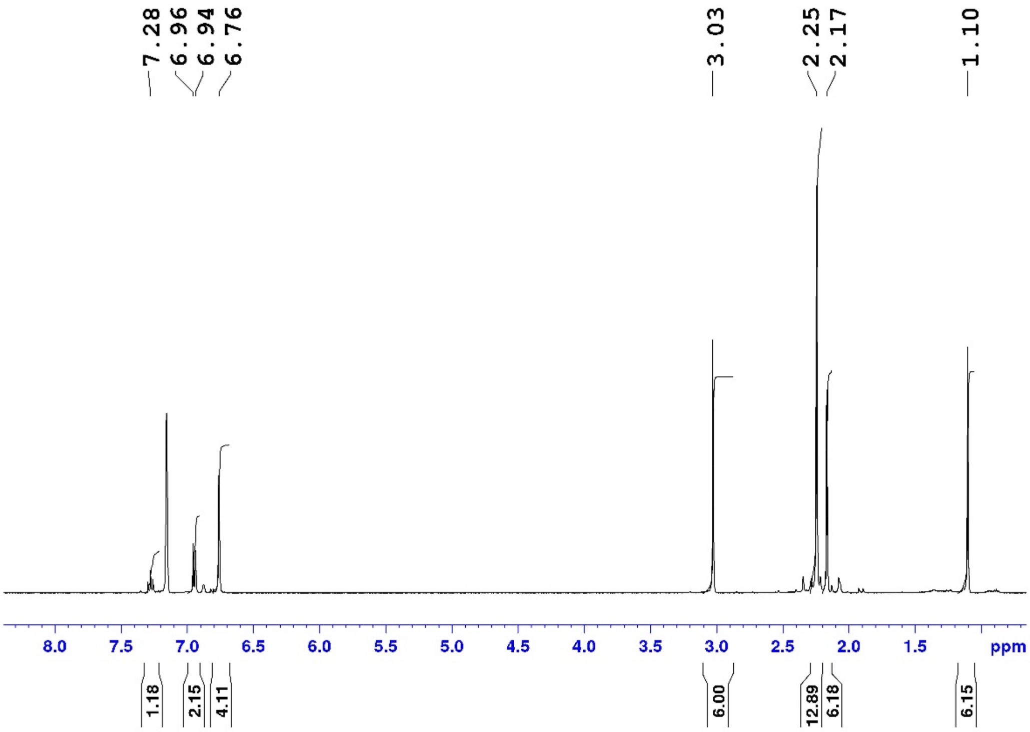

Figure S8. ^{1}H NMR spectrum of **2** in C_6D_6 at RT.

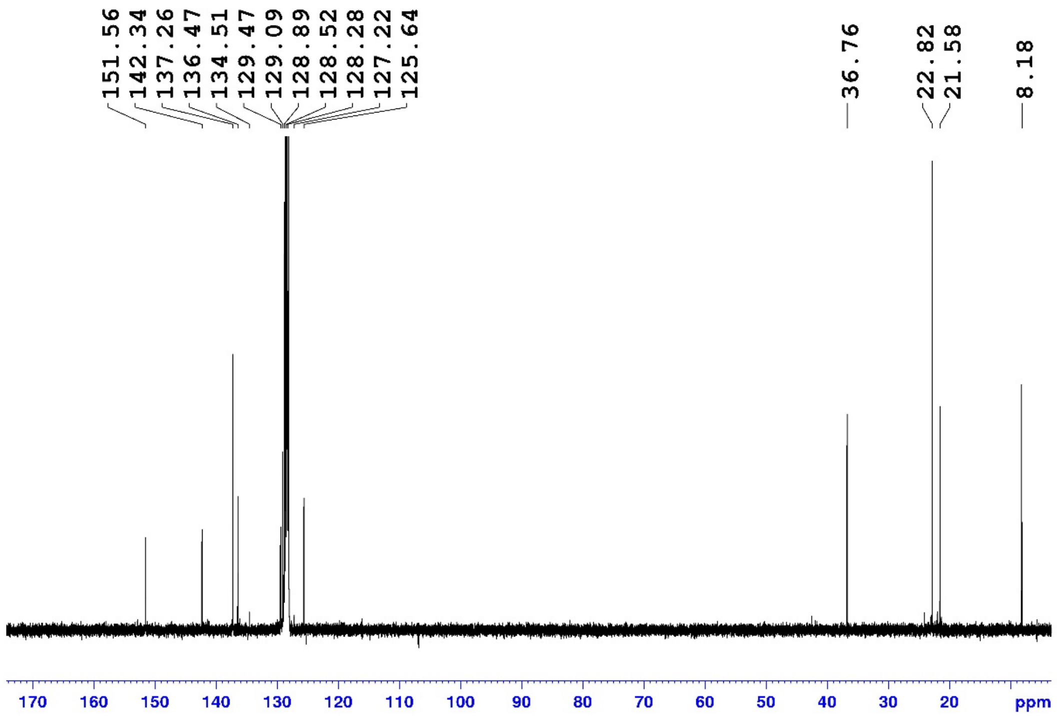

Figure S9. $^{13}C\{^{1}H\}$ NMR spectrum of **2** in C_6D_6 at RT.

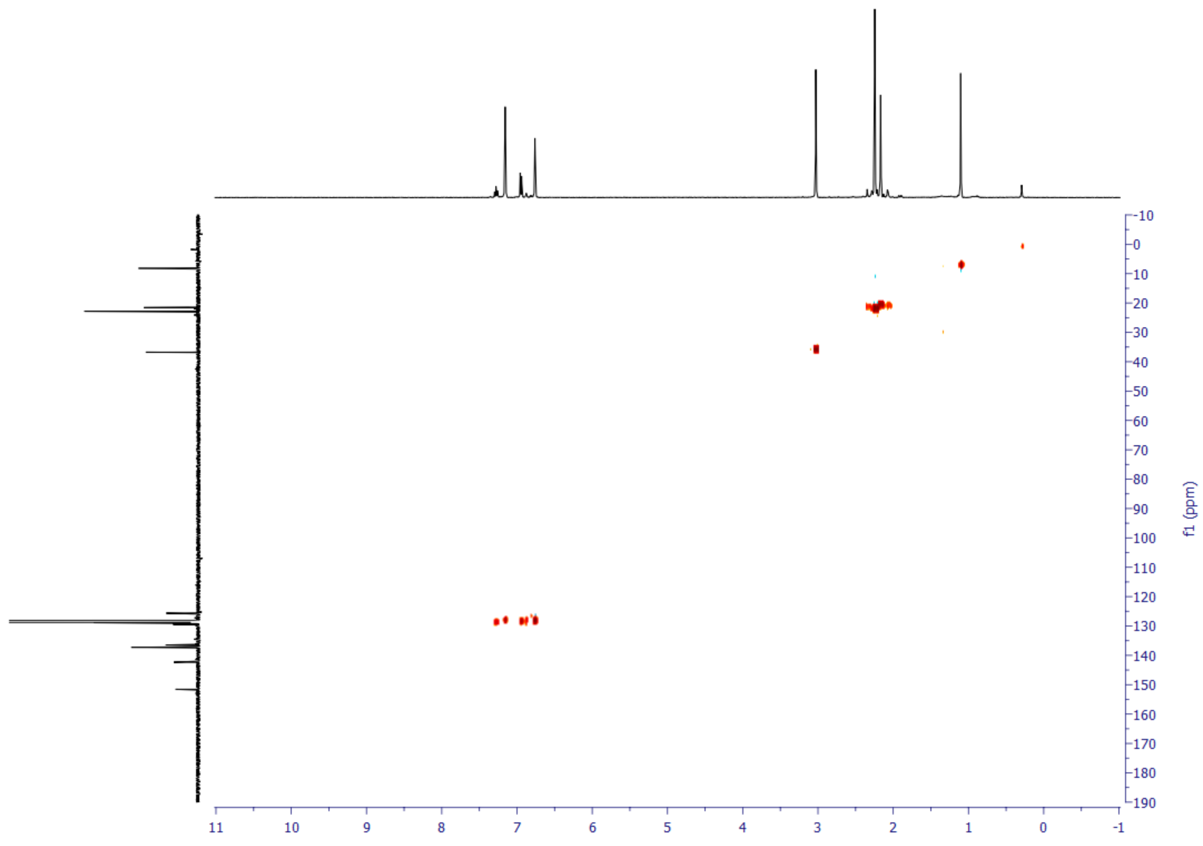

Figure S10. ^{13}C-^{1}H HSQC spectrum of **2** in C_6D_6 at RT.

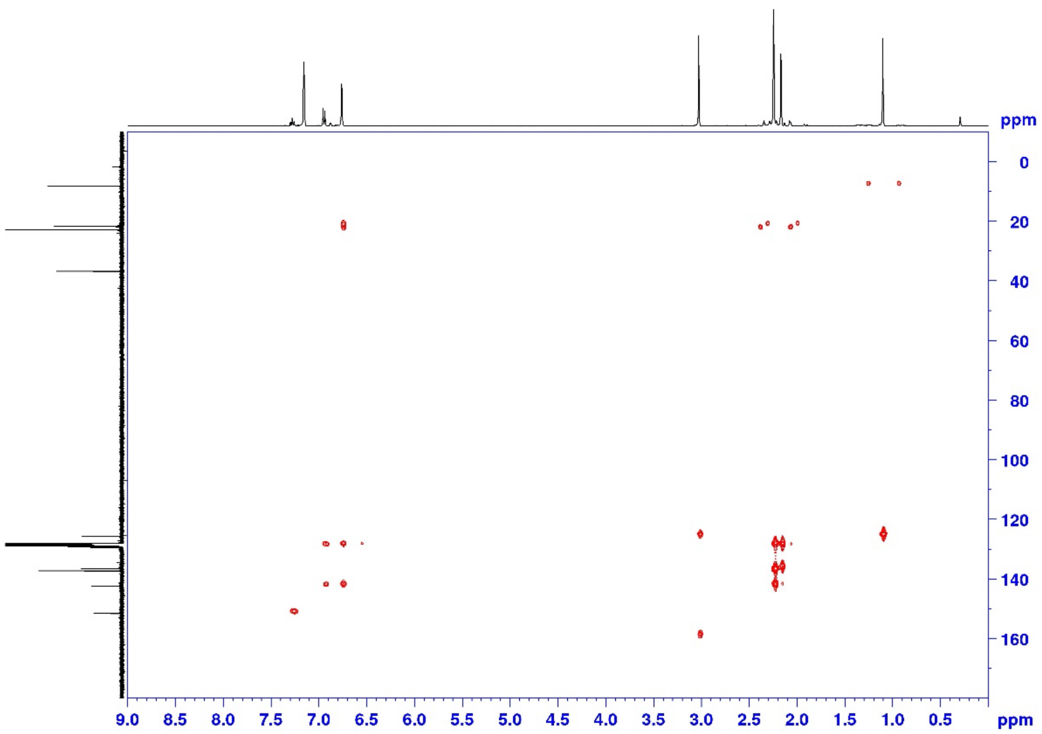

Figure S11. ^{13}C-^{1}H HMBC spectrum of **2** in C_6D_6 at RT

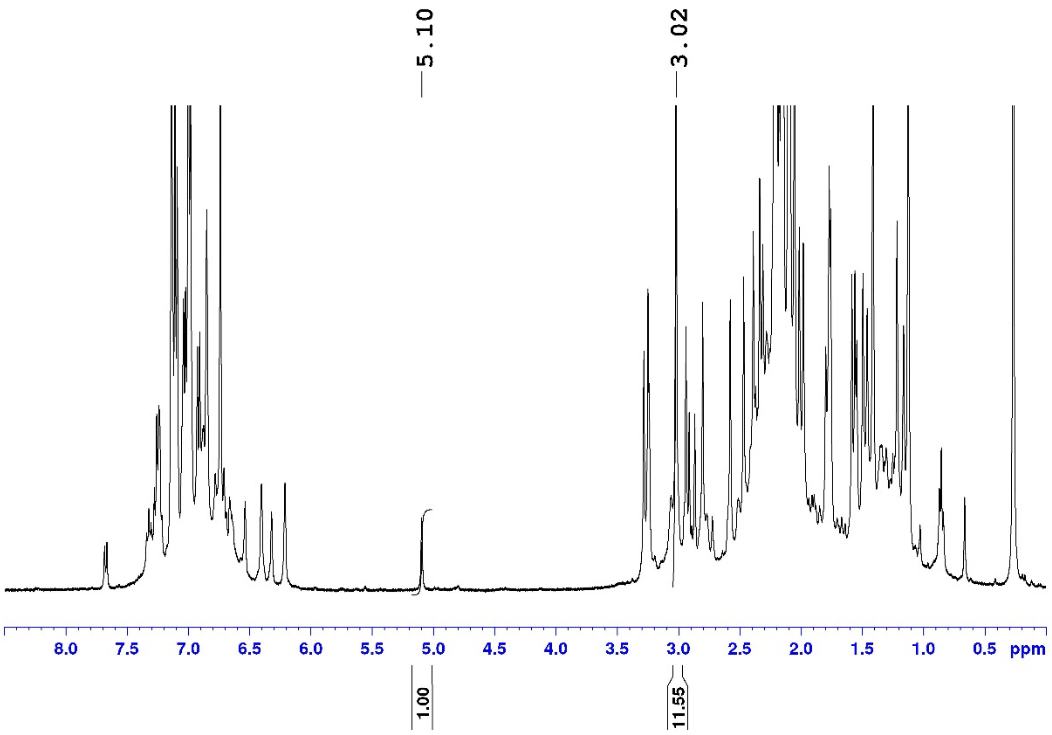

Figure S12. ^{1}H NMR spectrum of reduction of **2** with 1.5 equivalent of KC_8 (crude) in C_6D_6 at RT.

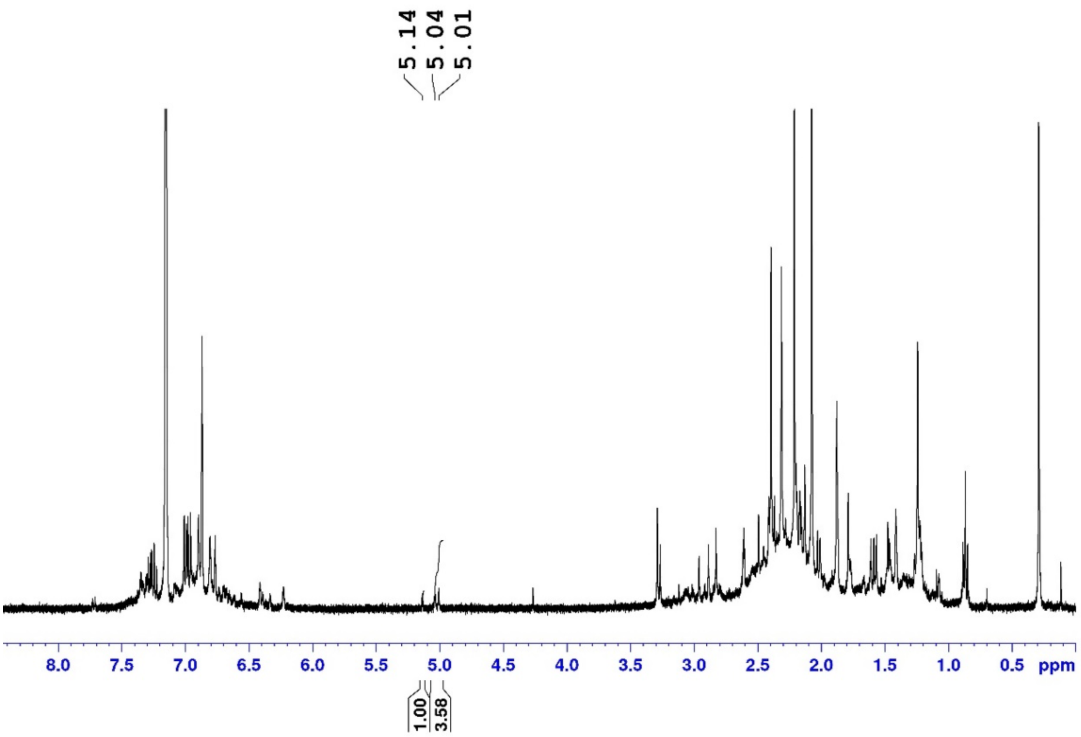

Figure S13. ^{1}H NMR spectrum of reduction of **2** in dilute solution in benzene (crude) in C_6D_6 at RT.

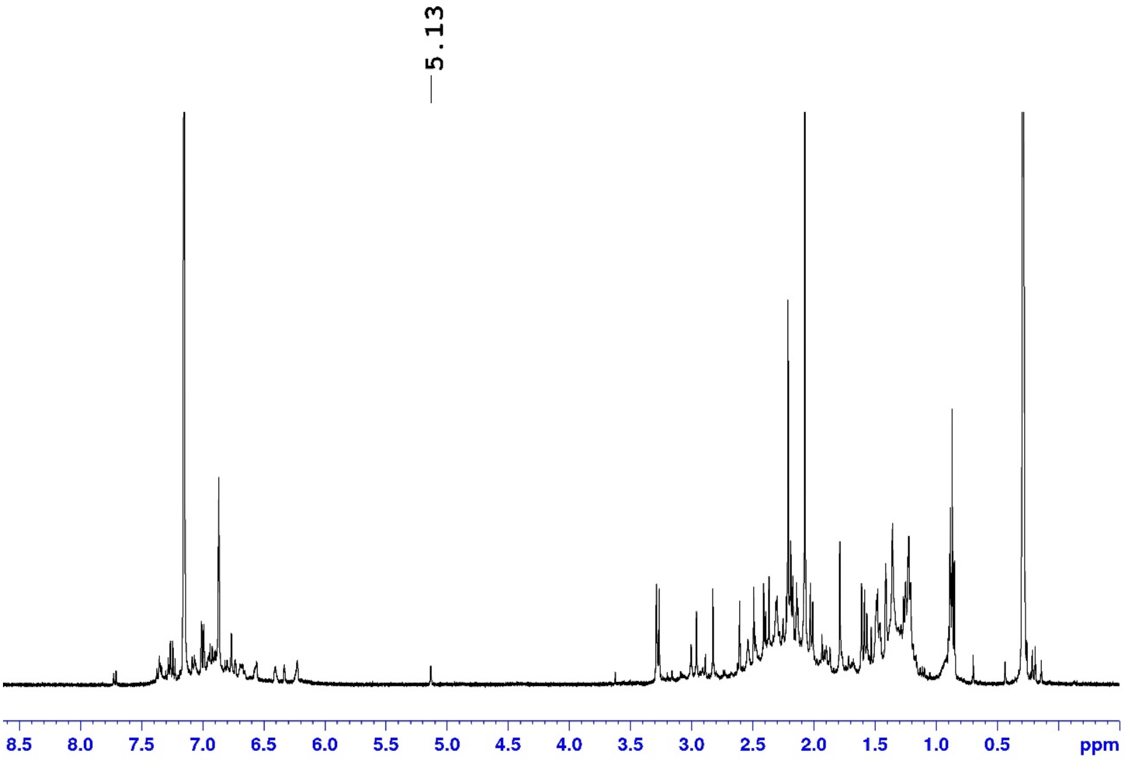

Figure S14. ^{1}H NMR spectrum of reduction of **2** in dilute solution in hexanes (crude) in C_6D_6 at RT.

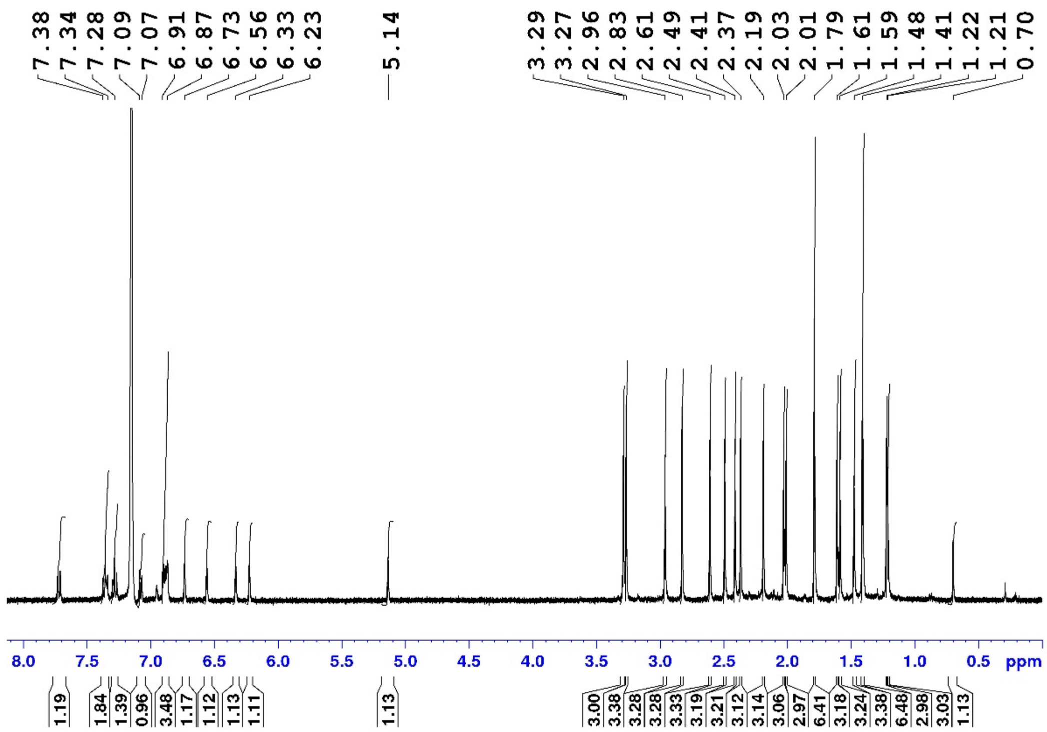

Figure S15.^{1}H NMR spectrum of **3** in C_6D_6 at RT.

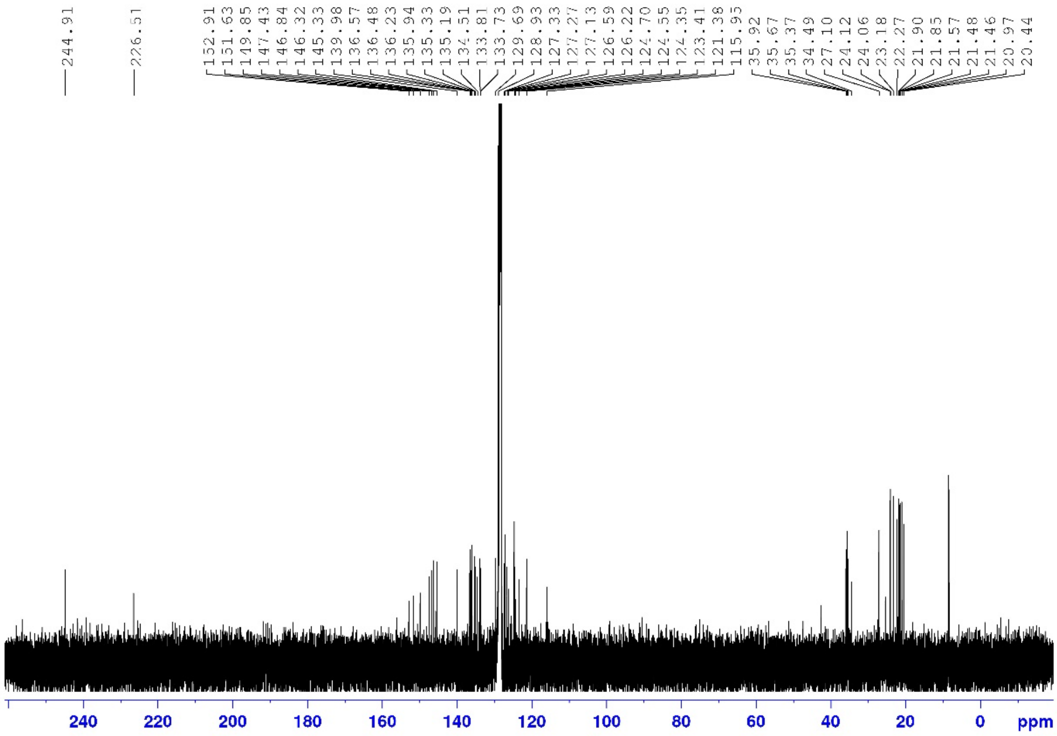

Figure S16. $^{13}C\{^1H\}$ NMR spectrum of **3** in C_6D_6 at RT.

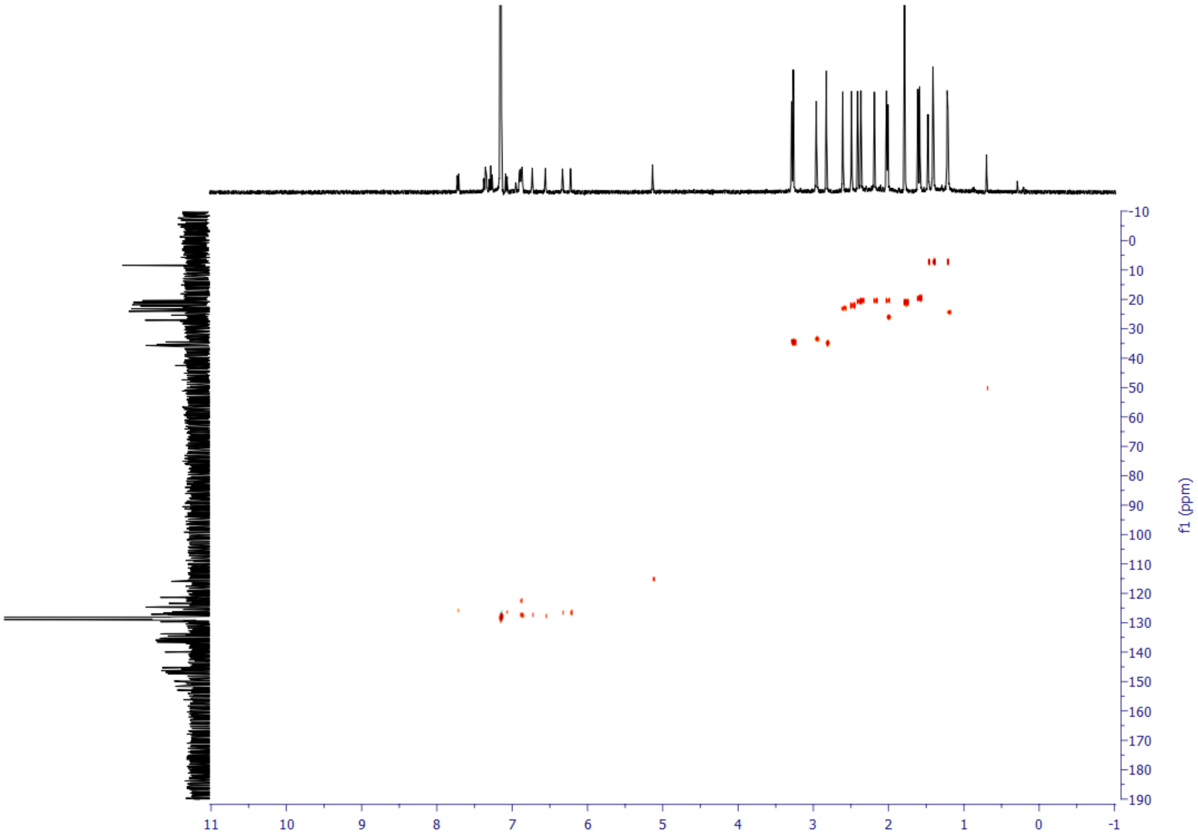

Figure S17. ^{13}C-^{1}H HSQC spectrum of **3** in C_6D_6 at RT.

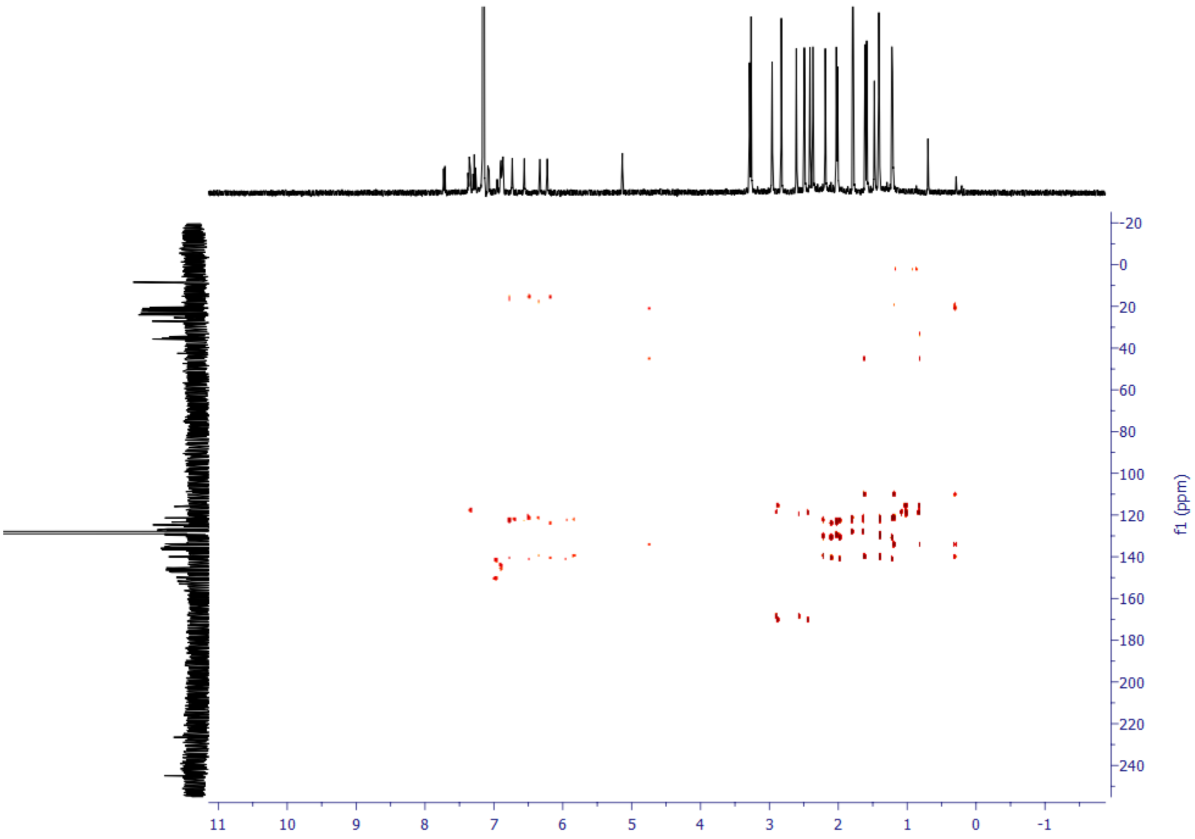

Figure S18. ^{13}C-^{1}H HMBC spectrum of **3** in C_6D_6 at RT

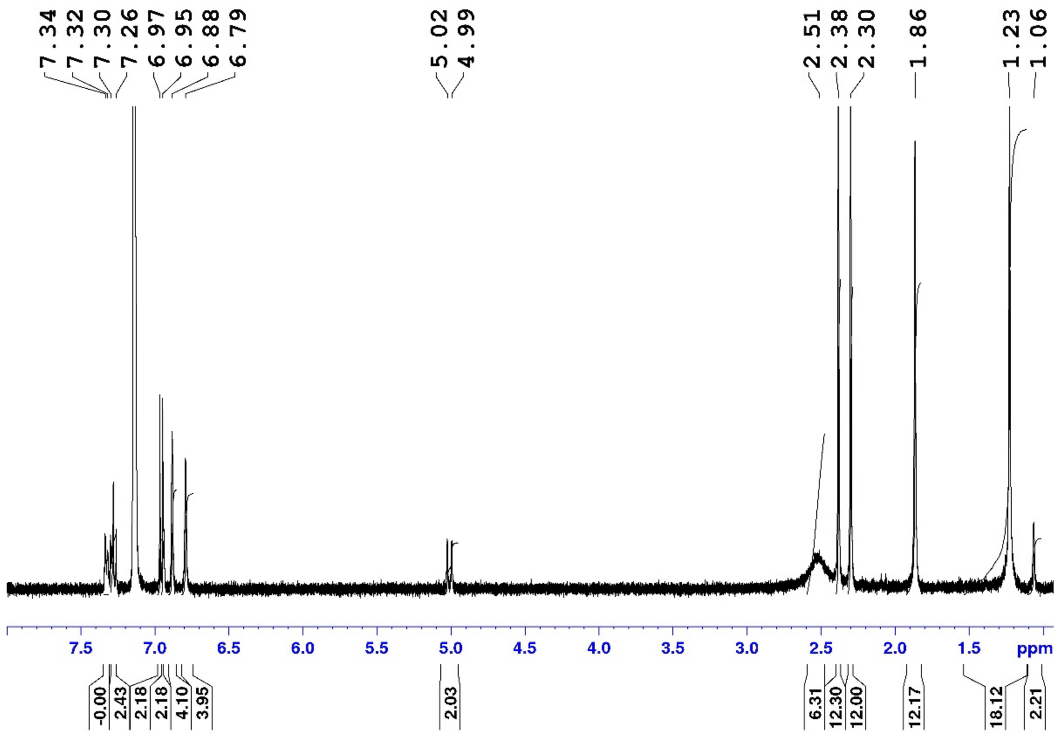

Figure S19.^{1}H NMR spectrum of **4** in C_6D_6 at RT.

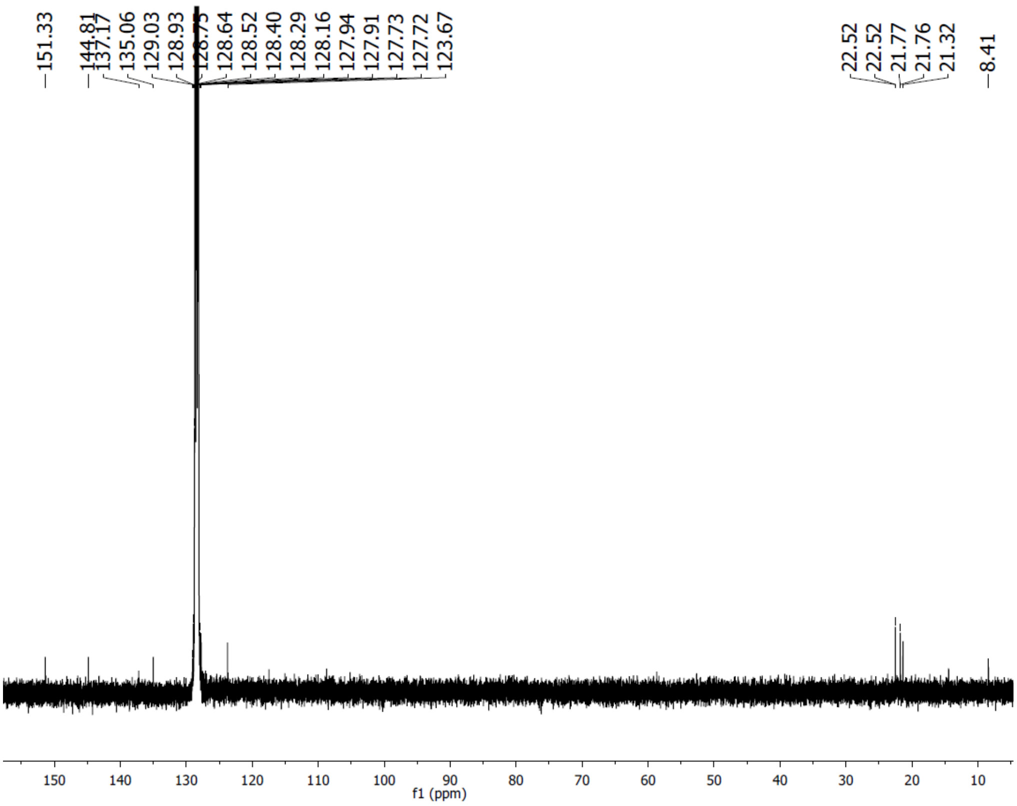

Figure S20. $^{13}C\{^1H\}$ NMR spectrum of **4** in C_6D_6 at RT.

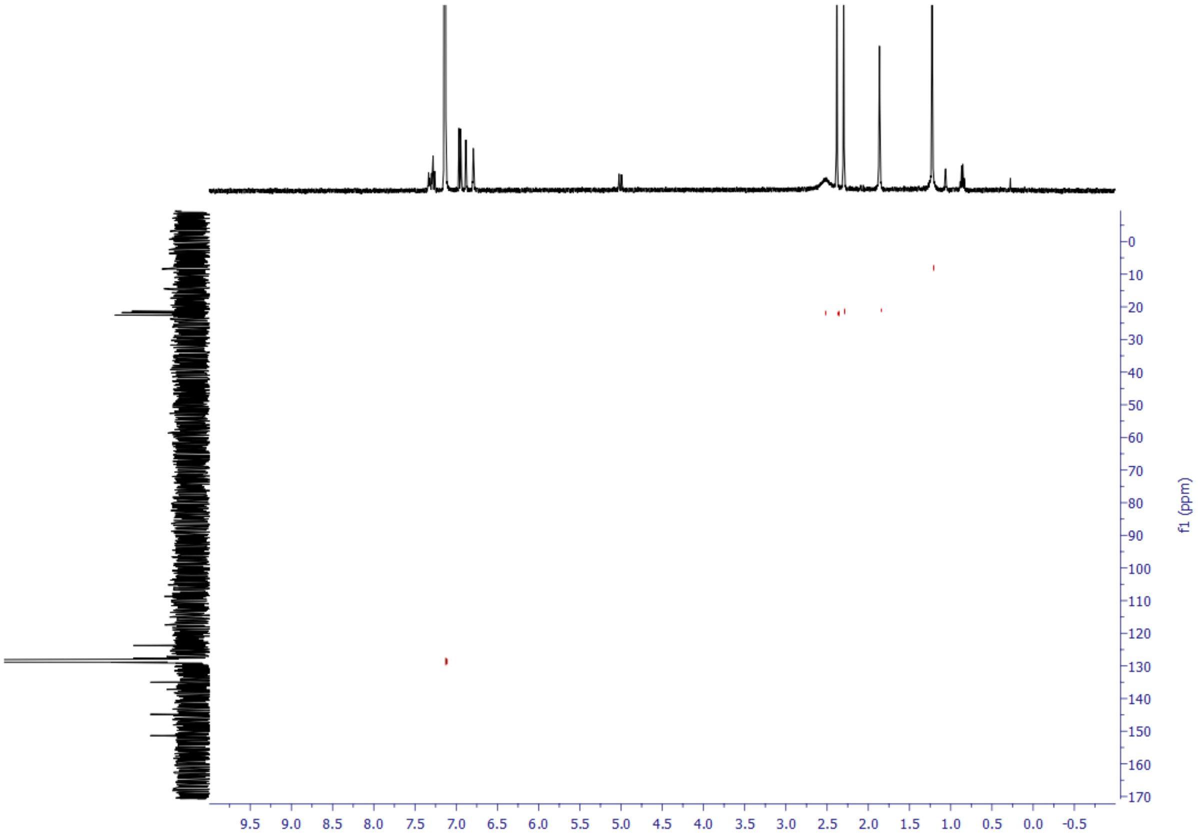

Figure S21. ^{13}C-^{1}H HSQC spectrum of **4** in C_6D_6 at RT

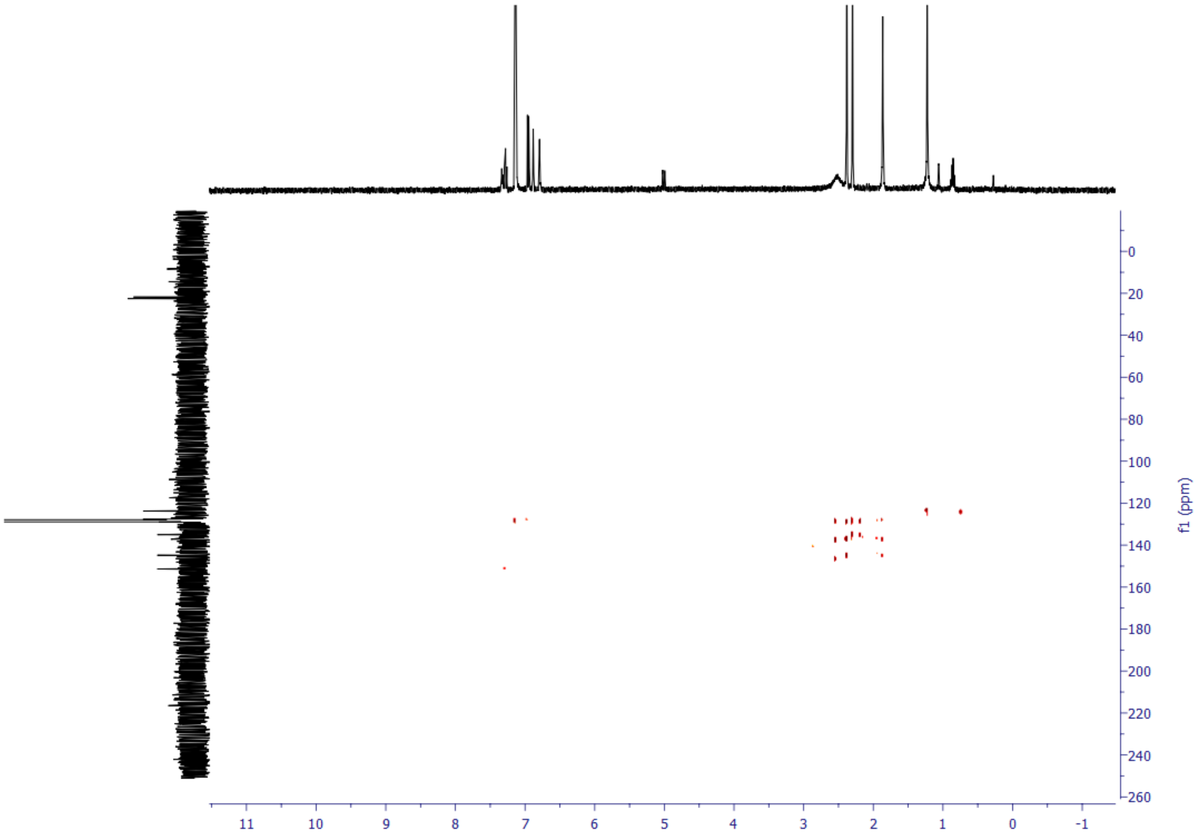

Figure S22. ^{13}C-^{1}H HMBC spectrum of **4** in C_6D_6 at RT.

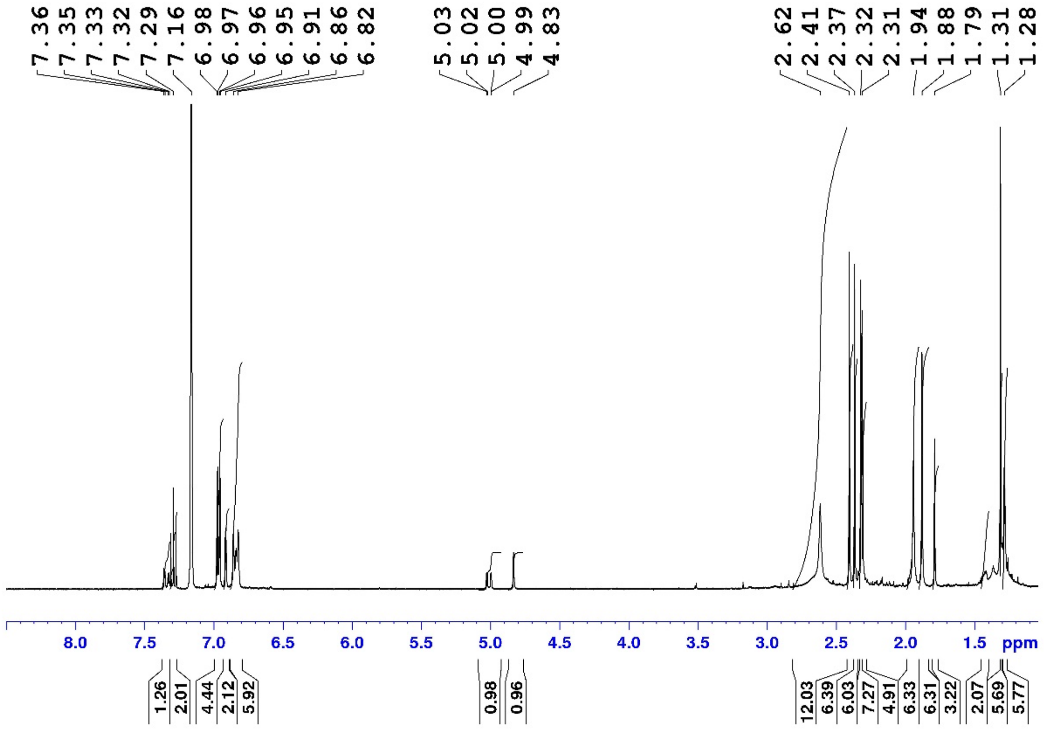

Figure S23.^{1}H NMR spectrum of **5** in C_6D_6 at RT.

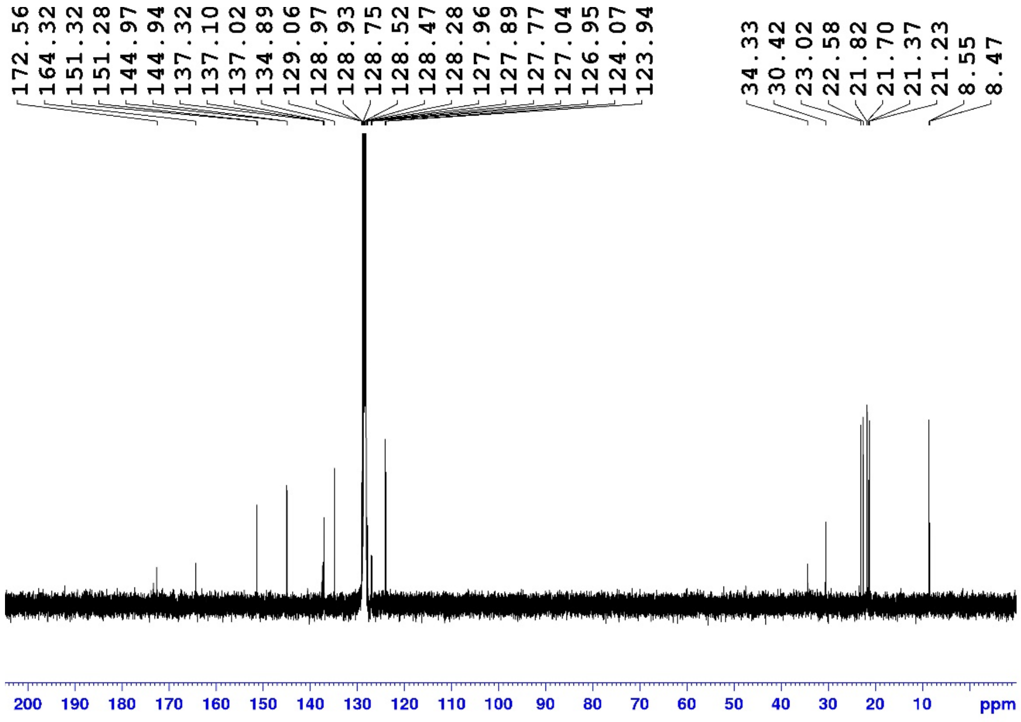

Figure S24. $^{13}C\{^{1}H\}$ NMR spectrum of **5** in C_6D_6 at RT.

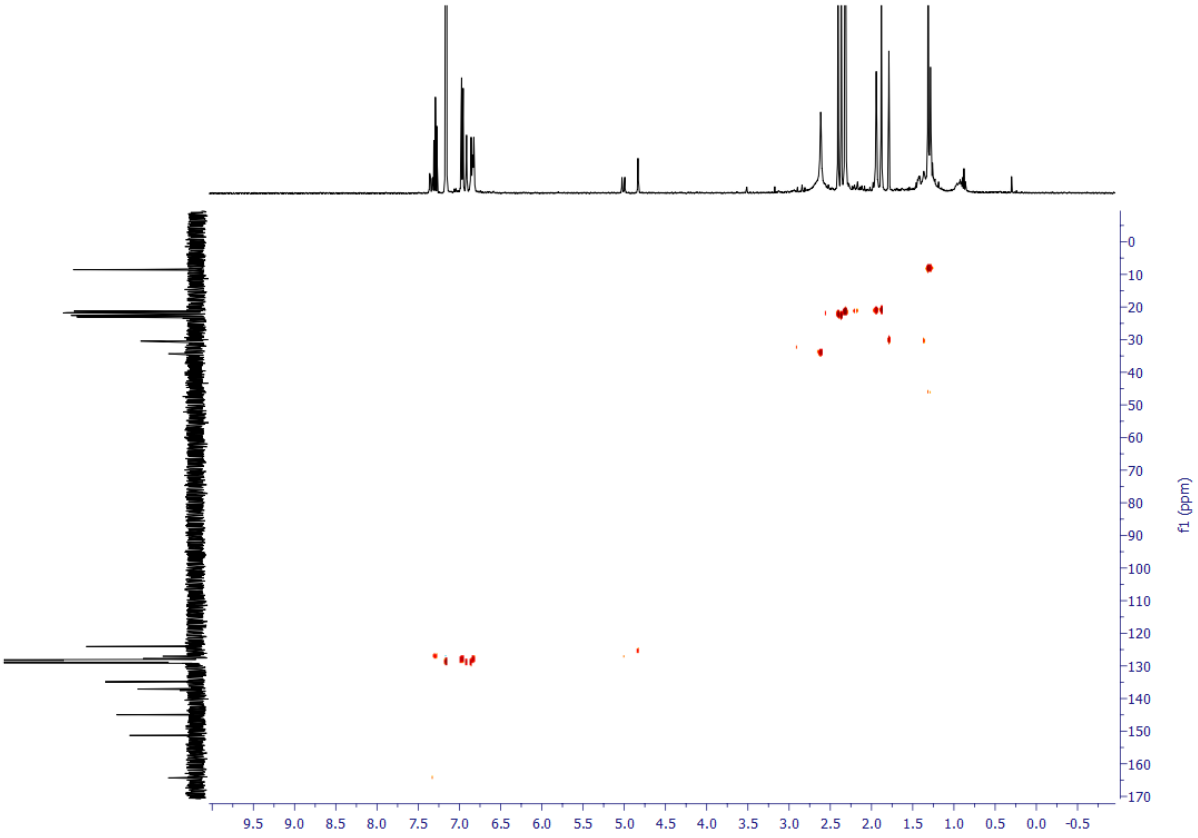

Figure S25. ^{13}C-^{1}H HSQC spectrum of **5** in C_6D_6 at RT.

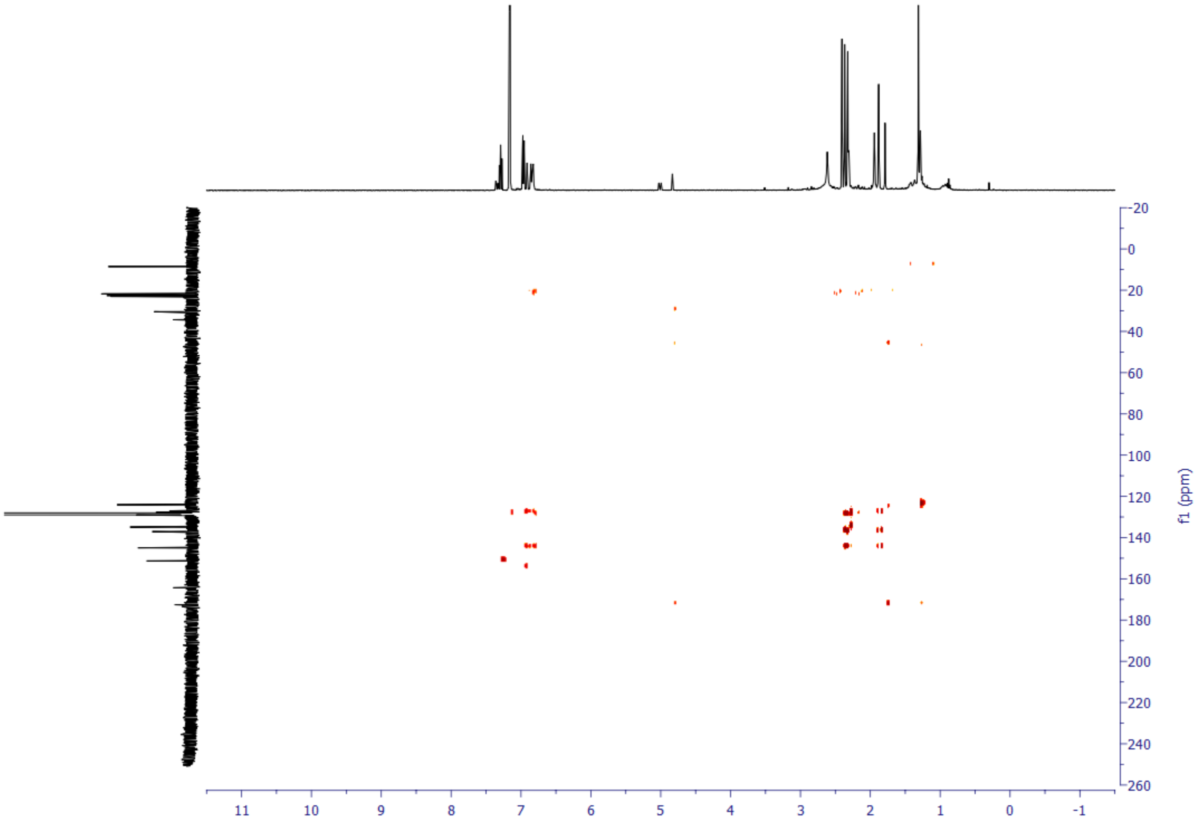

Figure S26. ^{13}C-^{1}H HMBC spectrum of **5** in C_6D_6 at RT

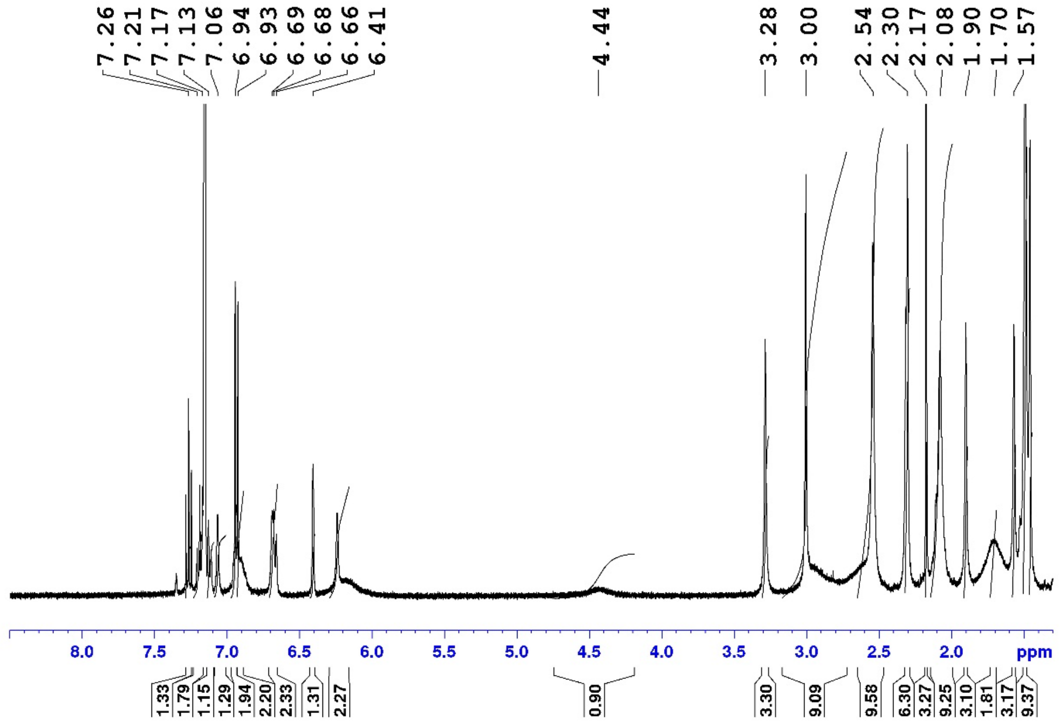

Figure 27. ^{1}H NMR spectrum of **8** in C_6D_6 at RT.

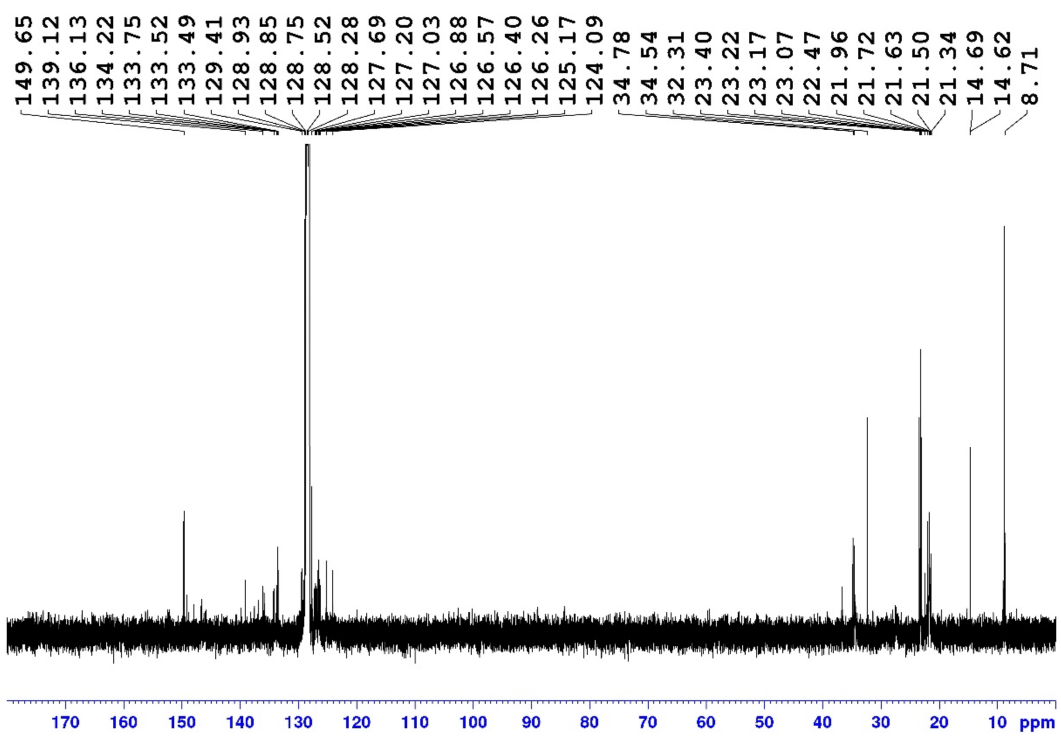

Figure S28. $^{13}C\{^1H\}$ NMR spectrum of **8** in C_6D_6 at RT.

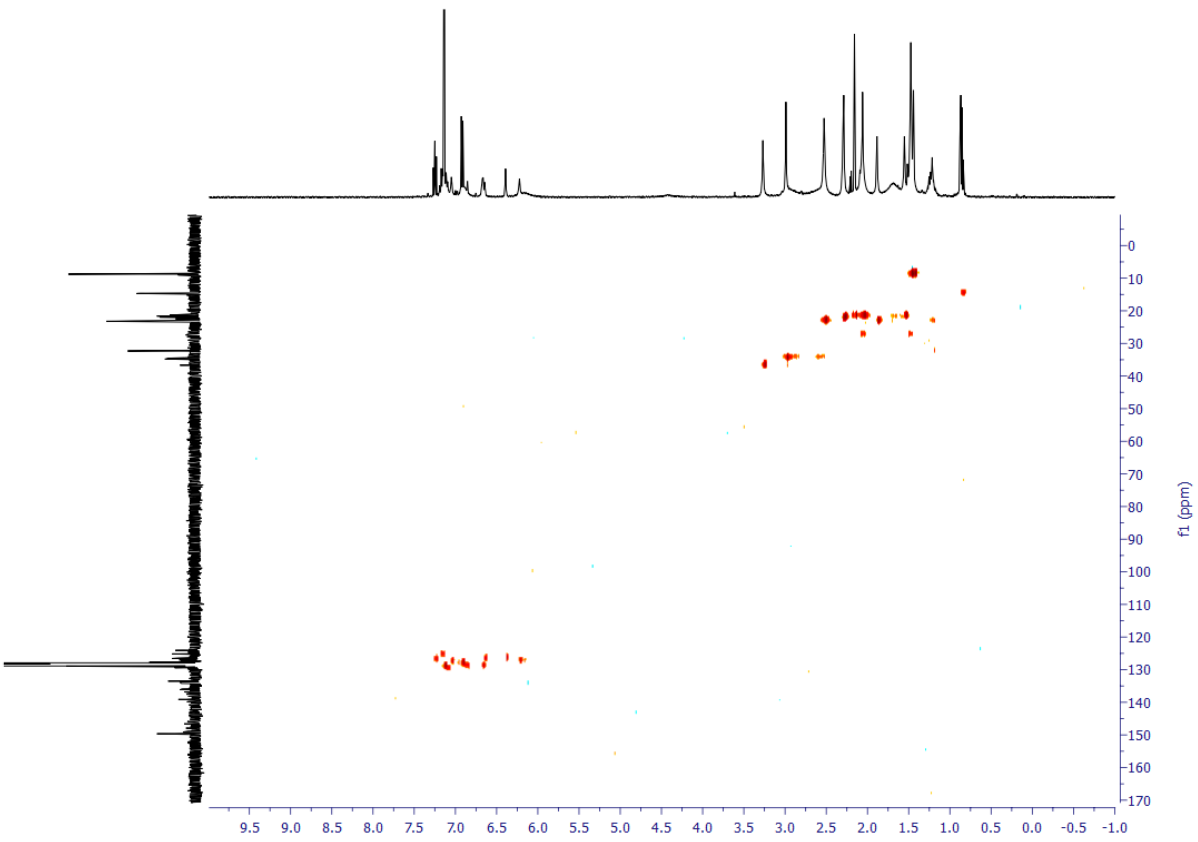

Figure S29. ^{13}C-^{1}H HSQC spectrum of **8** in C_6D_6 at RT.

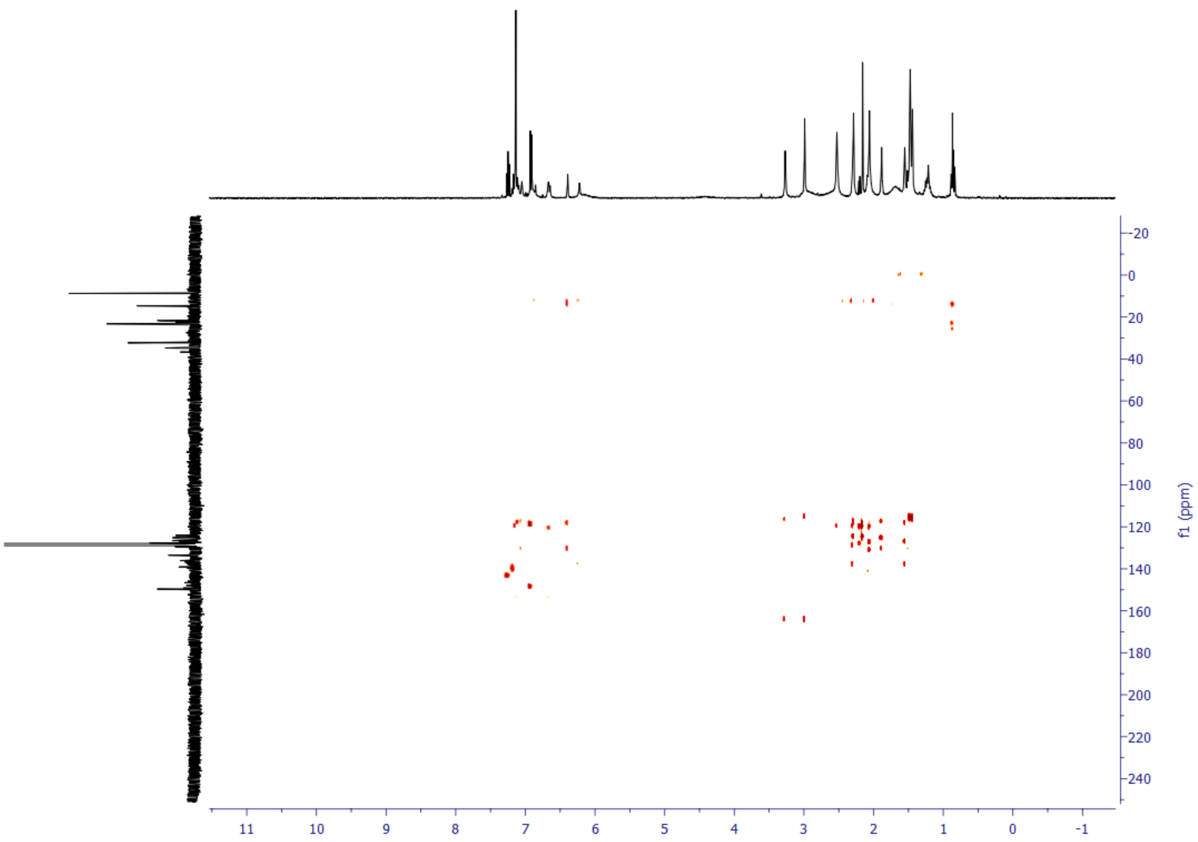

Figure S30. ^{13}C-^{1}H HMBC spectrum of **8** in C_6D_6 at RT.

1.4. UV-Vis spectra

The UV-vis absorption spectra of **3** was measured on a METTLER TOLEDO UV-vis Excellence UV5 spectrophotometer.

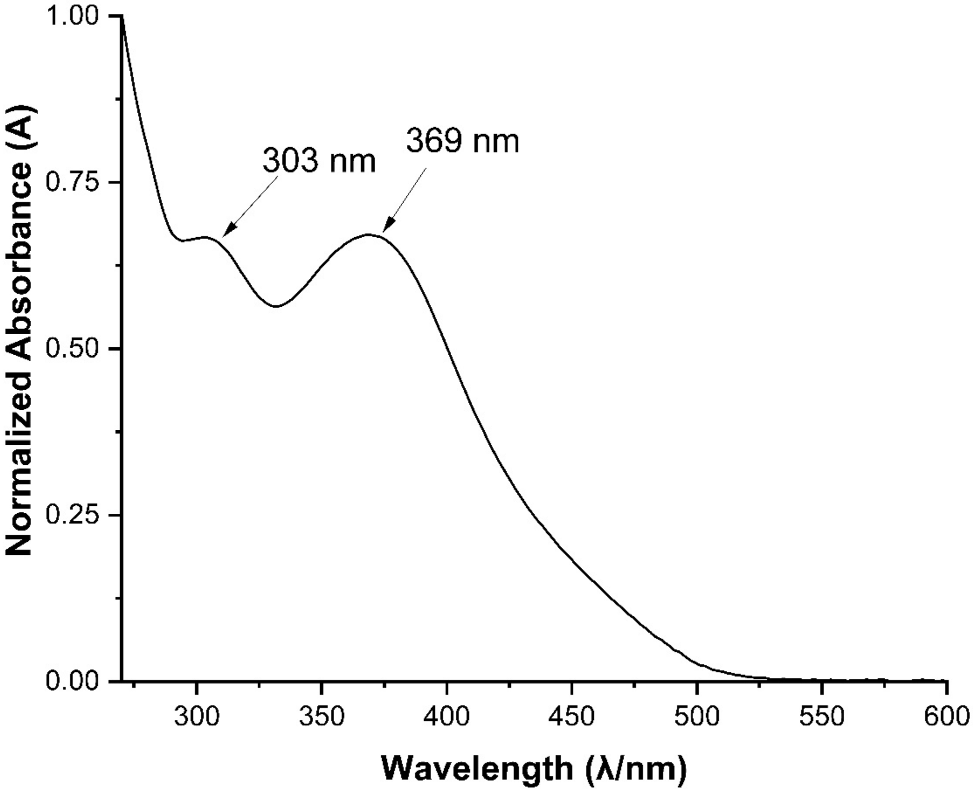

Figure S31. UV/Vis spectrum of compound **3** in hexanes at RT.

1.5. Crystal structure determination

The crystal data of **1** and **4-8** were collected on a XtaLAB Synergy, Dualflex, HyPix diffractometer with a hybrid pixel array detector and multi-layer mirror monochromated $Cu_{K\alpha}$ radiation. The crystal data of **2** were collected on a Bruker X8-APEX II diffractometer with a CCD area detector and multi-layer mirror monochromated $Mo_{K\alpha}$ radiation. The crystal data of **3** were collected on a Bruker D8 Quest diffractometer with a CMOS area detector and multi-layer mirror monochromated $Mo_{K\alpha}$ radiation. The structures were solved using the intrinsic phasing method,[5] refined with the ShelXL program[6] and expanded using Fourier techniques. All non-hydrogen atoms were refined anisotropically. Hydrogen atoms were included in structure factor calculations. All hydrogen atoms were assigned to idealized geometrical positions unless otherwise stated.

Crystal data for 1 (CCDC: 2107311**)**

Table

Data	**MaHa101_DeDh_a**
Empirical formula	$C_{31}H_{39}AlN_2$
Formula weight (g·mol^{-1})	466.62
Temperature (K)	100.0(6)
Radiation, λ (Å)	$Cu_{K\alpha}$ 1.54184
Crystal system	
Space group	$P2_1/n$
Unit cell dimensions	
a (Å)	8.67310(10)
b (Å)	13.6845(2)
c (Å)	22.6164(3)
α (°)	90
β (°)	95.0260(10)
γ (°)	90
Volume (Å^3)	2673.95(6)
Z	4
Calculated density (Mg·m^{-3})	1.159
Absorbtion coefficient (mm^{-1})	0.805
F(000)	1008
Theta range for collection	3.779 to 77.488°
Reflections collected	19589
Independent reflections	5475
Minimum/maximum transmission	0.66191/1.00000
Refinement method	Full-matrix least-squares on F^2
Data / parameters / restraints	5475 / 411 / 437
Goodness-of-fit on F^2	1.049
Final R indices [I>2σ(I)]	R_1 = 0.0366, wR^2 = 0.0979
R indices (all data)	R_1 = 0.0407, wR^2 = 0.1011
Maximum/minimum residual electron density (e·Å^{-3})	0.265 / –0.251

Refinement details: All hydrogen atoms except H1 and H2 were assigned to idealized positions. The coordinates of H1 and H2 of residue 1 (MAIN) were refined freely. The atomic displacement parameters for atoms N1 to C9 of the residues 2 and 12 (IMEM) were restrained with the RIGU keyword in the ShelXL input ('enhanced rigid bond' restraint for all bonds in the connectivity list. Standard values of 0.004 for both parameters s1 and s2 were used). The displacement parameters of atoms N1 to C9 of the residues 2 and 12 (IMEM) were restrained to the same value with similarity restraint SIMU. During refinement, the distances between

atoms Al1 of residue 1 (MAIN) and C2 of residues 2 and 12 (IMEM) were restrained to the same value using SADI. The 1-2 and 1-3 distances in N1 to C9 of residues 2 and 12 (IMEM) were restrained to the same values with the SAME keyword.

Crystal data for 2.C_7H_8 (CCDC: 2107312)

Table

Identification code	test1_a
Empirical formula	$C_{159}H_{188}Al_4I_8N_8$
Formula weight	3334.28
Temperature/K	100(2)
Crystal system	triclinic
Space group	P-1
a/Å	15.879(10)
b/Å	16.076(18)
c/Å	16.533(11)
α/°	92.956(13)
β/°	109.505(18)
γ/°	102.27(2)
Volume/Å^3	3853(6)
Z	1
ρ_{calc}g/cm^3	1.437
μ/mm^{-1}	1.683
F(000)	1674.0
Crystal size/mm^3	0.86 × 0.557 × 0.206
Radiation	MoK$_\alpha$ (λ = 0.71073)
2Θ range for data collection/°	3.336 to 52.986
Index ranges	-19 ≤ h ≤ 19, -20 ≤ k ≤ 20, -20 ≤ l ≤ 20
Reflections collected	58874
Independent reflections	15886 [R_{int} = 0.0591, R_{sigma} = 0.0512]
Data/restraints/parameters	15886/251/816
Goodness-of-fit on F^2	1.174
Final R indexes [I>=2σ (I)]	R_1 = 0.0552, wR_2 = 0.1173
Final R indexes [all data]	R_1 = 0.0806, wR_2 = 0.1432
Largest diff. peak/hole / e Å^{-3}	2.33/-1.35

Refinement details: The structure has disordered toluene solvent molecules. Therefore, necessary restraints and constraints were applied to all these disordered groups. More specifically, the similarity restraint SIMU and the rigid bond restraint RIGU have been applied, assuming that the atoms in these moieties move similarly in direction and magnitude. The same distances restraint SAME ensured similar geometries for all disordered residues of the same type. There are two disagreeable reflections [-1 0 1, -1 0 1], so OMIT instruction has been applied to remove them. OMIT has also been applied to ignore the reflection beyond 53°.

Crystal data for $3\cdot 2C_6H_6$ (CCDC 2107317)

Table

Empirical formula	$C_{71}H_{83}Al_2N_4$
Formula weight	1046.37
Temperature/K	100(2)
Crystal system	triclinic
Space group	P-1
a/Å	13.438(2)
b/Å	15.123(3)
c/Å	15.478(2)
α/°	75.326(6)
β/°	80.106(11)
γ/°	84.764(7)
Volume/Å^3	2994.0(9)
Z	2
ρ_{calc}g/cm^3	1.161
μ/mm^{-1}	0.094
F(000)	1126.0
Crystal size/mm^3	0.649 × 0.464 × 0.408
Radiation	MoKα (λ = 0.71073)
2Θ range for data collection/°	4.05 to 61.148
Index ranges	$-19 \le h \le 19$, $-21 \le k \le 21$, $-21 \le l \le 22$
Reflections collected	115032
Independent reflections	18206 [R_{int} = 0.0282, R_{sigma} = 0.0222]
Data/restraints/parameters	18206/0/714
Goodness-of-fit on F^2	1.030
Final R indexes [I>=2σ (I)]	R_1 = 0.0399, wR_2 = 0.1120
Final R indexes [all data]	R_1 = 0.0423, wR_2 = 0.1143
Largest diff. peak/hole / e Å^{-3}	0.49/-0.32

Refinement details: There are some disagreeable reflections [0 -4 3, 6 3 9, 1 1 1, 0 -1 1, -1 1 3, -1 1 0, -1 -1 3, -1 1 2] which have been omitted.

Crystal data for 4 (CCDC: 2107329)

Table

Identification code	MaHa090_DeDh_a
Empirical formula	$C_{68}H_{80}N_4Al_2$
Formula weight	1007.32
Temperature/K	100.15
Crystal system	monoclinic
Space group	I2/a
a/Å	16.9198(2)
b/Å	22.2833(2)
c/Å	17.4576(3)
α/°	90.0
β/°	117.934(2)
γ/°	90.0
Volume/Å^3	5815.16(14)
Z	4
ρ_{calc}g/cm^3	1.151
μ/mm^{-1}	0.776
F(000)	2168.0
Crystal size/mm^3	0.12 × 0.06 × 0.03
Radiation	Cu Kα (λ = 1.54184)
2Θ range for data collection/°	6.0042 to 155.1518
Index ranges	-21 ≤ h ≤ 15, -28 ≤ k ≤ 27, -21 ≤ l ≤ 22
Reflections collected	33342
Independent reflections	6108 [R_{int} = 0.0281, R_{sigma} = 0.0184]
Data/restraints/parameters	6108/0/344
Goodness-of-fit on F^2	1.024
Final R indexes [I>=2σ (I)]	R_1 = 0.0384, wR_2 = 0.0984
Final R indexes [all data]	R_1 = 0.0434, wR_2 = 0.1017
Largest diff. peak/hole / e Å^{-3}	0.29/-0.24.

Crystal data for $5 \cdot 2C_6H_6$ (CCDC: 2107337)

Table

Identification code	MaHa094_DeDh_a
Empirical formula	$C_{78}H_{91}N_4Al_2$
Formula weight	1138.50
Temperature/K	100.15
Crystal system	monoclinic
Space group	$P2_1/c$
a/Å	15.45280(10)
b/Å	15.23290(10)
c/Å	28.0296(3)
α/°	90
β/°	96.7420(10)
γ/°	90
Volume/Å^3	6552.29(9)
Z	4
ρ_{calc}g/cm^3	1.154
μ/mm^{-1}	0.745
F(000)	2452.0
Crystal size/mm^3	0.22 × 0.15 × 0.14
Radiation	CuKα (λ = 1.54184)
2Θ range for data collection/°	5.76 to 154.556
Index ranges	$-19 \le h \le 19$, $-17 \le k \le 19$, $-26 \le l \le 35$
Reflections collected	49192
Independent reflections	13342 [R_{int} = 0.0406, R_{sigma} = 0.0350]
Data/restraints/parameters	13342/384/833
Goodness-of-fit on F^2	1.033
Final R indexes [I>=2σ (I)]	R_1 = 0.0462, wR_2 = 0.1164
Final R indexes [all data]	R_1 = 0.0526, wR_2 = 0.1207
Largest diff. peak/hole / e Å^{-3}	0.32/-0.31

Refinement details: The displacement parameters of atoms Al1 and Al2 of the residues 1 and 21 (MAIN) were constrained to the same value with the EADP keyword. The coordinates of atoms Al1 and Al2 of the residues 1 and 21 (MAIN) were made to the same value. The atomic displacement parameters of atoms Al1 to C9 of residues 1 and 21 (MAIN) were restrained with the RIGU keyword in ShelXL input ('enhanced rigid bond' restraint for all bonds in the connectivity list values of 0.004 for both parameters s1 and s2 were used). The displacement parameters of atoms Al1 to C9 of the residues 1 and 21 (MAIN) were restrained to the same value with similarity restraint SIMU.

Crystal data for 8·C_6H_{14} (CCDC: 2107370)

Table

Data	**MaHa139_DeDh_a**
Empirical formula	$C_{68}H_{88}Al_2N_4$
Formula weight (g·mol^{-1})	1015.38
Temperature (K)	100.00(10)
Radiation, λ (Å)	$Cu_{K\alpha}$ 1.54184
Crystal system	
Space group	$P\bar{1}$
Unit cell dimensions	
a (Å)	11.88400(10)
b (Å)	15.41410(10)
c (Å)	18.41380(10)
α (°)	112.4900(10)
β (°)	90.8700(10)
γ (°)	105.4900(10)
Volume (Å^3)	2977.13(4)
Z	2
Calculated density (Mg·m^{-3})	1.133
Absorption coefficient (mm^{-1})	0.759
F(000)	1100
Theta range for collection	2.620 to 74.804°
Reflections collected	129644
Independent reflections	11861
Minimum/maximum transmission	0.82974/1.00000
Refinement method	Full-matrix least-squares on F^2
Data / parameters / restraints	11861 / 688 / 0
Goodness-of-fit on F^2	1.079
Final R indices [I>2σ(I)]	R_1 = 0.0423, wR^2 = 0.1159
R indices (all data)	R_1 = 0.0480, wR^2 = 0.1198
Maximum/minimum residual electron density (e·Å^{-3})	0.571 / –0.744

2. Computational chemistry

2.1. Computational details

All computations were carried out using the Gaussian 09 (Revision E.01) software.[7] Geometry optimizations and vibrational frequency calculations were performed in the gas phase using the dispersion corrected DFT functional theory, specifically the PBE1PBE functional,[8] def2-SVP basis set,[9] and Grimme's D3 correction (GD3) with Becke-Johnson damping (BJ).[10] A similar level of theory has been employed before for geometry optimizations and electronic structure calculations of low-valent aluminium compounds.[11] All geometry optimizations were performed without any symmetry constraints. Radicals and triplet state species were computed at the unrestricted level. Transition state geometries were obtained using opt = (ts, noeigentest, calcfc) algorithms.[12]

All optimized transition state structures were confirmed as maxima with only one imaginary frequency, and the magnitudes of all frequencies were verified to be greater than the residual frequencies due to rotations and translations. Additionally, each transition state established was ensured to be on the preferred reaction path by performing "plus-and-minus-displacement" minimization calculations, which involves the displacement of transition state structure by ca. 0.05 Å or 5° along the imaginary frequency normal mode in both directions,[13] and the displaced geometries were subsequently optimized to the nearest minimum. Zero-point vibrational energies and thermal corrections were obtained from frequency calculations with a standard state of 298 K and 1 atm. Single-point energies were computed on the PBE1PBE-D3BJ/def2-SVP optimized geometries using the PBE1PBE-D3BJ/def2-TZVP level of theory combined with the SMD solvation model (SCRF = SMD) for inclusion of the benzene solvent effect (ε=2.2706).[14] Dissociation energies computed for dialumenes, the dimers of alumylenes, also includes the counterpoise correction to avoid the basis set superposition error (BSSE).[15] The energies (ΔG) given are corrected for zero-point vibrational energies (ZPVEs).

2.2. Homocoupling of the radical [(NHC)Ar*Al·]

1) Radical **[(NHC)Ar*Al·]** (Ar* = 2,6-$C_6H_3Mes_2$, Mes = 2,4,6-$Me_3C_6H_2$)

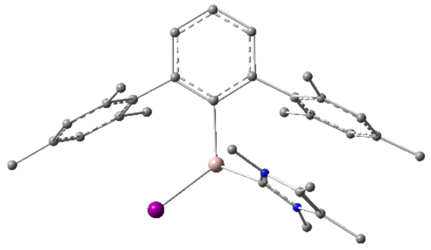

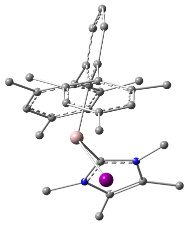

(*rotated view)*

Hydrogen atoms are omitted for clarity.

Number of imaginary frequencies = 0

E_{total} = -1850.759657 a.u

$G_{correction}$ = 0.531945 a.u

Cartesian coordinates:

C	0.46578300	-1.47196500	0.24631600
C	-0.66249700	-2.28645000	0.52599600
C	-0.52915100	-3.49205500	1.22518900
H	-1.42054100	-4.09459300	1.42252400
C	0.72054800	-3.93203000	1.65000600
H	0.81901900	-4.87603600	2.19185200
C	1.84506800	-3.16878200	1.35487700
H	2.83903800	-3.51222100	1.65522100
C	1.72981500	-1.95955300	0.65692300
C	2.98894100	-1.23448100	0.31852900
C	3.53482400	-1.36916400	-0.97260900
C	4.71649400	-0.69387300	-1.28369200
H	5.13242700	-0.79591900	-2.29067300
C	5.37842400	0.10341000	-0.34923600
C	4.83307000	0.20152800	0.93124200
H	5.33491000	0.82186800	1.68019300
C	3.64834600	-0.44834900	1.28029600
C	3.05621700	-0.23491800	2.64288300
H	2.23107600	0.49463900	2.58032600
H	3.80124800	0.17166400	3.34126100
H	2.64112800	-1.15873900	3.06937700
C	6.61547700	0.86681100	-0.72235400
H	7.18279300	0.35771000	-1.51531500
H	7.28163500	1.00713800	0.14168000
H	6.35167000	1.86930900	-1.09947000
C	2.86049900	-2.22159400	-2.00992500
H	3.50211900	-2.35061400	-2.89261500
H	1.91448800	-1.76327000	-2.34645600
H	2.60301600	-3.21522700	-1.61309700
C	-2.03333600	-1.92502100	0.05279300
C	-2.38736700	-2.14512600	-1.29702600

C	-3.67863400	-1.82687900	-1.72152100
H	-3.94718100	-2.00967200	-2.76700700
C	-4.63561900	-1.29357600	-0.85284600
C	-4.27408100	-1.10809300	0.48074700
H	-5.01136300	-0.70914800	1.18463000
C	-2.99860400	-1.43649400	0.95266100
C	-2.69994600	-1.30241000	2.41843400
H	-2.94942000	-2.23446800	2.95236000
H	-3.29703600	-0.49845800	2.87419600
H	-1.63555100	-1.11474200	2.60535900
C	-6.01851500	-0.96802300	-1.34186800
H	-5.99475400	-0.32650900	-2.23785900
H	-6.60955300	-0.45357200	-0.57049600
H	-6.56652800	-1.88179500	-1.62380400
C	-1.40753200	-2.74598300	-2.26345400
H	-1.90197500	-3.02779000	-3.20343400
H	-0.92286400	-3.63666700	-1.83688200
H	-0.59861700	-2.03384500	-2.50277600
Al	0.24058500	0.20880300	-0.83361200
C	-1.42526400	1.16290400	-0.10641500
N	-1.63653800	1.58130800	1.15824700
C	-2.85044800	2.23814500	1.27438700
C	-3.41137500	2.22333100	0.02659700
N	-2.51014500	1.56797400	-0.79482000
C	-2.68766200	1.39811400	-2.21828000
H	-1.85377100	0.78794600	-2.59491900
H	-2.67306200	2.37491600	-2.72410800
H	-3.63210900	0.88080700	-2.42800100
C	-4.71331200	2.73524600	-0.46398300
H	-4.58699500	3.45477100	-1.28822900
H	-5.25351700	3.24460700	0.34411000
H	-5.35148400	1.91536500	-0.83067700
C	-3.34796400	2.79966900	2.55411500
H	-4.31162400	3.30147200	2.39925700
H	-2.64904600	3.53977500	2.97459000
H	-3.49933300	2.01586300	3.31424400
C	-0.66592600	1.45213200	2.22162500
H	-0.03467100	2.35143900	2.27809600
H	-0.02229300	0.58890900	2.00962000
H	-1.17645400	1.28928600	3.17845800
I	1.85597600	2.20693500	-0.54341500

2) Diiododialane **[(NHC)(I)Ar*Al–AlAr*(I)(NHC)]** (Ar* = 2,6-$C_6H_3Mes_2$, Mes = 2,4,6-$Me_3C_6H_2$)

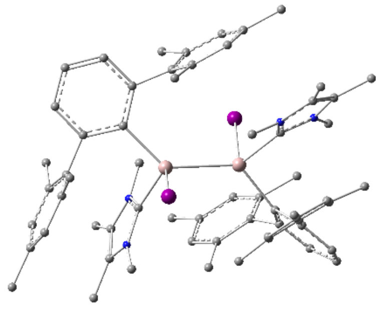

Hydrogen atoms are omitted for clarity.

Number of imaginary frequencies = 0

E_{total} = -3701.620088 a.u

$G_{correction}$ = 1.103474 a.u

Cartesian coordinates:

C	1.89033600	1.97995100	-1.40856900
C	2.73763000	1.53005300	-2.46306000
C	3.42398700	2.42753300	-3.29507500
H	4.04398800	2.02789400	-4.10175800
C	3.30493700	3.79933900	-3.12872700
H	3.85068900	4.49172800	-3.77450900
C	2.43452600	4.26914100	-2.15709400
H	2.26556900	5.34392400	-2.04768400
C	1.72010300	3.38966900	-1.32752900
C	0.69064300	4.08737700	-0.49850300
C	-0.64196700	4.06889400	-0.95047100
C	-1.59378000	4.86564300	-0.30829300
H	-2.61262500	4.87958400	-0.70383000
C	-1.26801000	5.66768900	0.78654200
C	0.06343000	5.68595900	1.20590300
H	0.34993400	6.32133900	2.04956100
C	1.05146100	4.93720200	0.56494400
C	-2.30797100	6.49082000	1.49180000
H	-3.19201000	6.65592000	0.85856300
H	-1.91229200	7.47494700	1.78452600
H	-2.64977800	5.99731200	2.41794200
C	2.89007400	0.10030700	-2.87381900
C	1.79954800	-0.54551800	-3.49720000
C	1.96888700	-1.82908200	-4.02079000
H	1.11432100	-2.31655600	-4.49685200
C	3.19050800	-2.50155600	-3.94972400
C	4.26853400	-1.83240700	-3.36782600
H	5.24654500	-2.32371200	-3.33303600
C	4.14811800	-0.53698100	-2.85426000
C	5.38906400	0.16110800	-2.37143400
H	5.90526400	0.65734900	-3.20999800
H	6.09694300	-0.55520600	-1.93100600
H	5.16546600	0.94148900	-1.63527700
C	3.32430800	-3.90615200	-4.46080900
H	2.87686500	-4.62513300	-3.75373700
H	4.37758300	-4.18869100	-4.60201100
H	2.80222000	-4.03666400	-5.42057900
C	0.49027900	0.16396500	-3.68235300
H	-0.17871800	-0.41653600	-4.33096100
H	0.64407100	1.16307500	-4.11769500
H	-0.02544200	0.30918500	-2.72158000
Al	1.23845800	0.62884300	0.03223000
C	3.05570200	-0.36434900	0.29429600
N	4.23479000	0.16884000	0.69741400
C	5.22528100	-0.79494700	0.76592100
C	4.64660000	-1.96621500	0.36328300
N	3.32677800	-1.66779400	0.09437300
C	2.38968500	-2.60868800	-0.46406900
H	1.37814700	-2.31109300	-0.17956900
H	2.58118800	-3.61165600	-0.07144400

H	2.45874000	-2.61277600	-1.55895600
C	5.22552400	-3.31801700	0.17404100
H	4.73915100	-4.06843600	0.81676900
H	6.29539200	-3.31166200	0.41893000
H	5.11880700	-3.65328000	-0.86985000
C	6.61721000	-0.49982100	1.18270000
H	7.19942600	-1.42862700	1.23671700
H	6.65511600	-0.02458100	2.17550300
H	7.12633600	0.17226000	0.47190100
C	4.48092600	1.56938800	0.95686300
H	4.35658800	1.80527100	2.02288300
H	3.76792300	2.16914300	0.38029600
H	5.49792300	1.82299100	0.63102700
Al	-1.08215300	-0.81479200	0.10026100
C	-2.01728600	-1.89681900	1.60689200
C	-2.53838700	0.59435700	-0.42709500
C	-3.40676200	-2.22434100	1.57273900
C	-1.30200700	-2.43735700	2.70885100
N	-2.91455700	1.55472300	0.44960100
N	-3.28114200	0.83827600	-1.53038200
C	-4.01103400	-2.99592500	2.57544500
C	-4.36628200	-1.82836500	0.49359800
C	-1.92840500	-3.20417400	3.70435200
C	0.16925600	-2.30015500	2.92662300
C	-3.89588200	2.37341000	-0.07607900
C	-4.12483900	1.91856300	-1.34348400
H	-5.07914800	-3.21678100	2.49120500
C	-3.28411900	-3.48444200	3.65088400
C	-4.58246500	-2.68198400	-0.60803600
C	-5.21789300	-0.72466600	0.68575300
H	-1.31876600	-3.58903700	4.52693100
C	1.03361300	-3.29304300	2.42764200
C	0.65680600	-1.33413200	3.82589300
C	-4.53833300	3.46523800	0.69517400
C	-5.06120100	2.39588300	-2.38777800
H	-3.76536400	-4.08414100	4.42746000
C	-5.51798000	-2.31409400	-1.57836900
C	-3.95871800	-4.04585800	-0.66434300
C	-6.15101700	-0.39382200	-0.30158300
C	-5.21870900	0.01685200	1.99019800
C	2.38856500	-3.24270600	2.76784600
C	2.02166800	-1.30071400	4.11524800
H	-5.15612300	4.08641500	0.03356600
H	-3.79402400	4.11761800	1.17138200
H	-5.19315900	3.07077600	1.48903200
H	-4.53437400	2.65764500	-3.31919500
H	-5.59276500	3.29095200	-2.04012600
H	-5.81433600	1.63116000	-2.63332000
H	-5.66461900	-2.97292100	-2.44026400
C	-6.29793700	-1.16124900	-1.45786500
H	-3.84274000	-4.39515600	-1.69937200
H	-4.61279700	-4.76116000	-0.13540900
H	-2.97702600	-4.07716400	-0.17910100
H	-6.79728200	0.47632200	-0.14843900
H	-5.83011300	-0.52665300	2.72997600
H	-5.65236700	1.02076400	1.88475400
H	-4.21529400	0.09211600	2.42541400

H	3.05524600	-4.02712100	2.39571700
C	2.90825500	-2.24092800	3.58888600
H	2.39786700	-0.52101100	4.78354000
C	-7.29542200	-0.79670300	-2.52123300
H	-6.80132300	-0.55259900	-3.47696400
H	-7.90240400	0.07142200	-2.22611700
H	-7.98307800	-1.63164100	-2.72874600
I	-0.95689500	-2.65640500	-1.81941000
I	1.29196900	1.92149600	2.34736100
C	-3.21511800	0.12417800	-2.78323000
H	-2.61323000	-0.78140800	-2.65370200
H	-4.22389200	-0.17133800	-3.09660200
H	-2.75490700	0.75791800	-3.55669400
C	-2.30017000	1.79092900	1.73535000
H	-1.74134000	0.89984800	2.04313900
H	-1.59804500	2.63461000	1.67506300
H	-3.06811200	1.99731300	2.49119400
C	-1.02832200	3.27513500	-2.16445200
H	-0.91935100	2.19576700	-1.98907200
H	-2.06993200	3.47715600	-2.44897400
H	-0.37629400	3.51573700	-3.01802500
C	2.48248100	5.08958700	0.99134700
H	3.00512900	5.82896600	0.36164000
H	2.54747400	5.42910900	2.03453700
H	3.02849500	4.14231400	0.90729100
C	-0.28977100	-0.41649500	4.54204200
H	0.24672000	0.40365500	5.03632400
H	-1.03068400	0.02010900	3.86023700
H	-0.85654400	-0.97573200	5.30524200
C	4.36832000	-2.18799000	3.93619800
H	4.53076900	-2.36303400	5.01226600
H	4.94503500	-2.94271400	3.38257700
H	4.79358800	-1.19950300	3.70214700
C	0.49084300	-4.43538400	1.61725300
H	1.29259400	-5.11897400	1.30230000
H	-0.23748000	-5.01605000	2.20509000
H	-0.04527300	-4.09171800	0.71891700

2.3. Free energy differences among selected experimental compounds

3) Masked dialumene **3**

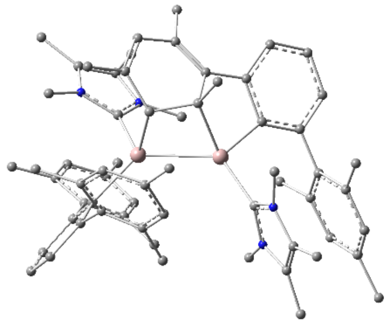

Hydrogen atoms are omitted for clarity.

Number of imaginary frequencies = 0

E_{total} = -3106.010239 a.u

$G_{correction}$ = 1.109388 a.u

Cartesian coordinates:

C	-1.65420700	-1.53546000	1.72554900
C	-2.56539300	-2.59504400	1.57872900
C	-2.64766400	-3.61367500	2.53995400
H	-3.37190200	-4.42357200	2.40924400
C	-1.83469800	-3.57566700	3.67128400
H	-1.93024500	-4.34877400	4.43872000
C	-0.91899200	-2.54087900	3.83791000
H	-0.32518600	-2.49464900	4.75210200
C	-0.79477300	-1.53200700	2.86525800
C	1.99864100	1.53218200	-1.69441500
C	2.71387600	0.79600500	-2.67685400
C	3.17649500	1.40580500	-3.85267900
H	3.71861500	0.80030800	-4.58508100
C	2.94371800	2.75311800	-4.10302400
H	3.30948900	3.21751400	-5.02237100
C	2.21756500	3.49304400	-3.17622100
H	1.99494200	4.54765200	-3.36446600
C	1.74692100	2.89431900	-2.00207600
C	-3.50626800	-2.66889400	0.42069600
C	-4.73726900	-1.99021400	0.47339100
C	-5.63481700	-2.11066600	-0.59389600
H	-6.58694300	-1.57259200	-0.54601300
C	-5.36012000	-2.91476900	-1.70129100
C	-4.13421000	-3.58769200	-1.73303800
H	-3.89065300	-4.21763700	-2.59474900
C	-3.19995000	-3.46533700	-0.70149300
C	-1.86724500	-4.14878600	-0.80585000
H	-1.82753400	-4.81873400	-1.67651100
H	-1.63769000	-4.72944700	0.09942400
H	-1.06154000	-3.40441300	-0.90781800
C	-5.10145900	-1.17317600	1.67890700
H	-5.97325300	-0.53276500	1.47937600
H	-4.25755000	-0.54961200	2.00508700
H	-5.34460700	-1.82732400	2.53187900

C	-6.34621400	-3.06203500	-2.82550100
H	-5.88998800	-2.82361300	-3.79992700
H	-7.21575700	-2.40329600	-2.68782700
H	-6.72126100	-4.09604100	-2.89473100
C	0.90694400	3.72106900	-1.08800100
C	-0.49410700	3.60523800	-1.15666000
C	-1.28030700	4.31528800	-0.24679000
H	-2.36840400	4.21372500	-0.30450200
C	-0.71606900	5.15416500	0.71656400
C	0.67122200	5.31746400	0.71138100
H	1.13263000	5.99749200	1.43401600
C	1.49078700	4.62479600	-0.18373600
C	2.97439800	4.85500100	-0.19432900
H	3.52818300	3.90535200	-0.22811200
H	3.29929900	5.42397900	0.68754400
H	3.27901200	5.41730700	-1.09181900
C	-1.57134800	5.83799000	1.74316800
H	-2.59038500	6.01648600	1.36785500
H	-1.14698300	6.80569500	2.04850900
H	-1.65678900	5.21905000	2.65297100
C	-1.13007300	2.76227300	-2.22253400
H	-2.22563200	2.84542300	-2.19128800
H	-0.85188000	1.70496800	-2.09445000
H	-0.78131700	3.06537400	-3.22173400
C	2.97193300	-0.67389200	-2.56571500
C	1.92352800	-1.58466400	-2.81747200
C	2.17599500	-2.95527900	-2.76226200
H	1.35376600	-3.64954200	-2.96445900
C	3.44226600	-3.46255100	-2.45798900
C	4.47091300	-2.54937700	-2.23514600
H	5.47514400	-2.92073100	-2.00722000
C	4.26416500	-1.16731500	-2.31076200
C	5.43818400	-0.24356800	-2.15693900
H	5.95823200	-0.11109000	-3.12041900
H	6.17380700	-0.65142800	-1.44726100
H	5.13131800	0.75596900	-1.82634900
C	3.68656900	-4.94434200	-2.41767200
H	2.89283600	-5.47275100	-1.86611900
H	4.64957200	-5.18330900	-1.94355900
H	3.70559100	-5.37222500	-3.43386400
C	0.55230800	-1.08738200	-3.15973400
H	-0.12217400	-1.91977400	-3.40705500
H	0.58021100	-0.38654500	-4.00829300
H	0.12837900	-0.53381000	-2.30491500
C	0.17579300	-0.41914700	2.95859100
C	1.42441500	-0.49507300	3.52059700
C	2.24352300	0.70614900	3.59444700
H	3.08903800	0.70808600	4.29232300
C	2.00528800	1.78937700	2.80657000
C	0.91423400	1.70836900	1.82066800
H	0.59027700	2.71199000	1.49841500
C	-0.29520700	0.92709200	2.38892200
C	-0.98009000	1.67754900	3.53685600
H	-1.30370200	2.68607700	3.22456800
H	-0.29250000	1.81440800	4.39214500
H	-1.85981200	1.12937300	3.91731600
C	2.05664700	-1.74166700	4.07922000

H	1.61073000	-2.66112100	3.67629000
H	1.96265600	-1.79199800	5.17861400
H	3.13459800	-1.75305300	3.85211900
C	2.80754100	3.04441600	2.93836400
H	3.21165800	3.37051200	1.96814800
H	3.64189400	2.93610600	3.64905200
H	2.16388800	3.86958800	3.28912300
Al	1.23500800	0.69534800	0.01692500
Al	-1.20107600	0.14247900	0.73690000
C	2.67927500	-0.70458300	0.52109800
N	3.95348800	-0.42740700	0.87096100
C	4.63685500	-1.57035200	1.25328500
C	3.74783800	-2.60014400	1.12820500
N	2.56488900	-2.03851800	0.68230100
C	1.34330200	-2.78418100	0.49990700
H	0.58751200	-2.10065700	0.09062300
H	0.96934100	-3.15692100	1.46392200
H	1.50473100	-3.61877600	-0.19384500
C	3.89241500	-4.05152900	1.38697800
H	3.17809300	-4.39834100	2.15067000
H	4.90492800	-4.27885600	1.74472700
H	3.71730900	-4.64053200	0.47301800
C	6.04819800	-1.55115300	1.70795000
H	6.37181800	-2.56272300	1.98518600
H	6.18205700	-0.90340500	2.58940300
H	6.73106300	-1.18607800	0.92330300
C	4.52047700	0.89749400	0.91922700
H	3.85499200	1.58137500	0.37885200
H	5.50627300	0.90843000	0.43786600
H	4.60968900	1.23549300	1.95949300
C	-2.94125500	0.87737200	-0.10645000
N	-3.42040200	0.53383600	-1.31935200
C	-4.60793200	1.18103500	-1.60511800
C	-4.87863800	1.97093800	-0.52131400
N	-3.84220900	1.76140200	0.37395600
C	-3.77202400	2.36093700	1.68297900
H	-4.65358300	2.08466400	2.27959300
H	-3.71130500	3.45618900	1.61078000
H	-2.87130700	1.99228100	2.18274500
C	-6.01154200	2.88545300	-0.23790700
H	-6.70387400	2.90903400	-1.08919000
H	-5.66929600	3.91675000	-0.05569700
H	-6.58222000	2.56613300	0.64928600
C	-5.35468800	0.95484000	-2.86552400
H	-5.63354500	-0.10561900	-2.97180800
H	-4.75997400	1.23473800	-3.74947400
H	-6.27621200	1.55079300	-2.87850300
C	-2.78316800	-0.40629100	-2.20688200
H	-3.44594100	-1.26009300	-2.39416800
H	-1.86869500	-0.76637300	-1.71968700
H	-2.51041800	0.08105000	-3.15410900

4) Dialumene **6**

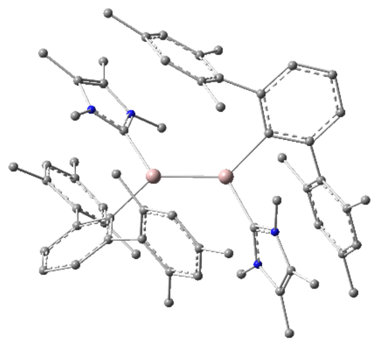

Hydrogen atoms are omitted for clarity.

Number of imaginary frequencies = 0

E_{total} = -3105.987663 a.u

$G_{correction}$ = 1.107864 a.u

BSSEcorrection = 0.001372124 a.u

Cartesian coordinates:

C	-1.63079600	2.45833000	0.14831300
C	-2.70765400	2.83101100	1.00030100
C	-3.18894300	4.14711700	1.02898400
H	-4.01006500	4.39902100	1.70613900
C	-2.62357800	5.13237700	0.22726900
H	-3.01024500	6.15447400	0.24876800
C	-1.54064500	4.80404200	-0.58140200
H	-1.06058600	5.57155300	-1.19569300
C	-1.03903100	3.49508400	-0.61592400
C	0.19662800	3.31688500	-1.44558600
C	1.42837400	3.70382500	-0.88208400
C	2.56958800	3.75309900	-1.68725800
H	3.51448900	4.07804600	-1.24120000
C	2.52653500	3.42910500	-3.04438500
C	1.30350300	3.01316700	-3.57733800
H	1.24480900	2.74419000	-4.63686300
C	0.14150900	2.95213000	-2.80523600
C	-1.13607800	2.47243900	-3.41967100
H	-1.99763100	3.06869900	-3.08494100
H	-1.31401000	1.42791900	-3.10327100
H	-1.09119900	2.49665100	-4.51777900
C	3.75223600	3.51665300	-3.90950300
H	4.53191800	4.13538400	-3.44148900
H	3.51943100	3.95538100	-4.89158100
H	4.18859200	2.52131500	-4.10191500
C	1.51195600	4.09431100	0.56589300
H	2.55891700	4.15354800	0.89686500
H	0.97603300	3.37113500	1.19777900
H	1.03826300	5.07233000	0.74769200
C	-3.33887500	1.87171500	1.95829800
C	-2.60980600	1.42151300	3.08392900
C	-3.22309800	0.56434900	3.99790300
H	-2.64834700	0.22711800	4.86657100
C	-4.53951800	0.12267600	3.83696300
C	-5.25292300	0.60181800	2.74020500
H	-6.29254800	0.28855300	2.60039400

C	-4.68779800	1.48981700	1.81821500
C	-5.55843400	2.04027800	0.72418300
H	-6.03866100	2.98123900	1.04046700
H	-6.36475900	1.33567000	0.47409600
H	-4.98937800	2.27407300	-0.18340200
C	-5.16104800	-0.82040700	4.82770200
H	-4.58286600	-1.75517500	4.91643500
H	-6.18884700	-1.08647700	4.54142500
H	-5.19952700	-0.37617800	5.83551500
C	-1.19290700	1.85212900	3.31288700
H	-0.87534400	1.63499600	4.34295000
H	-1.05956800	2.92569700	3.11449000
H	-0.52240100	1.30939500	2.62029900
Al	-1.04127700	0.55196000	-0.12579700
C	-2.77684200	-0.51273000	-0.02529900
N	-3.85481600	-0.33838100	-0.82651400
C	-4.87727800	-1.21355200	-0.50360600
C	-4.41882300	-1.95753700	0.54795600
N	-3.13538000	-1.51419000	0.81334400
C	-2.33279100	-2.01309700	1.90441400
H	-1.39193300	-1.43717600	1.92028400
H	-2.09484500	-3.07494100	1.75768500
H	-2.86593900	-1.87686200	2.85491800
C	-5.07571500	-3.01831700	1.34834700
H	-4.48956200	-3.95118100	1.34992000
H	-6.07002800	-3.24647500	0.94310900
H	-5.20389500	-2.70500600	2.39718700
C	-6.18029700	-1.25469000	-1.21182700
H	-6.81376000	-2.04400300	-0.78683200
H	-6.05833800	-1.46626500	-2.28506800
H	-6.72926400	-0.30355800	-1.11906900
C	-3.86491300	0.58621900	-1.93491700
H	-3.26654800	0.18215100	-2.76397500
H	-3.42838600	1.54241200	-1.61955300
H	-4.89558600	0.75716600	-2.26700100
Al	1.01931500	-0.63522000	0.06740900
C	1.67542000	-2.40982100	-0.60896700
C	2.71739300	0.48174900	0.26862800
C	2.83451500	-3.01609400	-0.04727000
C	1.06010200	-3.09608400	-1.68676700
N	3.68277200	0.70110800	-0.65218900
N	3.14371700	1.14046300	1.37151000
C	3.37302300	-4.19336300	-0.58214800
C	3.50067200	-2.45520900	1.16749900
C	1.62276400	-4.26444500	-2.21859100
C	-0.26136400	-2.70462800	-2.27387100
C	4.70013400	1.49012500	-0.14570500
C	3.58986700	0.24950000	-2.02042500
C	4.35857400	1.76390000	1.15042900
C	2.44816000	1.16227600	2.63491000
H	4.25952900	-4.63423200	-0.11700500
C	2.78504700	-4.80996300	-1.68132800
C	2.86000400	-2.54107400	2.42661800
C	4.79916500	-1.91413000	1.09432800
H	1.12552700	-4.75521500	-3.06060200
C	-1.41239100	-3.34985300	-1.78442800
C	-0.36774500	-1.85007600	-3.38969700

C	5.88187700	1.90699800	-0.93935600
H	3.02020700	0.97597900	-2.61729000
H	3.06776700	-0.71437500	-2.04083700
H	4.59539500	0.12419300	-2.44013800
C	5.06963800	2.52354800	2.20662700
H	1.60357800	0.45848300	2.56095300
H	2.05692800	2.16771700	2.84834300
H	3.12081200	0.84367900	3.44155500
H	3.21519100	-5.72248000	-2.10181700
C	3.49884400	-2.02708400	3.55496100
C	1.51519700	-3.19098900	2.56990900
C	5.39258300	-1.38598000	2.24665300
C	5.58670700	-1.91820300	-0.18513200
C	-2.64044000	-3.15223400	-2.42345900
C	-1.31894200	-4.29933400	-0.62411500
C	-1.60774400	-1.69202700	-4.01153800
C	0.81889100	-1.08983700	-3.89414700
H	6.53115400	2.55702500	-0.33862000
H	5.58922100	2.46566700	-1.84172900
H	6.48533100	1.04409600	-1.26511100
H	4.44122900	3.32490000	2.62689600
H	5.97826800	2.98703900	1.80099900
H	5.37054900	1.86651600	3.03902800
H	2.99333600	-2.10155100	4.52320400
C	4.75917300	-1.42451300	3.48688200
H	1.28574000	-3.39400100	3.62554900
H	1.46198200	-4.13257000	2.00385300
H	0.72925200	-2.52745000	2.16389500
H	6.39019500	-0.94171500	2.16905500
H	6.19299700	-2.83576000	-0.26851300
H	6.28247400	-1.06718300	-0.22057000
H	4.93835900	-1.89376300	-1.06892100
H	-3.52542400	-3.66813900	-2.03879400
C	-2.75522400	-2.34847200	-3.55661800
H	-2.31597200	-4.52548700	-0.21831000
H	-0.68573100	-3.89227300	0.17670100
H	-0.85469300	-5.25162600	-0.92782000
H	-1.67318800	-1.04727000	-4.89426700
H	1.73146000	-1.70356100	-3.88305100
H	0.98952200	-0.22143800	-3.23283300
H	0.65490700	-0.71728000	-4.91546600
C	5.40833100	-0.85982100	4.71850700
C	-4.05867000	-2.21664800	-4.29262200
H	4.79479100	-0.06425700	5.17358800
H	6.39737300	-0.43447600	4.49462800
H	5.54445500	-1.63340500	5.49131100
H	-4.85240700	-2.80526700	-3.81047500
H	-3.96544000	-2.57436700	-5.33053800
H	-4.39822500	-1.16945900	-4.34917900

5) Alumylene 7 (**[(NHC)Ar*Al:]** (Ar* = 2,6-$C_6H_3Mes_2$, Mes = 2,4,6-$Me_3C_6H_2$))

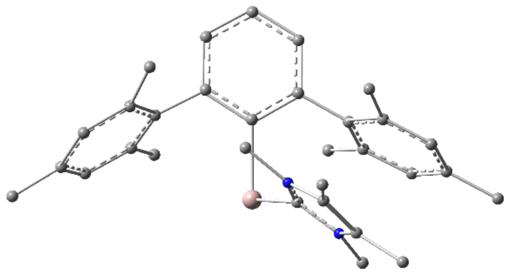

Hydrogen atoms are omitted for clarity.

Number of imaginary frequencies = 0

E_{total} = -1552.947063 a.u

$G_{correction}$ = 0.532312 a.u

Cartesian coordinates:

C	0.72120600	-1.07724100	0.31590700
C	-0.31976700	-1.79502100	0.94954500
C	-0.07187100	-2.63572500	2.04093300
H	-0.90482700	-3.17317500	2.50465100
C	1.22250800	-2.79326800	2.52979000
H	1.41547500	-3.45379800	3.37890000
C	2.26891500	-2.10760700	1.91987900
H	3.29231600	-2.22816300	2.28742800
C	2.02820100	-1.26379400	0.82800900
C	3.18127500	-0.55442200	0.19529900
C	3.68352500	-1.01140100	-1.04539300
C	4.72121100	-0.30992300	-1.65854300
H	5.09952700	-0.66733600	-2.62115600
C	5.29463700	0.82493300	-1.07799800
C	4.82727000	1.22305500	0.17295400
H	5.28764500	2.08602200	0.66501300
C	3.79418200	0.54342300	0.82878800
C	3.41732200	0.97163300	2.22174400
H	2.41741100	0.62448700	2.51188500
H	3.46331600	2.06621100	2.33013600
H	4.12098700	0.54730300	2.95745900
C	6.37828700	1.58542200	-1.78625400
H	7.12799500	0.90676900	-2.22112900
H	6.89738900	2.27726900	-1.10745100
H	5.96403000	2.18158600	-2.61633100
C	3.13356700	-2.24905100	-1.69126000
H	3.80745600	-2.61324800	-2.47932400
H	2.15313900	-2.04346600	-2.15905300
H	2.97798500	-3.05151900	-0.95555500
C	-1.72610200	-1.68805400	0.45524400
C	-2.16037600	-2.50351500	-0.61145600
C	-3.49604900	-2.43766400	-1.01846500
H	-3.82840200	-3.08093900	-1.83897300
C	-4.41745600	-1.58970400	-0.39856300
C	-3.96609900	-0.78736000	0.65173000
H	-4.67090900	-0.11780500	1.15551300
C	-2.63876400	-0.82538400	1.09102000
C	-2.21103000	0.01777000	2.25764500
H	-2.23095500	-0.56520100	3.19326700

H	-2.87592200	0.88445100	2.38607500
H	-1.18009700	0.37318600	2.13236300
C	-5.84079600	-1.52106700	-0.87354000
H	-5.93083100	-0.89854700	-1.78056900
H	-6.50139700	-1.08638800	-0.10955400
H	-6.22735200	-2.51824300	-1.13171700
C	-1.21625100	-3.47121700	-1.26726600
H	-1.72744600	-4.06100900	-2.04056700
H	-0.78325300	-4.16151200	-0.52716600
H	-0.37006000	-2.94825300	-1.74119900
Al	0.34087500	-0.06790000	-1.45297600
C	-0.90485400	1.43516700	-0.60707000
N	-0.63868200	2.37714100	0.32603300
C	-1.66425800	3.30355800	0.42704000
C	-2.61076200	2.92003200	-0.48373500
N	-2.10802000	1.79124400	-1.11003100
C	-2.78213300	1.05260900	-2.15169000
H	-2.02177800	0.47558700	-2.70461700
H	-3.28133000	1.74407300	-2.84360400
H	-3.51232000	0.35215600	-1.72471600
C	-3.93958900	3.49511400	-0.80710900
H	-3.99121500	3.86102100	-1.84531900
H	-4.16088900	4.34222000	-0.14519900
H	-4.74003800	2.74884600	-0.67837800
C	-1.63424300	4.43519100	1.38620900
H	-2.53346800	5.05483100	1.27584200
H	-0.75917400	5.08466400	1.22391500
H	-1.59659000	4.08500200	2.43083600
C	0.57943800	2.39853100	1.09518100
H	1.27073400	3.17482300	0.73296700
H	1.05713000	1.41701200	0.99243200
H	0.36439000	2.57496100	2.15810900

6) C-H activation intermediate **9**

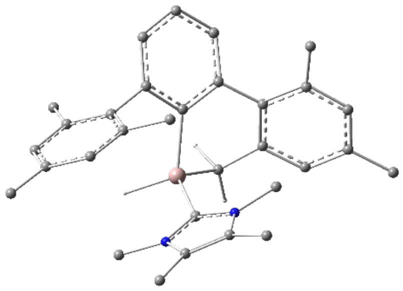

Selected hydrogen atoms are omitted for clarity.

Number of imaginary frequencies = 0

E_{total} = -1553.003677 a.u

$G_{correction}$ = 0.532657 a.u

Cartesian coordinates:

C	0.43557500	-1.45667400	0.12611300
C	1.75550900	-1.88944800	-0.16059000
C	1.95364600	-3.11142300	-0.82175300

H	2.96886700	-3.48454400	-0.97234600
C	0.87449500	-3.85376800	-1.29316800
H	1.04791300	-4.80449300	-1.80407100
C	-0.42143500	-3.36804200	-1.13932100
H	-1.27164300	-3.91596900	-1.55616700
C	-0.63812600	-2.18288600	-0.42529600
C	-2.02844500	-1.65142000	-0.29124900
C	-2.45937000	-0.63383500	-1.16389800
C	-3.76476000	-0.14268100	-1.05294700
H	-4.09567000	0.63868600	-1.74518600
C	-4.65702400	-0.63362100	-0.09792500
C	-4.20662200	-1.63737100	0.76396200
H	-4.88532700	-2.03306900	1.52602500
C	-2.91261100	-2.15762200	0.68204600
C	2.92283000	-1.00352000	0.13724400
C	3.04235800	-0.32964500	1.38578800
C	4.11261700	0.55481100	1.58027300
C	5.05355200	0.82769400	0.58855100
C	4.89271000	0.19866100	-0.64498400
H	5.59188100	0.42498100	-1.45602600
C	3.85038700	-0.70382200	-0.88940000
C	2.00625900	-0.44979600	2.44461900
H	-0.98788200	-0.55413700	2.68201000
Al	0.19331800	-0.18618100	1.64839000
C	-0.39605500	1.60982100	0.80132900
N	0.21089300	2.28767700	-0.19739200
C	-0.64209900	3.21285400	-0.77298900
C	-1.82438400	3.11661900	-0.09076800
N	-1.63716000	2.13835100	0.86888400
H	2.28319300	0.11784400	3.34434800
H	1.87090700	-1.50619100	2.74678600
H	4.19689500	1.05462400	2.55024000
C	-2.46229800	-3.22949300	1.62941400
H	-2.10423800	-4.11906500	1.09018200
H	-1.62075000	-2.86825300	2.24113500
H	-3.27476700	-3.53318700	2.30406200
C	-1.54160200	-0.09698800	-2.22465800
H	-2.00720700	0.73632100	-2.77004300
H	-0.59312500	0.24716000	-1.78857700
H	-1.27515300	-0.88233000	-2.94959800
C	3.73453500	-1.24687500	-2.29072700
H	4.19796300	-2.24046500	-2.39600600
H	2.68735200	-1.35445500	-2.60698700
H	4.24741600	-0.57757600	-2.99698200
C	-2.66773900	1.73272300	1.80058800
H	-3.54044800	1.35594600	1.25062600
H	-2.27210300	0.91792700	2.41911000
H	-2.95994300	2.58101000	2.43628000
C	1.55434700	2.03322300	-0.66948200
H	2.09922200	1.43439000	0.06784400
H	1.53481000	1.47744000	-1.61867200
H	2.08865100	2.98096700	-0.81490100
C	6.20635900	1.75397700	0.84659500
H	7.05067900	1.21298500	1.30635700
H	6.57847400	2.20683500	-0.08430800
H	5.92815300	2.56410900	1.53725900
C	-6.06396600	-0.11466500	-0.00537200

H	-6.78723500	-0.85677100	-0.38177800
H	-6.34658700	0.10661100	1.03580100
H	-6.19697800	0.80254000	-0.59775800
C	-3.11144400	3.83391500	-0.25981400
H	-3.39443100	4.39018900	0.64792300
H	-3.04374700	4.55321600	-1.08584600
H	-3.93018500	3.13259300	-0.48725300
C	-0.24498400	4.07246500	-1.91476100
H	0.59776300	4.73419600	-1.65800400
H	0.05969400	3.47134000	-2.78623200
H	-1.08497400	4.70774000	-2.22329000

7) C-H activation product **8**

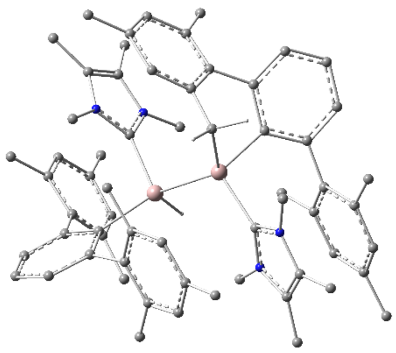

Hydrogen atoms are omitted for clarity.

Number of imaginary frequencies = 0

E_{total} = -3106.044656 a.u

$G_{correction}$ = 1.105305 a.u

Cartesian coordinates:

C	1.10147400	-2.56459600	-0.04982500
C	0.26408100	-3.47923500	-0.75183000
C	0.37370600	-4.86132500	-0.53510800
H	-0.21668500	-5.54703800	-1.14611300
C	1.23637800	-5.37467800	0.42607900
H	1.30619200	-6.45348500	0.58780900
C	2.01608100	-4.49692200	1.16711200
H	2.70106300	-4.87872700	1.93027500
C	1.96751300	-3.11519900	0.92138500
C	-0.73193600	-3.02941300	-1.77337800
C	-0.35427100	-2.09164800	-2.77777800
C	-1.25921400	-1.80308400	-3.80675600
H	-0.94108000	-1.11425800	-4.59561900
C	-2.53191400	-2.37625900	-3.87122800
C	-2.90881500	-3.23226900	-2.84060800
H	-3.91066800	-3.67309700	-2.84734000
C	-2.03798800	-3.56269500	-1.79310600
C	0.95981300	-1.40069100	-2.71646800
Al	1.04585100	-0.67184100	-0.81372200
H	1.08532200	-0.71986400	-3.57116600
H	1.76622200	-2.15259800	-2.74730300
H	-0.53436100	0.60771100	1.90056200
C	2.93092500	-2.30461500	1.72995100

C	4.28935600	-2.26788600	1.36117700
C	2.52291400	-1.69076000	2.92995200
C	5.20686900	-1.59279800	2.17169900
C	3.47593600	-1.05603300	3.73215900
C	4.82419500	-0.99174200	3.37166100
H	6.25791300	-1.55925400	1.86687200
H	3.15073300	-0.59962200	4.67291800
C	5.14709100	0.65033900	-0.68474500
C	4.66480700	1.27422800	0.43086900
C	2.94714000	0.13671600	-0.49901500
N	4.07136400	-0.02777600	-1.23637400
N	3.32789800	0.93998800	0.51573000
Al	-0.85431000	0.72788800	0.30361300
C	-1.48108700	2.64568900	-0.06102000
C	-2.60625600	-0.35774900	0.15663900
C	-2.66002800	3.15654600	0.55014800
C	-0.82119700	3.51022300	-0.96595100
N	-3.51322200	-0.33332100	-0.84330400
N	-3.09477800	-1.25629900	1.03784100
C	-3.17467600	4.41402300	0.21088000
C	-3.36868800	2.38934400	1.62037800
C	-1.36074600	4.75810800	-1.31583900
C	0.54222100	3.22250700	-1.51163500
C	-4.56523400	-1.19924200	-0.59856900
C	-4.30211600	-1.78095100	0.61023300
H	-4.07878700	4.77340200	0.71064000
C	-2.54484700	5.20620400	-0.74377600
C	-2.77278700	2.26945600	2.89638500
C	-4.64053900	1.83036600	1.39909800
H	-0.82428300	5.38689900	-2.03276000
C	1.64545000	3.80998000	-0.86066400
C	0.74205400	2.50141800	-2.70299400
H	-2.96033000	6.17909900	-1.01884200
C	-3.40115800	1.49397300	3.87081600
C	-5.23109100	1.04934500	2.39941000
C	2.92413100	3.65633500	-1.40306300
C	2.03542000	2.37589700	-3.21722000
H	-2.92629900	1.39846500	4.85244300
C	-4.61874900	0.84829700	3.63435600
H	-6.20684900	0.59315000	2.20413100
H	3.77509300	4.12248900	-0.89618600
C	3.13988000	2.95599600	-2.59038800
H	2.17507400	1.83030500	-4.15609000
C	4.12760900	-0.75842000	-2.48114400
H	3.53069700	-0.24941100	-3.24931700
H	5.16829500	-0.82722800	-2.81589300
H	3.73370000	-1.77121100	-2.34955300
C	-0.41332400	1.87338000	-3.41778200
H	-0.14899400	1.60524300	-4.45029400
H	-0.71413000	0.94882900	-2.89924400
H	-1.28630500	2.54197300	-3.43754700
C	1.44880600	4.63879800	0.37854900
H	2.40985200	4.86276700	0.86477700
H	0.96288500	5.59828200	0.13971900
H	0.78771500	4.13901300	1.10199900
C	-3.36193300	0.45470200	-2.04333400
H	-2.82879700	1.38052300	-1.79646700

H	-2.79100300	-0.10920800	-2.79293900
H	-4.34784100	0.71230800	-2.44789700
C	-2.43208700	-1.64277500	2.26028800
H	-1.89731900	-2.59576500	2.12795300
H	-1.71048100	-0.85635800	2.51499700
H	-3.16529700	-1.73627400	3.07090100
C	-2.57779000	-4.46700000	-0.71587000
H	-2.41203600	-5.53298100	-0.93972400
H	-2.10980700	-4.27085800	0.25840300
H	-3.66365900	-4.32400300	-0.62107900
C	4.77155100	-2.99222500	0.13752300
H	5.00076200	-4.04493500	0.37200200
H	4.00321200	-3.01392200	-0.64581900
H	5.68903200	-2.53574100	-0.26307800
C	1.08340500	-1.71471400	3.34594800
H	0.96433800	-1.39247700	4.39038600
H	0.48840000	-1.03548100	2.71299700
H	0.65247200	-2.72021900	3.23180900
C	2.47595800	1.41410000	1.58018900
H	2.36860300	2.50409000	1.52054200
H	1.48324400	0.95701900	1.48877500
H	2.90015900	1.12950300	2.55045500
C	-1.50612600	3.00375500	3.22796700
H	-1.31014900	2.97596300	4.30922100
H	-0.64930700	2.54444000	2.71200800
H	-1.56284500	4.05482900	2.90698800
C	-5.41366500	2.09385900	0.13847900
H	-6.08449400	1.25507000	-0.09605500
H	-6.04544700	2.99039300	0.25573100
H	-4.75951400	2.28462900	-0.71983400
C	4.50417200	2.89136000	-3.21290400
H	4.60357200	3.63708300	-4.01945300
H	5.29220500	3.09766100	-2.47497000
H	4.70226500	1.90731600	-3.66408000
C	-5.25127700	-0.01038100	4.69218500
H	-5.37309200	0.54021100	5.63839100
H	-6.24371700	-0.36803300	4.38166300
H	-4.63148200	-0.89412300	4.91924300
C	-3.47067600	-2.04089900	-4.99455900
H	-2.98116300	-2.15308600	-5.97447600
H	-4.35951400	-2.68854000	-4.98561100
H	-3.81855400	-0.99515400	-4.93221500
C	5.83547500	-0.34450000	4.27549300
H	6.16580900	-1.04290000	5.06285300
H	6.73356900	-0.03241100	3.72216500
H	5.42172500	0.53875000	4.78595500
C	-5.08506100	-2.74915300	1.41503400
H	-5.43434400	-2.29699500	2.35805100
H	-5.97013900	-3.08150500	0.85705300
H	-4.49373700	-3.64218200	1.67065000
C	-5.70062300	-1.38182800	-1.53486700
H	-6.37131300	-2.16925200	-1.16712300
H	-6.29780400	-0.46221800	-1.64836300
H	-5.34928100	-1.67943900	-2.53464000
C	5.33118000	2.14460100	1.42732300
H	6.39074800	2.27895600	1.17474300
H	4.86431800	3.14177000	1.46727000

H	5.27601200	1.70950200	2.43685700
C	6.52687900	0.60080300	-1.22883400
H	7.19060600	1.22248000	-0.61409600
H	6.93298400	-0.42405300	-1.21717100
H	6.58781400	0.97504000	-2.26233700

8) Pentalene product **4**

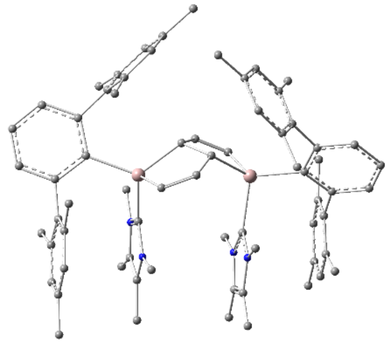

Hydrogen atoms are omitted for clarity.

Number of imaginary frequencies = 0

E_{total} = -3337.851511 a.u

$G_{correction}$ = 1.195506 a.u

Cartesian coordinates:

Al	2.06773300	0.49942100	-0.18904700
C	1.37286300	0.71864500	-2.02148600
H	1.74012400	0.54071600	-3.04133600
C	0.11745400	1.22699000	-1.89168800
H	-0.49289600	1.46876000	-2.77885800
C	-0.50456300	1.52995600	-0.58653800
H	-0.96567700	2.53045600	-0.66332100
N	1.70712400	-2.59098900	-0.44314900
C	1.88956000	-1.51145700	0.34370100
N	2.02668400	-2.01806700	1.58769300
C	1.96266900	-3.40252900	1.58690100
C	1.77030100	-3.76765300	0.28176200
C	1.48709700	-2.54666700	-1.87080400
H	2.35024600	-2.96601100	-2.40190000
H	1.35255400	-1.49409800	-2.15784200
H	0.57883800	-3.11356700	-2.11570800
C	2.16627100	-1.20265700	2.77112200
H	2.93250000	-1.62138700	3.43697400
H	1.20835500	-1.12251300	3.30516100
H	2.47413100	-0.19639900	2.46507500
C	3.99209300	0.96543500	0.33454100
C	4.31937400	2.16895800	1.01140300
C	5.63889000	2.48151300	1.36679000
H	5.83752600	3.42549300	1.88228100
C	6.68417100	1.62029800	1.05808800
H	7.71197300	1.87078000	1.33308000
C	6.40198300	0.44397300	0.37483700
H	7.21187600	-0.23686700	0.09620200

C	5.08579000	0.12060100	0.01552400
C	3.29011200	3.19230300	1.36061700
C	2.82544200	3.30337300	2.68446800
C	1.85682700	4.26072000	2.99041000
H	1.47936500	4.32312700	4.01591100
C	1.35443100	5.13371100	2.02390200
C	1.85748000	5.03365200	0.72637300
H	1.48195200	5.71048100	-0.04537500
C	2.81043500	4.07383200	0.37350100
C	3.36197700	2.41459100	3.77142900
H	2.66744000	2.36599800	4.62265900
H	4.32857400	2.78637300	4.15007400
H	3.55074000	1.39493300	3.40838200
C	3.25591200	3.95461900	-1.05490600
H	4.32777200	3.72284900	-1.13243600
H	3.05427800	4.88278200	-1.60869800
H	2.70986700	3.13829000	-1.56157600
C	4.93665800	-1.13874100	-0.77458000
C	4.82253700	-1.06607800	-2.17988800
C	4.79564400	-2.24915500	-2.92124300
H	4.71803500	-2.18441900	-4.01122200
C	4.87576600	-3.50671900	-2.31418200
C	5.00505300	-3.55497200	-0.92606600
H	5.09406400	-4.52655200	-0.42910800
C	5.05547200	-2.39231400	-0.14915800
C	4.80586100	0.26464600	-2.87315800
H	5.73016600	0.82363700	-2.65801900
H	3.97202500	0.88985800	-2.52342500
H	4.71715700	0.14361800	-3.96178300
C	5.29294100	-2.49695800	1.33017200
H	6.36305800	-2.37371500	1.56485100
H	4.98122200	-3.47949200	1.71343500
H	4.76563000	-1.70734500	1.88089200
C	-1.37260000	0.71690700	2.02200800
H	-1.73977400	0.53818800	3.04174900
C	-0.11734200	1.22572000	1.89250200
H	0.49298300	1.46708200	2.77981000
C	0.50455000	1.52967400	0.58754500
H	0.96552500	2.53018900	0.66492300
Al	-2.06754000	0.49892200	0.18945200
N	-1.70670500	-2.59156700	0.44083800
C	-1.88914700	-1.51139500	-0.34510300
N	-2.02634800	-2.01697100	-1.58950800
C	-1.96249200	-3.40144900	-1.58983400
C	-1.77004200	-3.76765500	-0.28501400
C	-1.48697300	-2.54836600	1.86857100
H	-2.35054100	-2.96749300	2.39915900
H	-1.35188800	-1.49606900	2.15635800
H	-0.57915400	-3.11602900	2.11331200
C	-2.16627300	-1.20071800	-2.77229800
H	-2.93502700	-1.61707800	-3.43675900
H	-1.20929700	-1.12296000	-3.30836700
H	-2.47072000	-0.19376400	-2.46520100
C	-3.99211600	0.96463000	-0.33358700
C	-4.31982500	2.16777400	-1.01090700
C	-5.63947200	2.47986600	-1.36619100
H	-5.83841200	3.42352100	-1.88215600

C	-6.68449400	1.61856200	-1.05685800
H	-7.71240900	1.86867500	-1.33176600
C	-6.40189500	0.44262500	-0.37311200
H	-7.21157700	-0.23826300	-0.09398200
C	-5.08554600	0.11968200	-0.01396600
C	-3.29082800	3.19115200	-1.36081300
C	-2.82647000	3.30186100	-2.68478000
C	-1.85814500	4.25936600	-2.99127000
H	-1.48089100	4.32148200	-4.01686800
C	-1.35578800	5.13283600	-2.02521300
C	-1.85854900	5.03312500	-0.72752300
H	-1.48301100	5.71031800	0.04389900
C	-2.81113300	4.07317400	-0.37410400
C	-3.36308500	2.41282300	-3.77151800
H	-2.66621000	2.35992400	-4.62059900
H	-4.32702700	2.78768500	-4.15393900
H	-3.55690200	1.39466900	-3.40698500
C	-3.25621400	3.95429700	1.05445700
H	-4.32801400	3.72236900	1.13234600
H	-3.05458000	4.88264800	1.60793100
H	-2.70992100	3.13820200	1.56123800
C	-4.93599400	-1.13932100	0.77661300
C	-4.82172700	-1.06608900	2.18187100
C	-4.79468800	-2.24887000	2.92372000
H	-4.71697100	-2.18368700	4.01366000
C	-4.87481900	-3.50667600	2.31718400
C	-5.00421300	-3.55550100	0.92908200
H	-5.09330100	-4.52728200	0.43252900
C	-5.05472700	-2.39317200	0.15170200
C	-4.80507100	0.26492100	2.87458600
H	-5.72940500	0.82379100	2.65925800
H	-3.97126000	0.88999300	2.52453300
H	-4.71629500	0.14435500	3.96325500
C	-5.29228700	-2.49842900	-1.32757200
H	-6.36237700	-2.37497100	-1.56225800
H	-4.98082400	-3.48120900	-1.71041200
H	-4.76476900	-1.70920600	-1.87865400
C	0.27966000	6.12226600	2.37064200
H	0.20840000	6.92231900	1.61976400
H	-0.70310500	5.62479300	2.42144300
H	0.45839000	6.58887800	3.35121500
C	4.80810400	-4.76217200	-3.13656100
H	3.78821300	-4.93826800	-3.52060400
H	5.47086000	-4.70576500	-4.01364200
H	5.09808700	-5.64572500	-2.54968700
C	-0.28128300	6.12149500	-2.37248500
H	-0.21033000	6.92204500	-1.62210700
H	0.70163000	5.62427000	-2.42285500
H	-0.46004900	6.58742700	-3.35337300
C	-4.80666300	-4.76183900	3.13995500
H	-3.78585600	-4.93935200	3.52093500
H	-5.46664000	-4.70398900	4.01901800
H	-5.09972900	-5.64522700	2.55436200
C	-2.10843100	-4.21609800	-2.82108900
H	-1.98900300	-5.28250200	-2.58914900
H	-3.10077900	-4.08484800	-3.28253100
H	-1.35618000	-3.95102800	-3.58172500

C	-1.71420000	-5.10011000	0.36283400
H	-2.58099900	-5.24399300	1.02774000
H	-1.72899500	-5.89953300	-0.38936300
H	-0.80591600	-5.22078200	0.97219800
C	2.10826500	-4.21830800	2.81745700
H	1.35596300	-3.95375800	3.57822000
H	1.98861200	-5.28446900	2.58451800
H	3.10057700	-4.08775500	3.27917900
C	1.71440700	-5.09959400	-0.36712200
H	2.58133300	-5.24312100	-1.03193400
H	1.72890800	-5.89959700	0.38446500
H	0.80623900	-5.21966200	-0.97678200

2.4. Optimized structures, frontier orbitals and dissociation of dialumenes 6 and II

9) Dialumene **6**

Note: Structural details and energies of **6** are already given in Section 2.3.

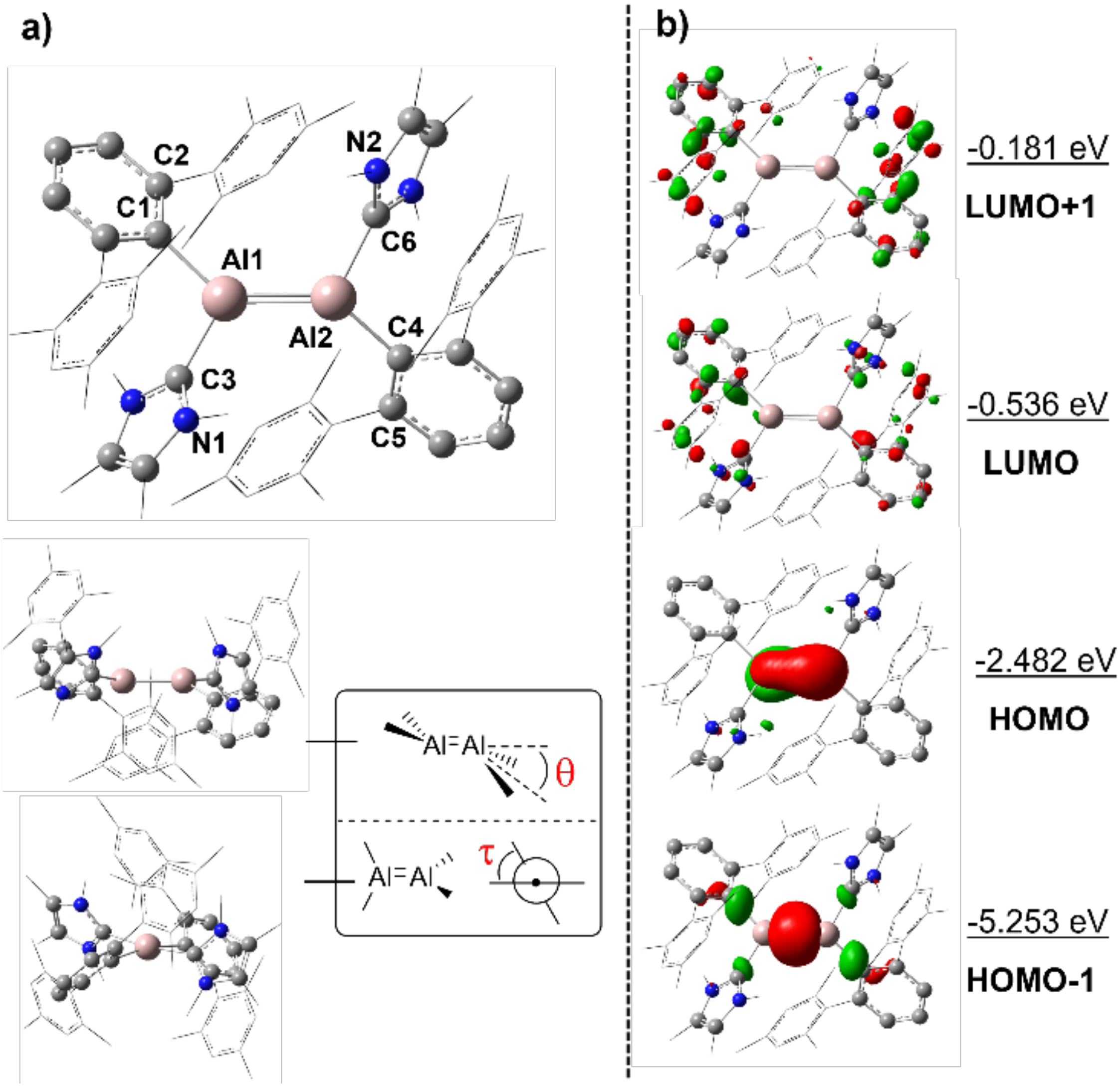

Figure S32. Computed structure (a) and frontier orbitals (±0.05 isovalue) (b) of dialumene **6**. Hydrogens are omitted for clarity. Mesityls of the terphenyl substituent and the methyls of NHC are depicted in wireframe for simplicity. Selected bond lengths [Å] and bond angles [°]: Al1-Al2 2.373, Al1-C1 2.007, Al1-C3 2.034, Al2-C4 2.005, Al2-C6 2.035; C1-Al1-C3 C4-Al2-C6 105.7, C2-C1-Al1-Al2 44.3, N1-C3-Al1-Al2 53.6, C5-C4-Al2-Al1 32.4, N2-C6-Al2-Al1 63.0, θ 9.6, 11.8, τ 7.7.

10) Dialumene **II**

Number of imaginary frequencies = 0

E_{total} = -2732.785665 a.u

$G_{correction}$ = 1.174695 a.u

$BSSE_{correction}$ = 0.002256363 a.u

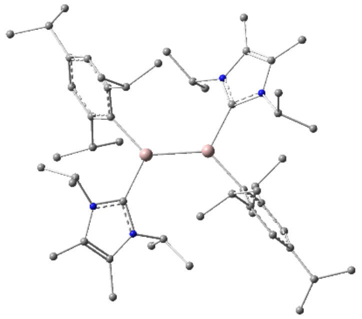

Cartesian coordinates:

Al	-0.92512300	-0.67493200	0.11637600
Al	0.97024400	0.75993000	0.17063100
N	0.23499700	-3.45139100	-0.46746000
N	-1.46873900	-3.57438800	0.85638500
N	-0.24984100	3.50886800	-0.42180000
N	1.47522200	3.66957900	0.87056700
C	-2.87609100	-0.18227000	0.03258600
C	-3.55513500	0.39642600	1.13066200
C	-4.88067600	0.82862600	0.99708800
C	-5.58143400	0.69364800	-0.19984000
C	-4.91744600	0.11167200	-1.28298600
C	-3.58886600	-0.31143700	-1.18671700
C	-2.85707600	0.58760000	2.46745000
C	-2.34636400	2.01580600	2.63728800
C	-3.70519200	0.15330700	3.65970200
C	-7.00901100	1.18218400	-0.32614400
C	-7.11547600	2.33846200	-1.32061800
C	-7.97032100	0.04977700	-0.68428200
C	-2.91774500	-0.92186100	-2.40664200
C	-3.26625600	-0.22303900	-3.71676500
C	-3.18451700	-2.42316500	-2.50056500
C	-0.69253800	-2.69009000	0.17208100
C	0.03578000	-4.79667200	-0.20240500
C	-1.04492100	-4.87693400	0.63622600
C	1.21744200	-2.86373800	-1.38780200
C	2.65527400	-3.22470900	-1.05465700
C	0.83443500	-3.13527000	-2.83457800
C	0.89105100	-5.90300500	-0.70690800
C	-1.65270600	-6.09227900	1.24017500
C	-2.51655500	-3.11837400	1.77465600
C	-3.91927300	-3.43378500	1.28190700
C	-2.24437400	-3.55313400	3.20704000
C	2.91842300	0.25796600	0.09855000
C	3.53250000	-0.45926900	1.15194700
C	4.81463200	-1.00040100	0.98901300
C	5.53216400	-0.84748200	-0.19507100
C	4.94631200	-0.09975000	-1.22110100
C	3.67070000	0.45449600	-1.08719400
C	2.82578600	-0.65315500	2.48317200
C	3.68661900	-0.23245100	3.67195900
C	2.28992200	-2.07175800	2.64978800

C	6.89960400	-1.47526200	-0.36414000
C	6.88411900	-2.55487300	-1.44662000
C	7.98087700	-0.43044100	-0.63569500
C	3.09062700	1.25174400	-2.24613000
C	2.47042900	0.33345900	-3.29698800
C	4.08300600	2.22561600	-2.87545400
C	0.69951000	2.76749400	0.21085300
C	-0.06087700	4.86002900	-0.17850600
C	1.03265300	4.96408500	0.64064600
C	-1.23231500	2.89741200	-1.32743000
C	-2.67056700	3.26993300	-1.00791200
C	-0.84221200	3.12621700	-2.77987800
C	-0.93378600	5.94996900	-0.68857600
C	1.63609600	6.19334000	1.22027300
C	2.57331500	3.23792700	1.73927100
C	3.93784100	3.54237700	1.14397100
C	2.39590900	3.71126900	3.17414000
H	-5.39124200	1.28909300	1.84920300
H	-5.45003100	-0.00210300	-2.23173800
H	-1.94995500	-0.05033500	2.43948200
H	-3.16974200	2.74646600	2.57240000
H	-1.60190100	2.25094500	1.86124600
H	-1.85282500	2.14210200	3.61492500
H	-4.10914200	-0.86164300	3.51960800
H	-4.56228900	0.82564800	3.82611000
H	-3.10496000	0.15837200	4.58334000
H	-7.30272500	1.56782400	0.66568200
H	-8.14367400	2.73225300	-1.36416900
H	-6.83875900	2.01315100	-2.33635400
H	-6.44223600	3.16326600	-1.04177200
H	-9.01147400	0.40896400	-0.71361500
H	-7.90931700	-0.76881800	0.04857200
H	-7.73648600	-0.37250300	-1.67465600
H	-1.82628100	-0.78998300	-2.24784500
H	-4.31116800	-0.40041800	-4.01873000
H	-2.62639800	-0.59432200	-4.53273600
H	-3.12238100	0.86560000	-3.64091400
H	-2.69169600	-2.86001500	-3.38475500
H	-4.26509600	-2.62684900	-2.58151800
H	-2.80590000	-2.94865300	-1.61167500
H	1.09990000	-1.77793800	-1.22028000
H	2.93201600	-4.23642000	-1.38602000
H	2.84547600	-3.13419500	0.02262400
H	3.32551100	-2.51012800	-1.55419400
H	-0.18116200	-2.76421900	-3.03650300
H	0.87221600	-4.20567200	-3.08741900
H	1.52965700	-2.60832200	-3.50343600
H	1.06174800	-5.83983800	-1.79063400
H	0.40970700	-6.86808700	-0.50236200
H	1.87683700	-5.91371400	-0.21670300
H	-1.31848300	-6.98512900	0.69570500
H	-2.75003700	-6.07047000	1.19466100
H	-1.36567500	-6.22520300	2.29539600
H	-2.41253600	-2.02415700	1.74512300
H	-4.13690900	-4.51239100	1.27356300
H	-4.07216700	-3.02973600	0.27206100
H	-4.65220400	-2.94855400	1.94323800

H	-2.41639400	-4.62663700	3.36880100
H	-2.91580100	-3.00591800	3.88434300
H	-1.20883900	-3.31242500	3.49052300
H	5.27418400	-1.56486700	1.80748000
H	5.50182300	0.05020300	-2.15182700
H	1.93396000	0.00565800	2.45882000
H	4.55239600	-0.89934100	3.81400200
H	3.09941800	-0.25563300	4.60387000
H	4.07851900	0.78860000	3.54037100
H	1.54369400	-2.29209000	1.87099400
H	1.79254100	-2.19113300	3.62651700
H	3.09794800	-2.81953700	2.58591200
H	7.14454900	-1.96739200	0.59321500
H	6.64638200	-2.12285100	-2.43204400
H	6.12576800	-3.32237700	-1.22933400
H	7.86429600	-3.05182900	-1.52708800
H	7.80083500	0.09099600	-1.58934500
H	8.97659900	-0.89823300	-0.69574100
H	8.00554400	0.33020000	0.15905900
H	2.26345100	1.85236500	-1.82306100
H	1.63562000	-0.23421900	-2.85625000
H	3.21682200	-0.37502900	-3.69283600
H	2.06822600	0.91334200	-4.14419100
H	3.57448600	2.87356400	-3.60696100
H	4.89025400	1.70350300	-3.41364300
H	4.55418500	2.87088500	-2.11803500
H	-1.11716700	1.81619300	-1.13454300
H	-3.34132700	2.54290300	-1.48862500
H	-2.94515400	4.27266500	-1.36637700
H	-2.86247100	3.20925200	0.07138500
H	-1.52908200	2.57360900	-3.43700800
H	0.17494100	2.74915800	-2.96415200
H	-0.88272700	4.18818500	-3.06656500
H	-1.91307100	5.96071300	-0.18550900
H	-1.11821000	5.86633000	-1.76881200
H	-0.45822600	6.92262300	-0.50746900
H	1.36812100	6.33280900	2.27973900
H	1.27953000	7.07634200	0.67387600
H	2.73277700	6.18546200	1.15368700
H	2.47119900	2.14208400	1.74627200
H	4.01413200	3.11615500	0.13427700
H	4.71885200	3.07165100	1.75914900
H	4.14977300	4.62122300	1.09499500
H	3.10564400	3.17413600	3.82008500
H	1.37933000	3.48730400	3.53027100
H	2.58662100	4.78645200	3.29888800

11) Alumylene **7** (singlet ground state)

Note: The ground state for alumylene **7** is singlet ($\Delta G_{singlet-triplet}$ = 17.0 kcal/mol).

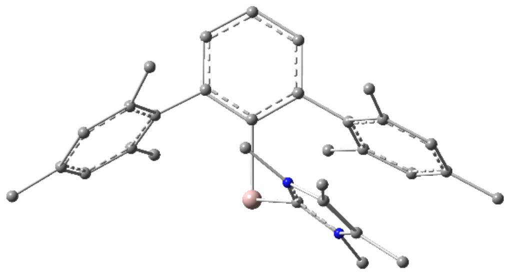

Hydrogen atoms are omitted for clarity.

Number of imaginary frequencies = 0

E_{total} = -1552.947063 a.u

$G_{correction}$ = 0.532312 a.u

Cartesian coordinates:

C	0.72120600	-1.07724100	0.31590700
C	-0.31976700	-1.79502100	0.94954500
C	-0.07187100	-2.63572500	2.04093300
H	-0.90482700	-3.17317500	2.50465100
C	1.22250800	-2.79326800	2.52979000
H	1.41547500	-3.45379800	3.37890000
C	2.26891500	-2.10760700	1.91987900
H	3.29231600	-2.22816300	2.28742800
C	2.02820100	-1.26379400	0.82800900
C	3.18127500	-0.55442200	0.19529900
C	3.68352500	-1.01140100	-1.04539300
C	4.72121100	-0.30992300	-1.65854300
H	5.09952700	-0.66733600	-2.62115600
C	5.29463700	0.82493300	-1.07799800
C	4.82727000	1.22305500	0.17295400
H	5.28764500	2.08602200	0.66501300
C	3.79418200	0.54342300	0.82878800
C	3.41732200	0.97163300	2.22174400
H	2.41741100	0.62448700	2.51188500
H	3.46331600	2.06621100	2.33013600
H	4.12098700	0.54730300	2.95745900
C	6.37828700	1.58542200	-1.78625400
H	7.12799500	0.90676900	-2.22112900
H	6.89738900	2.27726900	-1.10745100
H	5.96403000	2.18158600	-2.61633100
C	3.13356700	-2.24905100	-1.69126000
H	3.80745600	-2.61324800	-2.47932400
H	2.15313900	-2.04346600	-2.15905300
H	2.97798500	-3.05151900	-0.95555500
C	-1.72610200	-1.68805400	0.45524400
C	-2.16037600	-2.50351500	-0.61145600
C	-3.49604900	-2.43766400	-1.01846500
H	-3.82840200	-3.08093900	-1.83897300
C	-4.41745600	-1.58970400	-0.39856300
C	-3.96609900	-0.78736000	0.65173000
H	-4.67090900	-0.11780500	1.15551300
C	-2.63876400	-0.82538400	1.09102000
C	-2.21103000	0.01777000	2.25764500
H	-2.23095500	-0.56520100	3.19326700

H	-2.87592200	0.88445100	2.38607500
H	-1.18009700	0.37318600	2.13236300
C	-5.84079600	-1.52106700	-0.87354000
H	-5.93083100	-0.89854700	-1.78056900
H	-6.50139700	-1.08638800	-0.10955400
H	-6.22735200	-2.51824300	-1.13171700
C	-1.21625100	-3.47121700	-1.26726600
H	-1.72744600	-4.06100900	-2.04056700
H	-0.78325300	-4.16151200	-0.52716600
H	-0.37006000	-2.94825300	-1.74119900
Al	0.34087500	-0.06790000	-1.45297600
C	-0.90485400	1.43516700	-0.60707000
N	-0.63868200	2.37714100	0.32603300
C	-1.66425800	3.30355800	0.42704000
C	-2.61076200	2.92003200	-0.48373500
N	-2.10802000	1.79124400	-1.11003100
C	-2.78213300	1.05260900	-2.15169000
H	-2.02177800	0.47558700	-2.70461700
H	-3.28133000	1.74407300	-2.84360400
H	-3.51232000	0.35215600	-1.72471600
C	-3.93958900	3.49511400	-0.80710900
H	-3.99121500	3.86102100	-1.84531900
H	-4.16088900	4.34222000	-0.14519900
H	-4.74003800	2.74884600	-0.67837800
C	-1.63424300	4.43519100	1.38620900
H	-2.53346800	5.05483100	1.27584200
H	-0.75917400	5.08466400	1.22391500
H	-1.59659000	4.08500200	2.43083600
C	0.57943800	2.39853100	1.09518100
H	1.27073400	3.17482300	0.73296700
H	1.05713000	1.41701200	0.99243200
H	0.36439000	2.57496100	2.15810900

12) Alumylene **7** (triplet ground state)

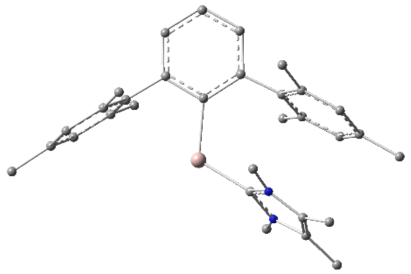

Hydrogen atoms are omitted for clarity.

Number of imaginary frequencies = 0

E_{total} = -1552.923643 a.u

$G_{correction}$ = 0.533081 a.u

Cartesian coordinates:

C	1.08748200	-0.99907300	-0.15172300
C	0.13926900	-2.04539200	-0.30253000
C	0.55306000	-3.37727300	-0.42280100

H	-0.20366300	-4.15797900	-0.54694200
C	1.90309400	-3.71623100	-0.39621500
H	2.21324800	-4.75917800	-0.49630300
C	2.85318500	-2.70701900	-0.24387400
H	3.91927400	-2.95143500	-0.21936700
C	2.45770900	-1.37356100	-0.12650800
C	3.49479400	-0.31059700	0.01680500
C	4.05747600	0.28138500	-1.13086100
C	5.01039700	1.29077900	-0.97566400
H	5.44243300	1.75075200	-1.86986100
C	5.42305600	1.72736700	0.28472400
C	4.86392800	1.11585800	1.40900000
H	5.18036400	1.43660500	2.40642500
C	3.90754100	0.10478200	1.29789000
C	3.32210900	-0.53243500	2.52437700
H	2.22827100	-0.39604900	2.54492500
H	3.74439600	-0.09703400	3.44084600
H	3.50204600	-1.61848900	2.53727900
C	6.41705400	2.84389500	0.42794500
H	7.11016000	2.88005300	-0.42540900
H	7.01121900	2.74063000	1.34791900
H	5.90734800	3.82106900	0.47650300
C	3.63495000	-0.16762800	-2.49999100
H	4.13273500	0.42131900	-3.28302200
H	2.54490500	-0.06195900	-2.62435300
H	3.86790500	-1.23130400	-2.66317900
C	-1.32435800	-1.76122600	-0.32317500
C	-1.93100800	-1.21320800	-1.47343500
C	-3.29366000	-0.91439300	-1.44955400
H	-3.75576200	-0.48600600	-2.34542500
C	-4.08676000	-1.16424100	-0.32539100
C	-3.48024100	-1.75712000	0.78159100
H	-4.08787400	-1.99330900	1.66176000
C	-2.11415700	-2.06511700	0.79998400
C	-1.50711400	-2.71720700	2.01093400
H	-1.28287500	-3.77883800	1.82203400
H	-2.18836300	-2.67136500	2.87355200
H	-0.54993800	-2.24884000	2.28416300
C	-5.54199200	-0.79612100	-0.31855000
H	-6.05430700	-1.15622000	-1.22417200
H	-5.67287900	0.29890100	-0.28923200
H	-6.06265100	-1.21660400	0.55379900
C	-1.12302300	-0.97812000	-2.71438000
H	-1.74864100	-0.60096100	-3.53593900
H	-0.63317800	-1.90701300	-3.04458800
H	-0.30894500	-0.25511900	-2.52744900
Al	0.72707700	0.96437000	-0.11632800
C	-1.21852400	1.50755700	0.14496700
N	-1.98871600	1.33570700	1.24807300
C	-3.25332600	1.86803200	1.07431500
C	-3.27179900	2.41123400	-0.17981900
N	-2.01764800	2.18315900	-0.72013300
C	-1.63680700	2.56870500	-2.05604100
H	-0.61716900	2.19324000	-2.23728300
H	-1.63848400	3.66312100	-2.16575000
H	-2.32233500	2.12749600	-2.79446400
C	-4.35817600	3.08540000	-0.93145900

H	-4.06201500	4.09118100	-1.26948800
H	-5.24873700	3.19517300	-0.29931500
H	-4.65135900	2.50925600	-1.82452500
C	-4.31018500	1.76505500	2.10834600
H	-5.22867400	2.26211700	1.77099200
H	-4.00609000	2.23138600	3.05877000
H	-4.55312100	0.71009000	2.31636900
C	-1.53021000	0.74366000	2.47971200
H	-1.60076000	1.47212400	3.30190500
H	-0.47659900	0.45589300	2.34887400
H	-2.11992800	-0.14805000	2.72492200

13) Alumylene (singlet ground state) derived from dialumene **II**

Note: The ground state for the alumylene from **II** is singlet ($\Delta G_{singlet\text{-}triplet}$ = 15.8 kcal/mol).

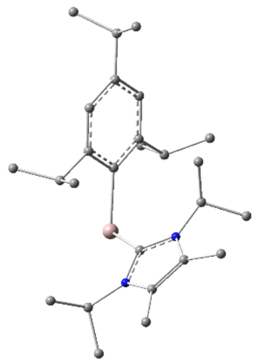

Hydrogen atoms are omitted for clarity.

Number of imaginary frequencies = 0

E_{total} = -1366.347523 a.u

$G_{correction}$ = 0.574632 a.u

Cartesian coordinates:

Al	-0.62956400	0.25946700	2.17707700
N	-3.07555500	-0.21092300	0.26996900
N	-1.72440400	1.13098500	-0.72790500
C	1.08690600	-0.06397500	1.07675300
C	2.15732900	0.85527900	1.07342600
C	3.32360800	0.59233400	0.34630200
C	3.47556700	-0.58056000	-0.39516600
C	2.42840800	-1.50539600	-0.37334300
C	1.25744100	-1.26095000	0.35008500
C	2.06043400	2.12813400	1.90124300
C	2.50236600	3.38519400	1.15799300
C	2.81322200	1.96583300	3.22086800
C	4.73015700	-0.83258900	-1.20500800
C	5.46000800	-2.09612000	-0.75178000
C	4.42776200	-0.87166300	-2.70315300
C	0.15323700	-2.30490200	0.33939600
C	0.61149800	-3.63602900	0.93065600
C	-0.44972200	-2.48457600	-1.05244300

C	-1.87263100	0.39831600	0.39977200
C	-3.67444300	0.11635600	-0.93888200
C	-2.81405700	0.97376000	-1.57361100
C	-3.62585200	-1.04377700	1.34663500
C	-4.93042500	-0.48236000	1.88872600
C	-3.69906600	-2.51778300	0.98210600
C	-4.99930500	-0.37059400	-1.40631800
C	-2.99020400	1.64406000	-2.88934200
C	-0.54077700	1.97242400	-0.94601400
C	0.37798700	1.42001400	-2.02391000
C	-0.90669300	3.43739300	-1.13351400
H	4.14452200	1.31713700	0.34690700
H	2.52886800	-2.43723700	-0.94030000
H	0.99026300	2.26069500	2.16318800
H	3.58257000	3.37053300	0.94339500
H	2.30245000	4.28579300	1.75963100
H	1.97848000	3.49395500	0.19566900
H	2.42499500	1.10441700	3.78471600
H	2.71313700	2.86370900	3.85176000
H	3.88592300	1.79340200	3.03641000
H	5.40311900	0.02355500	-1.02393400
H	4.84364800	-2.99389000	-0.91902500
H	5.69864600	-2.05292700	0.32135700
H	6.40076300	-2.23065700	-1.30921700
H	3.75987300	-1.71274000	-2.94979600
H	5.35073600	-0.99437200	-3.29237500
H	3.93066300	0.05389300	-3.03137400
H	-0.65084500	-1.92554900	1.00276900
H	1.02034100	-3.49575600	1.94214500
H	1.39853300	-4.09620900	0.31230300
H	-0.22445400	-4.35172100	0.99204100
H	-1.25796300	-3.23394800	-1.05003900
H	0.31405400	-2.82933900	-1.76744200
H	-0.86228800	-1.53865800	-1.43447200
H	-2.87426400	-0.93378100	2.15324400
H	-5.19757100	-1.01663300	2.81203500
H	-5.76887500	-0.59646300	1.18661400
H	-4.81687700	0.58283800	2.13729600
H	-3.94520400	-3.10137900	1.88144000
H	-2.73464400	-2.87710200	0.59939400
H	-4.47126200	-2.73225200	0.22854800
H	-5.83398500	0.12734300	-0.88809000
H	-5.11714100	-1.45312800	-1.26307400
H	-5.11311400	-0.16644300	-2.47891800
H	-3.27406900	2.70270800	-2.78173400
H	-3.78948300	1.14773000	-3.45514400
H	-2.07906600	1.60389900	-3.50131200
H	0.00954700	1.88065300	-0.00118500
H	0.66674500	0.38678300	-1.78635500
H	1.30069900	2.01763500	-2.05140900
H	-0.07355700	1.45054200	-3.02669600
H	0.00389500	4.04917300	-1.05810400
H	-1.60385500	3.77132100	-0.35091900
H	-1.35644600	3.64204000	-2.11527400

14) Alumylene (triplet ground state) derived from dialumene **II**

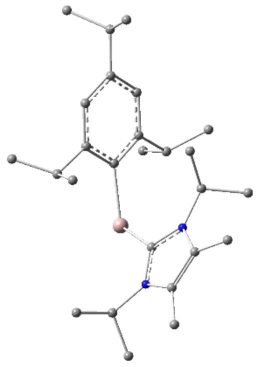

Hydrogen atoms are omitted for clarity.

Number of imaginary frequencies = 0

E_{total} = -1366.321338 a.u

$G_{correction}$ = 0.571827 a.u

Cartesian coordinates:

Al	-0.68845200	-0.05086300	1.84404800
N	-3.30123500	-0.18049900	0.31737500
N	-1.83318000	0.98477400	-0.77809300
C	1.17320200	-0.19376600	1.08287200
C	2.11713500	0.85604500	1.17114900
C	3.32686300	0.77763400	0.47418600
C	3.64659500	-0.32379700	-0.32075900
C	2.73303200	-1.37728500	-0.37378800
C	1.52071800	-1.33724200	0.32258000
C	1.84179200	2.05738900	2.05754200
C	2.29668900	3.38870000	1.47017200
C	2.42623400	1.83334000	3.45182000
C	4.94193700	-0.36722100	-1.10364100
C	5.81847500	-1.55036300	-0.69583400
C	4.67933500	-0.36129500	-2.60975100
C	0.58976800	-2.53570300	0.22574900
C	1.29576300	-3.85394100	0.53092800
C	-0.14370400	-2.58949200	-1.11263000
C	-2.01644100	0.28440200	0.38488700
C	-3.90244700	0.20129400	-0.87317000
C	-2.97500400	0.93121300	-1.56684000
C	-3.83399100	-1.07426500	1.34389000
C	-5.09646000	-0.53744300	1.99876500
C	-3.95316900	-2.50425300	0.83585200
C	-5.30746600	-0.09807800	-1.25713800
C	-3.12428300	1.57552700	-2.89893600
C	-0.62156500	1.77319500	-1.01555600
C	0.27936500	1.18386700	-2.08917900
C	-0.92693400	3.25106800	-1.21684000
H	4.04830700	1.59753300	0.54173900
H	2.97188100	-2.25800800	-0.97802600
H	0.73872900	2.10467900	2.17649800
H	3.39465400	3.46368700	1.41843100
H	1.94532500	4.22524000	2.09409600
H	1.90557500	3.53606700	0.45141500
H	2.03045900	0.90770100	3.89630400
H	2.18166300	2.67058800	4.12528100

H	3.52352400	1.74017900	3.40451700
H	5.49375300	0.55676700	-0.85811900
H	5.32844000	-2.50899400	-0.92941800
H	6.02493200	-1.53777200	0.38475600
H	6.78124700	-1.53049700	-1.23092500
H	4.13208800	-1.26624100	-2.91906700
H	5.62330800	-0.33096300	-3.17712600
H	4.07344900	0.50935500	-2.90347300
H	-0.18079300	-2.38932700	1.01026600
H	1.81887800	-3.80793000	1.49780700
H	2.03967500	-4.11109400	-0.24032600
H	0.56990900	-4.68157400	0.57252600
H	-0.81287500	-3.46445300	-1.16073900
H	0.56483900	-2.66345800	-1.95410100
H	-0.75652100	-1.68798600	-1.26368000
H	-3.03850100	-1.07797000	2.11224500
H	-5.32655300	-1.13422100	2.89383100
H	-5.97233900	-0.58862400	1.33600000
H	-4.95489000	0.50665400	2.31359600
H	-4.20966100	-3.17584800	1.66832400
H	-2.99303700	-2.83505200	0.41291400
H	-4.73063000	-2.61573400	0.06587200
H	-6.02999000	0.48521700	-0.66435600
H	-5.56202100	-1.16055600	-1.13697600
H	-5.46707100	0.16107700	-2.31168400
H	-3.30041900	2.66057000	-2.82643200
H	-3.98030900	1.13981000	-3.43077800
H	-2.23567700	1.42504000	-3.52722700
H	-0.07704700	1.68466700	-0.06409600
H	0.52671500	0.13993600	-1.85675700
H	1.22568300	1.74413900	-2.11522400
H	-0.17011000	1.23173500	-3.09266300
H	0.00962600	3.82368900	-1.14979600
H	-1.60495900	3.61877400	-0.43248300
H	-1.37110800	3.46730800	-2.19851700

2.5. Frontier orbitals of alumylene 7 and its base-free version

15) Alumylene **7** (singlet ground state)

Note: Energies of **7** are already given in Section 2.3.

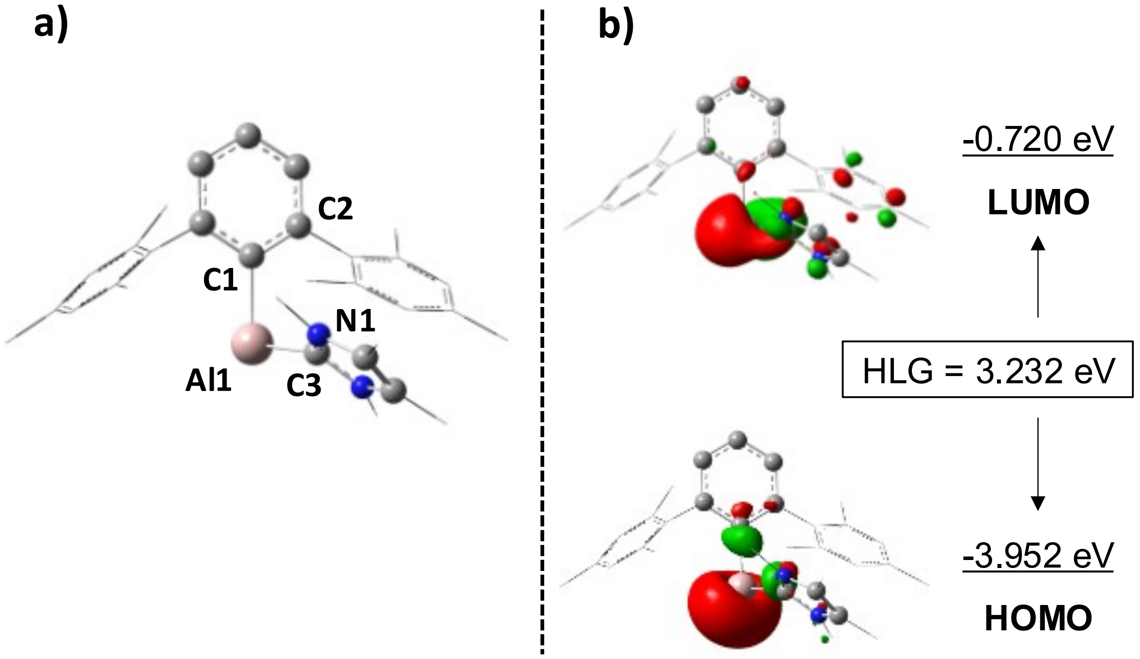

Figure S33. Computed structure (a) and frontier orbitals (±0.05 isovalue) (b) of alumylene **7**. HLG: HOMO-LUMO gap. Hydrogens are omitted for clarity. Mesityl groups of the terphenyl substituent and the methyl groups of the NHC are depicted in wireframe style for simplicity. Selected bond lengths [Å] and bond angles [°]: Al1-C1 2.072, Al1-C3 2.128, C1-Al1-C3 96.4, C2-C1-Al1-C3 69.2, N1-C3-Al1-C1 61.6.

16) Base-free alumylene (singlet ground state) derived from alumylene **7**

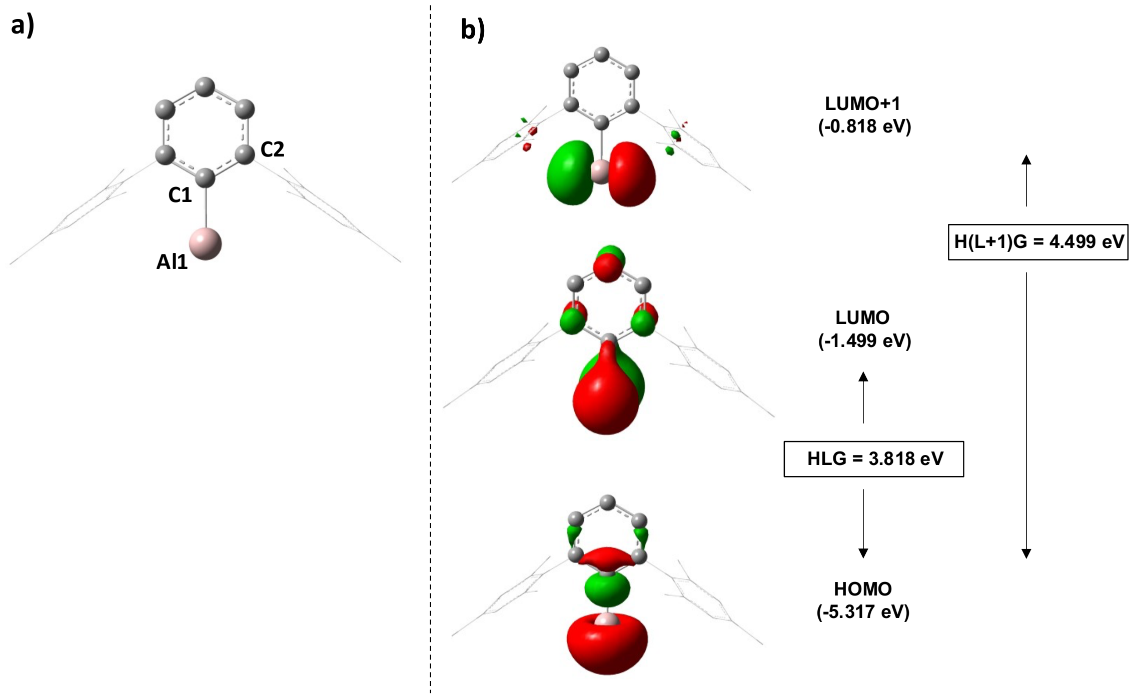

Figure S34. Computed structure (a) and frontier orbitals (±0.05 isovalue) (b) of base-free alumylene derived from alumylene **7**. Hydrogens are omitted for clarity. Mesityls of the terphenyl substituent are depicted in wireframe style for simplicity. Selected bond lengths [Å] and bond angles [°]: Al1-C1 2.029, C1-C2 1.407.

Number of imaginary frequencies = 0

E_{total} = -1170.163693 a.u

$G_{correction}$ = 0.355950 a.u

Cartesian coordinates:

C	-0.00002800	0.47970200	0.01734100
C	-1.21169700	1.19500900	0.04089400
C	-1.21153500	2.59220800	0.08707400
H	-2.15882400	3.13890800	0.10516900
C	0.00010200	3.28405900	0.10997400
H	0.00015600	4.37658700	0.14619300
C	1.21166100	2.59208900	0.08696400
H	2.15900700	3.13869400	0.10496200
C	1.21170600	1.19487800	0.04078600
C	2.47141900	0.39416900	0.01356000
C	3.04286400	-0.05846000	1.21939600
C	4.18069800	-0.86719200	1.16869300
H	4.62245900	-1.21591700	2.10703100
C	4.76609000	-1.24207000	-0.04256500
C	4.18576000	-0.77798800	-1.22495500
H	4.63138800	-1.05618900	-2.18476800
C	3.04804400	0.03256500	-1.22010900
C	2.44585700	0.50864200	-2.51142300
H	1.41533200	0.13827500	-2.63570300
H	3.03626200	0.17094300	-3.37409900

H	2.37919500	1.60681500	-2.54257000
C	5.96399700	-2.14621600	-0.07338500
H	6.59289200	-2.01401100	0.81900900
H	6.58547400	-1.96329800	-0.96193800
H	5.65537100	-3.20473800	-0.10202500
C	2.43542100	0.32056200	2.54008600
H	3.02668100	-0.07386300	3.37774200
H	1.40726300	-0.06468700	2.63595300
H	2.36108300	1.41324800	2.64900100
C	-2.47141700	0.39430000	0.01376500
C	-3.04255100	-0.05864100	1.21957400
C	-4.18036800	-0.86749700	1.16890900
H	-4.62190500	-1.21649000	2.10724500
C	-4.76595600	-1.24212900	-0.04226500
C	-4.18590300	-0.77769000	-1.22471700
H	-4.63169700	-1.05570400	-2.18451400
C	-3.04830600	0.03293300	-1.21991700
C	-2.44639500	0.50941100	-2.51120900
H	-2.38018400	1.60761700	-2.54221700
H	-3.03672300	0.17159400	-3.37389200
H	-1.41572300	0.13949500	-2.63558000
C	-5.96377500	-2.14638000	-0.07342700
H	-5.65503900	-3.20442500	-0.11537500
H	-6.59183900	-1.95464200	-0.95551000
H	-6.58611800	-2.02352400	0.82483300
C	-2.43495200	0.32017900	2.54025100
H	-3.02598100	-0.07458000	3.37791200
H	-2.36084700	1.41285800	2.64941000
H	-1.40668600	-0.06484200	2.63585800
Al	-0.00041500	-1.54821400	-0.05168400

2.6. Mechanisms explored for the formation of benzene deconstruction product and C-H activation product using model alumylene 7’

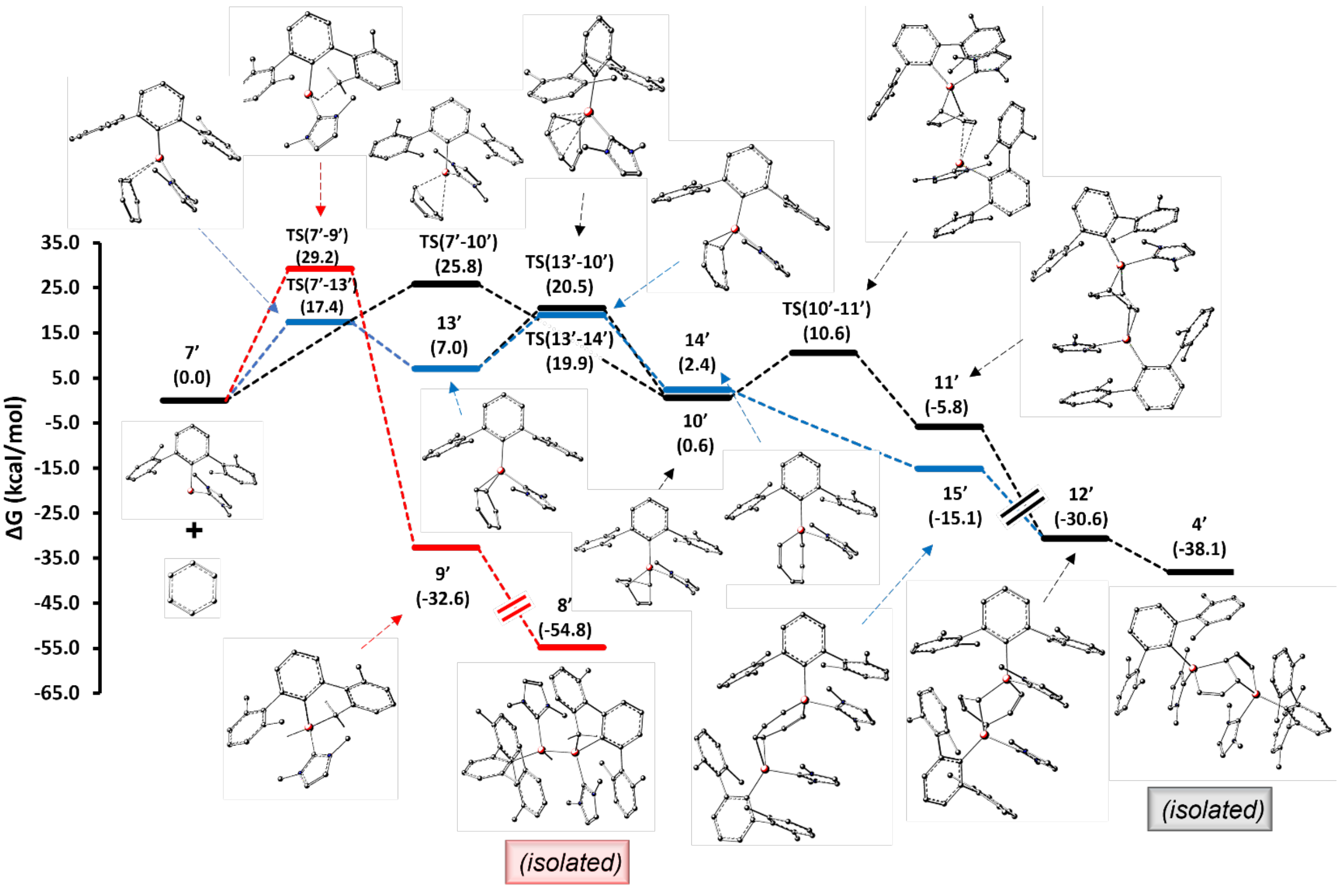

Figure S35. Mechanisms computed for the formation of benzene deconstruction product **4’** (black and blue pathways) and the C-H activation product **8’** (red pathway) from alumylene **7’**. Free energies in parentheses are in kcal/mol.

17) Alumylene (**7‘**)

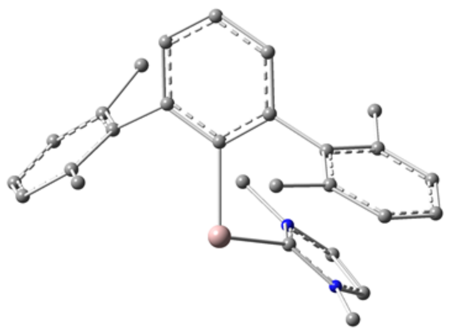

(Hydrogens are omitted for clarity)

Number of imaginary frequencies = 0

E_{total} = -1395.961237 a.u

$G_{correction}$ = 0.431441 a.u

Cartesian coordinates:

C	-0.46925500	-0.87791800	0.12540300
C	-1.78972000	-1.20034500	0.52213600
C	-2.04390800	-2.22383800	1.44327300
H	-3.07744000	-2.44502200	1.72579700
C	-0.99738900	-2.95815800	1.99360400
H	-1.20112000	-3.76080600	2.70676200
C	0.31187800	-2.66083800	1.62418600
H	1.14632500	-3.22773200	2.04845900
C	0.57230900	-1.63896600	0.70400600
C	1.99646000	-1.37270600	0.33738500
C	2.56965400	-2.02601300	-0.77344100
C	3.92626000	-1.82139300	-1.05447900
H	4.37510700	-2.33452400	-1.90910900
C	4.70541700	-0.99417600	-0.25194200
C	4.13021600	-0.34192700	0.83661400
H	4.73960300	0.31111700	1.46758800
C	2.77783000	-0.51992100	1.14448800
C	2.17557200	0.15470300	2.34230800
H	1.97865900	-0.57132800	3.14732400
H	1.20770100	0.61089300	2.09272500
H	2.84319500	0.93285300	2.73874500
C	1.75540100	-2.97428500	-1.60740900
H	2.34054700	-3.35852600	-2.45412200
H	0.85252400	-2.48695500	-2.00740300
H	1.41020400	-3.82946300	-1.00550500
C	-2.94077900	-0.42488000	-0.03161700
C	-3.38181500	-0.66961700	-1.35126100
C	-4.41035600	0.11726800	-1.87796000
H	-4.74502400	-0.06478000	-2.90264400
C	-5.01371100	1.11330900	-1.11691200
C	-4.61798400	1.30832200	0.20166200
H	-5.11499100	2.06574400	0.81485600
C	-3.59650500	0.53641100	0.76610300
C	-3.26552600	0.70914700	2.22391700
H	-3.90249600	0.05606900	2.84368800
H	-3.44620800	1.74306900	2.55490300
H	-2.22823900	0.43291700	2.45517100
C	-2.78449500	-1.77403700	-2.17317300
H	-3.41502500	-1.99797700	-3.04488500
H	-2.65823300	-2.69149700	-1.57992100
H	-1.78423400	-1.49111300	-2.54948500
Al	-0.02183500	0.34202100	-1.47889000
C	1.02016000	1.87351200	-0.45323000
N	0.54208700	2.77624900	0.43909000
C	1.39647200	3.84724200	0.57537300
C	2.45009000	3.60507500	-0.25073800
N	2.19414400	2.40530100	-0.87362600
C	3.04314500	1.77202100	-1.85675100
H	3.69061600	1.01904200	-1.38823800
H	3.65338500	2.53379500	-2.35917800
H	2.39561500	1.27055400	-2.59723300
C	-0.72935900	2.62689400	1.10360500
H	-0.66324400	2.99556300	2.13578100
H	-0.97911400	1.55898000	1.11689500

H	-1.52630600	3.16697000	0.56985700
H	5.76601700	-0.85459600	-0.47644600
H	-5.81013800	1.72548400	-1.54727300
H	3.35070800	4.18264300	-0.44061200
H	1.19148000	4.68218200	1.23991200

18) Benzene

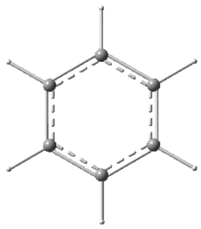

Number of imaginary frequencies = 0

E_{total} = -231.811400 a.u

$G_{correction}$ = 0.073809 a.u

Cartesian coordinates:

C	1.09521100	0.86353400	0.00000000
C	-0.20030100	1.38010300	0.00000700
C	-1.29550300	0.51665300	-0.00000900
C	-1.09519800	-0.86355100	0.00000100
C	0.20027900	-1.38010600	0.00000700
C	1.29551100	-0.51663300	-0.00000400
H	1.95357400	1.54047900	-0.00000300
H	-0.35721900	2.46197700	0.00000400
H	-2.31086700	0.92172500	-0.00001200
H	-1.95360200	-1.54044300	0.00000000
H	0.35726200	-2.46197100	0.00000600
H	2.31085100	-0.92176700	-0.00000400

19) TS[7‘-10‘]

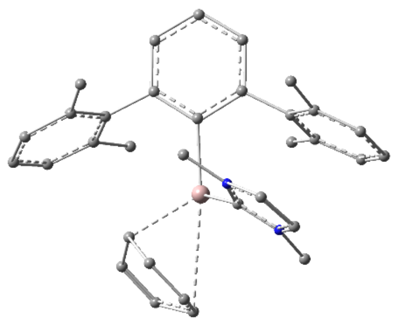

(Hydrogens are omitted for clarity)

Number of imaginary frequencies = 1 (140.5 cm^{-1})

E_{total} = -1627.762082 a.u

$G_{correction}$ = 0.528362 a.u

Cartesian coordinates:

C	1.55623500	3.63400500	-1.79835700
C	0.29420900	3.92706600	-1.19124800
C	0.17332200	3.78422400	0.20540000
C	1.03172900	2.92012900	0.86188200
C	1.90872000	2.12059100	0.03646200
C	2.39641900	2.77048700	-1.16510000
H	1.80853200	4.07381800	-2.76663900
H	-0.42719800	4.54668400	-1.73057800
H	-0.61266100	4.31675600	0.74971800
H	0.98951500	2.77857000	1.94449400
H	3.35922600	2.47559900	-1.58665600
H	2.60738300	1.45602700	0.55570600
Al	0.15071900	0.78484700	-0.53782400
C	0.32185800	-1.25221200	-0.24589800
C	-1.33769000	1.19328400	0.88893400
C	-0.81361400	-2.08170900	-0.44196800
C	1.56347400	-1.92060100	-0.09685500
N	-1.36948600	0.82848700	2.19188000
N	-2.50388900	1.84303600	0.68761100
C	-0.71276100	-3.47843700	-0.45285300
C	-2.17667600	-1.51172500	-0.67377100
C	1.65333800	-3.31862000	-0.10366000
C	2.84278800	-1.16389500	0.05005200
C	-2.53397900	1.24390500	2.79789800
C	-3.25569300	1.88183900	1.83921600
H	-1.61491900	-4.07763200	-0.60926700
C	0.51815400	-4.10279700	-0.27807800
C	-3.12976500	-1.52506500	0.36507300
C	-2.53453000	-1.04585000	-1.95795700
H	2.63291200	-3.78970400	0.01982900
C	3.46140900	-1.04592300	1.31022400
C	3.45358200	-0.60699000	-1.09513000
H	0.59347000	-5.19298200	-0.29054300
C	-4.41256400	-1.02230800	0.12365100
C	-3.83292400	-0.56957000	-2.16812300
C	4.66139300	-0.33254600	1.41739000
C	4.66566800	0.07317300	-0.95336100
H	-5.14640100	-1.02211000	0.93455900
C	-4.76623100	-0.54814500	-1.13575200
H	-4.11355900	-0.21794000	-3.16471400
H	5.13196100	-0.22374400	2.39875100
C	5.26322800	0.22353500	0.29472300
H	5.14320900	0.49794200	-1.84059000
H	6.20307800	0.77247800	0.39046800
H	-5.77739300	-0.17455700	-1.31788600
H	-2.75397400	1.04741000	3.84354700
H	-4.23630200	2.34740000	1.87802100
C	-1.55605100	-1.08673500	-3.09601200
H	-2.05917500	-0.90291900	-4.05541100
H	-0.77501300	-0.31418700	-2.97116300
H	-1.03855200	-2.05604600	-3.14504600
C	-2.80276800	-2.11383500	1.70794500
H	-3.43321800	-1.67773900	2.49661700
H	-2.97600800	-3.20279800	1.70914000
H	-1.74642800	-1.97084800	1.96796900

C	2.80291500	-0.70530700	-2.44225900
H	3.51529400	-0.46985600	-3.24511300
H	2.38024100	-1.70461100	-2.62022200
H	1.96288200	0.01214200	-2.51748000
C	2.88197500	-1.69054300	2.53950200
H	2.99861900	-1.04147400	3.42091100
H	1.82025200	-1.93784400	2.41468700
H	3.40017200	-2.63747700	2.76529300
C	-2.91912900	2.44310700	-0.56187700
H	-2.12708400	2.26243200	-1.30333800
H	-3.85950700	1.98860400	-0.89834300
H	-3.04082500	3.52832600	-0.43351500
C	-0.27690200	0.17501500	2.87310600
H	0.30465100	-0.38793700	2.13222200
H	0.37525000	0.91164400	3.36592100
H	-0.66899100	-0.52239700	3.62473300

20) Intermediate **10‘**

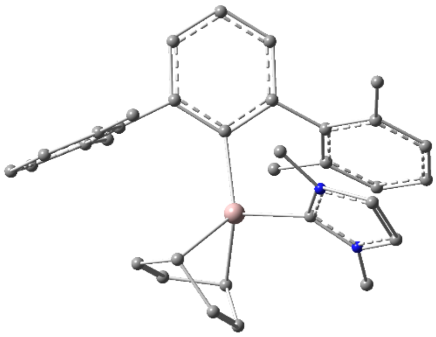

(Hydrogens are omitted for clarity)

Number of imaginary frequencies = 0

E_{total} = -1627.805958 a.u

$G_{correction}$ = 0.531337 a.u

Cartesian coordinates:

C	-1.54456900	-1.78596600	-2.25492000
C	-0.04503400	-1.90382600	-2.10349800
C	0.27975100	-3.24566500	-1.50112100
C	-0.40134300	-3.51886100	-0.36656100
C	-1.36491400	-2.44272800	0.04700100
C	-2.23075400	-2.07212700	-1.12745100
H	-2.01925300	-1.52386900	-3.20514400
H	0.52610100	-1.65010000	-3.00543200
H	0.97467200	-3.94895600	-1.97131100
H	-0.30093400	-4.45772400	0.18755800
H	-3.32019000	-2.04669700	-1.05365300
H	-1.91321900	-2.63511700	0.97588500
Al	-0.07726500	-0.89276800	-0.31931000
C	-0.47967700	1.05111000	0.08287900
C	1.66253400	-1.30133100	0.77080700
C	0.57964600	1.99447500	0.09259300

C	-1.75143300	1.52490300	0.48991300
N	1.76055100	-1.23401700	2.11707600
N	2.85340000	-1.78621600	0.36506000
C	0.39247800	3.30754300	0.54305700
C	1.93112700	1.65494000	-0.44521800
C	-1.92136900	2.83841400	0.95340400
C	-3.00982100	0.72736100	0.34763000
C	2.99066400	-1.67303300	2.55089500
C	3.68562900	-2.01928400	1.43508300
H	1.23322000	4.00657400	0.52161600
C	-0.85277600	3.72608500	0.99728400
C	3.05090500	1.59342800	0.40971100
C	2.09254800	1.48163100	-1.83897300
H	-2.91901100	3.16450800	1.26081200
C	-3.55677900	-0.00390500	1.41574400
C	-3.70287000	0.81752000	-0.87885300
H	-0.99654600	4.74820600	1.35669300
C	4.31048200	1.31584600	-0.13221200
C	3.37037700	1.22091300	-2.34508300
C	-4.78232600	-0.65702300	1.23732500
C	-4.93096100	0.16848900	-1.01646000
H	5.17589800	1.25801200	0.53411300
C	4.47308600	1.13191000	-1.50105200
H	3.49884600	1.10140400	-3.42428500
H	-5.20508700	-1.23077300	2.06679900
C	-5.46916600	-0.57145000	0.03267300
H	-5.46679400	0.23965100	-1.96670200
H	-6.42930300	-1.07893600	-0.08879400
H	5.46499200	0.93156800	-1.91463600
H	3.26924800	-1.69364200	3.60076700
H	4.69546700	-2.40135500	1.31528800
C	0.93394100	1.63211200	-2.78396200
H	1.28789100	1.72452000	-3.82018000
H	0.25596500	0.76616800	-2.73653500
H	0.33111600	2.51804600	-2.53637900
C	2.93098500	1.85303400	1.88545900
H	3.66707500	1.26165600	2.44963500
H	3.12104700	2.91502200	2.11313700
H	1.92519200	1.62915700	2.26217200
C	-3.12016200	1.58104700	-2.03104700
H	-3.81228600	1.59826600	-2.88430700
H	-2.87866200	2.61860000	-1.75451700
H	-2.17963200	1.11258700	-2.36002300
C	-2.84686500	-0.12079300	2.73325500
H	-2.17504000	-0.99409400	2.72738600
H	-2.23849500	0.76853200	2.95013900
H	-3.56099700	-0.26220200	3.55730400
C	3.22423400	-2.05063100	-1.00760000
H	2.51441900	-1.54258600	-1.66712600
H	4.23094000	-1.65679400	-1.19366100
H	3.18883400	-3.13004900	-1.20711800
C	0.68912400	-0.80448000	2.98682800
H	0.05319500	-0.09197400	2.44699300
H	0.07849800	-1.66178500	3.30449000
H	1.10947400	-0.30613000	3.86992200

21) TS[10‘-11‘]

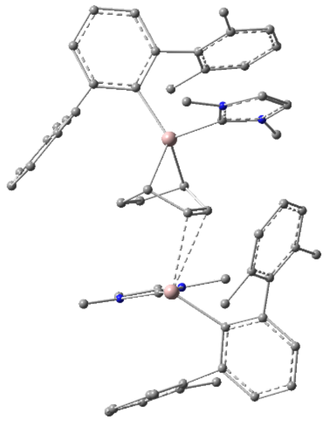

(Hydrogens are omitted for clarity)

Number of imaginary frequencies = 1 (89.8 cm^{-1})

E_{total} = -3023.793632 a.u

$G_{correction}$ = 0.991519 a.u

Cartesian coordinates:

C	-0.29990500	1.65858600	0.83363000
C	-0.64114000	0.24335700	1.23591900
C	0.20397400	-0.71996900	0.45255500
C	0.20611100	-0.49390000	-0.90008200
C	-0.65883400	0.66030600	-1.32136800
C	-0.30099000	1.86504200	-0.50142800
H	-0.03570600	2.42513500	1.56911900
H	-0.67523200	0.06784100	2.31857900
H	0.69627000	-1.58573900	0.89805700
H	0.57880600	-1.22605100	-1.61920200
H	-0.05804700	2.81769300	-0.97475500
H	-0.67238000	0.84592900	-2.39987000
Al	-2.22068400	0.16531100	-0.08521100
C	-3.97892300	1.15745600	-0.04028200
C	-2.68155200	-1.82332600	-0.50661200
C	-5.08581800	0.59556000	0.64194700
C	-4.22466700	2.34581800	-0.77257400
N	-3.14651600	-2.27674900	-1.69147100
N	-2.58051900	-2.92399200	0.26559800
C	-6.37269400	1.13915000	0.53008900
C	-4.93745600	-0.54760600	1.59311700
C	-5.51927600	2.87214500	-0.88740800
C	-3.13283200	3.18283200	-1.36212800
C	-3.35180900	-3.63675800	-1.65903500
C	-2.98950000	-4.04648700	-0.41402500
H	-7.19783100	0.68003200	1.08198300
C	-6.59763900	2.26272900	-0.25636900
C	-5.48950300	-1.80822500	1.28861600
C	-4.34990200	-0.31702700	2.85780000
H	-5.66780000	3.78712300	-1.46768200
C	-2.76209200	3.08640100	-2.71278700

C	-2.54124100	4.16188100	-0.53212700
H	-7.60327200	2.68017300	-0.35145600
C	-5.43851600	-2.82687300	2.24621200
C	-4.32878400	-1.35615300	3.79484000
C	-1.77577800	3.94686700	-3.21301200
C	-1.58564400	5.02373900	-1.07099600
H	-5.86500900	-3.80515300	2.00648100
C	-4.86990900	-2.60288300	3.49584800
H	-3.89190600	-1.17332300	4.78031200
H	-1.47374300	3.85924800	-4.26039400
C	-1.19382300	4.91292700	-2.40362200
H	-1.13335200	5.78512300	-0.42942700
H	-0.43384800	5.58416600	-2.81114400
H	-4.85164800	-3.40142900	4.24201900
H	-3.73082500	-4.19314600	-2.51152700
H	-2.98266600	-5.03534500	0.03543700
C	-3.79684700	1.03080600	3.22251800
H	-3.55927700	1.07919100	4.29426000
H	-2.87734500	1.25216100	2.65870600
H	-4.51067300	1.83269300	2.98256000
C	-6.15372800	-2.06282300	-0.03448100
H	-6.11217600	-3.12923400	-0.29856900
H	-7.21536900	-1.76741900	-0.00956700
H	-5.68699500	-1.47769100	-0.83800400
C	-2.91271200	4.25918400	0.91777100
H	-2.42051600	5.11796700	1.39531600
H	-4.00000300	4.35522000	1.05737200
H	-2.60323100	3.34537600	1.44877800
C	-3.40531600	2.09864200	-3.64385900
H	-4.16055500	1.48949600	-3.13174400
H	-3.90324700	2.61576200	-4.47968900
H	-2.65168900	1.42808400	-4.08691600
C	-2.03534300	-2.93623000	1.60363300
H	-2.24271400	-1.97321600	2.08213000
H	-2.51730500	-3.73022200	2.18533300
H	-0.94908100	-3.09404300	1.56194700
C	-3.40310000	-1.43051900	-2.83393100
H	-4.47447800	-1.19679500	-2.92046300
H	-2.83914000	-0.49861400	-2.70888200
H	-3.05944600	-1.92961300	-3.74951800
Al	2.71841000	0.32021500	-0.84927900
C	4.01955000	-1.21436200	-0.28776400
C	2.72097700	1.37104500	1.01747300
C	5.34081800	-0.85036500	0.10264800
C	3.82066800	-2.59628400	-0.54608100
N	2.80203600	2.72265800	1.07281200
N	2.62823100	0.99134100	2.31607400
C	6.35076100	-1.80187500	0.28513000
C	5.72552200	0.58495900	0.28233800
C	4.83735000	-3.54222800	-0.35403700
C	2.53552000	-3.12140700	-1.09882900
C	2.74942400	3.17722200	2.36801300
C	2.85627800	3.60032700	-0.07519800
C	2.63298600	2.07875500	3.15971200
C	2.49187800	-0.37019000	2.77448500
H	7.34819100	-1.46761000	0.58716300
C	6.10278900	-3.15469600	0.07255000

C	5.89395900	1.13060700	1.57112500
C	5.98579300	1.37955300	-0.85789600
H	4.63080800	-4.59615700	-0.56565000
C	1.64015300	-3.82913300	-0.27219300
C	2.26473800	-2.98420900	-2.47758300
H	2.80737800	4.23264300	2.61836600
H	2.85009800	2.96967500	-0.97986800
H	1.97207800	4.25467500	-0.08686400
H	3.77693300	4.19798100	-0.05064400
H	2.56396500	1.98366100	4.23987600
H	1.43853600	-0.60290000	2.98342500
H	2.86389900	-1.03415800	1.98433400
H	3.08694500	-0.51360200	3.68564500
H	6.89406300	-3.89513400	0.21377500
C	6.21469000	2.48582200	1.70673000
C	5.79706800	0.27464200	2.80161400
C	6.31977500	2.72637600	-0.68389000
C	5.95722600	0.78764900	-2.23782600
C	0.46511800	-4.34973200	-0.82232200
C	1.95014800	-4.04639100	1.18231600
C	1.07812000	-3.51819300	-2.99321500
C	3.22550700	-2.27657300	-3.38553800
H	6.32186300	2.91027300	2.70906900
C	6.41483400	3.28554600	0.58724500
H	5.38474900	0.84103700	3.65018100
H	5.18793200	-0.62031800	2.63039900
H	6.79755700	-0.07954300	3.10226400
H	6.51809800	3.34025300	-1.56678400
H	4.92045300	0.55823400	-2.54229200
H	6.37893300	1.48789500	-2.97248200
H	6.52472600	-0.15398900	-2.27779400
H	-0.22647100	-4.90123800	-0.17914700
C	0.17813100	-4.19088200	-2.17397200
H	2.28947500	-3.11944400	1.66649900
H	1.07354300	-4.42932400	1.72571200
H	2.76839000	-4.77181500	1.31379000
H	0.86885200	-3.40718700	-4.06076900
H	3.19726300	-1.18651800	-3.19768100
H	4.26039800	-2.60317700	-3.20691000
H	2.97294200	-2.44962300	-4.44109900
H	6.67212400	4.34169100	0.70489300
H	-0.74291600	-4.60610100	-2.59014400

22) Intermediate **11‘**

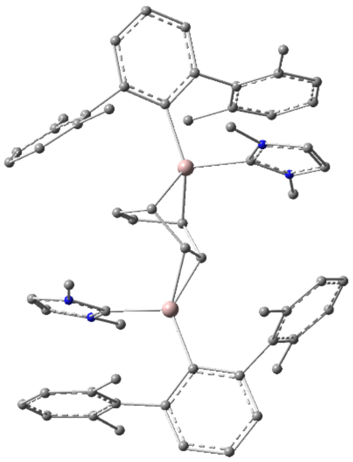

(Hydrogens are omitted for clarity)

Number of imaginary frequencies = 0

E_{total} = -3023.823792 a.u

$G_{correction}$ = 0.990375 a.u

Cartesian coordinates:

C	-0.42517300	1.35678400	1.40443600
C	-0.67033400	-0.11421500	1.43428300
C	0.38450600	-0.89756300	0.62131200
C	0.50057200	-0.33909000	-0.82096900
C	-0.38848800	0.90774400	-0.96898800
C	-0.25872100	1.87439800	0.15578200
H	-0.41490500	1.98488000	2.30317200
H	-0.83455900	-0.51273700	2.44552200
H	0.22050300	-1.98421300	0.64299000
H	0.30137400	-1.08877300	-1.60418100
H	-0.11469100	2.94316300	-0.01123300
H	-0.32728400	1.38399000	-1.95609800
Al	-2.05479600	0.19623000	-0.03917200
C	-3.79479200	1.24247600	-0.05020400
C	-2.56238400	-1.64050600	-0.90511300
C	-5.00914400	0.59333600	0.29389400
C	-3.92168800	2.56552400	-0.55048900
N	-2.85795400	-1.85305100	-2.20612700
N	-2.53974800	-2.86846300	-0.35247600
C	-6.25838500	1.19099800	0.07125700
C	-5.05771000	-0.72059900	1.00553600
C	-5.17797700	3.14645100	-0.77844400
C	-2.76594100	3.49404800	-0.75610800
C	-3.02521200	-3.19406400	-2.46626500
C	-2.82717600	-3.83750700	-1.28390800
H	-7.16511700	0.65373700	0.36341700
C	-6.34977800	2.45731100	-0.49104100
C	-5.61690900	-1.85275300	0.37754400
C	-4.68108600	-0.78070800	2.36631900
H	-5.22307700	4.16748000	-1.16838800
C	-2.17342500	3.68065800	-2.01657000
C	-2.35751800	4.28106300	0.34262900
H	-7.32462400	2.91700100	-0.67245700

C	-5.79203900	-3.02890700	1.11480400
C	-4.88362900	-1.96985500	3.07466200
C	-1.14563700	4.62190000	-2.15425500
C	-1.34239900	5.22383800	0.16736900
H	-6.22463400	-3.90560800	0.62413200
C	-5.44103800	-3.08665500	2.45982900
H	-4.61024400	-2.00934300	4.13254600
H	-0.68100800	4.76183100	-3.13449700
C	-0.72929800	5.38993900	-1.07222100
H	-1.02525000	5.82953500	1.02061000
H	0.06372500	6.13191200	-1.19872700
H	-5.60060400	-4.00502700	3.03075800
H	-3.27428500	-3.57448100	-3.45291000
H	-2.87279600	-4.89262800	-1.03220600
C	-4.11630100	0.41928500	3.07105300
H	-4.06291900	0.24624900	4.15495700
H	-3.10128600	0.65063700	2.71042100
H	-4.72702200	1.31542300	2.88576700
C	-6.05976100	-1.81029100	-1.05802500
H	-5.96865800	-2.80154000	-1.52571400
H	-7.11494100	-1.50162900	-1.13996400
H	-5.47567000	-1.08697100	-1.64135500
C	-2.98164000	4.08480100	1.69231800
H	-2.57710100	4.79827800	2.42389600
H	-4.07536600	4.20191000	1.65569100
H	-2.78565400	3.06376800	2.05419500
C	-2.60487200	2.87440100	-3.20598600
H	-2.10383500	1.89429300	-3.19728900
H	-3.68873600	2.69112600	-3.20102800
H	-2.33884900	3.37825700	-4.14618300
C	-2.26309400	-3.13632100	1.04259400
H	-2.16135200	-2.17607500	1.55961300
H	-3.09614300	-3.69814300	1.48101900
H	-1.32363000	-3.69584100	1.13179000
C	-2.94786300	-0.79583000	-3.18701500
H	-3.41514500	0.08430600	-2.72757700
H	-1.94684500	-0.51796800	-3.54741700
H	-3.56435200	-1.13057800	-4.03087500
Al	2.24608400	-0.34929400	0.13015100
C	3.91336200	-1.42951000	-0.16146000
C	2.60945600	1.51472000	0.86264100
C	5.21854000	-0.88156300	-0.24041800
C	3.81685100	-2.83005200	-0.34205000
N	2.69293800	2.67577600	0.17831600
N	2.63616200	1.87187200	2.16596900
C	6.34318600	-1.68578700	-0.46459100
C	5.48761600	0.58059000	-0.11061100
C	4.94655400	-3.62541000	-0.57334100
C	2.51414500	-3.55716900	-0.30562500
C	2.74401500	3.74735300	1.03678500
C	2.52625200	2.79232200	-1.25277900
C	2.70940900	3.23989000	2.29735100
C	2.41428700	0.95808800	3.26241000
H	7.32964600	-1.21501700	-0.51418200
C	6.21524800	-3.05994900	-0.63093700
C	5.76330800	1.13317400	1.15483300
C	5.55658300	1.37856100	-1.26701600

H	4.81668400	-4.70337600	-0.70708800
C	2.08784800	-4.15723700	0.89455000
C	1.77999500	-3.72962500	-1.49394000
H	2.80026200	4.77282000	0.68403200
H	2.38227200	1.78049000	-1.65736700
H	1.62454100	3.38304100	-1.46916100
H	3.41106900	3.25798000	-1.70417200
H	2.73478700	3.73389000	3.26428900
H	1.58416500	1.32308500	3.88302200
H	2.12308100	-0.01440700	2.83492200
H	3.31762600	0.84805400	3.87794000
H	7.09521800	-3.68340600	-0.80772400
C	6.07018300	2.49268200	1.25202400
C	5.75720300	0.25773700	2.37358600
C	5.89855500	2.72966100	-1.13828000
C	5.27181700	0.78389000	-2.61604600
C	0.95720000	-4.97796400	0.87632500
C	2.83210900	-3.90381600	2.17192300
C	0.64001000	-4.53973000	-1.47259000
C	2.22622500	-3.06771700	-2.76517700
H	6.26856000	2.92776000	2.23536800
C	6.14395700	3.28817800	0.11181800
H	5.80697500	0.85435600	3.29590500
H	4.86491000	-0.38412800	2.40501300
H	6.62048600	-0.42712200	2.36557500
H	5.97032700	3.34958900	-2.03695600
H	5.29157000	1.55114500	-3.40303900
H	6.00829900	0.00654900	-2.87248200
H	4.28815200	0.28816300	-2.63223500
H	0.63779600	-5.46700400	1.80109200
C	0.24548300	-5.18127600	-0.30325000
H	2.81531200	-2.82722200	2.40828000
H	2.38256400	-4.45132700	3.01215200
H	3.89096100	-4.19221100	2.09035400
H	0.06865600	-4.68114300	-2.39381300
H	2.25504400	-1.97300900	-2.64380400
H	3.24423900	-3.38264200	-3.04210900
H	1.54860500	-3.30803900	-3.59663000
H	6.40015700	4.34714500	0.19865900
H	-0.62387100	-5.84413300	-0.30768800

23) Product **4‘**

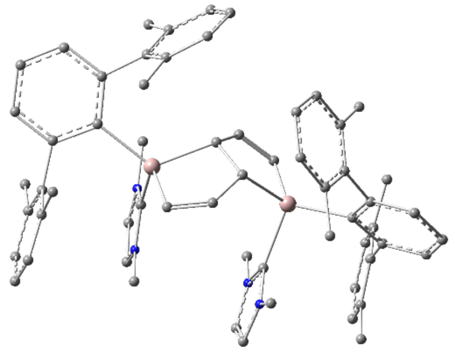

(Hydrogens are omitted for clarity)

Number of imaginary frequencies = 0

E_{total} = -3023.871524 a.u

$G_{correction}$ = 0.990698 a.u

Cartesian coordinates:

Al	2.07896400	0.13253200	-0.19936400
C	1.30584100	0.18973200	-2.00865800
H	1.64459900	-0.06569700	-3.02138400
C	0.07890000	0.76391900	-1.88153100
H	-0.53722400	0.99054100	-2.76909300
C	-0.50897000	1.15438400	-0.58335500
H	-0.93744700	2.16426500	-0.69632200
N	1.74055500	-2.96660000	-0.11545800
C	2.00089400	-1.81811700	0.54419500
N	2.22528700	-2.20131100	1.82167800
C	2.12699400	-3.56584600	1.95877400
C	1.82700600	-4.05243200	0.72359000
C	1.48545900	-3.05967000	-1.53847900
H	2.40620600	-3.34186900	-2.06492300
H	1.15564200	-2.07162700	-1.89044800
H	0.69911900	-3.80364300	-1.71738800
C	2.50305900	-1.28497800	2.90520700
H	3.26310000	-1.71620100	3.57022600
H	1.58630200	-1.06680600	3.46976100
H	2.88928300	-0.34987800	2.48276200
C	4.00443800	0.69090100	0.19519800
C	4.33123900	1.89713400	0.86500900
C	5.65740000	2.23259300	1.17191200
H	5.85883400	3.17520500	1.68858500
C	6.70543800	1.39340000	0.81383200
H	7.73780300	1.66034200	1.05388300
C	6.42207100	0.22176500	0.12192200
H	7.23485200	-0.43535800	-0.20137800
C	5.09956900	-0.12430400	-0.18768400
C	3.29158900	2.90513300	1.23191000
C	2.88031000	3.05926400	2.56861500
C	1.90067000	4.01100000	2.87522400
H	1.56105700	4.11286700	3.90977700
C	1.35985900	4.82149500	1.88555200
C	1.79268900	4.68599700	0.56965800
H	1.36716700	5.31800900	-0.21352500
C	2.74857300	3.72939000	0.22173600
C	3.46881000	2.23542500	3.68102400
H	2.68790100	1.90903200	4.38518900
H	4.20164200	2.82151700	4.26047900
H	3.99930800	1.35189500	3.30387600
C	3.13436800	3.55363100	-1.21791000
H	4.20983800	3.35839600	-1.33552800
H	2.86836100	4.44358800	-1.80547600
H	2.59733700	2.69535700	-1.66002200
C	4.93266100	-1.36213700	-1.00908700
C	4.74108100	-1.23584600	-2.40252200
C	4.69092200	-2.39246600	-3.18742900
H	4.55418200	-2.29458200	-4.26774200
C	4.83164800	-3.65426700	-2.61533300

C	5.02862100	-3.77190900	-1.24268300
H	5.15316500	-4.76001100	-0.79021900
C	5.09755100	-2.63487300	-0.42884800
C	4.66955000	0.11888000	-3.04310400
H	5.58825500	0.69193600	-2.84160400
H	3.83429000	0.70932200	-2.64092700
H	4.54227000	0.03480500	-4.13128400
C	5.40968500	-2.78385300	1.03321900
H	6.49738800	-2.73021400	1.20595800
H	5.06118900	-3.75330900	1.41780300
H	4.96204900	-1.97776300	1.62841000
C	-1.30530700	0.18768600	2.00834000
H	-1.64397000	-0.06870300	3.02085300
C	-0.07870100	0.76268400	1.88160400
H	0.53731100	0.98909800	2.76930200
C	0.50898300	1.15424000	0.58368200
H	0.93706900	2.16421600	0.69723900
Al	-2.07869400	0.13185800	0.19915600
N	-1.74084800	-2.96709200	0.11223700
C	-2.00124100	-1.81799000	-0.54631200
N	-2.22592400	-2.19997800	-1.82409900
C	-2.12773000	-3.56439200	-1.96247900
C	-1.82761600	-4.05214900	-0.72777500
C	-1.48572700	-3.06136300	1.53518700
H	-2.40713500	-3.34126600	2.06172100
H	-1.15325900	-2.07434900	1.88754700
H	-0.70143100	-3.80758600	1.71362700
C	-2.50403100	-1.28263300	-2.90669200
H	-3.26517500	-1.71268100	-3.57122000
H	-1.58767900	-1.06492100	-3.47208500
H	-2.88899800	-0.34749200	-2.48323100
C	-4.00427900	0.69040100	-0.19446400
C	-4.33143100	1.89632600	-0.86461600
C	-5.65778200	2.23167700	-1.17087700
H	-5.85949500	3.17406100	-1.68785800
C	-6.70560700	1.39262400	-0.81185800
H	-7.73810800	1.65946100	-1.05144100
C	-6.42185100	0.22128800	-0.11959300
H	-7.23445900	-0.43567500	0.20446900
C	-5.09917400	-0.12466100	0.18938200
C	-3.29201800	2.90430100	-1.23229800
C	-2.88085300	3.05814200	-2.56903100
C	-1.90180000	4.01042300	-2.87604700
H	-1.56243800	4.11218500	-3.91069900
C	-1.36134300	4.82153900	-1.88676800
C	-1.79392600	4.68618200	-0.57074200
H	-1.36867500	5.31868900	0.21218700
C	-2.74924800	3.72922100	-0.22243900
C	-3.46799400	2.23325900	-3.68143900
H	-2.68463100	1.89751200	-4.37853400
H	-4.19200500	2.82220400	-4.26897500
H	-4.00754500	1.35548100	-3.30373900
C	-3.13477500	3.55370400	1.21731000
H	-4.21020200	3.35837100	1.33517200
H	-2.86874500	4.44379800	1.80465500
H	-2.59759100	2.69556800	1.65949400
C	-4.93185200	-1.36217200	1.01120100

C	-4.73970600	-1.23536800	2.40450100
C	-4.68948300	-2.39168300	3.18986900
H	-4.55226300	-2.29338000	4.27008200
C	-4.83076700	-3.65368000	2.61835700
C	-5.02821700	-3.77184200	1.24581100
H	-5.15311900	-4.76010300	0.79379200
C	-5.09710300	-2.63512900	0.43152400
C	-4.66765700	0.11960400	3.04448600
H	-5.58675100	0.69237300	2.84392000
H	-3.83301500	0.71003600	2.64102600
H	-4.53900400	0.03598200	4.13253900
C	-5.40954700	-2.78461100	-1.03042400
H	-6.49720500	-2.73000100	-1.20311100
H	-5.06199500	-3.75458800	-1.41454400
H	-4.96116400	-1.97927300	-1.62607200
H	1.67624500	-5.07038900	0.37540800
H	2.27824000	-4.07163500	2.90835200
H	-2.27917300	-4.06930000	-2.91249400
H	-1.67685500	-5.07044200	-0.38057600
H	-4.79468400	-4.54749800	3.24668000
H	-0.59823200	5.56144800	-2.14084700
H	4.79559700	-4.54833500	-3.24330500
H	0.59623700	5.56099100	2.13929500

24) TS[7‘-13‘]

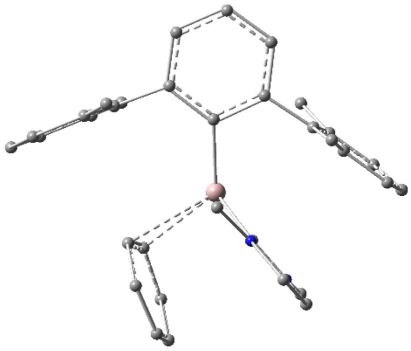

(Selected hydrogens are omitted for clarity)

Number of imaginary frequencies = 1 (108.4 cm^{-1})

E_{total} = -1627.777402 a.u

$G_{correction}$ = 0.528755 a.u

Cartesian coordinates:

Al	-0.14898500	0.32380400	-0.99605300
C	1.64473200	2.19002100	-0.14350700
C	1.18458100	3.20266700	0.71058900
C	0.33463700	4.20060100	0.26055000
C	-0.02229600	4.22250500	-1.11342900
C	0.42643300	3.25363600	-1.97568900
C	1.21263900	2.14733800	-1.50568900
H	2.39727200	1.48413900	0.20834200
H	1.81883100	1.59914700	-2.22999800
H	0.16549800	3.29879600	-3.03601900

H	1.54553600	3.22347900	1.74305800
H	-0.00939000	4.98853800	0.93339900
H	-0.63320000	5.04473600	-1.49768300
C	0.42620500	-1.38885000	-0.05244900
C	1.72924800	-1.89003200	0.19628400
C	-0.64364200	-2.31388900	0.10691800
C	1.93583300	-3.21441500	0.60421100
C	-0.42470100	-3.63186400	0.51689800
C	0.86654300	-4.08839600	0.77011800
H	2.95770000	-3.56085100	0.78470500
H	-1.27960800	-4.30569600	0.62809500
H	1.03834500	-5.12110800	1.08314500
C	-1.32597000	1.29067200	0.42306400
N	-1.27184900	1.36227000	1.77355100
N	-2.43306200	1.99053400	0.08862800
C	-2.32360900	2.09235000	2.27390900
C	-0.20879600	0.81651600	2.58464100
C	-3.06309000	2.48844400	1.20335000
C	-2.90554600	2.18602400	-1.26464900
H	0.48812600	1.60959400	2.88764000
H	-0.62919400	0.33574000	3.47719200
H	0.33079400	0.07523900	1.98232900
H	-2.20461200	1.66404800	-1.93957700
H	-3.90337100	1.74237000	-1.38158500
H	-2.92706900	3.25748700	-1.50438500
C	2.92867000	-1.02077400	0.03311200
C	3.38506200	-0.68979200	-1.26041600
C	3.60985300	-0.53871700	1.17033000
C	4.48361500	0.16575200	-1.39311100
C	4.70420500	0.31555900	0.99824100
C	5.13377500	0.67696500	-0.27448100
H	4.83330900	0.43016600	-2.39477200
H	5.21936400	0.70764400	1.87952900
C	-2.04572400	-1.88893100	-0.20082700
C	-2.49777500	-1.91542100	-1.54117100
C	-2.92109300	-1.49551700	0.83088200
C	-3.78996600	-1.46576900	-1.82922400
C	-4.20372700	-1.03925200	0.50382200
C	-4.63290200	-1.01123200	-0.81828800
H	-4.13763000	-1.48133400	-2.86550300
H	-4.87576100	-0.71855500	1.30487800
C	3.18533900	-0.92917100	2.55845200
H	3.58852200	-1.91721500	2.83464000
H	3.54995600	-0.20502500	3.30142900
H	2.09357600	-1.01116200	2.64587000
C	2.71909000	-1.25435200	-2.48198900
H	3.31862200	-1.06092800	-3.38243000
H	2.56587000	-2.33956000	-2.38545600
H	1.71520900	-0.81541800	-2.63918400
C	-2.52929100	-1.61908900	2.27505500
H	-2.97101200	-0.81325000	2.88017500
H	-2.89435000	-2.57423600	2.68868600
H	-1.44056300	-1.61827200	2.40419000
C	-1.63205500	-2.47975200	-2.63026500
H	-0.69166700	-1.91207400	-2.72069800
H	-1.36314600	-3.52539700	-2.41405500
H	-2.14636200	-2.44298300	-3.60037000

H	-5.63910500	-0.65907200	-1.06120800
H	5.98311100	1.35350700	-0.39540100
H	-2.45904000	2.26986400	3.33725500
H	-3.97504800	3.07557300	1.14575000

25) Intermediate **13'**

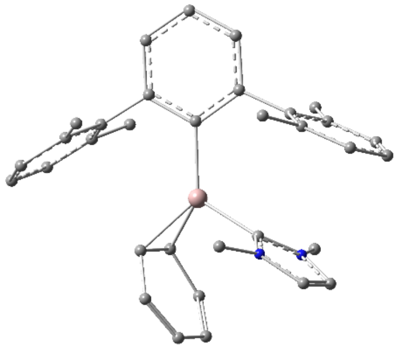

(Selected hydrogens are omitted for clarity)

Number of imaginary frequencies = 0

E_{total} = -1627.794815 a.u

$G_{correction}$ = 0.528884 a.u

Cartesian coordinates:

Al	0.24664100	0.57122400	-0.14649000
C	1.35260300	2.18577800	0.24874900
C	0.77406900	3.35924900	0.89605200
C	-0.12541600	4.17528100	0.29420300
C	-0.41856800	4.02171100	-1.12726600
C	0.19901300	3.06190900	-1.85800800
C	1.03809300	2.01883800	-1.26963200
H	2.38992300	1.97255300	0.53326600
H	1.89464300	1.72756500	-1.89105000
H	0.07914800	3.05757800	-2.94762400
H	1.09818900	3.57918200	1.91999000
H	-0.54900300	5.02627200	0.83460100
H	-1.05430800	4.76319400	-1.61968400
C	0.67795400	-1.36319800	-0.00014800
C	2.02967600	-1.77898800	0.07042000
C	-0.30413300	-2.38158300	0.02690900
C	2.37242300	-3.13176700	0.16007400
C	0.04976200	-3.73264100	0.12148600
C	1.38619600	-4.11361700	0.18664800
H	3.42918400	-3.40908000	0.21151700
H	-0.74024900	-4.48918900	0.14027500
H	1.65759400	-5.16976200	0.25753300
C	-1.65220000	1.19543100	0.22462700
N	-2.14825000	1.60761900	1.41151600
N	-2.63098700	1.44950000	-0.66894500
C	-3.41865600	2.11076000	1.26576800
C	-1.41781800	1.60700000	2.65762200
C	-3.72394600	2.01052900	-0.05498700

C	-2.51520500	1.26575100	-2.09697500
H	-1.36318700	2.62896900	3.05472800
H	-1.89858200	0.94166700	3.38770300
H	-0.39534700	1.26047600	2.45084300
H	-1.53678000	0.81249400	-2.30346400
H	-3.31297000	0.60511400	-2.45836900
H	-2.55800400	2.24157400	-2.59783100
C	3.12956400	-0.77286100	0.05856700
C	3.66163600	-0.33325100	-1.16768700
C	3.62896300	-0.27651500	1.27803600
C	4.68656400	0.61848400	-1.15639000
C	4.66090400	0.66535900	1.25259700
C	5.18458700	1.11508100	0.04374900
H	5.09725300	0.97145900	-2.10607500
H	5.04838000	1.05896100	2.19616000
C	-1.75820000	-2.05874500	-0.04686900
C	-2.40504300	-2.03220100	-1.29592500
C	-2.48270800	-1.82782300	1.13867400
C	-3.77923500	-1.77092200	-1.34082600
C	-3.84834200	-1.54287100	1.05613000
C	-4.49619400	-1.51725100	-0.17591200
H	-4.29048100	-1.76911900	-2.30807900
H	-4.41201100	-1.35175700	1.97344400
C	3.03159000	-0.72241500	2.58062400
H	3.07397600	-1.81605800	2.69549700
H	3.54787100	-0.26105600	3.43395700
H	1.96601500	-0.44391000	2.62860800
C	3.13214300	-0.87385300	-2.46507300
H	3.65718700	-0.43091600	-3.32277800
H	3.24250400	-1.96803000	-2.51675500
H	2.05643500	-0.66136800	-2.57046300
C	-1.79385300	-1.91342700	2.46963800
H	-2.45658500	-1.59179000	3.28572600
H	-1.47365500	-2.94650000	2.67852900
H	-0.87684600	-1.30611600	2.49067100
C	-1.62896200	-2.28728900	-2.55577600
H	-0.75539400	-1.61978100	-2.62714600
H	-1.22858500	-3.31280000	-2.57600600
H	-2.25523700	-2.14471900	-3.44773200
H	-4.61963500	2.28988100	-0.60192200
H	-3.99362800	2.49635500	2.10266700
H	-5.56735800	-1.30635000	-0.22632500
H	5.98430500	1.85962300	0.03737100

26) TS[13‘-10‘]

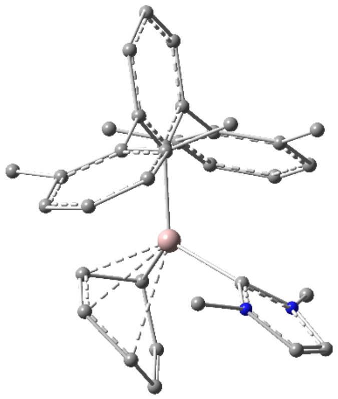

(Selected hydrogens are omitted for clarity)

Number of imaginary frequencies = 1 (131.3 cm^{-1})

E_{total} = -1627.771007 a.u

$G_{correction}$ = 0.529381 a.u

Cartesian coordinates:

Al	0.13527000	0.52822700	0.58395200
C	0.25905000	0.48198600	2.70118700
C	-0.89890200	1.26784800	2.56543800
C	-0.78388300	2.58038600	2.04607900
C	0.48915800	3.25764900	2.22278300
C	1.61512800	2.51007100	2.27747900
C	1.45950600	1.05738800	1.95795100
H	0.29460100	-0.39127500	3.35985500
H	2.37059100	0.46155300	2.05905200
H	2.57662700	2.92112100	2.60181900
H	-1.86783700	0.88964900	2.91363600
H	-1.69327300	3.16382600	1.88556900
H	0.49905900	4.33578800	2.41619000
C	0.09865700	-1.35771800	-0.11653300
C	1.31615600	-2.02852100	-0.39153600
C	-1.08358500	-2.12140000	-0.25779800
C	1.34492600	-3.38507400	-0.73915500
C	-1.04371200	-3.47978100	-0.60035600
C	0.16835300	-4.11912200	-0.83129500
H	2.30849400	-3.86008800	-0.94469300
H	-1.98338300	-4.03260900	-0.68977800
H	0.19462700	-5.17938600	-1.09491300
C	-0.41323600	1.96832100	-0.77134000
N	-1.60692000	2.54426300	-1.06109000
N	0.51263600	2.82046700	-1.27993500
C	-1.42920100	3.74573000	-1.70684100
C	-0.09002400	3.91798800	-1.85223700
C	2.62807800	-1.31767900	-0.37380700
C	3.02962200	-0.59026900	-1.51032900
C	3.48272800	-1.43683800	0.73869200
C	4.27708300	0.04224800	-1.50391700
C	4.71772000	-0.78355100	0.71535900
C	5.11355800	-0.04400900	-0.39547500
H	4.59613600	0.60472400	-2.38620700
H	5.37632000	-0.85704000	1.58492800

C	-2.44720600	-1.53514000	-0.09697400
C	-3.15679400	-1.14170600	-1.25128800
C	-3.06202000	-1.48799500	1.16990000
C	-4.47935500	-0.70880100	-1.12035800
C	-4.37121900	-1.00166800	1.26852800
C	-5.08097800	-0.62490800	0.13235300
H	-5.03652500	-0.41685800	-2.01513700
H	-4.84643800	-0.94973200	2.25178200
C	-2.35030200	-2.01694200	2.37938400
H	-2.88210300	-1.75397200	3.30436800
H	-2.27487100	-3.11528800	2.32850500
H	-1.32214300	-1.63478900	2.43925200
C	-2.49162200	-1.16563100	-2.59717800
H	-2.16671900	-2.18064600	-2.87146700
H	-3.16611400	-0.79343600	-3.38106700
H	-1.58141600	-0.54453400	-2.59406700
C	3.06441400	-2.24276400	1.93448800
H	2.05473800	-1.96228400	2.26985000
H	3.02507000	-3.31789800	1.69782800
H	3.76194300	-2.10280900	2.77192800
C	2.13706200	-0.50960600	-2.71687800
H	2.61846300	0.05052700	-3.53126900
H	1.88405500	-1.51371000	-3.09092400
H	1.17562200	-0.02541300	-2.48178300
C	-2.88146100	2.09384200	-0.55915700
H	-3.28063000	2.82318300	0.16008800
H	-3.59423700	1.95539100	-1.38190500
H	-2.75298100	1.13501300	-0.04712900
C	1.94474700	2.64229200	-1.18530100
H	2.36131900	2.27787900	-2.13461100
H	2.41371200	3.59638500	-0.91405400
H	2.15560100	1.91169400	-0.39030500
H	-2.26381600	4.37455100	-2.00444700
H	0.47988000	4.72195400	-2.30936100
H	6.08333700	0.45958100	-0.40036700
H	-6.11091400	-0.27087300	0.22342200

27) TS[13‘-14‘]

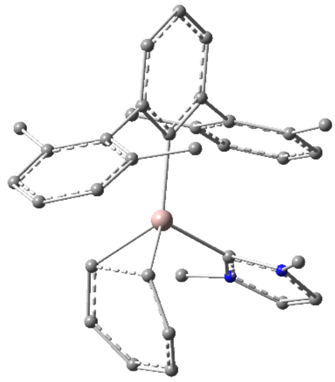

(Selected hydrogens are omitted for clarity)

Number of imaginary frequencies = 1 (402.4 cm^{-1})

E_{total} = -1627.769026 a.u

$G_{correction}$ = 0.525884 a.u

Cartesian coordinates:

Al	-0.28793600	0.66243100	0.22836700
C	-1.44210500	2.09935700	-0.39609600
C	-0.94766000	3.36182000	-0.77375100
C	0.08922300	4.06725100	-0.17029700
C	0.43887100	3.91378400	1.19308400
C	-0.18555700	3.01751900	2.05350400
C	-0.83364000	1.81886100	1.69221700
H	-2.50753500	1.93675700	-0.61210200
H	-1.63620100	1.51246000	2.38147900
H	-0.25230900	3.33288700	3.10494800
H	-1.49932900	3.90554100	-1.55511200
H	0.43890600	4.98098300	-0.66349500
H	1.01728300	4.73023200	1.64097700
C	-0.63540200	-1.29863500	0.00201100
C	-1.96557800	-1.76002200	-0.14739100
C	0.38438400	-2.27774700	-0.03714900
C	-2.25218800	-3.11648900	-0.33978300
C	0.08753200	-3.63286800	-0.23569900
C	-1.22771300	-4.05575800	-0.39077600
H	-3.29469000	-3.42949500	-0.44651000
H	0.90519500	-4.35912200	-0.25720000
H	-1.45378200	-5.11428400	-0.54205400
C	1.58518700	1.22530600	-0.38042500
N	1.92844000	1.47864000	-1.66141400
N	2.67662600	1.54072500	0.34437500
C	3.21705300	1.95308900	-1.74022100
C	3.69072000	1.98987100	-0.46692800
C	1.82473200	-1.94082500	0.16425200
C	2.67793900	-1.79437600	-0.94672700
C	2.33686600	-1.85460900	1.47323700
C	4.03643500	-1.54356200	-0.73303700
C	3.70762000	-1.63857700	1.65033300
C	4.55330800	-1.47649300	0.55732400
H	4.69820600	-1.41566900	-1.59396500
H	4.11223200	-1.59536400	2.66563200
C	-3.11692500	-0.81369000	-0.08223400
C	-3.64959900	-0.45861100	1.17134500
C	-3.67311700	-0.30061000	-1.26894800
C	-4.72669900	0.43191300	1.21964900
C	-4.75992300	0.57390600	-1.18455300
C	-5.28105300	0.94451600	0.05158900
H	-5.13653600	0.72032100	2.19119200
H	-5.19120200	0.97988300	-2.10356900
C	2.14297700	-1.93529500	-2.34228500
H	2.83263500	-1.49883600	-3.07925600
H	2.00117900	-2.99656000	-2.60422500
H	1.15690200	-1.46264600	-2.44813000
C	1.42709400	-2.00134700	2.65921700
H	0.87125800	-2.95060700	2.62227500
H	1.99168200	-1.96296700	3.60114500
H	0.66585000	-1.20405800	2.68063000
C	-3.07131100	-0.64176100	-2.60053400

H	-2.98618300	-1.72857200	-2.74737100
H	-3.66361800	-0.22149200	-3.42517600
H	-2.04916000	-0.23471400	-2.67234900
C	-3.08415500	-1.04242100	2.43465000
H	-3.19104700	-2.13861300	2.44593800
H	-2.00771500	-0.83515000	2.52897000
H	-3.59236500	-0.63656500	3.32028400
C	2.75668900	1.52294000	1.78831900
H	1.86580300	1.02480100	2.18422100
H	3.65607000	0.98022300	2.10210500
H	2.76421700	2.55300100	2.16799300
C	1.02737600	1.39805200	-2.78869900
H	1.54618800	0.95951100	-3.65106700
H	0.16971700	0.77118200	-2.51880400
H	0.64538300	2.39880500	-3.03468000
H	5.62124800	-1.30187700	0.71136100
H	-6.12315500	1.63873200	0.10459700
H	4.65872600	2.28884400	-0.07576500
H	3.68557000	2.21831400	-2.68360300

28) Intermediate **14‘**

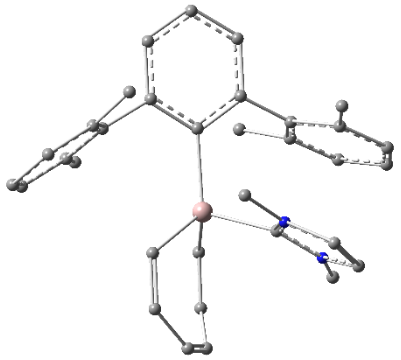

(Hydrogens are omitted for clarity)

Number of imaginary frequencies = 0

E_{total} = -1627.798660 a.u

$G_{correction}$ = 0.527542 a.u

Cartesian coordinates:

Al	0.13980900	0.70874200	-0.48167000
C	1.54068200	2.05162200	-0.08081500
C	1.33265000	3.37677600	-0.28115500
C	0.19140300	4.05756200	-0.87227300
C	-0.67506800	3.68990100	-1.85769400
C	-0.78595700	2.47120200	-2.64336200
C	-0.52023800	1.19133000	-2.28056800
H	2.52969300	1.81926400	0.33643000
H	-0.66819200	0.46743900	-3.09433000
H	-1.18257100	2.66306700	-3.65363900
H	2.11013200	4.08719000	0.04462300
H	0.08525000	5.09990700	-0.54457500
H	-1.34870000	4.49053500	-2.19133500

C	0.52875600	-1.25034600	-0.11734700
C	1.84378500	-1.75131900	0.04903300
C	-0.51902300	-2.20055800	-0.04124700
C	2.08545600	-3.11198300	0.28293000
C	-0.26841200	-3.55691400	0.20136100
C	1.03385200	-4.01676200	0.36484600
H	3.11709400	-3.45754300	0.39352000
H	-1.10914500	-4.25525000	0.24932100
H	1.22802600	-5.07700900	0.54594600
C	-1.25384300	1.31314300	0.94353500
N	-0.93199300	1.45291100	2.24970200
N	-2.51450300	1.78195800	0.84946700
C	-1.97097600	2.00244000	2.96284900
C	0.35753900	1.11105200	2.80976000
C	-2.97712800	2.21069900	2.07171900
C	-3.28593700	1.85865100	-0.37300100
H	0.79940900	0.29638400	2.22347300
H	1.03736300	1.97325900	2.76867200
H	0.22848700	0.77665100	3.84730500
H	-3.17195100	2.85200300	-0.82872300
H	-2.91935200	1.10483400	-1.07636700
H	-4.33976000	1.65868400	-0.14619700
C	3.03237100	-0.85150400	-0.02521900
C	3.44699700	-0.33070100	-1.26771200
C	3.74227300	-0.53326200	1.15041900
C	4.55204800	0.52485500	-1.30890000
C	4.83473800	0.33652500	1.07202600
C	5.23832900	0.86644700	-0.14904200
H	4.87018000	0.93427000	-2.27128500
H	5.37492100	0.59871600	1.98616200
C	-1.94781100	-1.81966800	-0.25817200
C	-2.79323800	-1.57599300	0.84138200
C	-2.46429800	-1.80468600	-1.57041500
C	-4.14455300	-1.29486500	0.61282700
C	-3.81968500	-1.51573500	-1.76327000
C	-4.65712100	-1.26496000	-0.68046500
H	-4.80112000	-1.10479500	1.46685000
H	-4.22173400	-1.50293500	-2.77991800
C	2.71519700	-0.65440500	-2.53821700
H	2.31103700	-1.67627300	-2.53007400
H	1.86512500	0.03369900	-2.68872500
H	3.37723400	-0.54266800	-3.40861400
C	3.36088300	-1.11782400	2.48296900
H	3.84232000	-2.09728500	2.64021000
H	3.67971100	-0.46187800	3.30647400
H	2.27959300	-1.29332900	2.56547500
C	-1.59471000	-2.17451000	-2.73668700
H	-1.43378300	-3.26493000	-2.76293700
H	-2.05606200	-1.87649700	-3.68853800
H	-0.59972200	-1.71613200	-2.66561300
C	-2.27052300	-1.66638900	2.24634000
H	-2.89079400	-1.08101900	2.94059500
H	-2.27023300	-2.71218900	2.59588300
H	-1.23189200	-1.31718100	2.31340800
H	6.09244800	1.54632300	-0.19689500
H	-5.71555100	-1.04765700	-0.84612900
H	-1.91465400	2.18868500	4.03164800

H	-3.97548200	2.61700100	2.20681300

29) Intermediate **15‘**

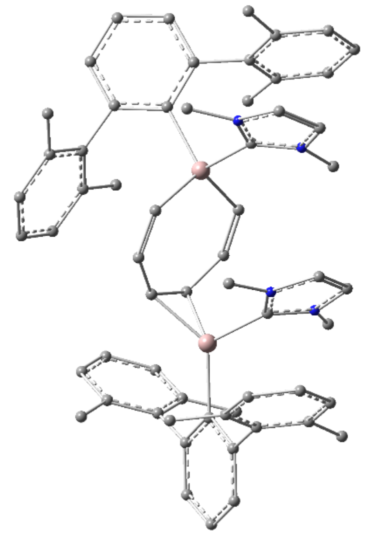

(Hydrogens are omitted for clarity)

Number of imaginary frequencies = 0

E_{total} = -3023.828469 a.u

$G_{correction}$ = 0.986062 a.u

Cartesian coordinates:

Al	-2.45289400	0.13545400	-0.05629800
C	1.06177800	0.46380500	1.74336800
H	1.48699300	0.38416400	2.75636500
C	-0.16326500	1.04393500	1.75418700
H	-0.58455000	1.33585700	2.73139400
C	-1.09948300	1.39167600	0.68651200
H	-1.64227800	2.30949500	0.94747100
N	-1.24321000	-2.55556100	0.82617400
C	-1.74168300	-1.76600200	-0.14763200
N	-1.68073800	-2.51654300	-1.27154500
C	-1.16506800	-3.76152000	-1.00375800
C	-0.88360800	-3.78329700	0.32651700
C	1.13616400	0.00889300	-1.51868500
H	1.59502300	-0.33528100	-2.45848900
C	-0.03880400	0.67333000	-1.72152500
H	-0.37600500	0.79625700	-2.76679200
C	-1.00331200	1.29419600	-0.82827900
H	-1.48160700	2.15891200	-1.30236100
Al	2.14929100	0.04020400	0.16239900
N	3.31973200	-2.31140800	1.78670200
C	2.76893100	-1.91850900	0.61415700
N	2.60746400	-3.06474600	-0.08189600
C	3.05888200	-4.15164200	0.63063900
C	3.50538300	-3.67438400	1.82341700
C	-4.43031100	0.27914200	-0.25000600

C	-5.31297400	-0.74491200	0.16707500
C	-5.01658300	1.44316300	-0.80549300
C	-6.69851600	-0.62300600	0.00162300
C	-6.40194200	1.55629700	-0.96048600
C	-7.24643500	0.52223000	-0.56629900
H	-7.34930700	-1.43485800	0.33915300
H	-6.81549600	2.47227600	-1.39203300
H	-8.32841900	0.61442000	-0.68919400
C	3.89974200	1.10591700	0.10837200
C	5.07885400	0.64380700	-0.52813700
C	3.98810200	2.37886400	0.73155300
C	6.27796300	1.36877500	-0.48594900
C	5.18997000	3.10085900	0.75757200
C	6.34159700	2.59386600	0.16763900
H	7.16167900	0.96967300	-0.99307300
H	5.20662000	4.08355500	1.23792300
H	7.27575300	3.16090600	0.19453200
C	-4.16482000	2.59644600	-1.21721000
C	-3.79384700	3.55847400	-0.25874100
C	-3.73446200	2.71215700	-2.55268800
C	-2.96957000	4.61875000	-0.65037000
C	-2.91669500	3.78708900	-2.91066800
C	-2.52984800	4.73287900	-1.96521000
H	-2.67006100	5.36282200	0.09255100
H	-2.56807900	3.87290600	-3.94321200
C	-4.81574700	-1.97934000	0.84310400
C	-4.71216000	-3.18724100	0.12509000
C	-4.51459700	-1.94095100	2.21963400
C	-4.28740500	-4.34104400	0.79032300
C	-4.11630800	-3.12015000	2.85812300
C	-3.99681900	-4.31227100	2.15084100
H	-4.19531900	-5.27643000	0.23151000
H	-3.89814400	-3.09621300	3.92951000
C	5.10622400	-0.59298800	-1.36830400
C	5.69413600	-1.78114700	-0.89487200
C	4.65038900	-0.51040100	-2.70292600
C	5.77434200	-2.89235400	-1.74343100
C	4.76255100	-1.63327300	-3.52946700
C	5.31614900	-2.82063100	-3.05526200
H	6.22496200	-3.81679600	-1.37054800
H	4.42070600	-1.56544800	-4.56592300
C	2.79864600	3.05262900	1.33623100
C	1.83057800	3.63572600	0.49496100
C	2.67365600	3.14911700	2.73573200
C	0.72173700	4.26041000	1.07248700
C	1.55366800	3.78177000	3.27819100
C	0.57442400	4.32566200	2.45285900
H	-0.04230500	4.68865100	0.41848000
H	1.44553100	3.83691700	4.36521900
C	-4.27093400	3.44711900	1.16117800
H	-3.87560000	4.26721100	1.77638100
H	-5.37051800	3.46958400	1.21534400
H	-3.95546400	2.49439900	1.61394100
C	-4.11430600	1.67155900	-3.56498500
H	-5.20648200	1.57310000	-3.65824300
H	-3.69912800	1.90794800	-4.55448200
H	-3.73900200	0.68132900	-3.26125000

C	-4.62378200	-0.65816100	2.99288400
H	-5.60317700	-0.18041500	2.84067600
H	-4.47830300	-0.82922500	4.06850400
H	-3.86976700	0.07482300	2.65696000
C	-5.07708900	-3.24425700	-1.33033700
H	-4.62879500	-4.12206700	-1.81862800
H	-6.16935200	-3.31315700	-1.46183800
H	-4.76362300	-2.33526200	-1.86193200
C	-1.02374800	-2.14858400	2.19735000
H	-1.73768200	-1.35660300	2.44896900
H	-1.18299000	-3.00776200	2.85929000
H	-0.01467800	-1.72284900	2.29958300
C	-1.98909600	-2.03593400	-2.59940400
H	-2.60673000	-2.76844200	-3.13481000
H	-2.53541400	-1.08844300	-2.50986500
H	-1.05861600	-1.83887800	-3.14986200
C	3.71135200	2.55403200	3.64425800
H	4.67253600	3.08742100	3.57675100
H	3.38096200	2.58137200	4.69253700
H	3.92371800	1.50864400	3.37531800
C	1.96671200	3.58973500	-0.99908200
H	2.98787600	3.84541700	-1.31954700
H	1.74709100	2.58147200	-1.38599200
H	1.26050500	4.28455200	-1.47509900
C	6.29643400	-1.85057000	0.47940300
H	6.22510600	-2.86617100	0.89626100
H	7.36543800	-1.58140800	0.44632700
H	5.81935900	-1.14034200	1.16577700
C	4.12361200	0.78501300	-3.24662700
H	3.80971600	0.67614600	-4.29423900
H	3.26638600	1.14699500	-2.66091500
H	4.89540100	1.56886100	-3.19233600
C	2.13864000	-3.13129700	-1.44994000
H	1.42345500	-2.31331000	-1.61181300
H	2.98477700	-3.01170000	-2.13926700
H	1.65395500	-4.10093900	-1.61623800
C	3.66459300	-1.41270700	2.86599400
H	4.59522000	-1.75003200	3.34067300
H	3.81569500	-0.40846100	2.45180100
H	2.86027000	-1.36935300	3.61358400
H	-3.68256700	-5.22506300	2.66368700
H	-1.87941900	5.56180600	-2.25459000
H	-0.46837200	-4.56544500	0.95509000
H	-1.04600800	-4.52062700	-1.77143100
H	3.94000200	-4.18409700	2.67883400
H	3.03670400	-5.16151300	0.23073300
H	5.39883100	-3.68910600	-3.71405900
H	-0.30484000	4.80758400	2.88857100

30) Intermediate **12‘**

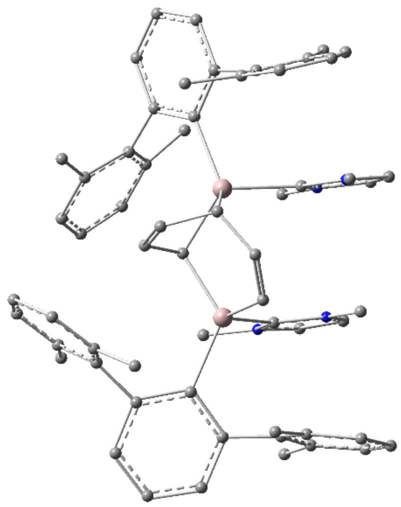

(Hydrogens are omitted for clarity)

Number of imaginary frequencies = 0

E_{total} = -3023.868616 a.u

$G_{correction}$ = 0.996280 a.u

Cartesian coordinates:

Al	-1.70357200	-0.04949600	-0.33861700
C	0.00373200	0.94025900	0.05462600
H	0.08346300	1.52214800	0.98486000
C	-0.23223700	1.82521600	-1.12183600
H	0.05248100	2.87966600	-1.07245000
C	-0.79792200	1.34992000	-2.25886600
H	-0.94029600	2.00995300	-3.12299600
N	-1.68811300	-2.31036600	1.73649600
C	-1.79575800	-2.00419600	0.42396100
N	-2.03839100	-3.18921000	-0.17938400
C	-2.09748000	-4.21163700	0.73759700
C	-1.87475400	-3.65450200	1.95861600
C	0.99727800	-1.30321400	-1.95019000
H	1.58950000	-2.13604400	-2.35819800
C	-0.18753600	-1.11773300	-2.58961600
H	-0.44700100	-1.81765500	-3.40327900
C	-1.23942700	-0.09491200	-2.32666700
H	-2.05011000	-0.21802400	-3.05597200
Al	1.58042800	-0.17469900	-0.42915000
N	2.02395100	-0.84531700	2.53464900
C	1.76970500	-1.34284300	1.30157000
N	1.58537700	-2.66497700	1.50068200
C	1.74553900	-2.99925300	2.82541000
C	2.01822300	-1.83994400	3.48554300
C	-3.47981700	0.77624600	0.31230700
C	-3.60116800	1.86992500	1.21344400
C	-4.70804700	0.25859500	-0.17263400
C	-4.84665900	2.40117700	1.57547200
C	-5.94982200	0.79390800	0.20057600
C	-6.02721500	1.86983100	1.07360900
H	-4.87652500	3.24976400	2.26529000
H	-6.86178800	0.35553100	-0.21576200

H	-6.99494300	2.29107900	1.35734200
C	3.49993000	0.57824000	-0.55534700
C	4.63273000	-0.25703500	-0.34365600
C	3.79585900	1.93980100	-0.82250300
C	5.94004900	0.24889600	-0.31434900
C	5.11149800	2.43234000	-0.79502300
C	6.18625300	1.60007800	-0.51869100
H	6.77314900	-0.44185700	-0.15395000
H	5.28060100	3.48935500	-1.01988600
H	7.20627100	1.99208300	-0.49801500
C	-4.80254600	-0.87697200	-1.13946000
C	-4.73877600	-0.61537500	-2.52271400
C	-5.12917000	-2.16534000	-0.67082200
C	-4.96488600	-1.66517500	-3.42070000
C	-5.37888600	-3.18346300	-1.59670000
C	-5.29258400	-2.93858400	-2.96432600
H	-4.90886300	-1.46729200	-4.49447900
H	-5.64662600	-4.18064300	-1.23471900
C	-2.42566000	2.54103500	1.84528000
C	-1.77484200	3.58998400	1.16551300
C	-2.04586400	2.20661700	3.15885800
C	-0.66857300	4.19718400	1.76333300
C	-0.91838900	2.81949100	3.72022600
C	-0.22018000	3.79645500	3.01909600
H	-0.15006200	4.99321900	1.22666400
H	-0.61132100	2.54782800	4.73496700
C	4.56352300	-1.74933200	-0.25583400
C	4.83851700	-2.41691400	0.95424000
C	4.41399800	-2.48760700	-1.45139000
C	4.88545300	-3.81614000	0.97054600
C	4.47880500	-3.88420600	-1.40104900
C	4.70605800	-4.54929000	-0.19879600
H	5.09167100	-4.33137300	1.91328800
H	4.37097500	-4.45346500	-2.32828300
C	2.79315300	2.95502500	-1.27089500
C	2.35483200	2.93129600	-2.61083900
C	2.42661400	4.02070300	-0.42497500
C	1.56296800	3.98245900	-3.08502200
C	1.63714400	5.05567200	-0.93510100
C	1.20739100	5.04192900	-2.25907700
H	1.22497400	3.96277600	-4.12438100
H	1.36786400	5.89235500	-0.28429400
C	-2.14799500	-3.39288400	-1.60810800
H	-1.15463600	-3.58327600	-2.03522200
H	-2.81978500	-4.23667400	-1.79888200
H	-2.56720100	-2.49317100	-2.06940500
C	-5.25299200	-2.43076800	0.80245200
H	-6.16397900	-1.96707000	1.21345600
H	-5.29564700	-3.50910600	1.01147500
H	-4.41125600	-1.99457700	1.35983300
C	-4.51490300	0.78252700	-3.02007200
H	-3.65996500	1.26266200	-2.52309400
H	-4.34232900	0.79670400	-4.10521800
H	-5.39551700	1.40883900	-2.80291700
C	1.34874300	-3.62240600	0.44134100
H	0.95388200	-3.07990100	-0.42847200
H	2.29120200	-4.11075800	0.16254900

H	0.62063300	-4.36887700	0.78306600
C	-2.89399300	1.30263700	4.00998200
H	-2.29199400	0.76847400	4.76082000
H	-3.63883000	1.90027900	4.56219200
H	-3.46226100	0.57900000	3.41227100
C	-2.30326700	4.08970300	-0.14683400
H	-1.60433500	4.79955300	-0.60990000
H	-2.47154600	3.26641100	-0.85326000
H	-3.27100900	4.59721500	0.00064200
C	2.84323600	4.03879400	1.01749700
H	2.29943000	3.26211200	1.57759000
H	3.91669000	3.83862900	1.14267400
H	2.61225700	5.00661100	1.48504600
C	2.70744800	1.79592700	-3.52458800
H	2.10264600	0.90555500	-3.27966900
H	2.50302900	2.05809100	-4.57212300
H	3.76486100	1.50900600	-3.43055200
C	2.23734200	0.56038000	2.81307700
H	2.90246000	0.66175900	3.68030200
H	2.71237600	1.02544700	1.93974800
H	1.28379700	1.07165200	3.01193600
C	-1.43041700	-1.34466900	2.77847300
H	-1.23218900	-0.36704400	2.31979900
H	-2.29972500	-1.26311800	3.44103000
H	-0.55094200	-1.65090300	3.36034900
C	5.16644800	-1.65330200	2.20613300
H	4.87884200	-2.21806700	3.10456800
H	6.25109300	-1.46440800	2.26822400
H	4.68051800	-0.67029500	2.22515000
C	4.30339800	-1.78506400	-2.77283400
H	5.22351000	-1.21333500	-2.97536000
H	4.15443000	-2.50295700	-3.59138100
H	3.47627700	-1.06355200	-2.78676700
H	0.59580200	5.86064200	-2.64635100
H	4.75941200	-5.64092000	-0.17656500
H	1.66520900	-4.02194400	3.18293800
H	2.21532400	-1.64676600	4.53646800
H	-1.84483300	-4.09420400	2.95158900
H	-2.29317600	-5.24052700	0.44860100
H	-5.48637000	-3.74253800	-3.67906600
H	0.65693100	4.27260200	3.46443800

31) TS[7‘-9‘]

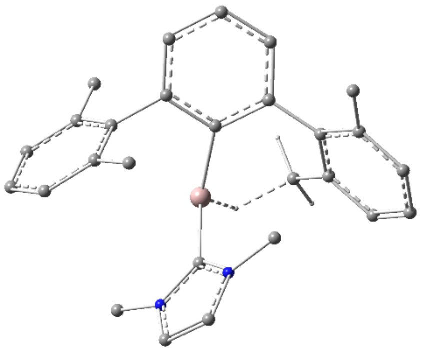

(Selected hydrogens are omitted for clarity)

Number of imaginary frequencies = 1 (1147.6 cm^{-1})

E_{total} = -1395.913822 a.u

$G_{correction}$ = 0.430538 a.u

Cartesian coordinates:

C	0.20225800	-1.17132100	0.24828400
C	1.53411900	-1.66349900	0.13326000
C	1.72581500	-2.97209700	-0.35367800
H	2.72838900	-3.40307500	-0.34906300
C	0.66488600	-3.72931200	-0.83380300
H	0.84556100	-4.74123300	-1.20589200
C	-0.62551100	-3.19631400	-0.85757000
H	-1.45689800	-3.76841000	-1.27862700
C	-0.85103900	-1.93593400	-0.30227800
C	-2.21884500	-1.33144600	-0.28656600
C	-2.57178800	-0.39723200	-1.28453200
C	-3.82457100	0.22358000	-1.22144400
H	-4.09884200	0.94879600	-1.99303400
C	-4.72396500	-0.08681700	-0.20571300
C	-4.38174000	-1.03000400	0.75906100
H	-5.08872100	-1.27892700	1.55478900
C	-3.13560900	-1.66278700	0.73480800
C	2.68461100	-0.76969500	0.39625900
C	2.67804900	0.08862100	1.54433000
C	3.65698700	1.10829800	1.63904900
C	4.63751600	1.25452100	0.67699000
C	4.66663400	0.39244300	-0.42317300
H	5.43380500	0.51442800	-1.19198600
C	3.70452600	-0.61244000	-0.57681800
C	1.72401400	-0.07660000	2.62070500
H	0.27559200	0.48047000	2.83903900
Al	-0.39433200	0.34394200	1.36766800
C	-0.42042500	2.07481100	0.22977700
N	0.36000400	2.50694200	-0.78412700
C	-0.13892300	3.66086300	-1.33446600
C	-1.27730100	3.95845700	-0.64899700
N	-1.42704900	2.97917700	0.30113400
H	-5.70200700	0.40039200	-0.17165100
H	-1.98448800	4.77523400	-0.76344900
H	0.34862400	4.17239700	-2.15988900
H	2.01608300	0.42095300	3.55304100
H	1.40078500	-1.10978400	2.79624200
H	3.64469900	1.76246200	2.51528300
H	5.39489500	2.03664200	0.77953700
C	-2.76114400	-2.65389000	1.79503300
H	-2.46228400	-3.61864500	1.35943600
H	-1.89557600	-2.28507500	2.37107300
H	-3.59219000	-2.82452300	2.49299100
C	-1.63872500	-0.09526800	-2.42278200
H	-1.99151200	0.76587200	-3.00806400
H	-0.61927200	0.10818000	-2.06860700
H	-1.55925100	-0.95964200	-3.10141600
C	3.76500500	-1.42464800	-1.84451400

H	4.21342400	-2.41750700	-1.68364300
H	2.76799900	-1.59856200	-2.27509800
H	4.38396600	-0.90935400	-2.59301100
C	-2.55977400	2.89231200	1.20201700
H	-3.46620200	2.61783500	0.64423300
H	-2.34998900	2.09962400	1.93800800
H	-2.70257500	3.85240600	1.71639500
C	1.56788100	1.83921400	-1.23031800
H	2.42637500	2.10810500	-0.59950800
H	1.42057700	0.75545100	-1.16143300
H	1.76702700	2.11851500	-2.27196700

32) Intermediate **9‘**

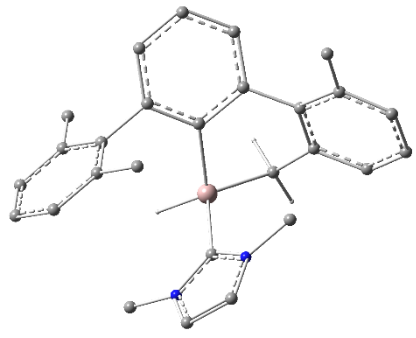

(Selected hydrogens are omitted for clarity)

Number of imaginary frequencies = 0

E_{total} = -1396.016764 a.u

$G_{correction}$ = 0.433242 a.u

Cartesian coordinates:

C	0.23847600	-1.09293000	0.21749400
C	1.55994500	-1.59462800	0.09564500
C	1.75758800	-2.91629700	-0.33264100
H	2.76714100	-3.33164800	-0.35254600
C	0.68511600	-3.70751200	-0.73495300
H	0.85990900	-4.73486300	-1.06485600
C	-0.60414800	-3.18155500	-0.73903700
H	-1.44610500	-3.78076900	-1.09777500
C	-0.82507500	-1.88725500	-0.25306900
C	-2.20754000	-1.31977500	-0.25555200
C	-2.58349300	-0.43774300	-1.28964300
C	-3.87561200	0.09885500	-1.29254300
H	-4.17182700	0.77131500	-2.10288700
C	-4.78434700	-0.22720600	-0.28918000
C	-4.40531100	-1.09412500	0.73114400
H	-5.11565500	-1.35039300	1.52180000
C	-3.12314800	-1.65289600	0.76210400
C	2.73574300	-0.69753700	0.32650600
C	2.78607900	0.16994500	1.45480000
C	3.87632500	1.04366900	1.58917100
C	4.88378800	1.10583600	0.63504800

C	4.80903700	0.29253700	-0.48891400
H	5.57971300	0.35524200	-1.26195100
C	3.74926200	-0.60781400	-0.66044200
C	1.67639500	0.24755900	2.43957700
H	-1.33603100	0.29694700	2.46195500
Al	-0.06501700	0.42246200	1.48026700
C	-0.46475100	2.10436400	0.33757200
N	0.27582600	2.60815500	-0.67639300
C	-0.40568800	3.59950300	-1.33926600
C	-1.61111900	3.72273400	-0.72071000
N	-1.62164100	2.80365100	0.30084900
H	-5.79413700	0.19135700	-0.30565600
H	-2.45111000	4.38180400	-0.92094300
H	0.01758800	4.13284200	-2.18579700
H	1.91131500	0.94610100	3.25517900
H	1.48290200	-0.74244400	2.89605400
H	3.91781000	1.69036800	2.46981500
H	5.72190600	1.79619800	0.76188800
C	-2.72016400	-2.57322900	1.87546000
H	-2.35882400	-3.53888800	1.49235800
H	-1.89386100	-2.13167100	2.45475500
H	-3.55879100	-2.76057700	2.56029000
C	-1.61980300	-0.09297900	-2.38887700
H	-2.07273900	0.59943500	-3.11251900
H	-0.70645800	0.36788300	-1.98520100
H	-1.29193500	-0.99574400	-2.92724200
C	3.71779100	-1.38631100	-1.95091400
H	4.14745800	-2.39473000	-1.84326100
H	2.69509900	-1.52067500	-2.33085200
H	4.31028900	-0.86595900	-2.71758300
C	-2.76504800	2.58276800	1.16548600
H	-3.58163900	2.12429400	0.59010800
H	-2.47170100	1.88483600	1.95875700
H	-3.09141900	3.53749600	1.59984000
C	1.58952600	2.12161900	-1.05496300
H	2.30483700	2.23766100	-0.23051600
H	1.53346000	1.05459900	-1.30811800
H	1.93977500	2.68775400	-1.92558400

33) C-H activation product **8‘**

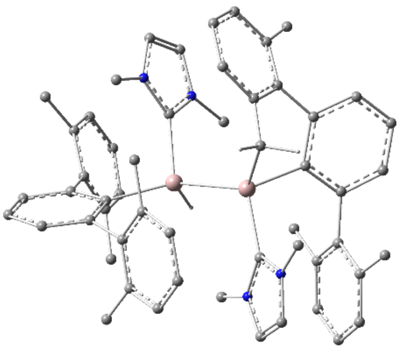

(Selected hydrogens are omitted for clarity)

Number of imaginary frequencies = 0

E_{total} = -2792.065631 a.u

$G_{correction}$ = 0.898652 a.u

Cartesian coordinates:

C	-2.06918400	1.95383100	-0.00800000
C	-1.69157800	3.11513600	-0.74168800
C	-2.33393700	4.34215500	-0.52065200
H	-2.08637800	5.19739400	-1.15271100
C	-3.29129400	4.48384900	0.47738100
H	-3.77933100	5.44775200	0.64292500
C	-3.62286600	3.38188600	1.25477600
H	-4.36919500	3.47260800	2.04978500
C	-3.03645700	2.13089900	1.00592500
C	-0.61806300	3.07395400	-1.78330300
C	-0.62380400	2.04714600	-2.76986000
C	0.33428700	2.09316600	-3.79352400
H	0.29967400	1.32867300	-4.57409200
C	1.30233800	3.09034100	-3.84139400
C	1.34499600	4.05134200	-2.83976700
H	2.12217500	4.82039800	-2.84964500
C	0.39553000	4.06002600	-1.80903000
C	-1.58026600	0.91178400	-2.68959500
Al	-1.29920700	0.23126700	-0.79228700
H	2.02996800	3.11204500	-4.65766000
H	-1.44865500	0.23076000	-3.54300300
H	-2.61748300	1.28863100	-2.69567400
H	0.73987200	-0.15211900	1.87344900
C	-3.55205600	1.00699100	1.84731500
C	-4.76251500	0.37961800	1.48828500
C	-2.90068500	0.64976400	3.04415900
C	-5.28563900	-0.62405200	2.30888200
C	-3.46227900	-0.34467300	3.85344100
C	-4.64151400	-0.98573200	3.48834800
H	-6.21854200	-1.11664300	2.02102100
H	-2.96077000	-0.61455300	4.78725300
H	-5.06721800	-1.76041500	4.13169000
C	-4.39609100	-2.74002700	-0.56879300
C	-3.70499200	-3.03000900	0.56446500
H	-5.28367500	-3.19477900	-0.99891700
H	-3.87345300	-3.78183400	1.32930300
C	-2.67936000	-1.28851200	-0.42418600
N	-3.75162400	-1.67606400	-1.15568800
N	-2.66515500	-2.13770900	0.62712400
Al	1.01182100	-0.28870600	0.27424700
C	2.29105100	-1.85793900	-0.04897900
C	2.22302900	1.36772100	-0.01976400
C	3.55791100	-1.89279200	0.59737700
C	2.02249200	-2.91282800	-0.95430800
N	3.12980200	1.62675500	-0.98913300
N	2.27315500	2.45035700	0.78968900
C	4.51345100	-2.86779100	0.28609200
C	3.89160700	-0.92112400	1.68483700
C	2.99696300	-3.87084000	-1.27464500
C	0.66106700	-3.16189200	-1.52608500
C	3.74543800	2.83937800	-0.78529300
C	3.20883000	3.35629500	0.35067200

H	5.47309000	-2.86274300	0.81083900
C	4.24888100	-3.84057500	-0.67288000
C	3.25226300	-1.05255500	2.93780000
C	4.85896800	0.08571500	1.49311500
H	2.75170100	-4.65908700	-1.99264200
C	-0.14084700	-4.13909000	-0.89775300
C	0.21826700	-2.54606600	-2.71126100
H	4.49929900	3.23198500	-1.46087700
H	3.40825700	4.28682700	0.87330600
H	5.00414100	-4.58871500	-0.92690700
C	3.51045900	-0.10866800	3.93522800
C	5.07925000	1.02400500	2.50825400
C	-1.38709400	-4.45722400	-1.44553200
C	-1.02878900	-2.89687400	-3.23892200
H	3.00873900	-0.20757700	4.90172700
C	4.39725400	0.94189200	3.71641000
H	5.81002800	1.82132800	2.34562700
H	-2.00979600	-5.20946400	-0.95419200
C	-1.83176400	-3.84236500	-2.61075600
H	-1.35893600	-2.42645300	-4.16951100
H	4.57878900	1.68023400	4.50173800
H	-2.80227400	-4.11134800	-3.03578200
C	-4.14831400	-1.09842100	-2.42030500
H	-3.36292400	-1.25512700	-3.16984700
H	-5.07442800	-1.58236700	-2.75347300
H	-4.32605700	-0.02265400	-2.31424800
C	1.06072400	-1.52149300	-3.40277600
H	0.73343200	-1.36847100	-4.44067100
H	0.97497300	-0.55437100	-2.88047000
H	2.12246300	-1.80705900	-3.40526700
C	0.34819700	-4.87257300	0.32120100
H	-0.48170900	-5.37329600	0.84091300
H	1.08552900	-5.64454400	0.04735100
H	0.86172000	-4.20353600	1.02707400
C	3.36656100	0.78451100	-2.13828800
H	3.19829000	-0.26161200	-1.85694200
H	2.68343300	1.06436900	-2.95119600
H	4.40507900	0.90566400	-2.47118100
C	1.44971900	2.63324800	1.96556800
H	0.38841500	2.60904400	1.68353900
H	1.64703100	1.83131600	2.68817600
H	1.68593800	3.60743300	2.41056700
C	0.53278600	5.12791000	-0.75494700
H	-0.04278400	6.03415800	-1.00218100
H	0.17929200	4.78833800	0.22804800
H	1.58548600	5.43248300	-0.66633400
C	-5.50763300	0.80927600	0.25762200
H	-6.12982300	1.69573700	0.46398400
H	-4.81677900	1.10209200	-0.54452400
H	-6.17159600	0.01096500	-0.10546300
C	-1.61874800	1.31416200	3.44930900
H	-1.38291200	1.11025500	4.50362000
H	-0.77761300	0.94127900	2.83985700
H	-1.66919000	2.40286900	3.30239700
C	-1.64912500	-2.16307300	1.65654700
H	-0.80938500	-2.79324000	1.33699500
H	-1.27238100	-1.15230100	1.83944300

H	-2.08676500	-2.55133400	2.58329200
C	2.33156300	-2.20346500	3.22016200
H	2.13360000	-2.28998400	4.29789600
H	1.36939400	-2.05521900	2.70595900
H	2.75637800	-3.15286900	2.86167600
C	5.70911300	0.14302100	0.25586800
H	5.96827400	1.18058300	0.00001000
H	6.65642400	-0.39824300	0.41865600
H	5.22336400	-0.33016500	-0.60464400

2.7. First few mechanistic steps explored for the formation of benzene deconstruction product and C-H activation product using the experimental alumylene 7

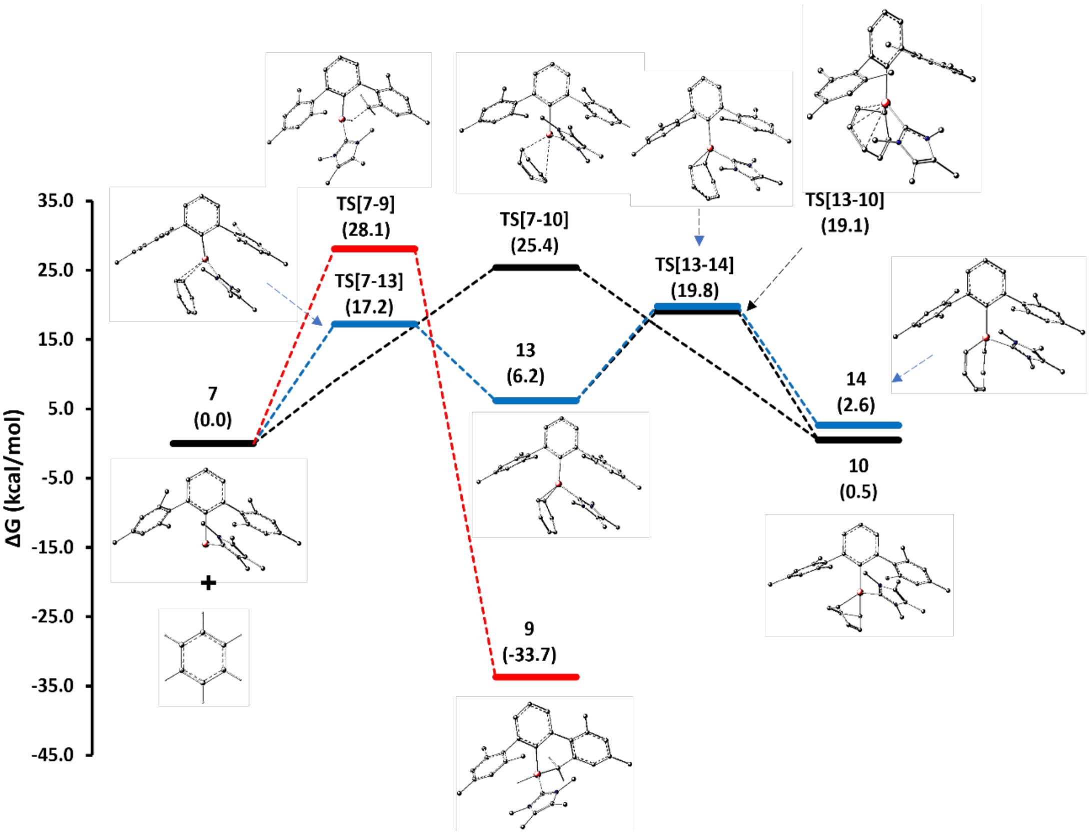

Figure S36. A few mechanistic steps computed for the formation of benzene deconstruction product **4** (black and blue pathways) and the C-H activation product **8** (red pathway) from the hypothesised experimental alumylene **7**. Free energies in parentheses are in kcal/mol.

The free energy profiles obtained using the hypothesized experimental alumylene **7** exhibit a similar trend and energetics to those obtained using the model alumylene **7'**. Thus, this specifies that the model used can reliably represent the mechanistic pathways followed by the experimental system.

Note: Details on structure and energies of species **7**, benzene, **4** and **8** are presented above.

34) TS[7-10]

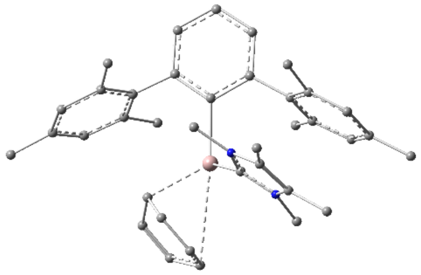

(Hydrogens are omitted for clarity)

Number of imaginary frequencies = 1 (120.6 cm^{-1})

E_{total} = -1784.750405 a.u

$G_{correction}$ = 0.629548 a.u

Cartesian coordinates:

C	-1.75349500	-3.31314800	-2.29961400
C	-0.46473800	-3.62795000	-1.75903900
C	-0.30689100	-3.62172100	-0.35956700
C	-1.17088900	-2.85604600	0.40523200
C	-2.09063100	-1.99906500	-0.31392700
C	-2.59833000	-2.54478300	-1.56122600
H	-2.02405900	-3.66687400	-3.29799400
H	0.25598500	-4.16962300	-2.37752900
H	0.50988900	-4.18115500	0.10742000
H	-1.10657500	-2.82785700	1.49551900
H	-3.58351900	-2.24298000	-1.92269700
H	-2.79790200	-1.42157000	0.29087000
Al	-0.40280300	-0.56531700	-0.73993500
C	-0.59304600	1.40501200	-0.15572100
C	1.19324200	-1.11548700	0.49057900
C	0.51865700	2.28381400	-0.24850000
C	-1.84554300	2.01116700	0.11888900
N	1.31067400	-0.94671600	1.82617600
N	2.36887800	-1.65531100	0.11063800
C	0.38286700	3.66385000	-0.04953500
C	1.89091300	1.79723800	-0.58921500
C	-1.96863300	3.39117900	0.32405000
C	-3.09738000	1.20069600	0.18506500
C	2.54578900	-1.37525400	2.28817900
C	3.22369400	-1.82064700	1.18813400
H	1.26594500	4.30416700	-0.13638200
C	-0.85688900	4.22271900	0.24316800
C	2.88041200	1.71531900	0.40805700
C	2.22922300	1.51073900	-1.93042600
H	-2.95566800	3.81181300	0.53767400
C	-3.66779100	0.86965300	1.42798600
C	-3.73412400	0.79439000	-1.00675200
H	-0.95738200	5.30051700	0.39391800

C	4.16861100	1.29049700	0.06616200
C	3.53271500	1.11235800	-2.23271900
C	-4.83142400	0.09326600	1.46226100
C	-4.90943400	0.04616000	-0.92785900
H	4.92636200	1.21537600	0.85272400
C	4.51716300	0.98288300	-1.24844500
H	3.78776500	0.90036800	-3.27595700
H	-5.25726200	-0.17995800	2.43288900
C	-5.46641200	-0.33391400	0.29604100
H	-5.40030500	-0.26182800	-1.85644200
C	1.21710300	1.65724600	-3.02964600
H	1.69656300	1.61500600	-4.01747800
H	0.46567200	0.84647500	-2.98413900
H	0.66429600	2.60403000	-2.94115200
C	2.58930300	2.12122700	1.82442800
H	3.27814300	1.62978900	2.52704800
H	2.71000900	3.20966500	1.95263200
H	1.55433900	1.89249000	2.10853100
C	-3.14258500	1.11732400	-2.34626900
H	-3.87889800	0.97814300	-3.15006200
H	-2.75850300	2.14679800	-2.38606800
H	-2.28440400	0.45007800	-2.55704900
C	-3.06993600	1.35715100	2.71872900
H	-3.28113800	0.66041200	3.54352400
H	-1.98436800	1.50266300	2.64124900
H	-3.49170700	2.33628600	3.00122000
C	2.69484200	-2.06233800	-1.23630300
H	1.86511400	-1.76164500	-1.89214100
H	3.62522400	-1.58135000	-1.56241500
H	2.79728200	-3.15664100	-1.28856000
C	0.24217000	-0.47624200	2.67234600
H	-0.49139400	0.04444500	2.04462000
H	-0.24886100	-1.31390200	3.19129700
H	0.62633800	0.23107900	3.41896900
C	5.90602200	0.53529000	-1.60657200
H	5.90937000	-0.48580600	-2.02405000
H	6.35336000	1.18935800	-2.37161400
H	6.57066600	0.54145500	-0.73069200
C	4.59983100	-2.35170200	1.04017500
H	5.08891300	-2.42836400	2.01972900
H	4.60679000	-3.35270000	0.58087900
H	5.21342600	-1.69026600	0.40737900
C	2.94216400	-1.30570600	3.71594200
H	2.25242100	-1.87333300	4.36083700
H	3.94698100	-1.72556300	3.85299100
H	2.96135700	-0.26745700	4.08552700
C	-6.69151700	-1.19985500	0.34601400
H	-7.17551600	-1.16086500	1.33239300
H	-7.43069500	-0.89609900	-0.41059400
H	-6.43469400	-2.25318800	0.14402100

35) Intermediate **10**

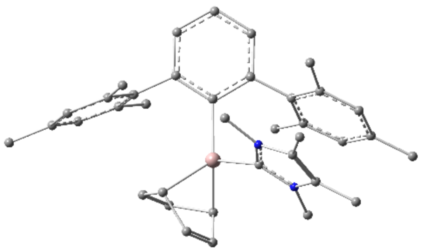

(Hydrogens are omitted for clarity)

Number of imaginary frequencies = 0

E_{total} = -1784.793549 a.u

$G_{correction}$ = 0.632451 a.u

Cartesian coordinates:

C	-1.95924900	-1.04278000	-2.60827600
C	-0.45131400	-1.14403000	-2.58814000
C	-0.06324600	-2.58442000	-2.37795400
C	-0.65893000	-3.16417600	-1.31242100
C	-1.60681900	-2.26346500	-0.57267600
C	-2.55894700	-1.63337300	-1.55346500
H	-2.50333400	-0.56019200	-3.42555200
H	0.04854700	-0.65006200	-3.43124400
H	0.61371100	-3.11671600	-3.05395000
H	-0.50059000	-4.20892200	-1.02585200
H	-3.64068900	-1.66429300	-1.40600200
H	-2.08555500	-2.70960800	0.30635400
Al	-0.34506200	-0.64552900	-0.59509200
C	-0.70846100	1.11909600	0.34589400
C	1.46799900	-1.27816100	0.20856800
C	0.35187000	2.02896700	0.59760100
C	-1.97312600	1.46341000	0.88448300
N	1.66980100	-1.56599200	1.51117900
N	2.64500400	-1.54587400	-0.38738700
C	0.16626400	3.17847100	1.37685400
C	1.70798700	1.85158300	-0.00248900
C	-2.13969700	2.60904900	1.67765200
C	-3.23004500	0.71354000	0.57766300
C	2.96280600	-2.00903300	1.74161700
C	3.58737300	-1.98866500	0.52548200
H	1.00704500	3.86056900	1.53124700
C	-1.07386200	3.46182600	1.93646000
C	2.82669800	1.58890300	0.81055600
C	1.88602800	2.04702000	-1.39108800
H	-3.13329800	2.83425600	2.07536300
C	-3.73604100	-0.27545600	1.43490900
C	-3.97386800	1.10468700	-0.55566600
H	-1.21443900	4.35511300	2.55044800
C	4.09044900	1.46999700	0.22222500
C	3.16807900	1.94138700	-1.93324900
C	-4.96424200	-0.87609600	1.13554900
C	-5.20144400	0.49547000	-0.80947200
H	4.95129100	1.24956300	0.86167200
C	4.28502600	1.64277500	-1.14721900

H	3.29881400	2.10611900	-3.00747800
H	-5.35031100	-1.65043900	1.80589900
C	-5.71352900	-0.50688600	0.01982100
H	-5.77228900	0.80579000	-1.69009900
C	0.72970400	2.41679000	-2.27692500
H	1.08321400	2.74404700	-3.26458500
H	0.04686800	1.56626100	-2.42715700
H	0.13072600	3.22427300	-1.83053300
C	2.70376600	1.46160700	2.30288500
H	3.48590100	0.80142200	2.70583200
H	2.81970100	2.44217000	2.79338500
H	1.71941200	1.08028500	2.60176800
C	-3.44030200	2.14731600	-1.49298200
H	-4.16639700	2.37762400	-2.28499600
H	-3.18724500	3.07982700	-0.96603000
H	-2.51366800	1.78980300	-1.96929000
C	-2.97740800	-0.72007300	2.65209600
H	-2.28526500	-1.53734700	2.39251700
H	-2.38114600	0.09676300	3.08268600
H	-3.65765100	-1.10115800	3.42741400
C	2.89604200	-1.43945000	-1.80523900
H	2.09319300	-0.85266200	-2.26049700
H	3.85181300	-0.93075400	-1.97714100
H	2.91155100	-2.43787200	-2.26517900
C	0.64050200	-1.48060800	2.51963300
H	-0.13600900	-0.78640600	2.17690500
H	0.18501700	-2.46555700	2.70152000
H	1.05946400	-1.09688400	3.45878400
C	-7.01706800	-1.17885100	-0.30193600
H	-7.42464600	-1.70972100	0.57035300
H	-7.77071400	-0.45367100	-0.64462700
H	-6.88977900	-1.91916600	-1.10946600
C	5.64783200	1.51690300	-1.76630200
H	5.69546700	0.67312500	-2.47548500
H	5.91462400	2.42118900	-2.33544900
H	6.42451100	1.35827900	-1.00441100
C	3.46073900	-2.38761800	3.08671300
H	2.85943400	-3.19487000	3.53420400
H	4.49742600	-2.74214900	3.02190600
H	3.44395900	-1.53507100	3.78551600
C	4.98210400	-2.31408800	0.14134400
H	5.50524900	-1.42552600	-0.24849500
H	5.54387000	-2.67907300	1.01067400
H	5.02236700	-3.09283500	-0.63631800

36) TS[7-13]

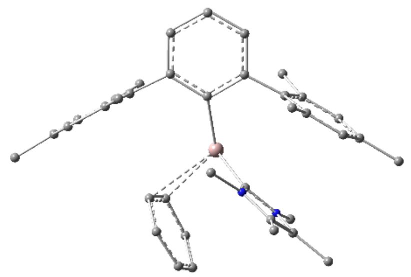

(Hydrogens are omitted for clarity)

Number of imaginary frequencies = 1 (121.0 cm^{-1})

E_{total} = -1784.765423 a.u

$G_{correction}$ = 0.630004 a.u

Cartesian coordinates:

Al	-0.13035100	0.00409200	-1.11437800
C	-1.83181500	-2.05256800	-0.40475600
C	-1.29382200	-3.10556600	0.34633300
C	-0.39104000	-4.00265100	-0.20565900
C	-0.05691600	-3.87252700	-1.57736000
C	-0.58506400	-2.86011500	-2.34176400
C	-1.43521100	-1.86101900	-1.76283200
H	-2.61128300	-1.42378300	0.02560900
H	-2.07989700	-1.27758300	-2.42271100
H	-0.33889500	-2.78955800	-3.40424400
H	-1.63177700	-3.23927200	1.37797100
H	0.01637600	-4.82419500	0.38700600
H	0.60248400	-4.61097500	-2.04315600
C	-0.69793100	1.57316200	0.05869500
C	-2.00634800	1.99598400	0.40408600
C	0.34608100	2.51541000	0.27717200
C	-2.24382900	3.26509000	0.94749000
C	0.09630100	3.77905600	0.81938200
C	-1.20000200	4.16053700	1.15768500
H	-3.26909500	3.55110500	1.20056500
H	0.93147200	4.46973800	0.97087300
H	-1.39535500	5.15138400	1.57504400
C	1.18231400	-1.05951500	0.09576400
N	1.23156800	-1.29975200	1.42437300
N	2.31630300	-1.60739200	-0.39027800
C	2.38213400	-1.98470400	1.77427500
C	0.18506900	-0.95801900	2.35579000
C	3.07919100	-2.17547700	0.61269100
C	2.68137300	-1.61949500	-1.78721400
H	-0.36445200	-1.85769500	2.66590400
H	0.60630200	-0.46838300	3.24284700
H	-0.50381300	-0.27018600	1.85083300
H	1.90478700	-1.05794700	-2.33702700
H	3.64727800	-1.11891900	-1.93446800
H	2.72791300	-2.65199400	-2.16040900
C	-3.17215800	1.08917500	0.20816200
C	-3.69589000	0.87604700	-1.08349600
C	-3.75794800	0.44253500	1.31422200
C	-4.75114300	-0.02462500	-1.24887000

C	-4.81250700	-0.45130700	1.10468800
C	-5.31328300	-0.71375000	-0.17170700
H	-5.14727300	-0.19242100	-2.25521500
H	-5.24885500	-0.96659200	1.96609400
C	1.75270700	2.16240200	-0.09199500
C	2.18990500	2.31600800	-1.42857000
C	2.65495100	1.69974400	0.88289300
C	3.48486800	1.91982000	-1.76890700
C	3.94057400	1.30179000	0.49765200
C	4.37049000	1.38968000	-0.82508200
H	3.81278200	2.03546600	-2.80666200
H	4.62789500	0.92299500	1.26080800
C	-3.27166300	0.70109700	2.71280800
H	-3.68827300	1.64064000	3.11122700
H	-3.57192800	-0.10908000	3.39304300
H	-2.17939700	0.81326700	2.75109300
C	-3.14225100	1.61203500	-2.26941700
H	-3.77828200	1.47106900	-3.15446900
H	-3.05665900	2.68944800	-2.06336300
H	-2.12223200	1.26388200	-2.52134900
C	-6.39998400	-1.72664400	-0.38597400
H	-7.02100800	-1.85095700	0.51304000
H	-7.05748900	-1.44336900	-1.22113300
H	-5.97067600	-2.71315900	-0.62904700
C	2.28616200	1.68447200	2.33787900
H	2.79343200	0.86630500	2.87093600
H	2.59489600	2.62707800	2.82039900
H	1.20248000	1.60039700	2.48271900
C	1.30659000	2.97177800	-2.45130300
H	0.34330100	2.44542400	-2.54169600
H	1.08725000	4.01213900	-2.16290600
H	1.78521000	2.97750200	-3.44025200
C	5.75331400	0.96103600	-1.22702500
H	6.30343800	0.52668800	-0.37971200
H	5.72844000	0.21184500	-2.03553300
H	6.34187800	1.81226300	-1.60554300
C	2.68839600	-2.38290600	3.16996500
H	3.63484100	-2.93725200	3.21045900
H	2.78472500	-1.50884100	3.83460200
H	1.90257200	-3.03114800	3.58960000
C	4.39192400	-2.81482800	0.35468200
H	5.11383900	-2.09332100	-0.06111300
H	4.81464100	-3.21372900	1.28573100
H	4.30665700	-3.64832700	-0.36043900

37) Intermediate **13**

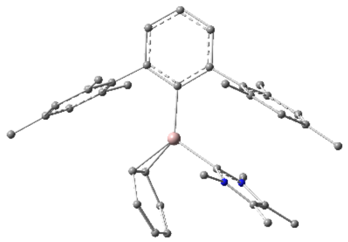

(Hydrogens are omitted for clarity)

Number of imaginary frequencies = 0

E_{total} = -1784.784479 a.u

$G_{correction}$ = 0.630562 a.u

Cartesian coordinates:

Al	0.49802100	0.40329300	-0.06221400
C	1.48489800	2.02213800	0.56838300
C	0.78105100	3.13923000	1.19287300
C	-0.05800100	3.96264200	0.51819800
C	-0.15564100	3.88396000	-0.93593400
C	0.59200000	2.98817900	-1.62568800
C	1.38362100	1.93956500	-0.98646600
H	2.48383200	1.82624900	0.97651300
H	2.32510700	1.70780100	-1.49921600
H	0.61964900	3.04353000	-2.72027800
H	0.95457100	3.30906000	2.26200100
H	-0.58042500	4.76814500	1.04205500
H	-0.74963700	4.63208300	-1.46865600
C	0.97653700	-1.52766200	-0.00358200
C	2.33867300	-1.91361900	0.01838000
C	0.01880500	-2.57048700	0.00866800
C	2.71315600	-3.26108700	0.04192300
C	0.40479900	-3.91539600	0.03302600
C	1.75096200	-4.26688300	0.04721500
H	3.77699600	-3.51471600	0.05826500
H	-0.36741100	-4.69036200	0.04250400
H	2.04845600	-5.31825600	0.06540500
C	-1.45042600	0.96831000	0.02528500
N	-2.14737800	1.33889400	1.11767400
N	-2.27470500	1.21937500	-1.01051900
C	-3.40051900	1.81513500	0.78066300
C	-1.64079800	1.28956400	2.46541300
C	-3.48141700	1.73939600	-0.58256700
C	-1.92674700	1.03228700	-2.39597200
H	-1.67984100	2.28815800	2.92021000
H	-2.21991900	0.57839600	3.07149000
H	-0.59033600	0.97055500	2.42080400
H	-0.88458600	0.68809300	-2.43999000
H	-2.58449000	0.28533200	-2.86113800
H	-1.99866300	1.98673900	-2.93404100
C	3.41593200	-0.88354200	0.02732200
C	3.94660900	-0.40863800	-1.18528700
C	3.91166000	-0.40090600	1.25243100
C	4.95557900	0.55708400	-1.15208300
C	4.92462200	0.56036900	1.24305100
C	5.45446900	1.06017600	0.05117300
H	5.36125300	0.93036000	-2.09741400
H	5.30452800	0.93770600	2.19747800
C	-1.43940900	-2.26346900	0.00753000
C	-2.14577500	-2.19170800	-1.20462300
C	-2.11184200	-2.06583100	1.22865700
C	-3.50869600	-1.87681600	-1.17912600
C	-3.47025300	-1.74612500	1.21238500

C	-4.18579300	-1.63290500	0.01657000
H	-4.05865600	-1.82344300	-2.12456400
H	-3.98927900	-1.58673300	2.16347200
C	3.33317300	-0.88564600	2.55006900
H	3.38052000	-1.98204100	2.63190600
H	3.85941700	-0.44730800	3.40945300
H	2.26800500	-0.61029900	2.61989400
C	3.41828400	-0.91570200	-2.49616400
H	3.95793500	-0.46863200	-3.34265000
H	3.50139600	-2.01087800	-2.56783700
H	2.34831100	-0.67318200	-2.60079000
C	6.50275000	2.13463700	0.06340600
H	7.15432100	2.07429700	-0.82060800
H	6.03676300	3.13437100	0.05898200
H	7.13495500	2.07278100	0.96131800
C	-1.37032300	-2.21364800	2.52602700
H	-2.01165400	-1.97554300	3.38653300
H	-0.99898900	-3.24295300	2.64932200
H	-0.47915400	-1.56815300	2.55634500
C	-1.44196700	-2.45725400	-2.50453700
H	-0.54066800	-1.83308600	-2.60700800
H	-1.09401600	-3.50060900	-2.55873800
H	-2.10088400	-2.27233300	-3.36491400
C	-5.63512600	-1.24320000	0.02782000
H	-6.10987900	-1.41843600	-0.94824200
H	-6.20011900	-1.80623300	0.78631200
H	-5.75081300	-0.17243700	0.26702200
C	-4.38388900	2.26922000	1.79290300
H	-5.30913700	2.60167500	1.30493800
H	-4.64552700	1.45976800	2.49380500
H	-3.99882700	3.11296800	2.38752100
C	-4.57867400	2.08360900	-1.51759700
H	-4.91646500	1.19950300	-2.08252400
H	-5.44175500	2.47946200	-0.96717200
H	-4.26827600	2.84804900	-2.24753800

38) TS[13-10]

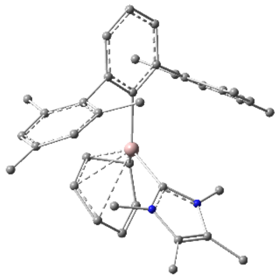

(Hydrogens are omitted for clarity)

Number of imaginary frequencies = 1 (130.3 cm^{-1})

E_{total} = -1784.757324 a.u

$G_{correction}$ = 0.628409 a.u

Cartesian coordinates:

Al	0.17743900	0.08020900	0.74625300
C	0.27344700	-0.40097200	2.81345600
C	-0.89626800	0.37921200	2.82423700
C	-0.79942500	1.77192200	2.58827700
C	0.46002800	2.41714100	2.91611300
C	1.59873200	1.69087300	2.83354500
C	1.47316700	0.33213800	2.22015400
H	0.31704400	-1.38721700	3.28603600
H	2.39350600	-0.25834000	2.21348400
H	2.54738100	2.04061900	3.25421800
H	-1.86250800	-0.07716400	3.07208700
H	-1.71702100	2.36204300	2.53347200
H	0.44941000	3.43220500	3.32836200
C	0.16689200	-1.61999100	-0.33154000
C	1.39421300	-2.19978900	-0.73809200
C	-1.00527100	-2.34921100	-0.64100600
C	1.44183000	-3.44799400	-1.37211600
C	-0.94584800	-3.60006600	-1.27037500
C	0.27525500	-4.15867100	-1.62867900
H	2.41244600	-3.85525700	-1.66959300
H	-1.87789100	-4.13207700	-1.48232600
H	0.31627400	-5.13582900	-2.11649700
C	-0.35343600	1.76435000	-0.29757900
N	-1.54442200	2.37983900	-0.48422100
N	0.57064800	2.70794500	-0.59628800
C	-1.37542000	3.70109800	-0.86275400
C	-0.02722400	3.90947300	-0.94136700
C	2.69541400	-1.49380800	-0.55325500
C	3.10825700	-0.54329500	-1.50253600
C	3.53987900	-1.82717000	0.52334900
C	4.34587000	0.08801600	-1.33950100
C	4.76148900	-1.16759600	0.65658500
C	5.18195100	-0.19911000	-0.26046100
H	4.66675900	0.82520000	-2.08261500
H	5.40591700	-1.41608900	1.50557700
C	-2.37529100	-1.82714900	-0.36095500
C	-3.09637600	-1.20641400	-1.39897300
C	-2.99198100	-2.05176000	0.88631700
C	-4.42030900	-0.81848800	-1.17250500
C	-4.30024500	-1.60406900	1.08669600
C	-5.03668900	-0.99267900	0.06750700
H	-4.97974800	-0.34571100	-1.98604600
H	-4.76842600	-1.76630600	2.06259600
C	6.49124100	0.51253600	-0.07612700
H	6.44733600	1.20775000	0.77854800
H	7.30907100	-0.19572700	0.12794200
H	6.76260600	1.09676300	-0.96702000
C	-6.44996800	-0.54518700	0.30779800
H	-6.50081900	0.20341300	1.11476200
H	-6.89137500	-0.09921700	-0.59470600
H	-7.08940500	-1.38852400	0.61344800
C	-2.27603000	-2.81915000	1.95766400
H	-2.81376800	-2.76977700	2.91474500
H	-2.18283600	-3.87917200	1.67086200
H	-1.25420200	-2.44320400	2.10393200

C	-2.44117700	-0.93319800	-2.72219800
H	-2.09592800	-1.86028600	-3.20405500
H	-3.12855000	-0.41983000	-3.40932100
H	-1.54454100	-0.30604800	-2.58999400
C	3.12002300	-2.86901100	1.51964700
H	2.11186100	-2.66159400	1.90887800
H	3.07401400	-3.86775800	1.05745900
H	3.81836400	-2.91490600	2.36679200
C	2.23640700	-0.21615700	-2.68229400
H	2.72678800	0.50428800	-3.35256500
H	1.99719400	-1.12081700	-3.26233100
H	1.26788400	0.20496500	-2.36772200
C	-2.81780200	1.81854700	-0.11466400
H	-3.25587700	2.38721800	0.71867200
H	-3.51191300	1.82319300	-0.96494600
H	-2.67762000	0.78254800	0.20958100
C	1.99921700	2.51596800	-0.50338300
H	2.45644600	2.43661000	-1.50004200
H	2.45224000	3.34973200	0.04716700
H	2.19255200	1.59112400	0.05920900
C	0.75278900	5.11949200	-1.29791900
H	0.08023800	5.93086500	-1.60454500
H	1.34905800	5.48424000	-0.44581900
H	1.44867300	4.92632100	-2.12976800
C	-2.51867800	4.61438800	-1.10625100
H	-3.12475700	4.76042000	-0.19759400
H	-2.15897600	5.60009500	-1.42786900
H	-3.18916100	4.22871800	-1.89108100

39) TS[13-14]

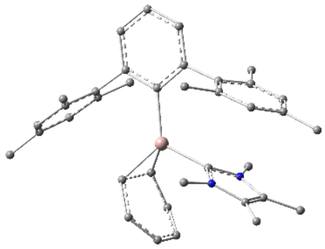

(Hydrogens are omitted for clarity)

Number of imaginary frequencies = 1 (401.7 cm^{-1})

E_{total} = -1784.758521 a.u

$G_{correction}$ = 0.628362 a.u

Cartesian coordinates:

Al	-0.53280400	-0.48133200	0.00065200
C	-1.38128700	-1.84871900	1.10370100
C	-0.75915400	-3.05501700	1.47718200
C	0.12069200	-3.81320500	0.71007000
C	0.11980000	-3.81427600	-0.70463900
C	-0.76100700	-3.05723200	-1.47177400
C	-1.38264900	-1.85036600	-1.09931500
H	-2.35641400	-1.67257100	1.58091700

H	-2.35838600	-1.67491200	-1.57552800
H	-1.07238700	-3.51271400	-2.42350700
H	-1.06930200	-3.50906500	2.43000100
H	0.62446500	-4.65605700	1.19705800
H	0.62295000	-4.65787100	-1.19098400
C	-0.96619700	1.48198500	-0.00040900
C	-2.32440100	1.88176300	-0.00082000
C	-0.00252700	2.51854400	-0.00058500
C	-2.68961400	3.23338900	-0.00143000
C	-0.37754400	3.86791100	-0.00119200
C	-1.72000300	4.23026400	-0.00162900
H	-3.75150700	3.49532800	-0.00174400
H	0.40092000	4.63655400	-0.00132200
H	-2.00885100	5.28435500	-0.00210900
C	1.45368900	-0.98378200	-0.00042200
N	2.21889000	-1.28223700	1.06646300
N	2.21739900	-1.28381700	-1.06793600
C	3.45427300	-1.77088500	0.68216100
C	3.45332100	-1.77189600	-0.68463300
C	1.45747400	2.21798400	-0.00015500
C	2.14889500	2.09746200	1.21826100
C	2.14918100	2.09578000	-1.21830600
C	3.51284800	1.79479800	1.19793300
C	3.51303700	1.79301200	-1.19731800
C	4.21068300	1.62025200	0.00052400
H	4.04903700	1.69844600	2.14788300
H	4.04936700	1.69521400	-2.14704400
C	-3.42105600	0.87114600	-0.00056500
C	-3.94879300	0.40837500	-1.21924500
C	-3.94907800	0.40936700	1.21839100
C	-4.98022600	-0.53296900	-1.19757700
C	-4.98042800	-0.53205100	1.19726100
C	-5.50360300	-1.02453500	-0.00002400
H	-5.38323800	-0.89753500	-2.14747600
H	-5.38362100	-0.89587200	2.14737000
C	1.42763100	2.31064500	2.51827700
H	2.07916200	2.10489100	3.37940200
H	1.06870000	3.34847200	2.60151700
H	0.53092800	1.67688100	2.59277300
C	1.42817000	2.30746900	-2.51870100
H	1.06895900	3.34511300	-2.60299300
H	2.07997300	2.10109100	-3.37947000
H	0.53165600	1.67340200	-2.59280700
C	5.66027300	1.23239800	0.00077700
H	6.18362400	1.61618700	-0.88721600
H	6.18117900	1.60819200	0.89358600
H	5.76984000	0.13422000	-0.00410800
C	-3.39515300	0.90495700	2.52299400
H	-3.44281800	2.00249700	2.59093000
H	-3.94207800	0.47844600	3.37537500
H	-2.33270600	0.63054000	2.62411900
C	-3.39432900	0.90259200	-2.52413200
H	-3.44088900	2.00012500	-2.59282300
H	-2.33213900	0.62702100	-2.62494700
H	-3.94157100	0.47601300	-3.37627600
C	-6.57267000	-2.07827200	0.00034000
H	-7.21455100	-2.00344500	0.89035900

H	-7.21205200	-2.00660500	-0.89172200
H	-6.12549600	-3.08657600	0.00278600
C	1.79921400	-1.16102200	-2.44140900
H	0.75812900	-0.81995700	-2.46011300
H	2.44018500	-0.44486700	-2.97457200
H	1.83664600	-2.14115600	-2.93599600
C	1.80247900	-1.15776700	2.44032900
H	2.44427300	-0.44112800	2.97182700
H	0.76148700	-0.81647200	2.45998000
H	1.84032400	-2.13735500	2.93596900
C	4.50140900	-2.15371900	1.65886700
H	4.80846200	-1.29457300	2.27770600
H	4.15495000	-2.94768400	2.33926300
H	5.39268500	-2.52685300	1.13822600
C	4.49907200	-2.15633100	-1.66220500
H	4.80513500	-1.29830400	-2.28308000
H	5.39114900	-2.52843800	-1.14219900
H	4.15171700	-2.95157100	-2.34066000

40) Intermediate **14**

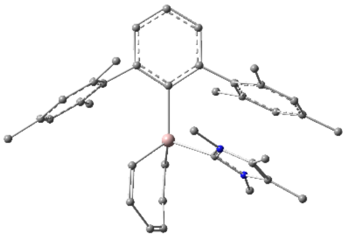

(Hydrogens are omitted for clarity)

Number of imaginary frequencies = 0

E_{total} = -1784.785890 a.u

$G_{correction}$ = 0.628873 a.u

Cartesian coordinates:

Al	0.44973800	-0.49763900	0.67793300
C	1.77107100	-1.86954700	0.13377300
C	1.64087100	-3.16804500	0.50200000
C	0.66281000	-3.78788000	1.38235700
C	0.01682200	-3.32663400	2.48982400
C	0.05415400	-2.03646100	3.15943100
C	0.19510000	-0.79951900	2.61941000
H	2.64953100	-1.67460500	-0.49657100
H	0.21397700	0.00432700	3.36971300
H	-0.09920100	-2.12630000	4.24726100
H	2.35066500	-3.90654200	0.09390700
H	0.51086200	-4.85715300	1.18480700
H	-0.55636000	-4.08903400	3.03398500
C	0.75430900	1.43094300	0.10296500
C	2.05272600	1.92844700	-0.17292200
C	-0.30534100	2.36965200	0.02589900
C	2.27321000	3.28002300	-0.47085200
C	-0.07515700	3.71744500	-0.27759100

C	1.21449800	4.17906900	-0.51763200
H	3.29396100	3.62123700	-0.66534200
H	-0.92341100	4.40701100	-0.32026100
H	1.39207600	5.23355200	-0.74409400
C	-1.20067900	-1.17593600	-0.38084000
N	-1.17567200	-1.31857100	-1.72388600
N	-2.41812900	-1.61742900	-0.01287700
C	-2.36904100	-1.83219300	-2.20366900
C	-0.01499400	-1.05431500	-2.54255700
C	-3.16493800	-2.01891900	-1.10775000
C	-2.88844900	-1.71866200	1.34917400
H	0.64123500	-0.35088100	-2.01677200
H	0.54959400	-1.97965000	-2.72815600
H	-0.31975800	-0.60630800	-3.49697000
H	-2.92236000	-2.77335900	1.65965000
H	-2.18967400	-1.18643600	2.00208900
H	-3.88439500	-1.26912200	1.43677900
C	3.23909700	1.02246200	-0.19407800
C	3.83107900	0.57668000	1.00251200
C	3.77095100	0.61314600	-1.43224200
C	4.91659200	-0.30006100	0.93768900
C	4.85401300	-0.26960400	-1.45272500
C	5.43619900	-0.74779700	-0.27746800
H	5.36543500	-0.64985500	1.87243700
H	5.25269400	-0.59632400	-2.41835500
C	-1.72765600	1.97262300	0.25466000
C	-2.56359600	1.69655300	-0.84265800
C	-2.25748500	1.94950500	1.56048000
C	-3.89971400	1.35103900	-0.61670800
C	-3.59963900	1.60968900	1.74391000
C	-4.43816200	1.30059300	0.66922700
H	-4.54010800	1.12544300	-1.47530800
H	-4.00381800	1.59255500	2.76120000
C	3.30433300	1.00369900	2.34142300
H	2.88284100	2.01807000	2.31091600
H	2.50241200	0.32469300	2.67773000
H	4.09677300	0.97226400	3.10280100
C	3.19931200	1.11484400	-2.72963200
H	3.56078700	2.13047500	-2.95993200
H	3.48757600	0.46271900	-3.56682400
H	2.10281000	1.18526500	-2.69537600
C	6.56708500	-1.73460300	-0.31421600
H	7.06851700	-1.74006800	-1.29279200
H	7.32127600	-1.51271300	0.45578500
H	6.20117200	-2.75735100	-0.12333500
C	-1.41777600	2.36590000	2.73329400
H	-1.37059400	3.46589900	2.79629400
H	-1.83514500	1.99125900	3.67852100
H	-0.38324700	2.01304900	2.63971600
C	-2.05197500	1.81551700	-2.24948700
H	-2.65622200	1.21462200	-2.94504000
H	-2.09366100	2.86289000	-2.59179600
H	-1.00058700	1.51012100	-2.32569100
C	-5.87661400	0.93265400	0.90018100
H	-6.41074700	1.72649000	1.44584300
H	-6.40721300	0.76366700	-0.04792100
H	-5.97072900	0.01618400	1.50693600

C	-4.55894800	-2.51008300	-0.98855400
H	-5.22562800	-1.72877500	-0.58889900
H	-4.94493500	-2.80939700	-1.97142600
H	-4.63006000	-3.38216500	-0.31970900
C	-2.62327700	-2.07808600	-3.64442700
H	-1.88532500	-2.77144300	-4.07815100
H	-3.61811400	-2.51992800	-3.78463400
H	-2.58763100	-1.14590800	-4.23201300

41) TS[7-9]

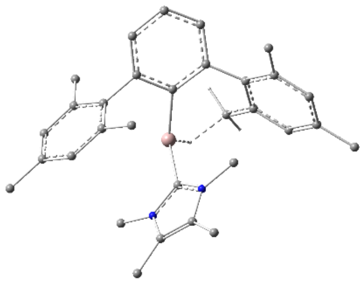

(Selected hydrogens are omitted for clarity)

Number of imaginary frequencies = 1 (1141.2 cm^{-1})

E_{total} = -1552.900593 a.u

$G_{correction}$ = 0.530540 a.u

Cartesian coordinates:

C	0.41050600	-1.60087300	0.17083900
C	1.74619800	-2.02428300	-0.08228800
C	1.94870100	-3.24206800	-0.76206400
H	2.96246900	-3.63176400	-0.87133600
C	0.88453600	-3.95846500	-1.29515100
H	1.07149100	-4.90070100	-1.81716000
C	-0.41743000	-3.46486600	-1.18831700
H	-1.25295300	-3.99035500	-1.65913400
C	-0.64742300	-2.30418400	-0.44744300
C	-2.02270300	-1.72850900	-0.33143800
C	-2.40194900	-0.67874100	-1.19230300
C	-3.67184800	-0.10622100	-1.05307100
H	-3.95909400	0.71009000	-1.72405000
C	-4.58174500	-0.55611600	-0.09604600
C	-4.19274700	-1.60987100	0.73691400
H	-4.88958300	-1.97820700	1.49611000
C	-2.93415100	-2.20428100	0.63667100
C	2.87726100	-1.12458800	0.23361300
C	2.92361000	-0.43612400	1.48848500
C	3.85295800	0.62063800	1.65401300
C	4.75006700	0.97952200	0.66374800
C	4.71987700	0.26838300	-0.54742400
H	5.41102900	0.54697800	-1.34854700
C	3.80814200	-0.76496800	-0.77511300
C	2.05885200	-0.79597700	2.59074600
H	0.60182500	-0.34541000	2.95906000

Al	-0.16629500	-0.28796000	1.52860500
C	-0.41671200	1.55742600	0.62672500
N	0.25472400	2.19279900	-0.35517300
C	-0.43836100	3.29918000	-0.80978600
C	-1.59479300	3.35274200	-0.07753600
N	-1.54633900	2.28302200	0.79583200
H	2.38997300	-0.41441600	3.56367900
H	1.78467200	-1.85657800	2.63759300
H	3.87198000	1.14566500	2.61418700
C	-2.53925000	-3.31608900	1.56136700
H	-2.22054800	-4.20932900	1.00412500
H	-1.68062100	-3.00554800	2.18051000
H	-3.36577400	-3.59326200	2.22997300
C	-1.47855800	-0.19439700	-2.27428800
H	-1.81569200	0.77012700	-2.68133400
H	-0.44913700	-0.08988400	-1.90615700
H	-1.43725100	-0.91853200	-3.10415000
C	3.80776000	-1.38585100	-2.14760500
H	4.30358000	-2.36898500	-2.16074500
H	2.78837000	-1.54520000	-2.52850800
H	4.34954600	-0.74117900	-2.85458300
C	-2.60150000	1.96501400	1.73353500
H	-3.52303900	1.70487500	1.19491800
H	-2.28152900	1.08985000	2.32263700
H	-2.78258300	2.81517700	2.40641200
C	1.53566400	1.77333300	-0.87900300
H	2.32474500	2.48694500	-0.60454300
H	1.79599100	0.79728700	-0.45634900
H	1.48638700	1.68493900	-1.97267900
C	-2.73955300	4.29472300	-0.12937100
H	-2.88659500	4.81525000	0.83028800
H	-2.57316100	5.05676600	-0.90129200
H	-3.67932800	3.77197400	-0.36926600
C	0.07115200	4.17518500	-1.89307300
H	1.05813100	4.59565500	-1.64402500
H	0.17695100	3.62669900	-2.84282700
H	-0.61710800	5.01249100	-2.06492800
C	-5.94521100	0.06063000	0.03561500
H	-6.15093800	0.36834800	1.07339200
H	-6.05383300	0.94409700	-0.61023900
H	-6.73489000	-0.65480400	-0.24607900
C	5.74843100	2.08199100	0.87133700
H	6.77737700	1.68682300	0.89702600
H	5.71187600	2.81771100	0.05148600
H	5.57171200	2.61536100	1.81632700

42) Intermediate **9**

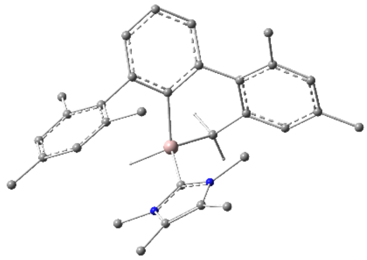

(Selected hydrogens are omitted for clarity)

Number of imaginary frequencies = 0

E_{total} = -1553.003677 a.u

$G_{correction}$ = 0.532657 a.u

Cartesian coordinates:

C	0.43557500	-1.45667400	0.12611300
C	1.75550900	-1.88944800	-0.16059000
C	1.95364600	-3.11142300	-0.82175300
H	2.96886700	-3.48454400	-0.97234600
C	0.87449500	-3.85376800	-1.29316800
H	1.04791300	-4.80449300	-1.80407100
C	-0.42143500	-3.36804200	-1.13932100
H	-1.27164300	-3.91596900	-1.55616700
C	-0.63812600	-2.18288600	-0.42529600
C	-2.02844500	-1.65142000	-0.29124900
C	-2.45937000	-0.63383500	-1.16389800
C	-3.76476000	-0.14268100	-1.05294700
H	-4.09567000	0.63868600	-1.74518600
C	-4.65702400	-0.63362100	-0.09792500
C	-4.20662200	-1.63737100	0.76396200
H	-4.88532700	-2.03306900	1.52602500
C	-2.91261100	-2.15762200	0.68204600
C	2.92283000	-1.00352000	0.13724400
C	3.04235800	-0.32964500	1.38578800
C	4.11261700	0.55481100	1.58027300
C	5.05355200	0.82769400	0.58855100
C	4.89271000	0.19866100	-0.64498400
H	5.59188100	0.42498100	-1.45602600
C	3.85038700	-0.70382200	-0.88940000
C	2.00625900	-0.44979600	2.44461900
H	-0.98788200	-0.55413700	2.68201000
Al	0.19331800	-0.18618100	1.64839000
C	-0.39605500	1.60982100	0.80132900
N	0.21089300	2.28767700	-0.19739200
C	-0.64209900	3.21285400	-0.77298900
C	-1.82438400	3.11661900	-0.09076800
N	-1.63716000	2.13835100	0.86888400
H	2.28319300	0.11784400	3.34434800
H	1.87090700	-1.50619100	2.74678600
H	4.19689500	1.05462400	2.55024000
C	-2.46229800	-3.22949300	1.62941400
H	-2.10423800	-4.11906500	1.09018200
H	-1.62075000	-2.86825300	2.24113500
H	-3.27476700	-3.53318700	2.30406200

C	-1.54160200	-0.09698800	-2.22465800
H	-2.00720700	0.73632100	-2.77004300
H	-0.59312500	0.24716000	-1.78857700
H	-1.27515300	-0.88233000	-2.94959800
C	3.73453500	-1.24687500	-2.29072700
H	4.19796300	-2.24046500	-2.39600600
H	2.68735200	-1.35445500	-2.60698700
H	4.24741600	-0.57757600	-2.99698200
C	-2.66773900	1.73272300	1.80058800
H	-3.54044800	1.35594600	1.25062600
H	-2.27210300	0.91792700	2.41911000
H	-2.95994300	2.58101000	2.43628000
C	1.55434700	2.03322300	-0.66948200
H	2.09922200	1.43439000	0.06784400
H	1.53481000	1.47744000	-1.61867200
H	2.08865100	2.98096700	-0.81490100
C	6.20635900	1.75397700	0.84659500
H	7.05067900	1.21298500	1.30635700
H	6.57847400	2.20683500	-0.08430800
H	5.92815300	2.56410900	1.53725900
C	-6.06396600	-0.11466500	-0.00537200
H	-6.78723500	-0.85677100	-0.38177800
H	-6.34658700	0.10661100	1.03580100
H	-6.19697800	0.80254000	-0.59775800
C	-3.11144400	3.83391500	-0.25981400
H	-3.39443100	4.39018900	0.64792300
H	-3.04374700	4.55321600	-1.08584600
H	-3.93018500	3.13259300	-0.48725300
C	-0.24498400	4.07246500	-1.91476100
H	0.59776300	4.73419600	-1.65800400
H	0.05969400	3.47134000	-2.78623200
H	-1.08497400	4.70774000	-2.22329000

3. References

1 N. Kuhn and T. Kratz, *Synthesis,*1993, 561–562.

2 D. Savaia, E. Tagliavini, C. Trombini and A. Umani-Ronchi, *J. Org. Chem.,* 1981, **46**, 5344–5348.

3 H. Schneider, A. Hock, R. Bertermann and U. Radius, *Chem. Eur. J.,* 2017, **23**, 12387–12398.

4 C. Weetman, A. Porzelt, P. Bag, F. Hanusch and S. Inoue, *Chem. Sci.*, 2020, **11**, 4817-4827

5 G. Sheldrick, *Acta Cryst.,* 2015, **A71**, 3–8

6 G. Sheldrick, *Acta Cryst.,* 2008, **A64**, 112–122.

7 Gaussian 09, Revision E.01, M. J. Frisch, G. W. Trucks, H. B. Schlegel, G. E. Scuseria, M. A. Robb, J. R. Cheeseman, G. Scalmani, V. Barone, B. Mennucci, G. A. Petersson, H. Nakatsuji, M. Caricato, X. Li, H. P. Hratchian, A. F. Izmaylov, J. Bloino, G. Zheng, J. L. Sonnenberg, M. Hada, M. Ehara, K. Toyota, R. Fukuda, J. Hasegawa, M. Ishida, T. Nakajima, Y. Honda, O. Kitao, H. Nakai, T. Vreven, J. A. Montgomery, Jr., J. E. Peralta, F. Ogliaro, M. Bearpark, J. J. Heyd, E. Brothers, K. N. Kudin, V. N. Staroverov, R. Kobayashi, J. Normand, K. Raghavachari, A. Rendell, J. C. Burant, S. S. Iyengar, J. Tomasi, M. Cossi, N. Rega, J. M. Millam, M. Klene, J. E. Knox, J. B. Cross, V. Bakken, C. Adamo, J. Jaramillo, R. Gomperts, R. E. Stratmann, O. Yazyev, A. J. Austin, R. Cammi, C. Pomelli, J. W. Ochterski, R. L. Martin, K. Morokuma, V. G. Zakrzewski, G. A. Voth, P. Salvador, J. J. Dannenberg, S. Dapprich, A. D. Daniels, Ö. Farkas, J. B. Foresman, J. V. Ortiz, J. Cioslowski and D. J. Fox, Gaussian, Inc., Wallingford CT, 2009.

8 (*a*) J. P. Perdew, K. Burke and M. Ernzerhof, *Phys. Rev. Lett.,* 1996, **77**, 3865–3868; (*b*) J. P. Perdew, K. Burke and M. Ernzerhof, *Phys. Rev. Lett.,* 1996, **78**, 1396–1396; (*c*) C. Adamo and V. J. Barone, *Chem. Phys.,* 1999, **110,** 6158–6170; (*d*) M. Ernzerhof and G. E. Scuseria, *J. Chem. Phys.,* 1999, **110**, 5029–5036.

9 F. Weigend and R. Ahlrichs, *Phys. Chem. Chem. Phys.,* 2005, **7**, 3297-3305.

10 (*a*) S. Grimme, J. Antony, S. Ehrlich and H. Krieg, *J. Chem. Phys.,* 2010, **132**, 154104; (*b*) S. Grimme, S. Ehrlich and L. Goerigk, *J. Comput. Chem.*, 2011, **32**, 1456–1465.

11 (*a*) J. D. Queen, A. Lehmann, J. C. Fettinger, H. M. Tuononen and P. P. Power. *J. Am. Chem. Soc.,* 2020, 142, 20554−20559; (*b*) J. Hicks, P. Vasko, J. M. Goicoechea and S. Aldridge, *J. Am. Chem. Soc.,* 2019, **141**, 11000−11003.

12 J. Baker, *J. Comp. Chem.,* 1986, **7**, 385-395.

13 (*a*) A. L. L. East and G. M. Berner, A. D. Morcom and L. Mihichuk, *J. Chem. Theory Comput.,* 2008, **4**, 1274-1282; (*b*) A. Jayaraman and A. L. L. East, *J. Org. Chem.,* 2012, **77**, 351-356; (*c*) S. Nilewar, A. Jayaraman and B. T. Sterenberg, *Organometallics,* 2018, **37**, 4699-4710; (*d*) A. Jayaraman, G. M. Berner, L. M. Mihichuk and A. L. L. East, *J. Mol. Catal. A, 2011*, **351**, 143-153; (*e*) A. Jayaraman, H. Powell-Davies and F.-G. Fontaine, *Tetrahedron,* 2019, **75**, 2118-2127.

14 A. V. Marenich, C. J. Cramer and D. G. Truhlar, *J. Phys. Chem. B,* 2009, **113**, 6378-6396.

15 (*a*) S. F. Boy and F. Bernardi, *Mol. Phys.*, 1970, **19**, 553; (*b*) S. Simon, M. Duran and J. J. Dannenberg, *J. Chem. Phys.*, 1996, **105**, 11024-11031.
